# Host Spin‐Crossover Thermodynamics Indicate Guest Fit

**DOI:** 10.1002/anie.202212634

**Published:** 2022-11-17

**Authors:** Jieyu Zheng, Larissa K. S. von Krbek, Tanya K. Ronson, Jonathan R. Nitschke

**Affiliations:** ^1^ Yusuf Hamied Department of Chemistry University of Cambridge Cambridge CB2 1EW UK; ^2^ Kekulé-Institut für Organische Chemie and Biochemie Rheinische Friedrich-Wilhelms-Universität Bonn Gerhard-Domagk-Str. 1 53121 Bonn Germany

**Keywords:** Host–Guest Chemistry, Metal-Organic Cages, Spin Crossover

## Abstract

Spin‐crossover (SCO) metal‐organic cages capable of switching between high‐spin and low‐spin states have the potential to be used as magnetic sensors and switches. Variation of the donor strength of heterocyclic aldehyde subcomponents in imine‐based ligands can tune the ligand field for a Fe^II^ center, which results in both homoleptic and heteroleptic cages with diverse SCO behaviors. The tetrahedral SCO cage built from 1‐methyl‐1H‐imidazole‐2‐carbaldehyde is capable of encapsulating various guests, which stabilize different cage spin states depending on guest size. Conversely, the SCO tetrahedron exhibits different affinities for guests in different spin states, which is inferred to result from subtle structural differences of the cavity caused by the change in metal center spin state. Examination of SCO thermodynamics across a series of host–guest complexes enabled sensitive probing of guest fit to the host cavity, providing information complementary to binding‐constant determination.

## Graphical abstract

The degree of fit between the binding partners within a host–guest complex is crucial for a highly selective interaction, yet difficult to separate from non‐covalent interactions between the partners. The spin‐crossover of a metallo‐supramolecular host can be used to quantify guest fit, providing a new tool to describe host–guest binding that is complementary to binding constant determination.

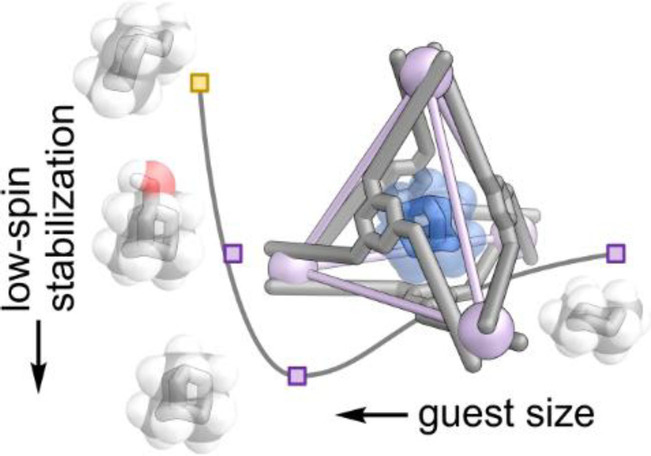

## Introduction

Metal‐organic cages[^1^, ^2^, ^3^, ^4^, ^5^, ^6^, ^7^] have shown great potential for molecular recognition,[^8^, ^9^, ^10^, ^11^] guest stabilization,[^12^, ^13^] and catalysis.[^14^, ^15^, ^16^, ^17^, ^18^, ^19^, ^20^, ^21^] These applications rely on the ability of cage cavities to accommodate guests.[^22^, ^23^, ^24^, ^25^, ^26^, ^27^, ^28^, ^29^, ^30^, ^31^, ^32^, ^33^] Guest‐binding ability depends on factors that include size‐ and shape‐match between host cavity and guest,[^34^, ^35^] charge complementarity,[^36^, ^37^, ^38^] hydrophobic effects,^[39]^ and cavity enclosure.^[40]^ For metal‐organic cages, these aspects mostly derive from the organic ligands used to build the host. The metal vertices can also play a key role. Metal ions with different radii can fine‐tune the cavity volume, and thereby the affinity, for different guests.[^41^, ^42^] For the same metal, different oxidation states can tune the overall charge of the host, thereby also modulating its guest binding abilities.[^43^, ^44^]

Certain transition metal ions, particularly Fe^II^ in an octahedral coordination environment, can adopt either a diamagnetic low‐spin (LS) state or a paramagnetic high‐spin (HS) state. Spin‐crossover (SCO) between these two states may be triggered by external stimuli, such as temperature or light.[^45^, ^46^, ^47^, ^48^, ^49^, ^50^, ^51^, ^52^, ^53^, ^54^] The presence of SCO in cages,[^55^, ^56^, ^57^, ^58^, ^59^, ^60^, ^61^, ^62^, ^63^, ^64^, ^65^] enriches their potential applications, for example as molecular sensors and switches.[^66^, ^67^, ^68^]

The spin states of Fe^II^ impact the structure of their complexes: the Fe^II^−N bond length is approximately 0.2 Å longer in the HS state (≈2.1 Å) than in the LS state (≈1.9 Å).[^69^, ^70^] HS Fe^II^ ions can also exhibit more distorted coordination spheres than their LS counterparts.^[71]^ Thus, when different Fe^II^ spin states are incorporated into a metal‐organic cage, these subtle structural differences may modify the cavity volume and thereby the guest‐binding abilities of the cage.

The Kepert group has reported varying guest sorption affinity in metal–organic frameworks for different spin states.^[72]^ For metal‐organic cages in solution, a similar dependence of the host–guest chemistry on the metal centers’ spin states might be expected. Hence, we prepared a series of SCO metal‐organic cages in order to investigate the effects of metal vertex spin state on guest binding.

We constructed this series of cages using subcomponent self‐assembly, where dynamic‐covalent C=N and coordinative N→Fe^II^ bonds form simultaneously as part of the same overall process.^[73]^ This technique allows the ligand field of a metal center to be tuned through the choice of subcomponent. The use of the subcomponent 2‐formylpyridine to construct Fe^II^ cages usually gives rise to LS Fe^II^ complexes due to the strong ligand field of the pyridylimines.^[73]^

**Scheme 1:**
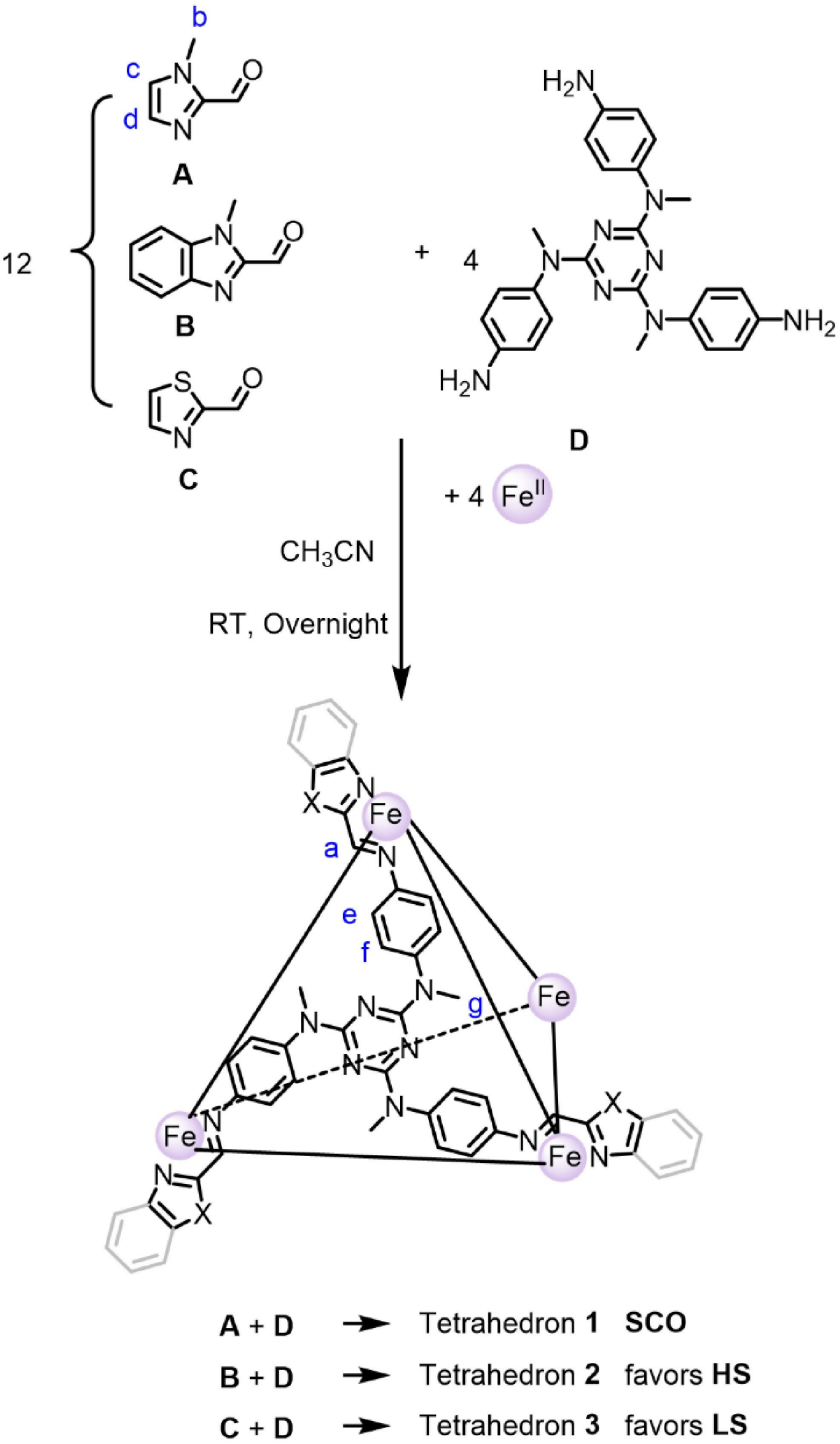
Subcomponent self‐assembly of tetrahedral cages **1**, **2**, and **3** from Fe^II^, azole‐based aldehydes **A**, **B**, and **C**, respectively, and triazine‐containing triamine **D**.

In order to construct metal‐organic cages with SCO properties, a weaker ligand field is required.[^74^, ^75^, ^76^] We thus incorporated three azole‐based aldehyde subcomponents, **A**, **B**, and **C**, to induce SCO in the resulting cages, as shown in Scheme 1. Each of these aldehydes reacted with triamine **D** and iron(II) salts to produce a cage with characteristic SCO behavior.

## Results and Discussion

### Construction of SCO Cages

Tetrahedron **1** was obtained as shown in Scheme 1 from the self‐assembly of triazine‐based triamine **D** (4 equiv), 1‐methyl‐1H‐imidazole‐2‐carbaldehyde **A** (12 equiv), and iron(II) trifluoromethanesulfonate (triflate or ^–^OTf, 4 equiv), and characterized by NMR spectroscopy, mass spectrometry, and X‐ray crystallography. ^1^H NMR spectroscopy indicated *T*‐symmetry, with signals appearing between 0 ppm and 64 ppm at 298 K, consistent with the presence of HS Fe^II^ at room temperature (Figure S2).

**Figure 1:**
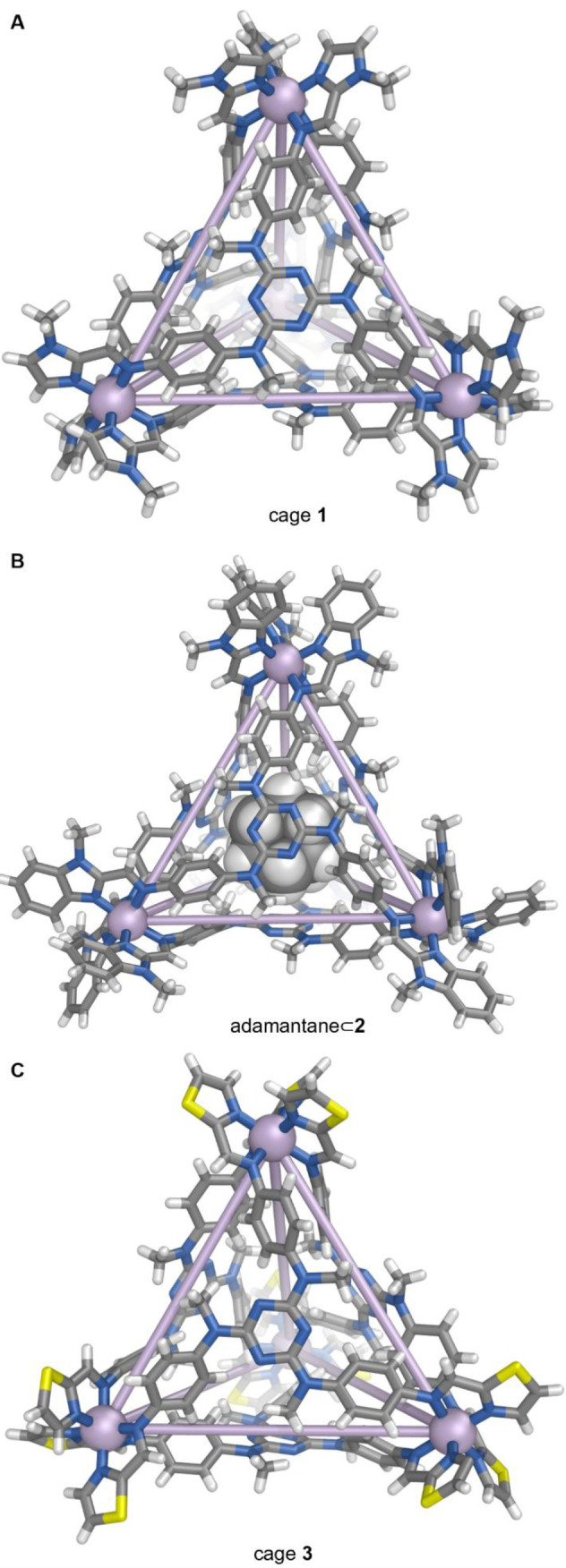
Crystal structures of A) tetrahedron **1**; B) adamantane⊂**2**, and C) tetrahedron **3**. Disorder, solvent of crystallization, and anions are omitted for clarity.

Cage **1** crystallized in the chiral space group *I*23, with one‐twelfth of the cage in the asymmetric unit. All metal vertices within each cage adopt the same Λ or Δ handedness to give a *T*‐symmetric architecture, consistent with the ^1^H NMR spectrum. The X‐ray diffraction data for tetrahedron **1** were collected at 180 K (Figure 1A), with an average Fe−N bond length of 1.98 Å, consistent with LS Fe^II^. Crystallographic evidence of LS Fe^II^ at low temperature, together with room‐temperature ^1^H NMR evidence of HS Fe^II^, led us to infer that **1** might undergo thermally‐controlled SCO.

**Figure 2:**
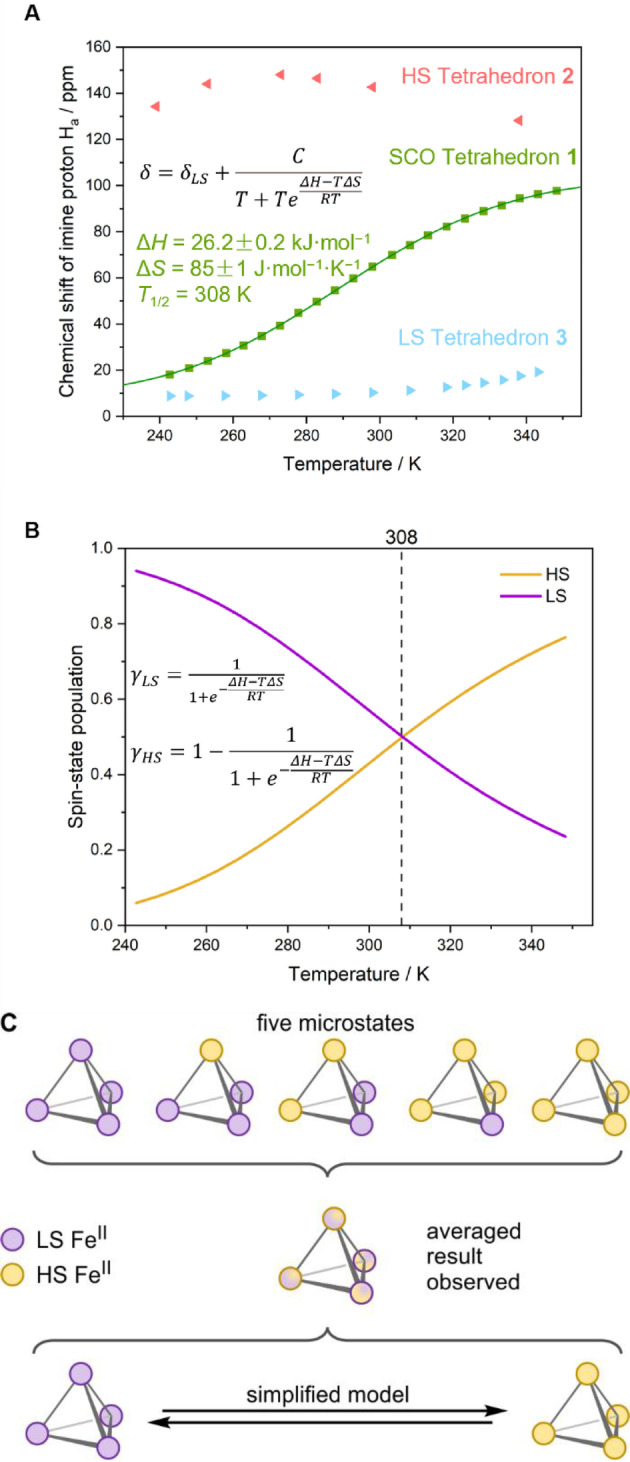
A) ^1^H NMR chemical shifts of the imine protons H_a_ of tetrahedra **1** (▪), **2** (◂), and **3** (▸) in CD_3_CN as a function of temperature, showing that **1** underwent relatively complete SCO, whereas **2** and **3** adopt HS and LS states, respectively. The thermodynamic parameters Δ*H* and Δ*S* of SCO in tetrahedron **1** were obtained by fitting the data to the equation shown,^[77]^ which refers to 1 mol of Fe^II^ centers, and not 1 mol of **1**. B) Spin state populations γ of tetrahedron **1** (LS: purple curve, HS: yellow curve) calculated by fitting the data to the equations shown.^[60]^ The SCO transition temperature *T*_1/2_ of 308 K is illustrated with a vertical dashed line. C) The five microstates of SCO tetrahedron **1** that are shown cannot be distinguished by ^1^H NMR; only their averaged result was observed. Therefore, SCO of tetrahedron **1** was treated as a two state system in (A) and (B).

The SCO of tetrahedron **1** was investigated by variable‐temperature ^1^H NMR spectroscopy (VT NMR) in acetonitrile from 243 K to 348 K. In principle, tetrahedron **1** is able to adopt five individual spin microstates (Figure 2C). Because SCO occurs rapidly on the ^1^H NMR time scale, only an averaged ^1^H NMR signal from these five states is observable in solution (Supporting Information, Figures S69–S70). Hence, we treated SCO as a two‐state process, as a two‐state spin system should reflect the averaged overall trends of the shifting microstate distribution, with the two extremes being the all‐LS/HS states. Since SCO is facilitated in solution when mechanical coupling between metal centers is relatively weak,^[78]^ and the distances between metal centers within **1** are long enough (15.47 Å) not to exhibit cooperativity,^[79]^ an ideal solution model was applied to study the SCO behavior of its metal centers (Supporting Information, Section 5).^[80]^ This model provided the thermodynamic values Δ*H*=26.2±0.2 kJ mol^−1^ and Δ*S*=85±1 J mol^−1^ K^−1^ (Figure 2A) for the two‐state simplified SCO process.

The spin‐state population γ of tetrahedron **1** was calculated based on the thermodynamic parameters from the same model. At 243 K, there was only 6 % HS Fe^II^, approaching 76 % at 348 K (Figure 2B). Thus, tetrahedron **1** underwent nearly complete SCO over the temperature range investigated, with a transition temperature *T*_1/2_ of 308 K.

HS tetrahedral cage **2** was obtained using the same procedure, but with weaker‐field aldehyde 1‐methyl‐1H‐benzimidazole‐2‐carbaldehyde **B** in place of **A** (Scheme 1, Figures 1B and 2A). The additional phenyl ring in **B** is inferred to increase steric hinderance around the metal center, which destabilizes the LS state. The stronger‐field thiazole‐2‐carbaldehyde **C** likewise produced cage **3** (Scheme 1, Figure 1C), which favored LS over the investigated temperature range. The data however suggested an onset of SCO, with a *T*_1/2_ extrapolated out to 414 K (Figures 2A, Supporting Information section 5.8, Figures S100–S103).

**Scheme 2:**
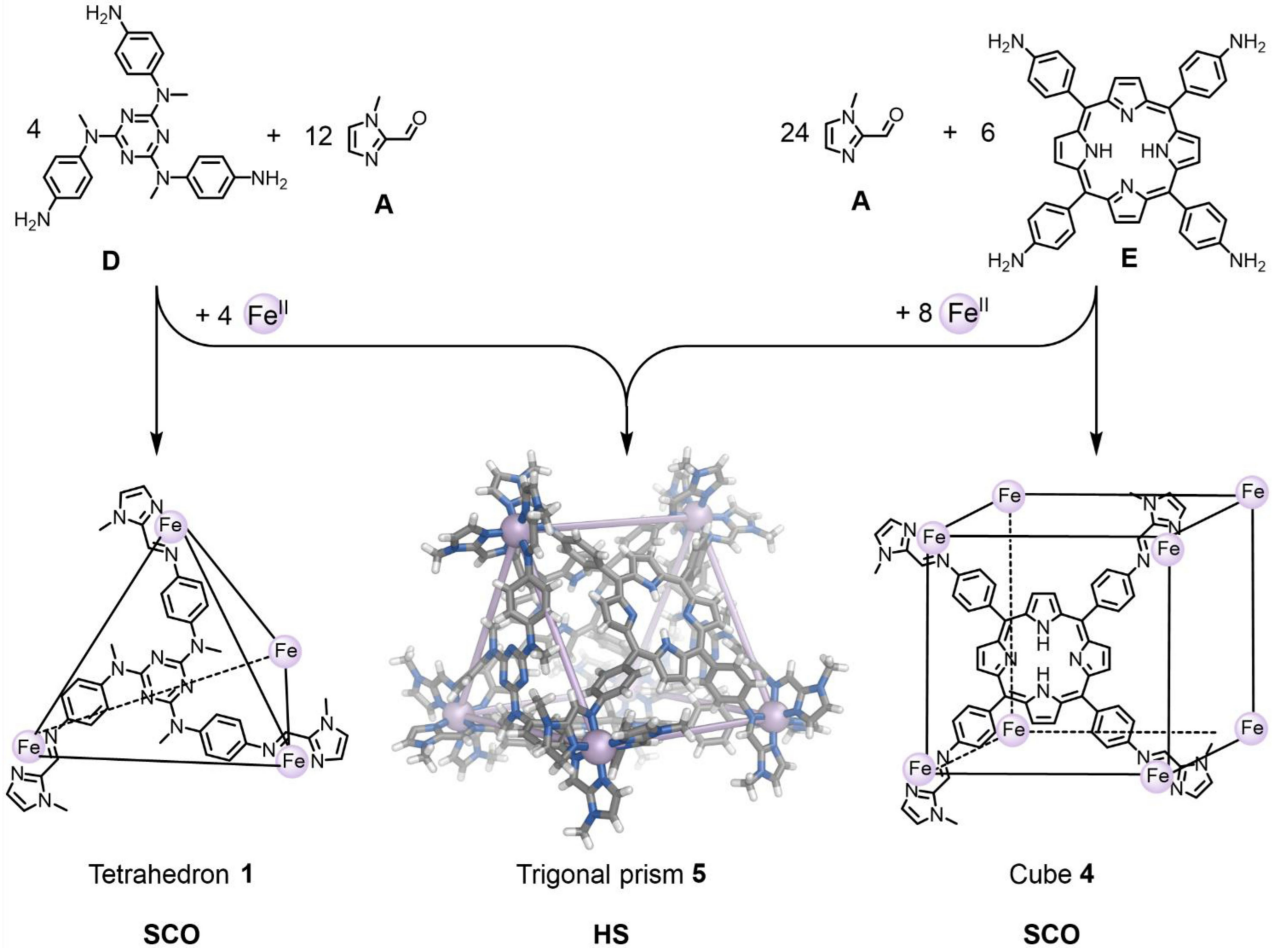
Homoleptic tetrahedron **1** and cube **4** constructed from **A** both exhibit SCO behavior, while heteroleptic trigonal prism **5**, which forms from the same subcomponent library, is HS throughout the investigated temperature range in acetonitrile (reaction conditions for all three cages: CH_3_CN, r.t., overnight, and a mixture of trigonal prism **5**, cube **4**, and a dynamic library is obtained). Disorder, solvent of crystallization and anions are omitted for clarity from the crystal structure of **5**.

Subcomponent **A** and 5,10,15,20‐tetra(4‐aminophenyl)porphyrin **E** formed cubic cage **4** (Scheme 2).[^56^, ^81^] Cube **4** underwent SCO with a *T*_1/2_ of 313 K (Supporting Information section 5.10, Figures S111 and S112) in similar fashion to its methyl‐free analog reported by Lützen et al.^[56]^ Imines formed from aldehyde **A** thus generated a useful ligand field to induce SCO near room temperature in self‐assembled cages.

When all of the subcomponents required to generate tetrahedron **1** and cube **4** were combined, the formation of heteroleptic trigonal prism **5** was observed together with cube **4** (Scheme 2, Figures S20 and S21). Its X‐ray crystal structure revealed **5** to possess the same structural arrangement as a previously reported Zn^II^ trigonal prism^[82]^ with *D*_3_ symmetry, consistent with the solution ^1^H NMR data. Its imine signals appeared above 100 ppm, and showed an upfield shift upon temperature increase, reflecting Curie–Weiss behavior consistent with **5** incorporating HS Fe^II^ vertices at room temperature and above.

The high‐spin character of **5** is noteworthy because homoleptic tetrahedron **1** and cube **4**, built from the same subcomponents, both exhibit SCO behavior, whereas heteroleptic trigonal prism **5** is exclusively HS over the temperature range accessible in acetonitrile. Its HS nature in solution might be attributed to the ability of the more flexible HS coordination sphere to distribute better the strain from differences in the preferred Fe−Fe distances of the two ligands.

The formation of HS **5** instead of a mixture of mixed‐spin **1** and **4** appeared counterintuitive, because the driving force for the reported^[83]^ formation of a LS structure from the rearrangement of a HS precursor was attributed to the formation of shorter, stronger Fe^II^−N bonds in the LS state. Such a driving force may be weaker for the **1**+**4**⇌2×**5** equilibrium because no fully LS structures are involved. Tetrahedron **1** may also be less stable than the other structures involved in this equilibrium, based upon the observation that the integrated intensities of its ^1^H NMR signals are enhanced slightly upon guest binding. Since both trigonal prism **5** and cube **4** were observed, mass balance suggests that the additional subcomponents capable of forming tetrahedron **1** are present in the mixture according to the aforementioned equilibrium between the three species. These subcomponents may combine with small amounts of **E** to produce multiple low‐symmetry structures, which gave rise to small, broad NMR signals. We thus infer the presence of a dynamic library of components, that form additional **1** due to the extra stabilization provided by guest binding (Figure S22). The formation of a Zn^II^ trigonal‐prismatic^[82]^ analog of **5** was also shown to be favored entropically; the more loosely‐bound HS Fe^II^ centers of **5** may lend strength to an entropic driving force for its formation, in preference to **1** and **4**.

### Interplay Between SCO and Host‐Guest Chemistry

We previously reported that small guests had a limited effect upon host SCO behavior,[^56^, ^60^] but detailed knowledge of the interplay between SCO and host–guest binding has not yet been obtained. Since triazine‐paneled cages have been shown to bind diverse guests in acetonitrile, with strong affinities for cyclic hydrocarbons,[^84^, ^85^, ^86^] investigations of the host–guest chemistry of **1** were carried out with cyclohexane, adamantane, 1‐adamantanol, and *cis*‐decalin.

These four guests range in volume from 110 to 169 Å^3^ (Table S3; Supporting Information Section 3.6). Their molecular volumes were calculated^[87]^ to include the internal void spaces of these guests, which must be bound along with the guests.

Furthermore, the sphericity Ψ^[88]^ of the guest was anticipated to impact binding, with higher sphericities corresponding to a better match to the near‐spherical cavity of **1**. 1‐Adamantanol and *cis*‐decalin have near‐identical molecular volumes (169 and 167 Å^3^, respectively). However, 1‐adamantanol is more compact than *cis*‐decalin, with a higher sphericity. We therefore corrected guest volumes for sphericity (*V*_mol_ Ψ^−1^) in the comparisons made below.

**Figure 3:**
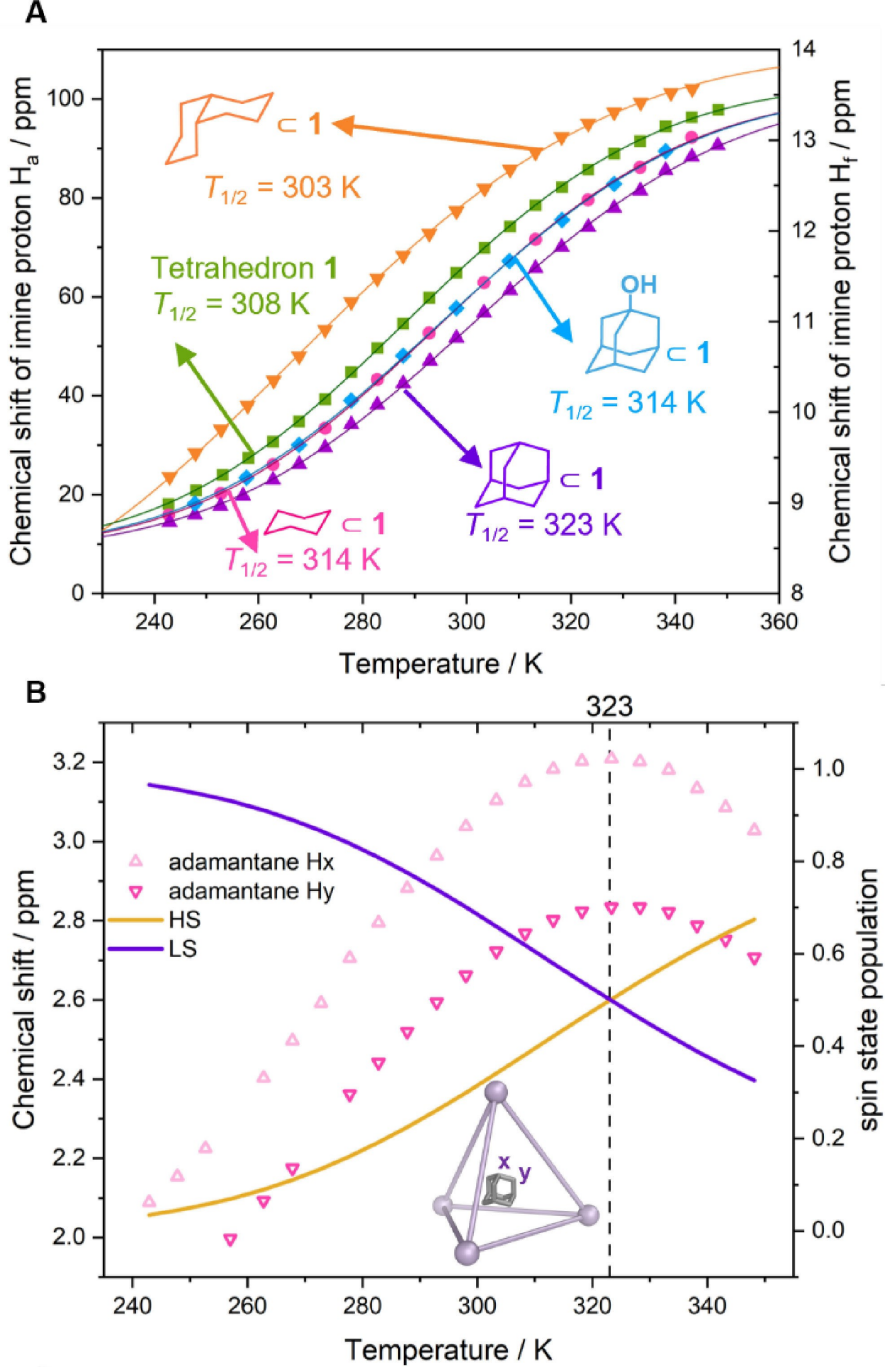
A) Measured ^1^H NMR chemical shifts of imine proton signals H_a_ and their fits to the equation in Figure 2A as a function of temperature for cage **1** (▪) and its guest adducts (adamantane⊂**1**: ▴, 1‐adamantanol⊂**1**: ⧫, cyclohexane⊂**1**: •). The imine proton signal H_a_ of *cis*‐decalin⊂**1** below 270 K were too broad to be observed; the thermodynamic parameters were obtained by analyzing phenyl proton H_f_, whose signal was observable at low temperature (*cis*‐decalin⊂**1**: ▾, Supporting Information Section 5.5). B) Chemical shifts of the encapsulated adamantane protons (H_*x*_ (▾), and H_y_ (▴)), and spin state population of adamantane⊂**1** (LS: purple curve, HS: yellow curve) over the indicated temperature range in CD_3_CN. The SCO *T*_1/2_ is given as a vertical line.

The host–guest complexes of all four guests were characterized by ^1^H NMR and ESI‐MS (Supporting Information Sections 4.1–4.4). The X‐ray crystal structure of adamantane⊂**1** was also determined (Supporting Information Section 3.2). Their SCO behaviors were studied by VT NMR (Figure 3, Table 1, Supporting Information Sections 5.2–5.5). Host‐guest exchange was in all cases slow on the NMR timescale. At 298 K, the signals corresponding to encapsulated guests exhibited an unusual downfield shift, indicating an anisotropic environment due to the mixed‐spin microstates (Figure 2C). Because of the change in ratio between microstates, we observed a complicated chemical‐shift temperature dependence (Figures 3B, S85, and S92). The number and arrangement of the paramagnetic HS centers in the individual cage microstates provide distinct magnetic environments for the encapsulated guest, which deviates from the upfield shift observed for LS‐only cages. Since the guests are non‐covalently bound, only through‐space pseudocontact contributions,[^89^, ^90^, ^91^] which depend on the local magnetic anisotropy, play a role. For cage **1**, three of these five microstates are anisotropic, with paramagnetic spin density concentrated on one side of the cage (Figure 2C, the three states in the top center). Since the tumbling guest atoms are on average equidistant from each Fe^II^ center, their nuclei experience an averaged environment of the cage spin states as a whole. Since a maximum in chemical shift was observed at around *T*_1/2_, where equal amount of HS and LS states were reached, we infer the anisotropy to be highest here.

**Table 1:** Enthalpy Δ*H* and entropy Δ*S* of SCO and the spin state populations γ_LS_ and γ_HS_ of LS and HS states at 298 K in tetrahedron **1** and its host–guest complexes. The relative affinities *K*_**G**⊂**HS**_ *K*_**G**⊂**LS**_^−1^ of the guests are for the two spin states of **1**, and the absolute affinities *K*_**G**⊂**HS**_ and *K*_**G**⊂**LS**_ of guests refer to the two individual spin states of **1**, as derived from the thermodynamic cycle (Figure 4B), as do their spin state populations γ. The observable association constants *K*_obs_ refer to the observed averaged spin state of **1** (Figure 4A). The difference between the absolute affinities *K*_**G**⊂**HS**_ and *K*_**G**⊂**LS**_ is often within the margin of error due to error propagation from the spin state population γ and the observable association constant *K*_obs_. The relative affinity *K*_**G**⊂**HS**_ *K*_**G**⊂**LS**_^−1^, however, only relies on the spin state population γ with much smaller errors and hence higher accuracy.

	Δ*H* [kJ mol^−1^]	Δ*S* [kJ K^−1^ mol^−1^]	γ_LS_	γ_HS_	*K*_**G**⊂**HS**_ *K*_**G**⊂**LS**_^−1^	*K*_obs_ [10^3^ M^−1^]	*K*_**G**⊂**LS**_ [10^3^ M^−1^]	*K*_**G**⊂**HS**_ [10^3^ M^−1^]
tetrahedron **1**	26.2±0.2	85±1	0.59±0.05	0.41±0.05				
cyclohexane⊂**1**	27.1±0.3	84±1	0.63±0.06	0.37±0.06	0.85±0.15	9.0±0.4	9.6±2.2	8.1±1.8
adamantane⊂**1**	26.4±0.3	84±1	0.70±0.05	0.30±0.05	0.62±0.10	73±16	87±33	53±20
1‐adamantanol⊂**1**	27.1±0.2	86±1	0.63±0.05	0.37±0.05	0.85±0.14	230±50	250±90	210±80
*cis*‐decalin⊂**1**	17.9±0.2	59±1	0.52±0.05	0.48±0.05	1.32±0.24	7.2±0.5	6.3±1.6	8.4±2.1

Despite the presence of these microstates, which rapidly equilibrate on the ^1^H NMR time scale, our two spin state model (discussed above), is still applicable to the bulk behavior of the system.

Fine‐grained details of the thermodynamics of host–guest binding of cyclohexane, adamantane, 1‐adamantanol, and *cis‐*decalin within **1** were revealed through comparison of the SCO behaviors of these host–guest adducts. Figure 3 shows the impacts upon SCO of guest binding: Bound cyclohexane and 1‐adamantanol both stabilized the LS state of **1** to a similar extent, with *T*_1/2_ for the adducts of both guests occurring at 314 K vs. 308 K for empty **1**. Adamantane stabilized LS **1** to an even greater extent (*T*_1/2_=323 K), whereas the larger guest *cis*‐decalin destabilized LS **1** (*T*_1/2_=303 K) in favor of the HS state. Intrigued by these guest effects, we sought to create a thermodynamic cycle to quantify guest influence on host SCO.

**Figure 4:**
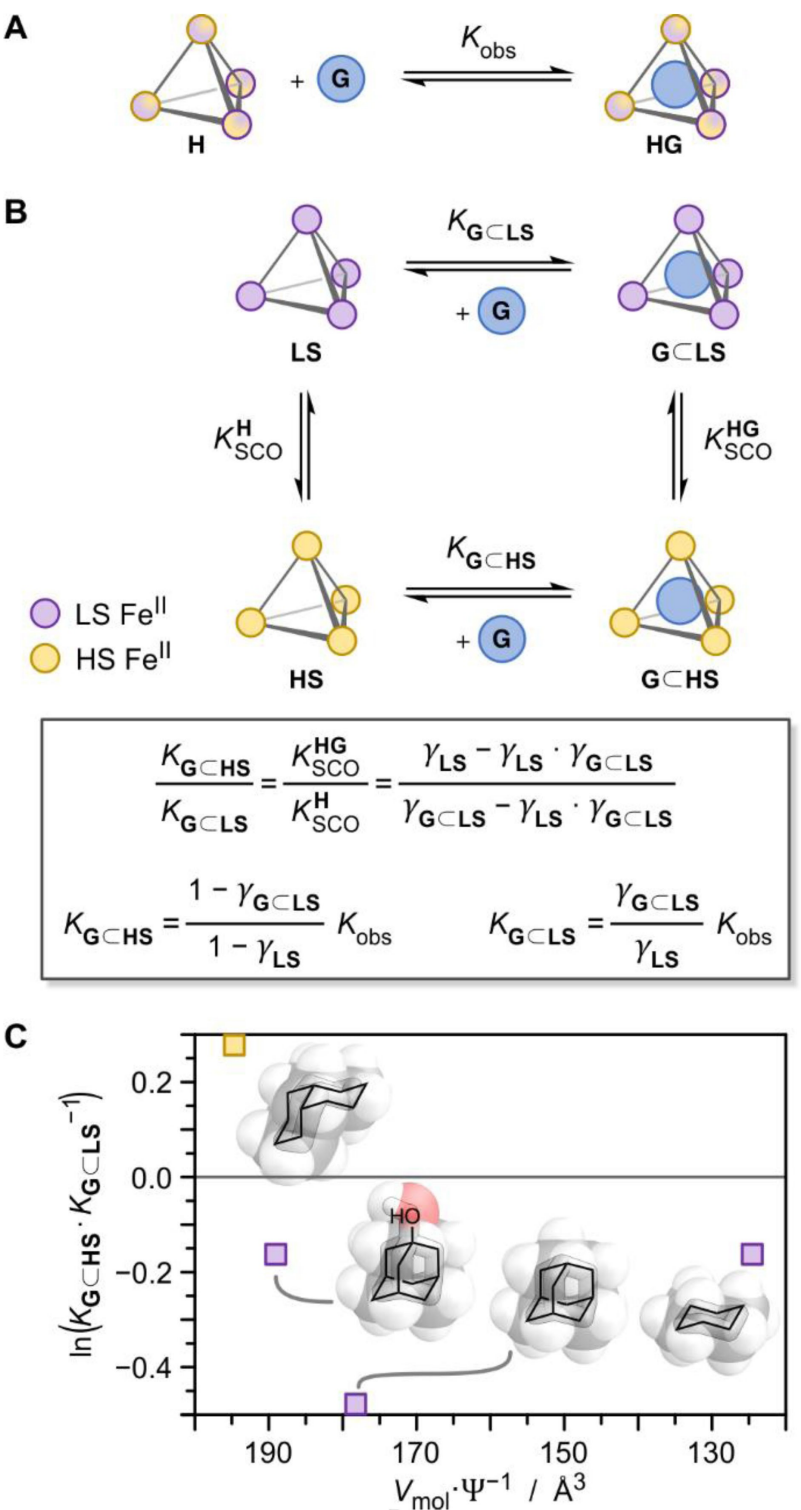
A) A 1 : 1 host–guest equilibrium can be observed by ^1^H NMR for tetrahedron **1** (**H**: host, **G**: guest, **HG**: host–guest complex, *K*_obs_: observed association constant). B) Thermodynamic cycle showing the interplay between host–guest complexation and SCO, as detailed in Supporting Information section 4.9.1. *insert*: Relative affinity *K*_**G**⊂**HS**_ *K*_**G**⊂**LS**_^−1^ of the guests for the two spin states of **1** and absolute affinities *K*_**G**⊂**HS**_ and *K*_**G**⊂**LS**_ of guests to the two individual spin states of **1**, as derived from the thermodynamic cycle, their spin state populations γ and *K*_obs_ (Table 1). C) Affinity bias of four guests for binding within **1** in the HS vs. LS state, expressed as the logarithm of the relative affinities ln(*K*_**G**⊂**HS**_ *K*_**G**⊂**LS**_^−1^), plotted against guest size, expressed as sphericity‐corrected molecular volumes *V*_mol_ Ψ^−1^. LS/HS state stabilization marked in purple and yellow, respectively. MM2 molecular models of guests are shown; from left to right: *cis*‐decalin, 1‐adamantanol, adamantane, and cyclohexane.

A model was derived (Figure 4A, Supporting Information section 4.9.1) to link the binding affinities of the guests in the two spin states of tetrahedron **1** to the thermodynamics of SCO (Table 1). Under the simplifying assumption that all Fe^II^ ions within tetrahedron **1** are either HS or LS, the host–guest equilibrium (H+G⇌HG, Figure 4A) is replaced by the thermodynamic cycle depicted in Figure 4B. The cycle consists of two known and two unknown equilibrium constants. The two known equilibrium constants are $K_{SCO}^{H}$ and $K_{SCO}^{HG}$ describing the SCO process of host **1** and its host–guest complexes, respectively. $K_{SCO}^{H}$ and $K_{SCO}^{HG}$ can be derived from their respective spin state populations γ_**LS**_ and γ_**G**⊂**LS**_ as determined by VT NMR (Figure 3 and Table 1).

**Figure 5:**
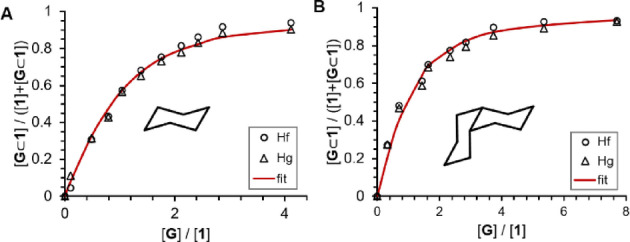
Association isotherms fitted according to a typical 1 : 1 binding model (Supporting Information, Eq. 28[^92^, ^93^, ^94^]) obtained from titration of different guests to tetrahedron **1** (for spectra see Supporting Information, Figures S65 and S66). Isotherms from phenylene protons H_f_ (○) and methyl protons H_g_ (▵) were fitted simultaneously (red line): A) cyclohexane, B) *cis*‐decalin.

With two of the association constants of the thermodynamic cycle known, the relative affinity *K*_**G**⊂**HS**_ *K*_**G**⊂**LS**_^−1^ of a guest to HS and LS **1** can be determined (equation in Figure 4B, insert, top). The logarithm of this ratio ln(*K*_**G**⊂**HS**_ *K*_**G**⊂**LS**_^−1^) is plotted against sphericity‐corrected guest volume *V*_mol_ Ψ^−1^ in Figure 4C, revealing a higher affinity for smaller guests to LS **1**, with an optimum at adamantane, whereas the larger guest *cis*‐decalin favored the HS state. This model furthermore allowed quantification of the affinities of the guests to the HS and LS states of tetrahedron **1** via NMR titrations (Table 1 and Supporting Information Section 4.9.2). Since only an averaged ^1^H NMR signal is observed for tetrahedron **1**, NMR titration data of the different guests binding to tetrahedron **1** were fitted to a simple 1 : 1 isotherm to determine *K*_obs_ (Figures 4A and 5; Figure 5 shows the association isotherms for cyclohexane and *cis*‐decalin, the rest is in Supporting Information, section 4.9.2).[^92^, ^93^, ^94^] With the equations depicted in the insert of Figure 4B, the association constants of the guests to the two spin states *K*_**G**⊂**HS**_ and *K*_**G**⊂**LS**_ can be calculated from *K*_obs_ and the spin state populations γ.

We infer that subtle structural differences between cages in the two spin states may account for the different affinities of guests in these states. In the HS state, cages tend to have larger cavities due to the longer Fe^II^−N bonds. For cage **1**, the cavity volume of the LS state (227 Å^3^) was calculated from its fully LS crystal structure at 180 K (Figure S31). Obtaining the structure of **1** in the HS state, however, would require temperatures too high for routine structure determination. The cavity of cage **2**, constructed from subcomponent **B**, provides a good approximation for the cavity of tetrahedron **1** in its HS state (Figure S33).

Cage **2** is similar structurally to cage **1**, but **2** adopts the HS state at 100 K, providing a good model for the HS state of cage **1**. The cavity volume of **2** was calculated to be 241 Å^3^, about 5 % larger than LS **1** (Table S2). Furthermore, HS cages have more loosely bound metal centers which may render them better able to adopt the expanded conformations required to accommodate larger guests. Thus, larger guests may prevent the HS framework of SCO tetrahedron **1** from shrinking as its Fe^II^ centers undergo transition to the LS state.^[63]^

The X‐ray crystal structure of adamantane⊂**1** was obtained in the LS state (Supporting Information, Section 3.5). The encapsulation of adamantane resulted in a similar cavity size as empty **1**, contracting by only 4 Å^3^ (Table S2). Previous studies have shown that the flexibility of subcomponent **D** allows for expansion or contraction to optimize contact with different guest species.^[86]^ This observation suggested that the LS state of cage **1** provided a more suitable host for smaller guests such as adamantane. We infer that in order to maintain the best binding configuration for smaller guests, the flexibility of the cage is limited, rendering the HS state energetically less accessible and resulting in higher SCO temperatures for smaller guests.

Among the guests that were observed to stabilize LS **1**, adamantane occupies around 78 % of the cavity volume of the LS cage (by sphericity‐corrected volume, *V*_mol_ Ψ^−1^, Table S3). We infer this value to be close to the optimum occupancy because of the above‐mentioned slight cavity contraction of **1** that occurs upon adamantane encapsulation. This near‐optimal fit of adamantane to LS tetrahedron **1** resulted in adamantane showing the most efficient stabilization of the LS state. Note that this occupancy is not at the optimum of Rebek's 55 % rule,^[34]^ because the van der Waals volumes used in that rule do not allow an adequate comparison of our guests as outlined above. Hence, the 78 % occupancy of adamantane is used as a benchmark herein.

Cyclohexane also favors binding within the smaller cavity of LS **1**; however, it is too small to fill the cavity optimally—it only takes up 55 % of the cavity of LS **1** (by *V*_mol_ Ψ^−1^, Table S3). We infer that it is not energetically favorable for LS **1** to shrink enough to generate an optimal match for cyclohexane. Since this match is not as good for cyclohexane as it is for adamantane, the driving force for cyclohexane⊂**1** to remain in its LS state is lower and so is its spin transition temperature *T*_1/2_. 1‐Adamantanol is slightly larger than adamantane (83 % occupancy by *V*_mol_ Ψ^−1^, Table S3), rendering its binding within **1** slightly less favorable in the LS state than for adamantane (Figure 4C).

Notably, smaller cyclohexane and larger 1‐adamantanol stabilize LS tetrahedron **1** to the same extent, even though their volumes deviate from that of adamantane by different amounts, ca. −50 Å^3^ and +10 Å^3^ (*V*_mol_ Ψ^−1^), respectively (Figure 4C). With a *V*_mol_ Ψ^−1^ of 195 Å^3^, the largest guest *cis*‐decalin occupies 86 % of the cavity of LS tetrahedron **1** (Table S3), well above the adamantane optimum of 78 %. For HS **1**, this cavity occupation reduces to 81 %, closer to the optimal 78 %. This optimal HS occupancy is reflected in nearly as strong a stabilization of the HS state of **1** as adamantane stabilizes the LS state.

The observed trend for LS/HS stabilization (Figure 4C) follows the general shape of a particle–particle interaction potential, such as the Lennard‐Jones potential.^[95]^ An optimum guest size for stabilizing LS **1** is observed at adamantane. As guest size decreases, a gradual decrease in the stabilization of LS **1** is predicted, analogous to how two particles experience less attraction as the distance between them increases. Increasing in size from the optimum, however, causes a sharp LS destabilization (1‐adamantanol), then a crossover to HS stabilization (*cis*‐decalin), and ultimately a failure of larger guests to bind at all. This behavior mirrors the repulsion experienced by two particles that approach each other too closely.

The SCO behavior of tetrahedron **1** thus served as a sensitive probe to determine optimal fit between cage cavity and guest size. Association constants alone do not provide information about the size match of a guest, because they depend on other non‐covalent interactions. For example, 1‐adamantanol binds three times more strongly to **1** than does adamantane (2.3×10^5^ m^−1^ and 7.3×10^4^ m^−1^, respectively, Table 1), potentially due to additional cation‐dipole interactions between the cavity and guest.^[35]^ However, adamantane provides a better fit for the cavity. Hence, SCO provided a unique perspective into the degree of host–guest size matching between neutral guests in a SCO host. Our work thus consolidates understanding gained previously[^56^, ^60^] and provides a framework for future fine‐grained studies of host–guest binding. The versatile approach of subcomponent self‐assembly could enable other cages that incorporate SCO‐favoring aldehyde **A** to similarly report on the size match of host to guest, providing complementary information to that provided by binding constant measurements.

## Conclusion

The use of SCO to determine guest fit within a host cavity thus provides information complementary to binding affinity (*K*_a_). We infer that aldehyde subcomponent **A** could be used in place of 2‐formylpyridine in many cage frameworks,^[73]^ enabling optimal guest fit to be quantified using the method described here. Such studies could enable binding affinity to be separated into “fit” and “specific‐interactions” contributions, potentially enabling the fine‐grained design of host–guest pairs for specific applications.^[96]^

## Conflict of interest

The authors declare no conflict of interest.

1

## Supporting information

As a service to our authors and readers, this journal provides supporting information supplied by the authors. Such materials are peer reviewed and may be re‐organized for online delivery, but are not copy‐edited or typeset. Technical support issues arising from supporting information (other than missing files) should be addressed to the authors.

Supporting Information

Supporting Information

Supporting Information

Supporting Information

Supporting Information

Supporting Information

## Data Availability

The data that support the findings of this study are available in the supplementary material of this article.
