# Supplementary material for: Host Spin‐Crossover Thermodynamics Indicate Guest Fit
Source: Angew Chem Int Ed Engl. 2022 Nov 17;61(50):e202212634. doi: 10.1002/anie.202212634 (PMC10098494; doi:10.1002/anie.202212634)
Supplement: Supplementary file 1 — Supporting Information [file ANIE-61-0-s001.pdf]

Angewandte Chemie
Eine Zeitschrift der Gesellschaft Deutscher Chemiker

Supporting Information

Host Spin-Crossover Thermodynamics Indicate Guest Fit

J. Zheng, L. K. S. von Krbek, T. K. Ronson, J. R. Nitschke**

Contents

1 Materials and methods 2
2 Supplemental experimental procedures 3
2.1 Tetrahedron **1** 3
2.2 Tetrahedron **2** 10
2.3 Tetrahedron **3** 13
2.4 Cube **4** and trigonal prism **5** 17
3 X-ray crystallography and volume calculations 23
3.1 Tetrahedron **1** 23
3.2 Adamantane⊂**1** 24
3.3 Adamantane⊂**2** 25
3.4 Tetrahedron **3** 26
3.5 Trigonal prism **5** 27
3.6 Volume calculations 28
4 Host-Guest Chemistry 33
4.1 Adamantane⊂**1** 33
4.2 1-Adamantanol⊂**1** 36
4.3 Cyclohexane⊂**1** 39
4.4 *cis*-Decalin⊂**1** 42
4.5 Adamantane⊂**2** 45
4.6 Adamantane⊂**3** 47
4.7 Comparison of guest chemical shifts in host-guest complexes of tetrahedron **1** 49
4.8 Zn(II) analogue of tetrahedron **1** and its host-guest compounds 50
4.9 Guest binding affinity depending on the spin states of tetrahedron **1** 51
4.9.1 Deriving individual association constants for both spin states of tetrahedron **1** 51
4.9.2 NMR titrations 56
5 Variable-Temperature ^{1}H NMR and Spin-Crossover Studies 60
5.1 Tetrahedron **1** 61
5.2 Adamantane⊂**1** 63
5.3 1-Adamantanol⊂**1** 66
5.4 Cyclohexane⊂**1** 70
5.5 *cis*-Decalin⊂**1** 74
5.6 Tetrahedron **2** 77
5.7 Adamantane⊂**2** 78
5.8 Tetrahedron **3** 79
5.9 Adamantane⊂**3** 82
5.10 Cube **4** and trigonal prism **5** 85
6 Supplemental References 87

1 Materials and methods

Unless stated otherwise, all reagents used in this project were purchased commercially and used without further purification. Iron(II) bis(trifluoromethylsulfonyl)imide (iron triflimide, $Fe(NTf_2)_2$) and N2,N4,N6-tris(4-aminophenyl)-N2,N4,N6-trimethyl-1,3,5-triazine-2,4,6-triamine (**D**) were prepared following previously reported literature procedures.[1,2]

NMR spectra were recorded on a Bruker 400 MHz Avance III HD Smart Probe (routine and paramagnetic ^{1}H NMR experiments, ^{19}F NMR experiments, COSY experiments, and DOSY experiments), a Bruker Avance III HD 500 MHz Smart Probe (routine and paramagnetic, and variable-temperature ^{1}H NMR experiments), and a Bruker Avance 500 MHz TCI-ATM Cryoprobe (routine ^{1}H NMR experiments). Chemical shifts (δ) are reported in ppm, and ^{1}H NMR spectra are referenced to the residual solvent peaks (CD_3CN: 1.94 ppm, CD_3NO_2: 4.33 ppm). All experiments were carried out at 298 K unless stated otherwise. Spectra were processed using Topspin 3.5pl2 and MestReNova versions 11.0 and 14.2. Chemical shifts versus temperatures were analyzed with Equation S29 using Origin 2020.

Paramagnetic ^{1}H NMR experiments were performed using the zg30 program with a sweep width (sw) of 356.96 ppm and a transmitter frequency offset (o1p) of 125 ppm. Variable-temperature ^{1}H NMR experiments were carried out according to the following procedure: the temperature was decreased from 298 K to 243 K during which spectra were collected in 5 K or 10 K intervals. Then, the temperature was increased to 298 K to record spectra at temperatures higher than 298 K in 5 K or 10 K intervals. COSY experiments were undertaken using the cosygpppqf program and DOSY experiments were carried out using the ledbpgp2s program.

T_1 measurements were conducted using the t1ir pulse program to assign the peaks in the ^{1}H NMR spectrum of tetrahedron **1**. Experiments were undertaken within a sweep width of 10 ppm centered at the signal peak or group of signals. Data was processed with Dynamic Centre 4.6.2 and fitted with equation S1:

$$I(t) = I_O(1 - a\, e^{-\frac{t}{T_1}}) \qquad \text{(S1.)}$$

Low-resolution electrospray ionization mass spectrometry (LR ESI-MS) was undertaken on a Micromass Quattro LC mass spectrometer (cone voltage 5-20 eV; desolvation temperature 313 K; ionization temperature 313 K) infused from a Harvard syringe pump at a rate of 10 μL·min^{-1}. High-resolution electrospray ionization mass spectrometry (HR ESI-MS) was undertaken on a Waters' Synapt G2-Si Quadrupole-Ion mobility-TOF hybrid instrument.

2 Supplemental experimental procedures

2.1 Tetrahedron **1**

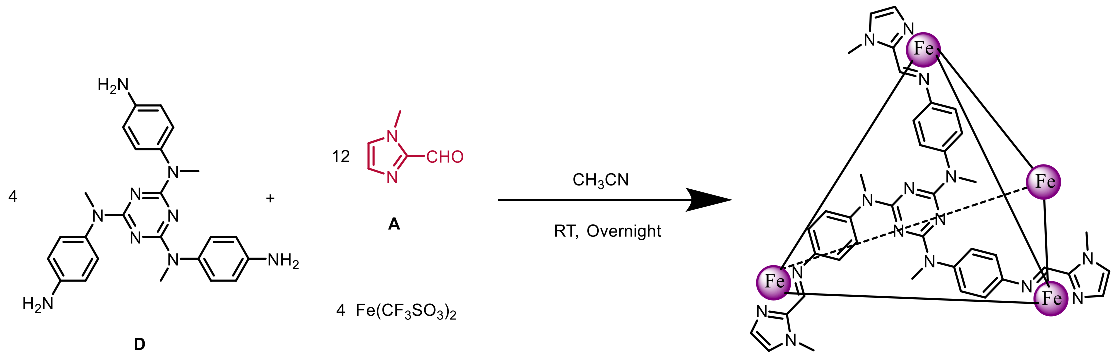

Scheme S1 Subcomponent self-assembly of tetrahedron **1**.

Triamine **D** (10.98 mg, 24.9 µmol, 4 equiv.), iron(II) triflate (8.81 mg, 24.9 µmol, 4 equiv.), and aldehyde **A** (8.25 mg, 74.6 µmol, 12 equiv.) were dissolved in MeCN (3 mL). The reaction mixture was degassed by three freeze-pump-thaw cycles then stirred at room temperature overnight. The product was precipitated with diethyl ether and separated by centrifugation. Then the solid was suspended in diethyl ether and washed with diethyl ether three times (3×15 mL). The resulting reddish purple amorphous solid contained unidentified impurities (6-10 ppm in the ^{1}H NMR spectrum, Figure S1, hypothesized to be intermediates or fragments) which were successfully removed by crystallization. For crystallization, the amorphous solid was dissolved in MeCN (3 mL), and slow vapor diffusion of diethyl ether into the solution afforded dark purple crystals of tetrahedron **1** (11.2 mg, 42%). Characterization and host-guest studies of tetrahedron **1** were carried out using crystalline samples after drying them under a stream of N_2.

^{1}H NMR (400 MHz, 298 K, CD_3CN): δ 64.72 (H_a), 36.06 (H_d), 35.53 (H_c), 11.81 (H_f), 4.49 (H_g), 4.03 (H_b), 0.98 (H_e) ppm.

LRMS (ESI$^+$-Q, CH_3CN) [charge, calculated for $Fe_4(C_{39}H_{39}N_{15})_4(CF_3SO_3)_8$]: *m/z* = 368.8 [$\mathbf{1}^{8+}$ 368.8], 463.4 [$\mathbf{1}(OTf)_1^{7+}$ 463.4], 565.6 [$\mathbf{1}(OTf)_2^{6+}$ 565.4], 707.8 [$\mathbf{1}(OTf)_3^{5+}$ 707.8], 922.7 [$\mathbf{1}(OTf)_4^{4+}$ 922.7], 1280.0 [$\mathbf{1}(OTf)_5^{3+}$ 1280.0].

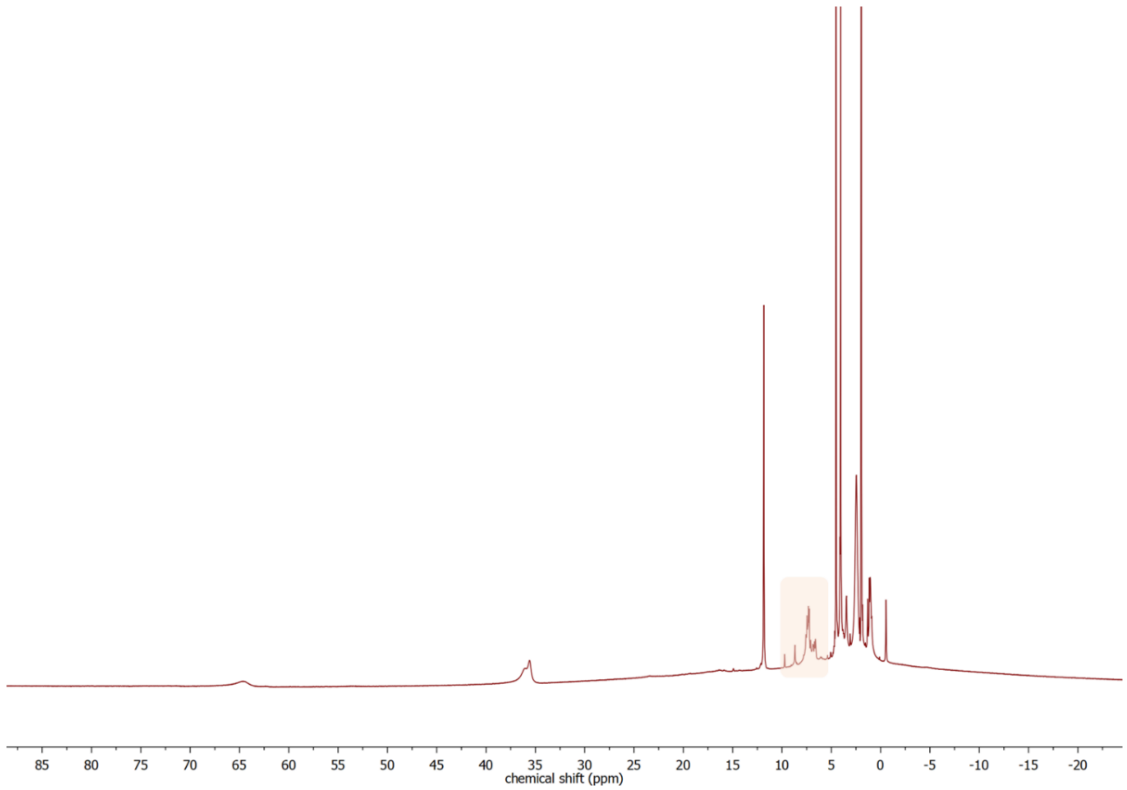

Figure S1 ^{1}H NMR spectrum of the amorphous sample of tetrahedron **1** obtained by precipitation from Et_2O (before crystallization, CD_3CN, 400 MHz, 298 K). The above-mentioned impurities are highlighted in orange.

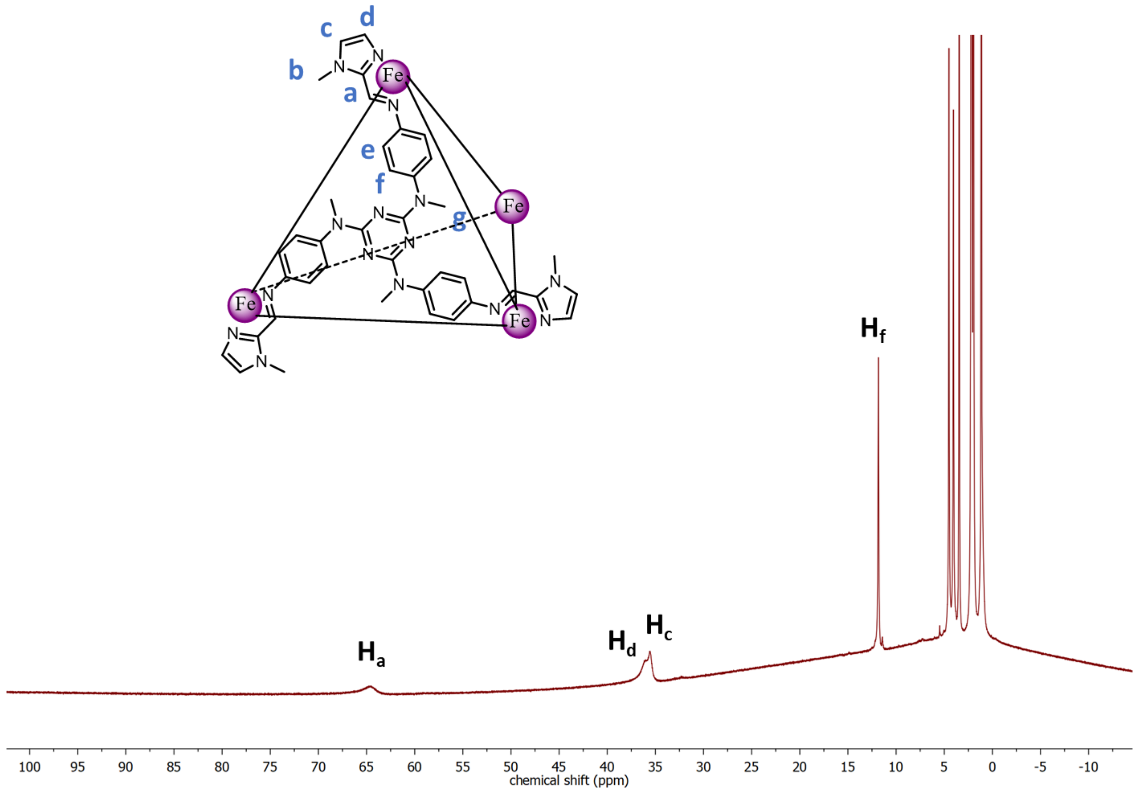

Figure S2 ^{1}H NMR spectrum of a crystalline sample of tetrahedron **1** (CD_3CN, 400 MHz, 298 K).

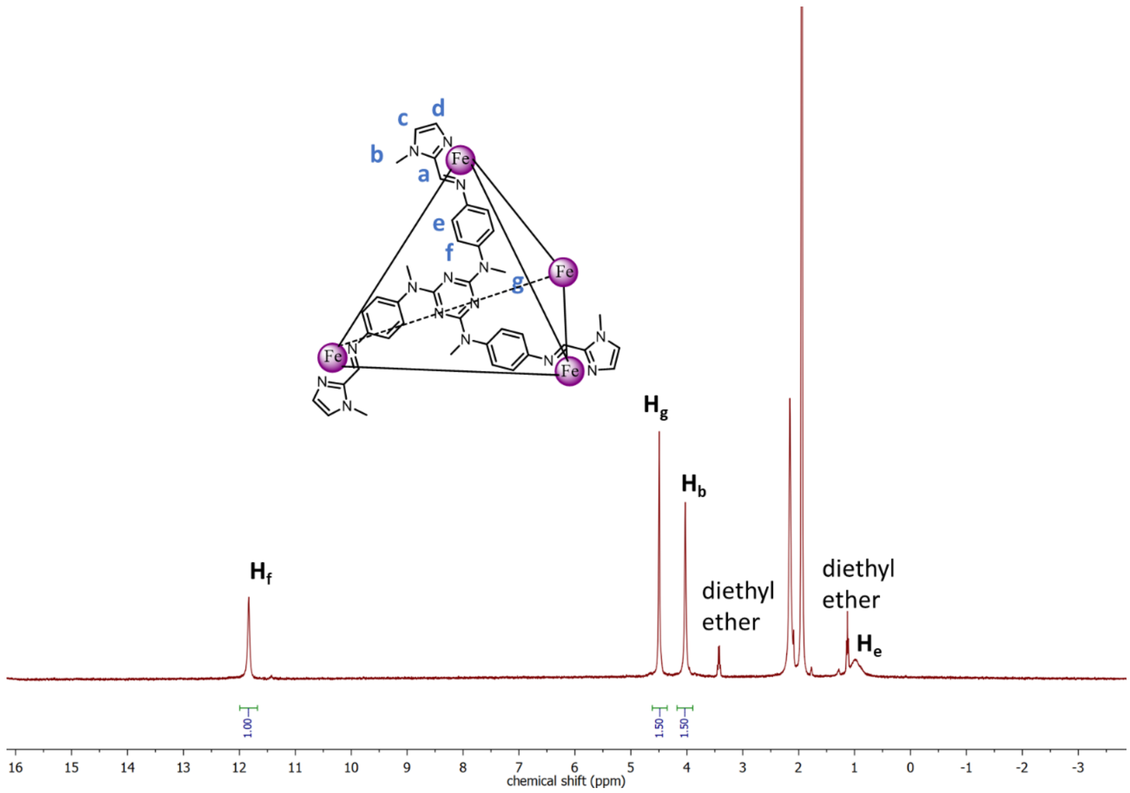

Figure S3 ^{1}H NMR spectrum of tetrahedron **1** in the diamagnetic region (CD_3CN, 400 MHz, 298 K).

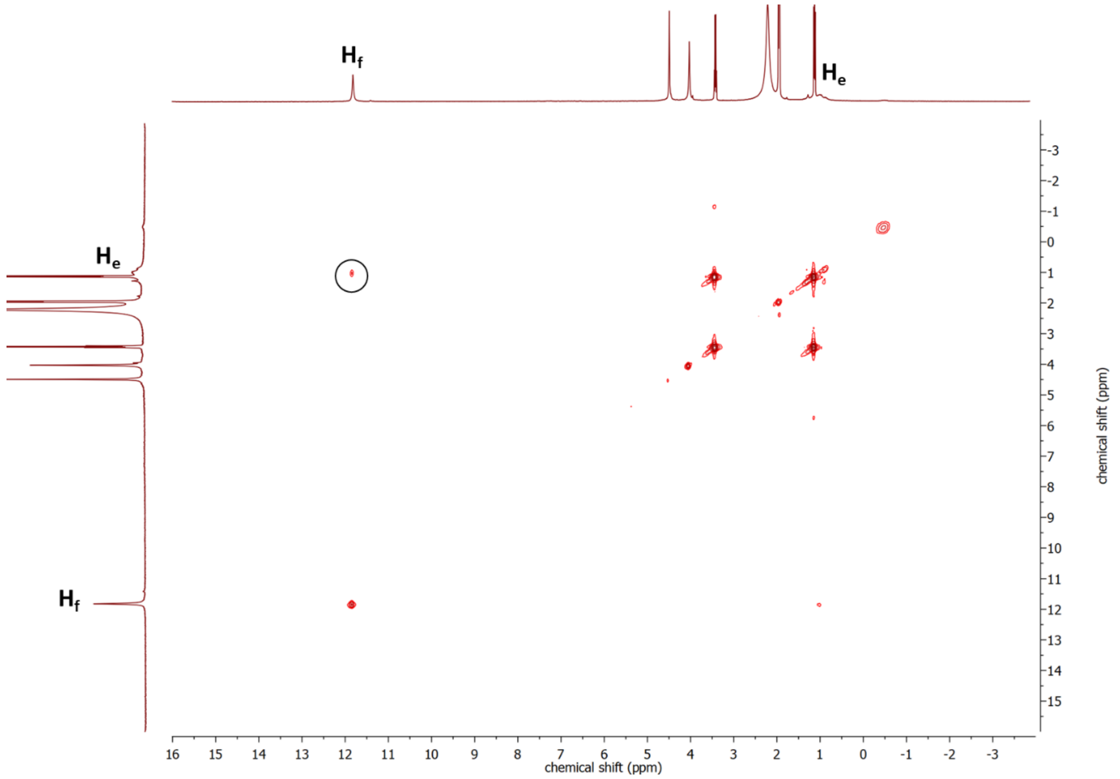

Figure S4 ^{1}H-^{1}H COSY spectrum of tetrahedron **1** in the diamagnetic region (CD_3CN, 400 MHz, 298 K). The black circle marks the cross peak between H_f and H_e.

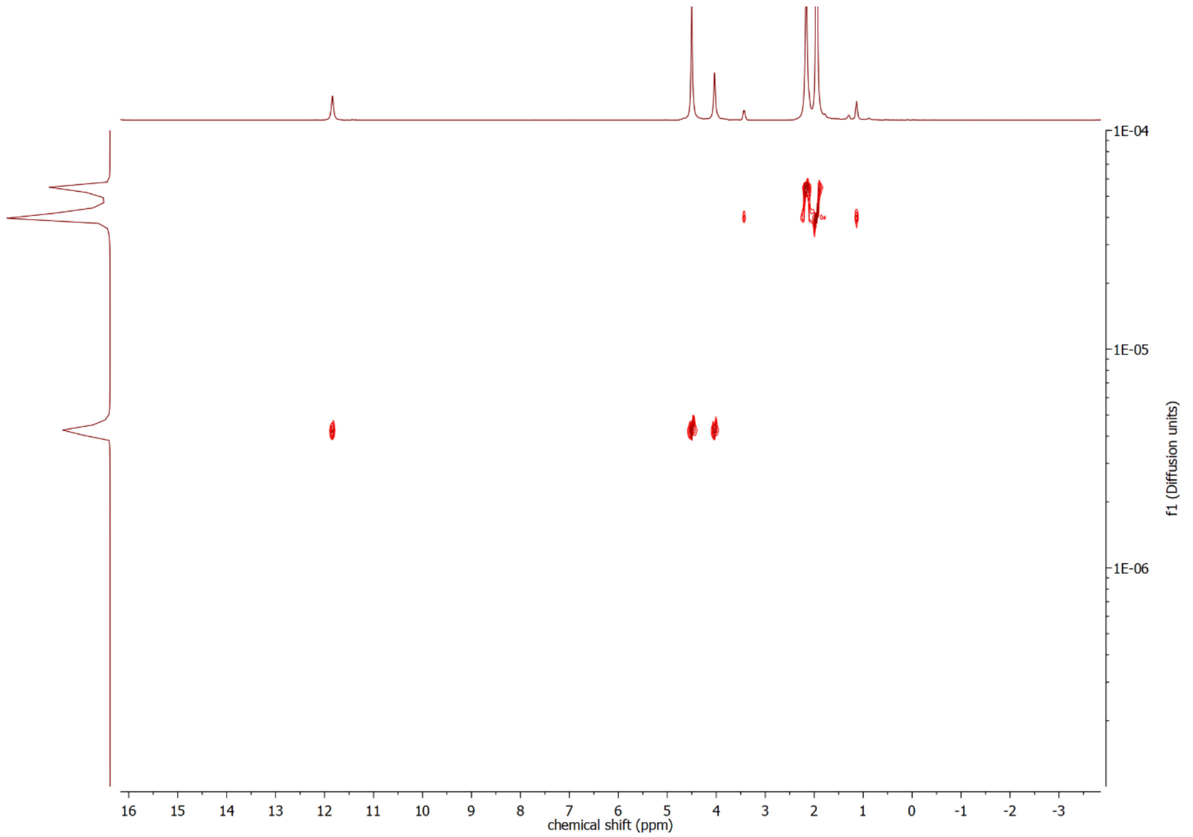

Figure S5 ^{1}H DOSY spectrum of tetrahedron **1** in the diamagnetic region (CD_3CN, 400 MHz, 298 K).

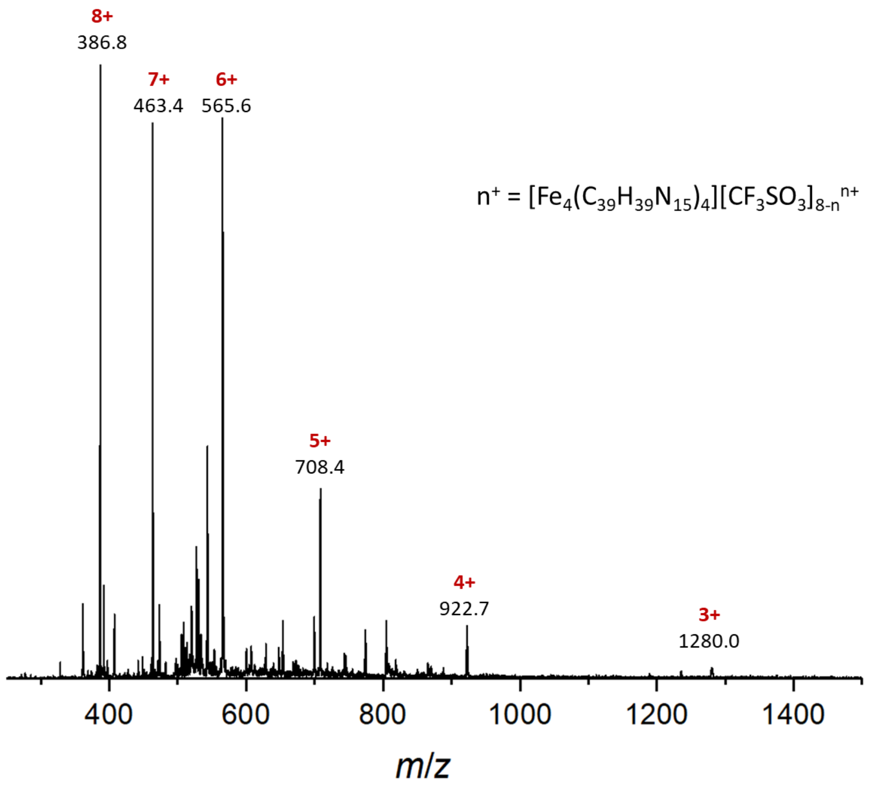

Figure S6 LRMS (ESI^{+}-Q, CH_3CN) of tetrahedron **1**.

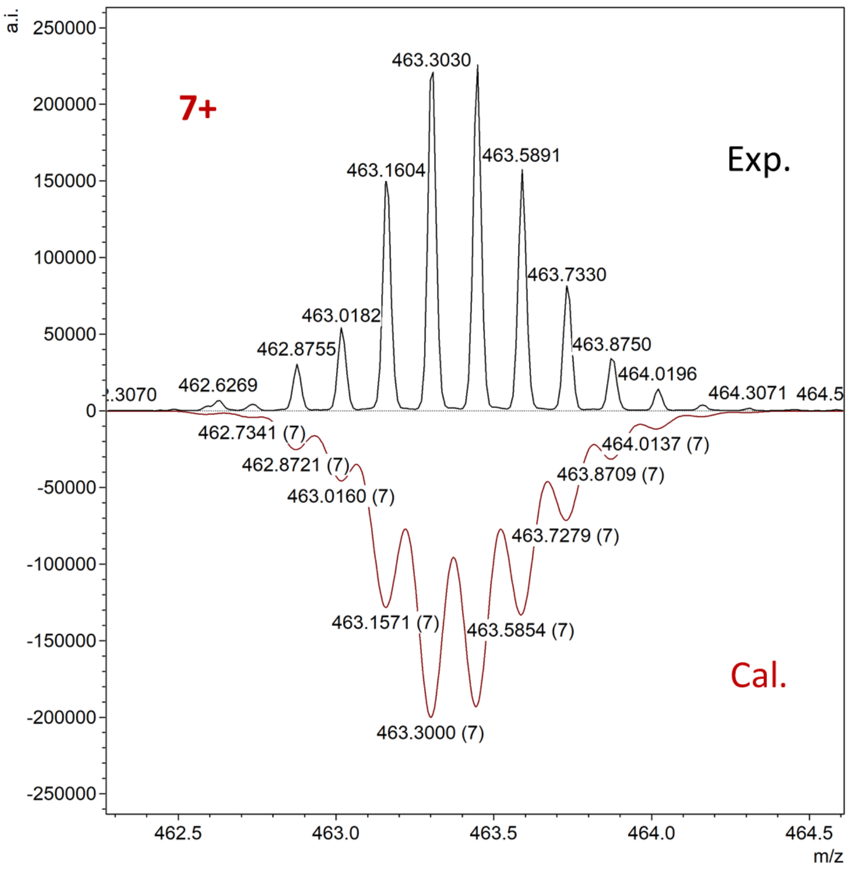

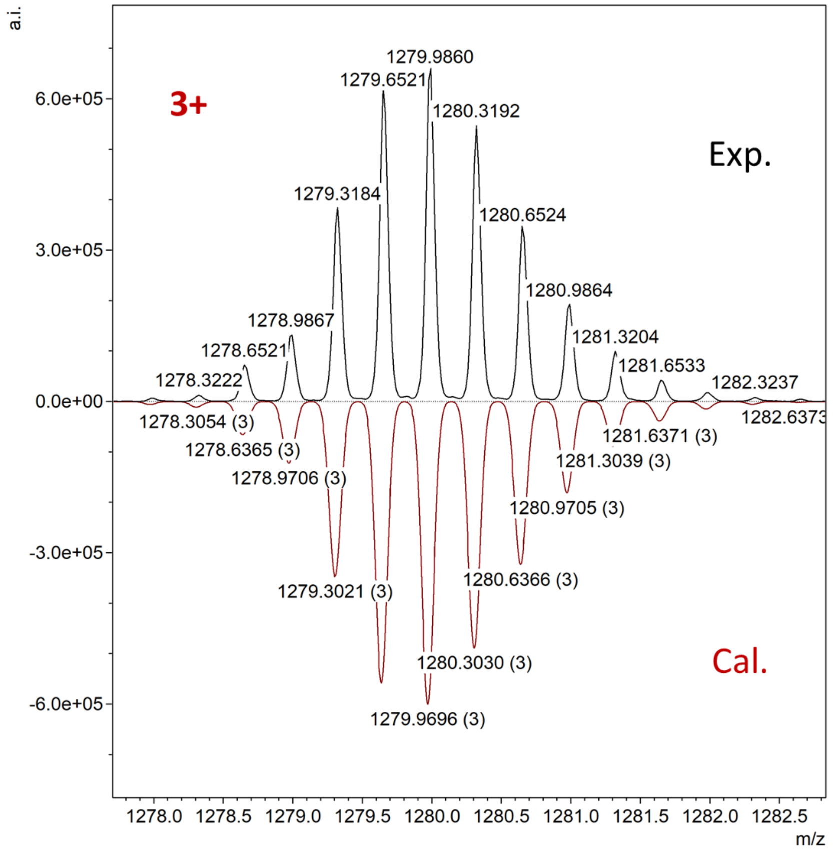

Figure S7 HRMS (ESI$^+$-QTOF, CH_3CN) of tetrahedron **1**.

Solomon's equation[3] suggests that T_1 is proportional to $[\Sigma(r_{ij})^{-6}]^{-1}$ (r_{ij} stands for the distance between the paramagnetic center and the proton), which can be used to assign proton signals in paramagnetic complexes following the methods previously applied for HS Fe(II)[4] and Co(II).[5,6] The distances were obtained from the crystal structure of tetrahedron **1** (Section 3.1). The broadness of the peak at 0.98 ppm prevented the measurement of T_1 due to very fast relaxation. This discrepancy could be attributed from the increased flexibility of the cage in solution and the existence of LS Fe(II) spin centers. However, the integrals in the ^{1}H NMR spectrum in the diamagnetic region and the cross peaks observed in the ^{1}H-^{1}H COSY spectrum of tetrahedron **1** are consistent with the assignments via *T*1 measurements.

Table S1 T_1 measurements and peak assignments for tetrahedron **1**.

**δ** (ppm)	**measured T_1** (s)	**normalized $[\Sigma(r_{ij})^{-6}]^{-1}$**	**normalized $[\Sigma(r_{ij})^{-6}]^{-1}/T_1$**	**proton assignment**
64.72	0.0102	0.01193	1.2	a
36.06	0.00746	0.00746	1	d
35.53	0.0399	0.0757	1.9	c
11.81	0.0904	0.1501	1.7	f
4.49	0.190	0.4685	2.5	g
4.03	0.0867	0.1416	1.6	b
0.98	—	0.01384	—	e

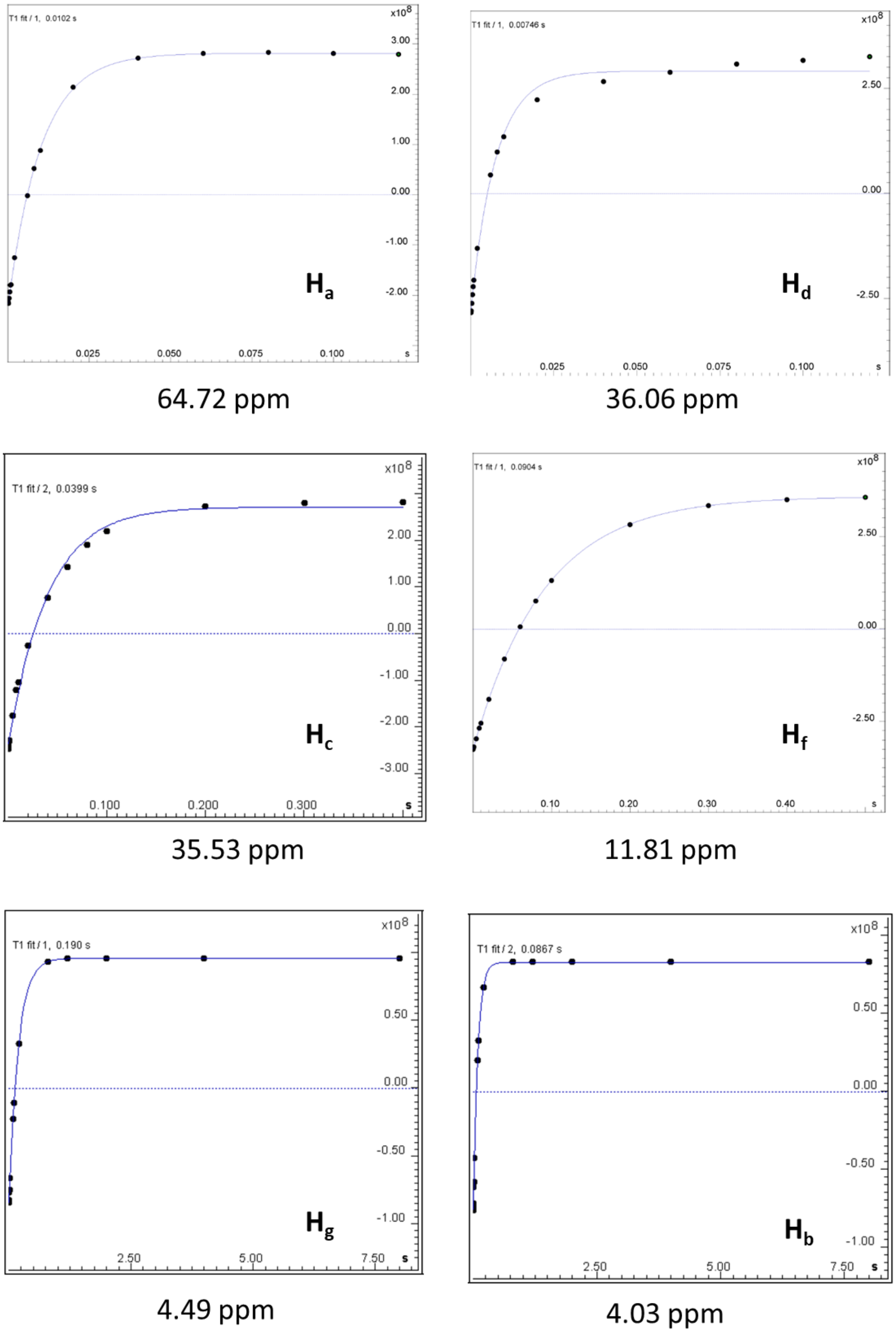

Figure S8 T_1 measurements for each signal in the paramagnetic ^{1}H NMR spectrum of tetrahedron **1** (CD_3CN, 400 MHz, 298 K). Fitted with equation S1, x axis corresponds to t (s) and y axis corresponds to intensity.

2.2 Tetrahedron **2**

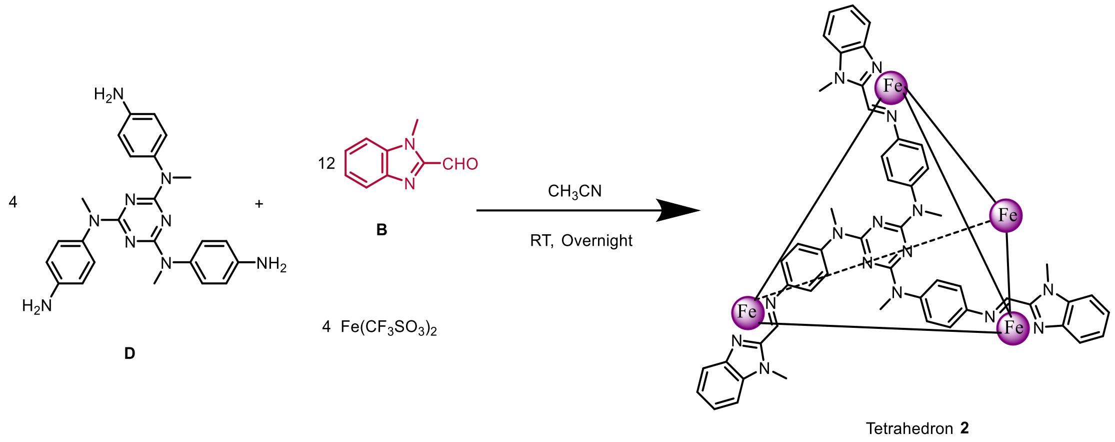

Scheme S2 Subcomponent self-assembly of tetrahedron **2**.

The synthetic procedure for tetrahedron **2** is similar to that of tetrahedron **1**. Triamine **D** (8.00 mg, 18.0 µmol, 4 equiv.), iron(II) triflate (6.41 mg, 18.0 µmol, 4 equiv.), and aldehyde **B** (8.71 mg, 54.0 µmol, 12 equiv.) were suspended in MeCN (4 mL). The reaction mixture was degassed by three freeze-pump-thaw cycles then stirred overnight at room temperature. After washing with diethyl ether three times, the amorphous product was dried under a stream of N_2 and used for characterization directly (yield: 18.7 mg, 85%). Attempts to obtain single crystals of empty tetrahedron **2** were not successful but **2** could be crystallized as its host-guest complex with adamantane (see Sections 3.3 and 4.5 below).

^{1}H NMR (500 MHz, 298 K, CD_3CN): δ 142.65, 31.98, 13.52, 12.15, 6.19, 4.74, 2.91, −7.80 ppm.

LRMS (ESI$^+$-Q, CH_3CN) [charge, calculated for $Fe_4(C_{51}H_{45}N_{15})_4(CF_3SO_3)_8$]: *m/z* = 461.8 [**2**$^{8+}$ 461.9], 549.0 [**2**(OTf)$^{7+}$ 549.2], 665.4 [**2**(OTf)$_2^{6+}$ 665.6], 828.3 [**2**(OTf)$_3^{5+}$ 828.5],1072.5 [**2**(OTf)$_4^{4+}$ 1072.9].

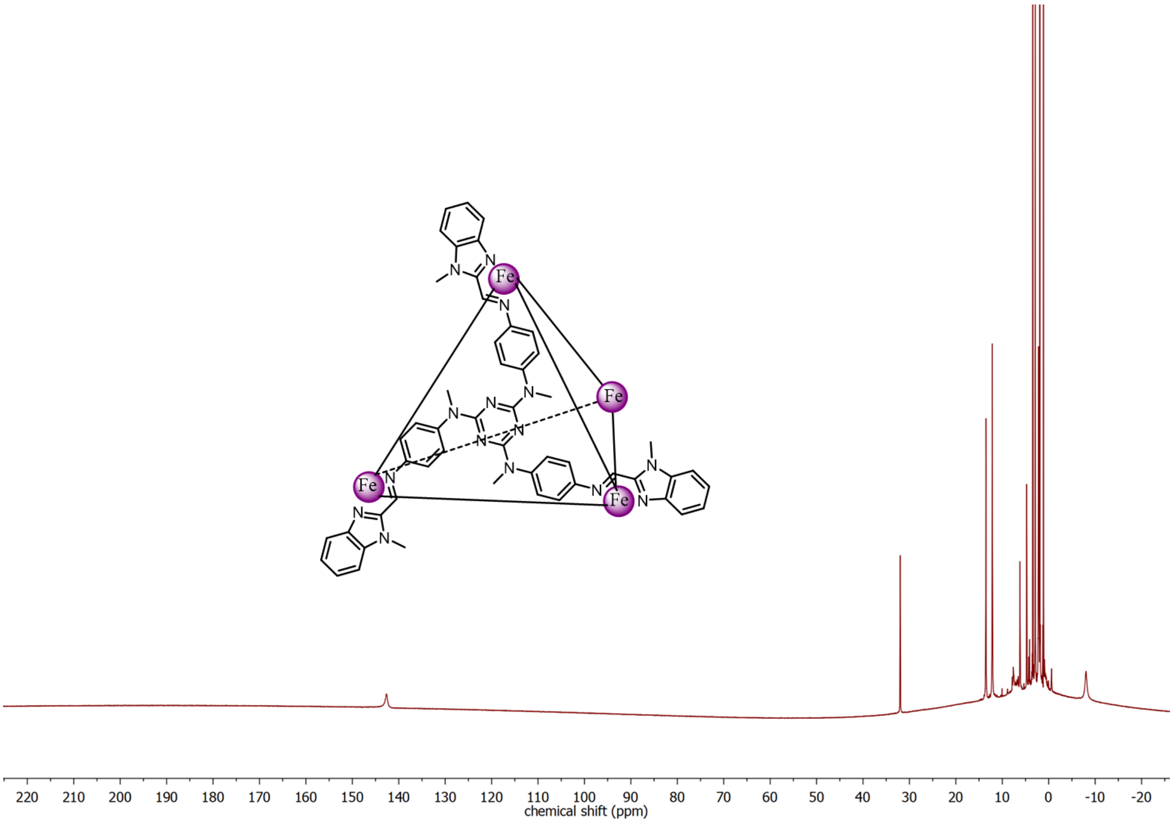

Figure S9 ^{1}H NMR spectrum of tetrahedron **2** (CD_3CN, 500 MHz, 298 K).

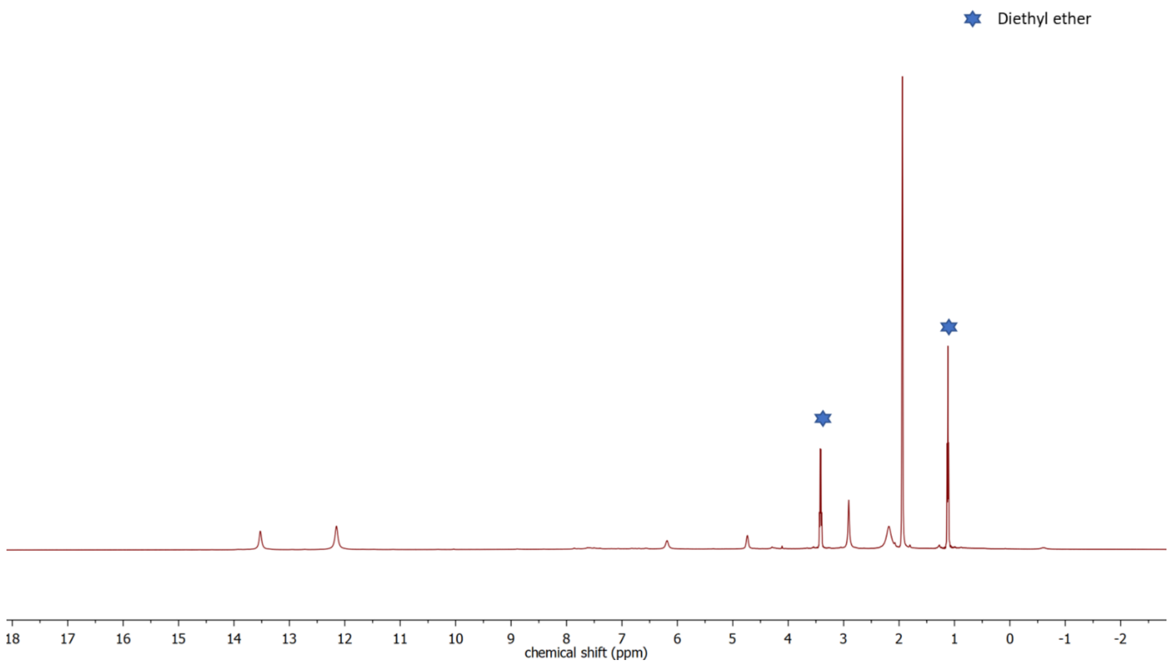

Figure S10 ^{1}H NMR spectrum of tetrahedron **2** in the diamagnetic region (CD_3CN, 500 MHz, 298 K).

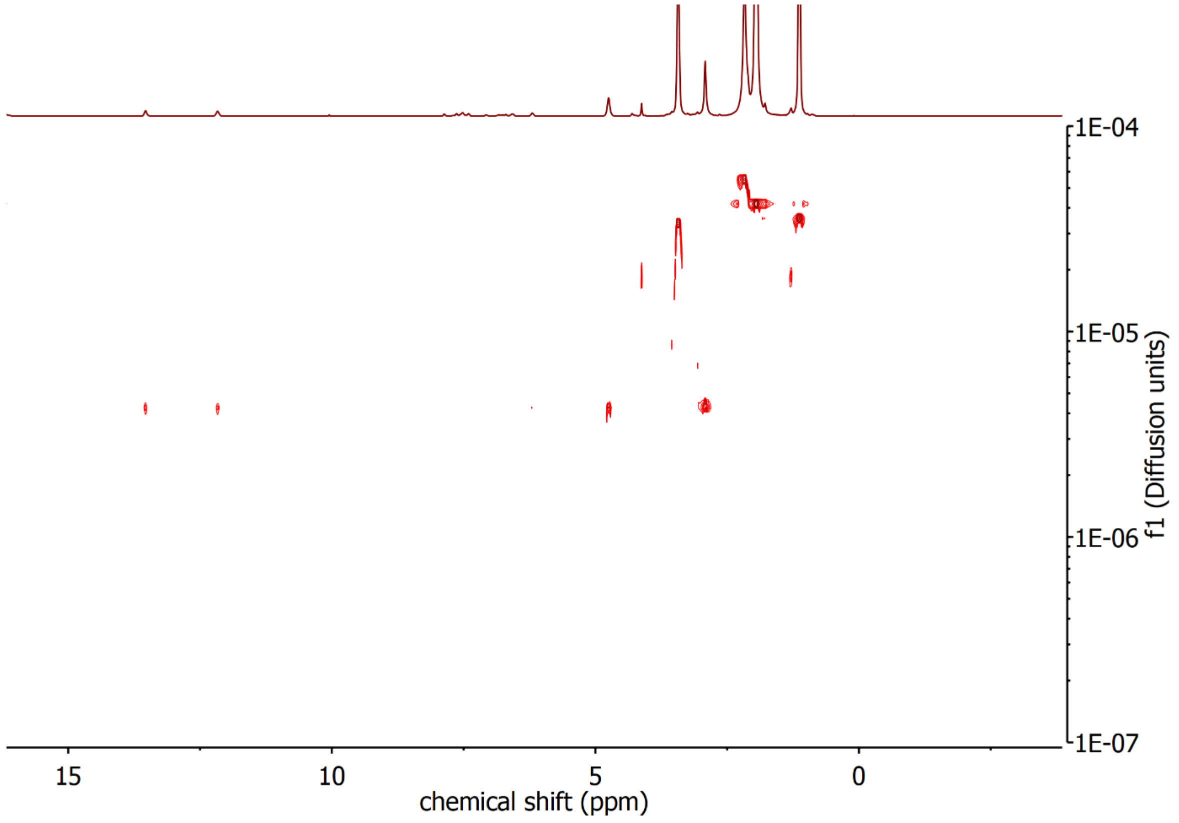

Figure S11 ^{1}H DOSY spectrum of tetrahedron **2** in the diamagnetic region (CD_3CN, 400 MHz, 298 K).

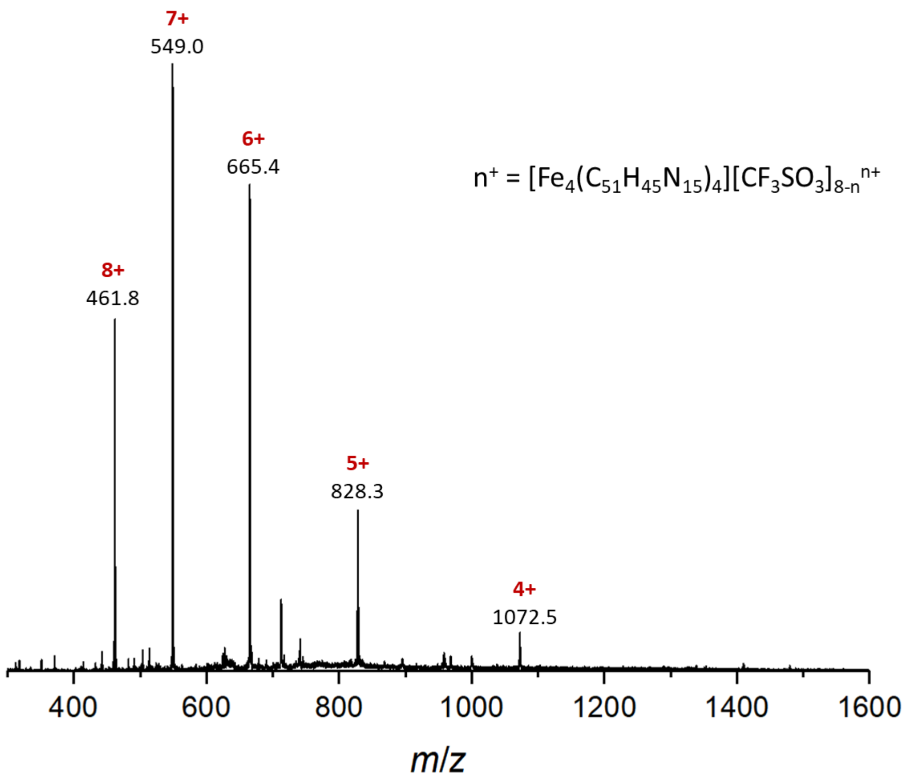

Figure S12 LRMS (ESI$^+$-Q, CH_3CN) of tetrahedron **2**.

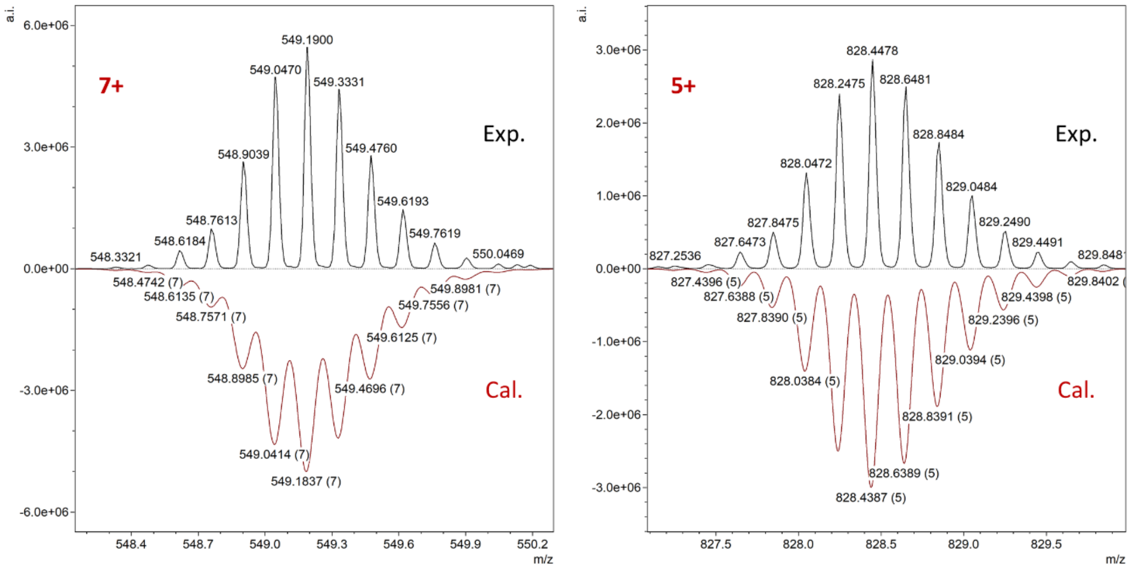

Figure S13 HRMS (ESI$^+$-QTOF, CH_3CN) of tetrahedron **2**.

2.3 Tetrahedron **3**

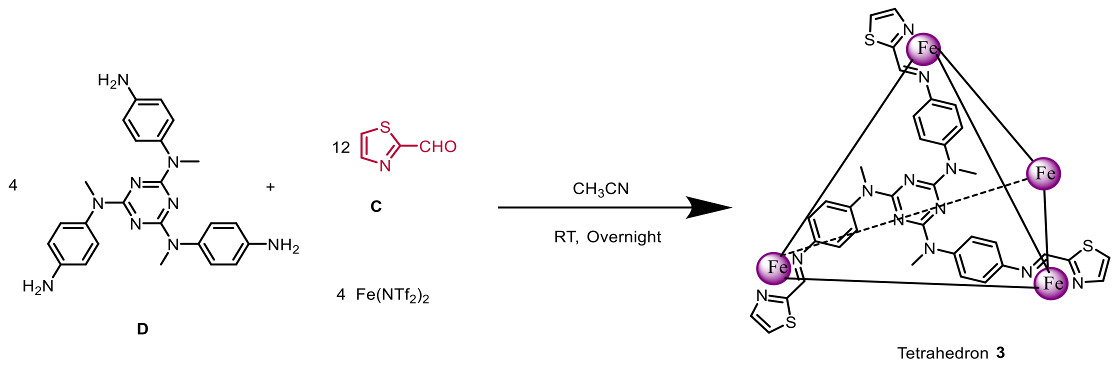

Scheme S3 Subcomponent self-assembly of tetrahedron **3**.

In the synthesis of tetrahedra **1** and **2**, $Fe(OTf)_2$ was used instead of $Fe(NTf_2)_2$ because encapsulation of NTf_2^- was observed in an analogous LS cage.[8] However, tetrahedron **3** could not form in the presence of $Fe(OTf)_2$, possibly due to the lack of a template; thus, $Fe(NTf_2)_2$ was used in the self-assembly of tetrahedron **3**. Encapsulated NTf_2^- in tetrahedron **3** can be observed in the ^{19}F NMR spectrum (Figure S14).

An analogous synthetic procedure as for tetrahedron **1** and **2** was applied for tetrahedron **3**. Triamine **D** (6.00 mg, 13.6 µmol, 4 equiv.), iron(II) triflimide (4.81 mg, 13.6 µmol, 4 equiv.), and aldehyde **C** (4.61 mg, 3.6 µL, 40.8 µmol, 12 equiv.) were dissolved in MeCN (1.5 mL), the solution degassed and stirred at room temperature overnight. A dark purple solid precipitated after the addition of diethyl ether to the assembly solution and was washed three times with diethyl ether prior to further characterization (yield: 11.2 mg, 76%). Dark purple single crystals were obtained for X-ray crystallography via slow vapor diffusion of diethyl ether into the solution.

However, signals for the cage were not detected by low-resolution ESI-MS. Only singly-charged species corresponding to cage fragments were observed, which might be attributed to the instability of the cage when diluted to very low concentration for mass spectrometry (less than 0.1 mg/mL). A similar situation was encountered in the HR-MS, where fragments comprised the majority of the signals. Cage signals can be observed only with very low intensity, however, one well-resolved isotope pattern was observed (Figure S17). Nevertheless, the DOSY spectrum reveals a similar diffusion coefficient (tetrahedron **3**: 6.72×10^{-10} m^2/s) as for the other two cages (tetrahedron **1**: 4.22×10^{-10} m^2/s, tetrahedron **2**: 3.51×10^{-10} m^2/s) and single crystals suitable for X-ray crystallography have been obtained to confirm its tetrahedral structure.

^1H NMR (400 MHz, 298 K, CD_3CN): δ 10.30, 9.08, 7.63, 7.52 (d, J = 8.4 Hz), 5.03 (d, J = 8.3 Hz), 3.44 ppm.

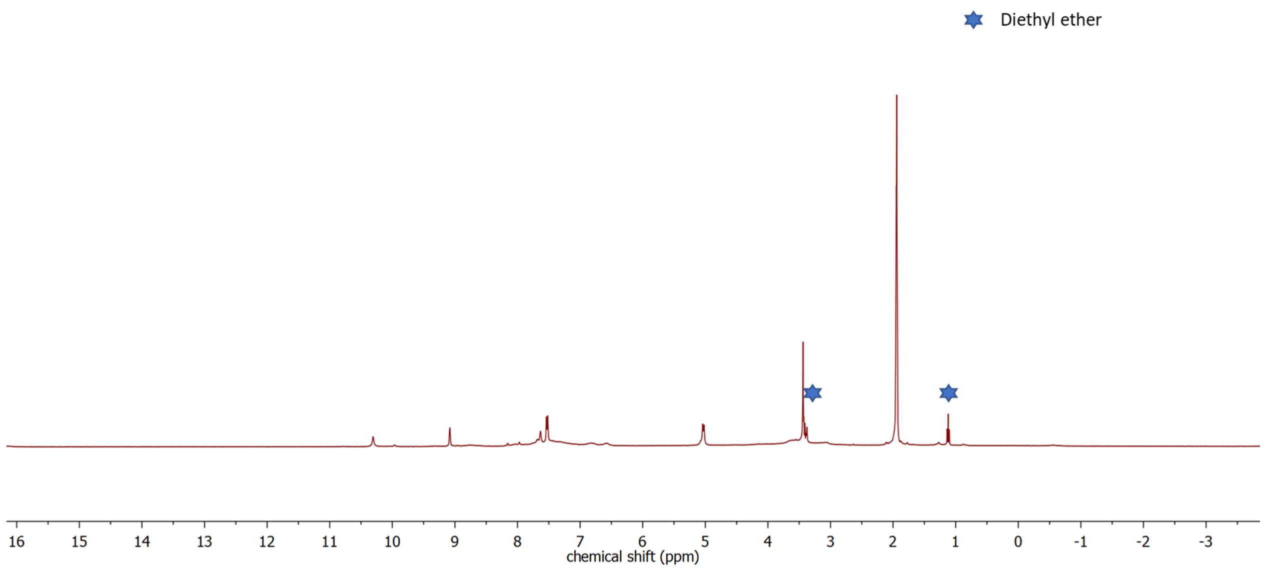

Figure S14 ^{1}H NMR spectrum of tetrahedron **3** (CD_3CN, 400 MHz, 298 K).

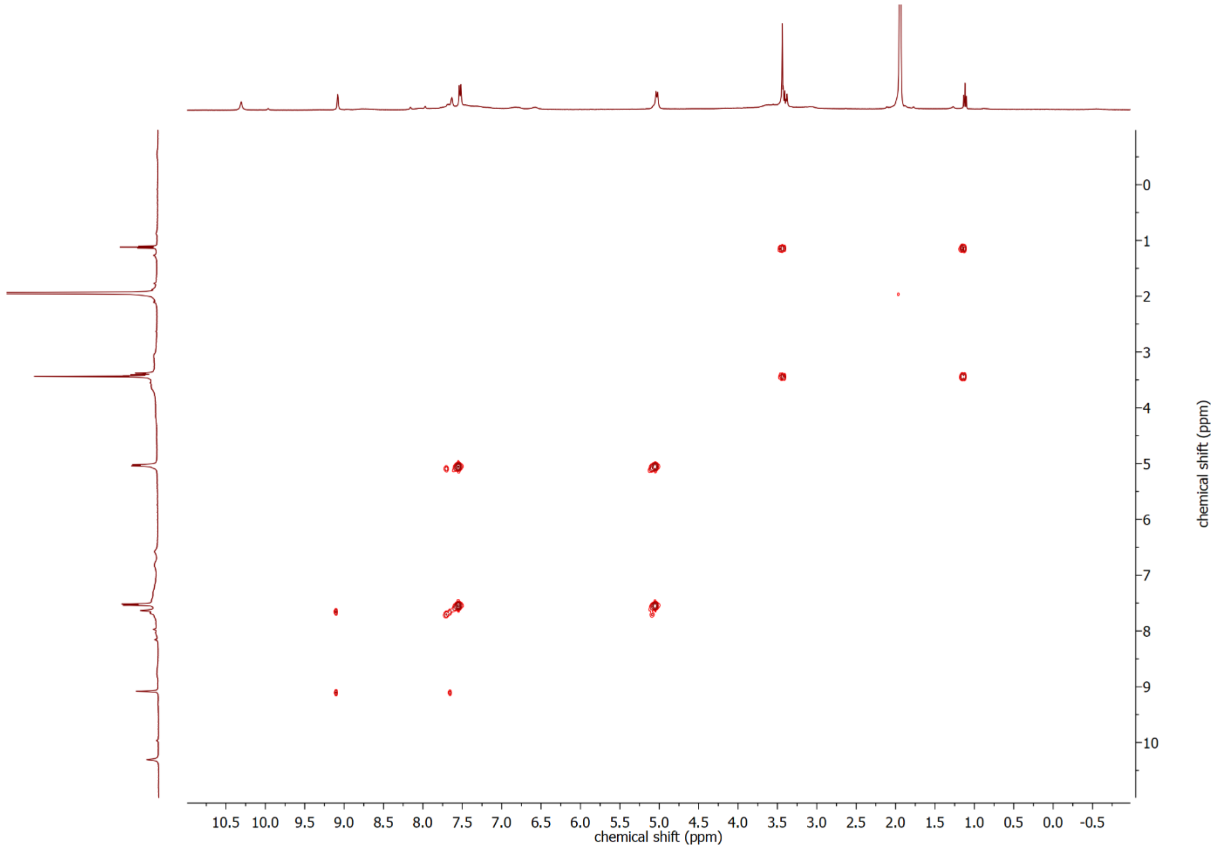

Figure S15 ^{1}H-^{1}H COSY spectrum of tetrahedron **3** (CD_3CN, 400 MHz, 298 K).

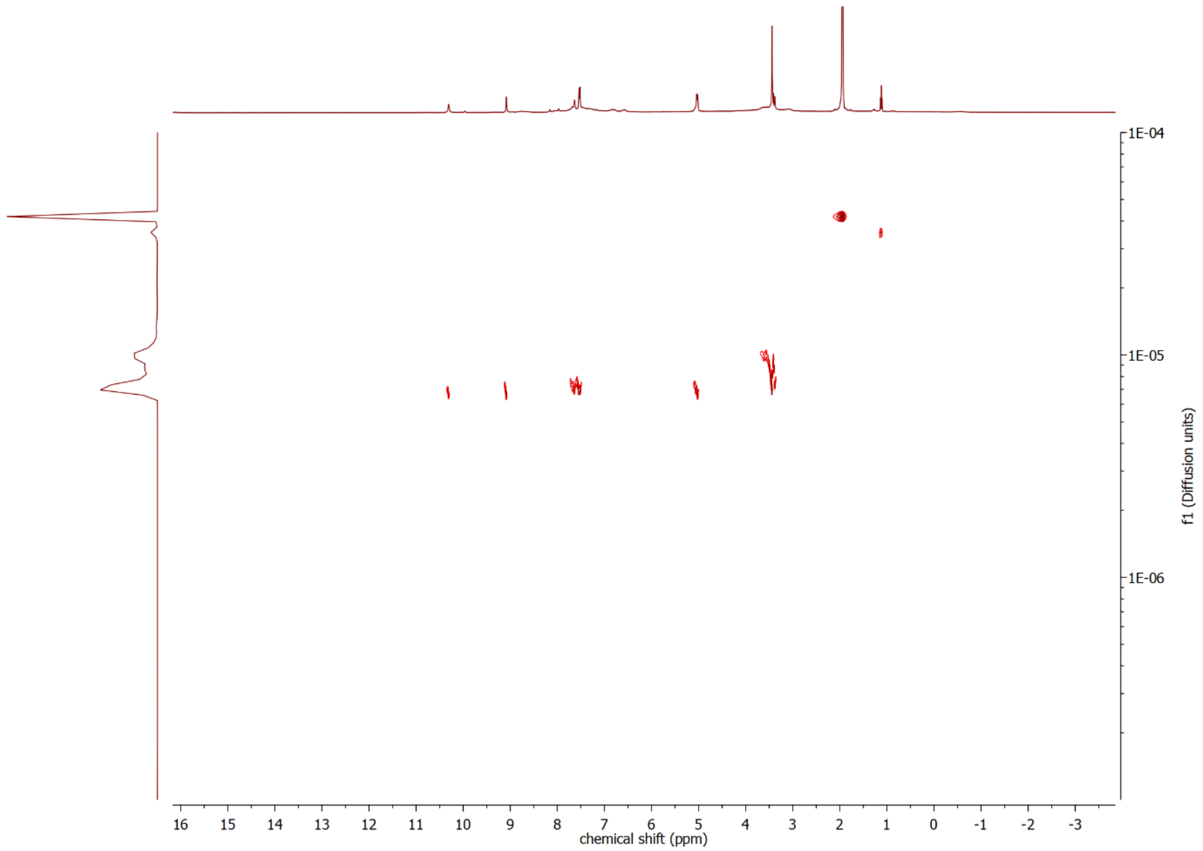

Figure S16 ^{1}H DOSY spectrum of tetrahedron **3** (CD_3CN, 400 MHz, 298 K).

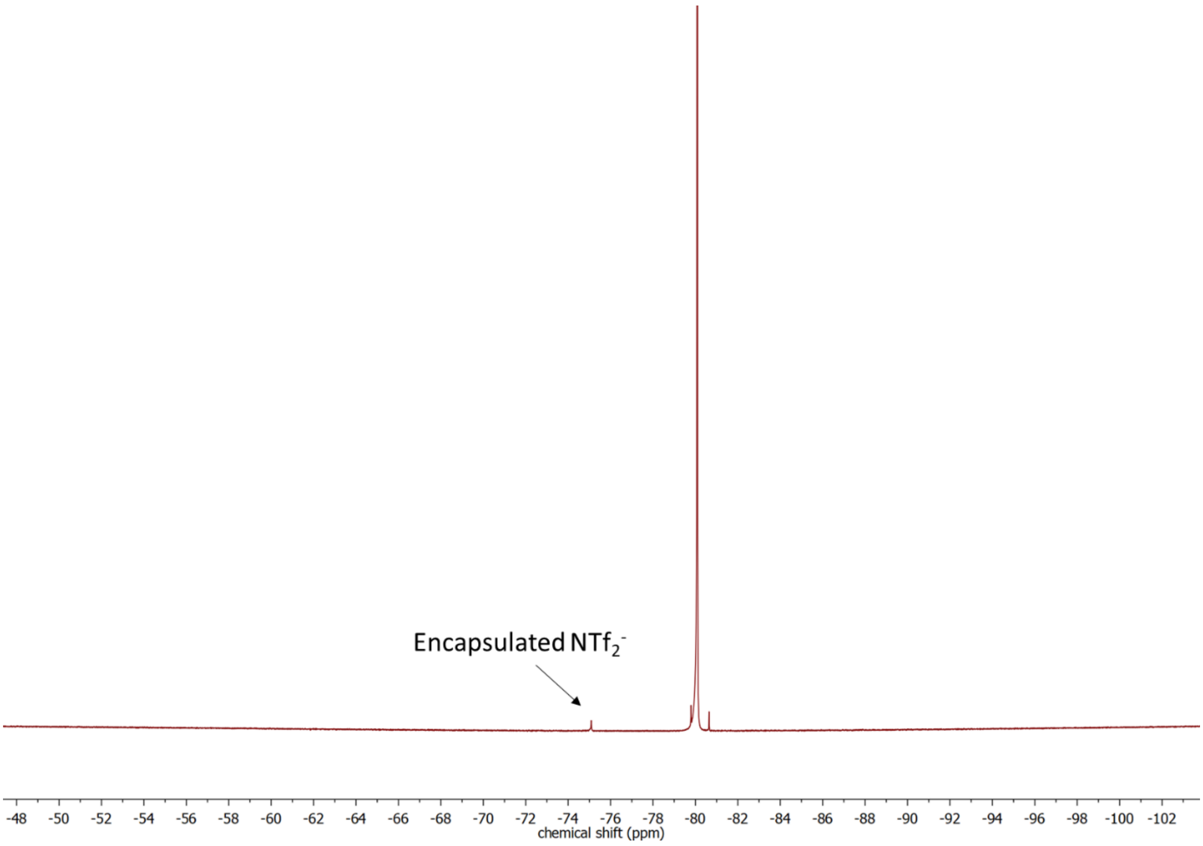

Figure S17 ^{19}F NMR spectrum of tetrahedron **3** (CD_3CN, 376 MHz, 298 K).

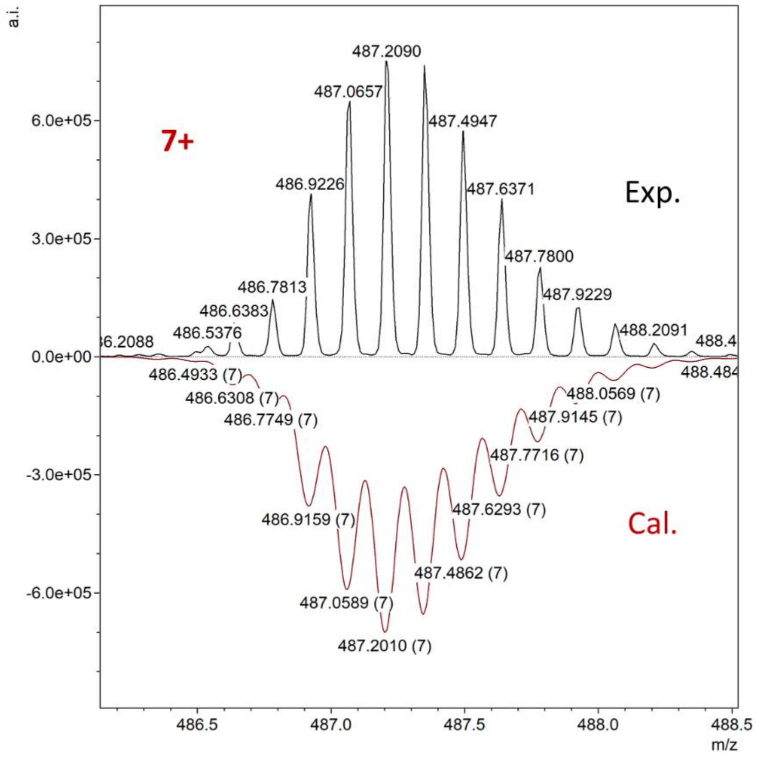

Figure S18 HRMS (ESI$^+$-QTOF, CH_3CN) of tetrahedron **3**.

2.4 Cube **4** and trigonal prism **5**

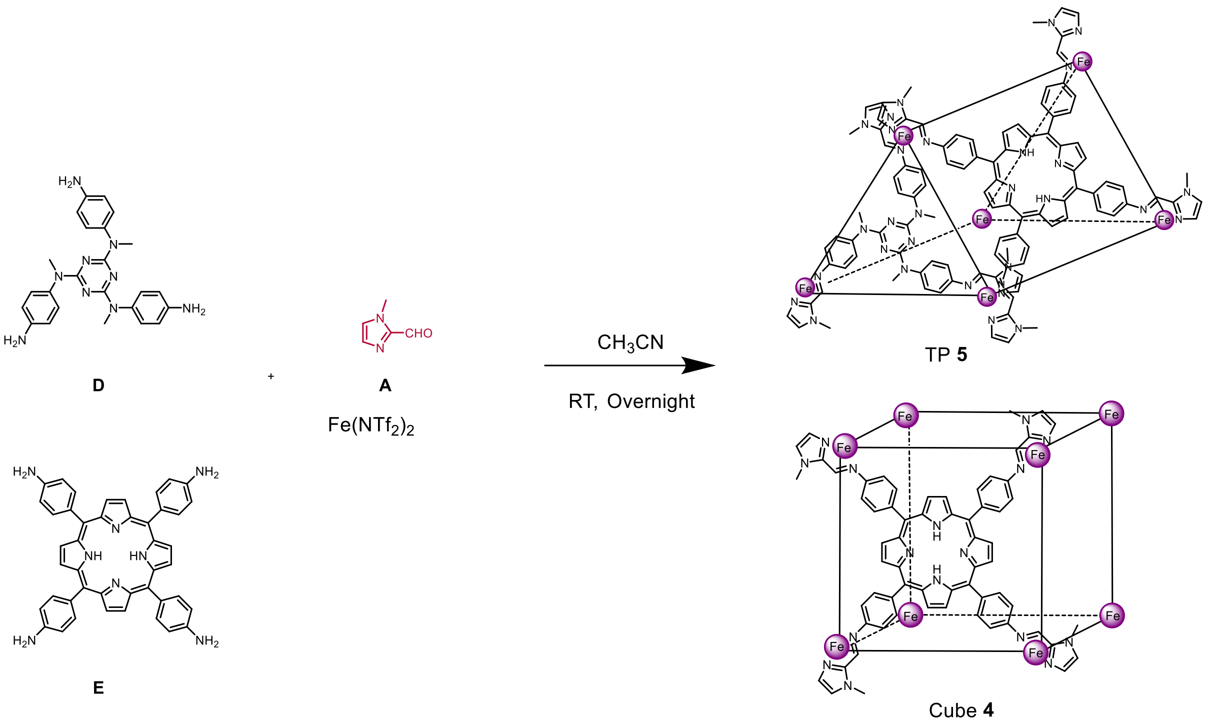

Scheme S4 Subcomponent self-assembly of trigonal prism **5** and cube **4**.

Triamine **D** (1.31 mg, 3.0 µmol, 2 equiv.), tetraamine **E** (5,10,15,20-Tetrakis(4-aminophenyl)porphyrin (3.00 mg, 4.0 µmol, 3 equiv.), iron(II) triflimide (5.48 mg, 9.0 µmol, 6 equiv.), and aldehyde **A** (2.94 mg, 27.0 µmol, 18 equiv.) were dissolved in MeCN (1 mL). The reaction mixture was degassed by three freeze-pump-thaw cycles then stirred overnight at room temperature. The product was precipitated with diethyl ether and washed with diethyl ether three times (The ratio of cube to trigonal prism is around **4** : **5** = 1.1 : 1 according to ^{1}H NMR integrations). Crystals of **4** and **5** were grown by diffusion of diethyl ether into an acetonitrile solution of the product mixture. However, the crystals of cube **4** only diffracted to low resolution and no reasonable solution was obtained. Crystals of trigonal prism **5** diffracted well and the structure was fully refined. In theory the formation of tetrahedron **1** should also be observed, but tetrahedron **1** seems to be less stable than the other structures involved in this equilibrium. A putative dynamic library containing the subcomponents of **1** was observed in the ^{1}H NMR spectrum from 6-10 ppm.

Cube **4** can also be obtained as the sole product via the self-assembly of tetraamine **E** (1 mg, 1.5 µmol, 6 equiv.), iron(II) triflimide (1.22 mg, 2.0 µmol, 8 equiv.), and aldehyde **A** (0.065 mg, 6.0 µmol, 24 equiv.) in MeCN. The yield was quite low which might be due to undefined fragments/intermediates like they were also observed in the self-assembly of tetrahedron **1**. These undefined intermediates/fragments also resulted in to quite low quality of spectra for cube **4**. Since cube **4** shows similar properties to another porphyrin-based SCO cage[7], it is not the focus of this work.

For trigonal prism **5**, which stays at the HS state, relaxation in NMR is quick and gives rise to very broad signals at room temperature. The ^{1}H NMR spectrum of trigonal prism **5** at 338 K displays sharper signals.

Peaks are assigned largely based on VT NMR (section 5.12), as signals of SCO cube **4** and HS trigonal prism **5** move towards opposite directions. 2D NMR characterizations are not as useful as for diamagnetic complexes for assignments here due to the wide distribution of peaks on the spectrum and fast relaxations of the HS state. ^{1}H DOSY was used to distinguish peaks corresponding to cages from fragments/intermediates.

^{1}H NMR Cube **4** (500 MHz, 298 K, CD_3CN): δ 43.97, 26.50, 25.22, 11.32, 10.30, 9.47, 3.89, −3.07 ppm.

^{1}H NMR trigonal prism **5** (500 MHz, 338 K, CD_3CN): δ 111.92, 109.16, 106.79, 67.09, 65.19, 62.36, 61.81, 59.36, 53.10, 17.04, 15.56, 14.43, 14.38, 14.08, 11.03, 9.58, 8.53, 5.42, −1.25, −3.13, −4.98,

−7.52 ppm. (Since the relaxation of **5** is very fast on the NMR time scale, some signals are very broad or merge with other signals and are hence hard to identify.)

LRMS (ESI$^+$-Q, CH_3CN) [charge, calculated for $Fe_8(C_{64}H_{50}N_{16})_6((NC_2S_2O_4F_6)_{16}]$: *m/z* = 519.2 [**4**$(NTf_2)_2^{14+}$ 519.0], 580.7 [**4**$(NTf_2)_3^{13+}$ 580.5], 652.4 [**4**$(NTf_2)_4^{12+}$ 652.2], 737.2 [**4**$(NTf_2)_5^{11+}$ 737.0], 838.9 [**4**$(NTf_2)_6^{10+}$ 838.8], 963.3 [**4**$(NTf_2)_7^{9+}$ 963.0], 1118.7 [**4**$(NTf_2)_8^{8+}$ 1118.4], 1318.5 [**4**$(NTf_2)_9^{7+}$ 1318.2], 1585.3 [**4**$(NTf_2)_{10}^{6+}$ 1584.6]

LRMS (ESI$^+$-Q, CH_3CN) [charge, calculated for $Fe_6(C_{39}H_{39}N_{15})_2(C_{64}H_{50}N_{16})_3((NC_2S_2O_4F_6)_{12}]$: *m/z* = 471.1 [**5**$(NTf_2)_1^{11+}$ 471.0], 546.2 [**5**$(NTf_2)_2^{10+}$ 546.0], 638.0 [**5**$(NTf_2)_3^{9+}$ 637.9], 752.8 [**5**$(NTf_2)_4^{8+}$ 752.6], 900.4 [**5**$(NTf_2)_5^{7+}$ 900.1], 1097.3 [**5**$(NTf_2)_6^{6+}$ 1096.9], 1372.5 [**5**$(NTf_2)_7^{5+}$ 1372.3]

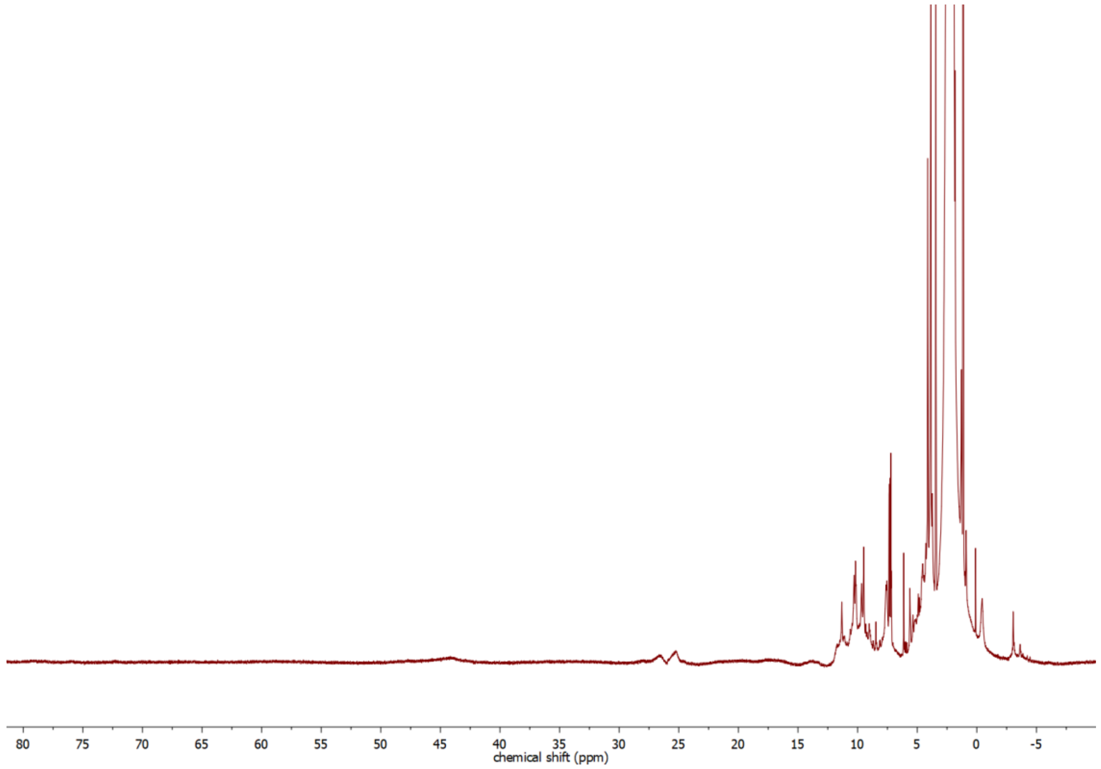

Figure S19 ^1H NMR spectrum of cube **4** (CD_3CN, 500 MHz, 298 K).

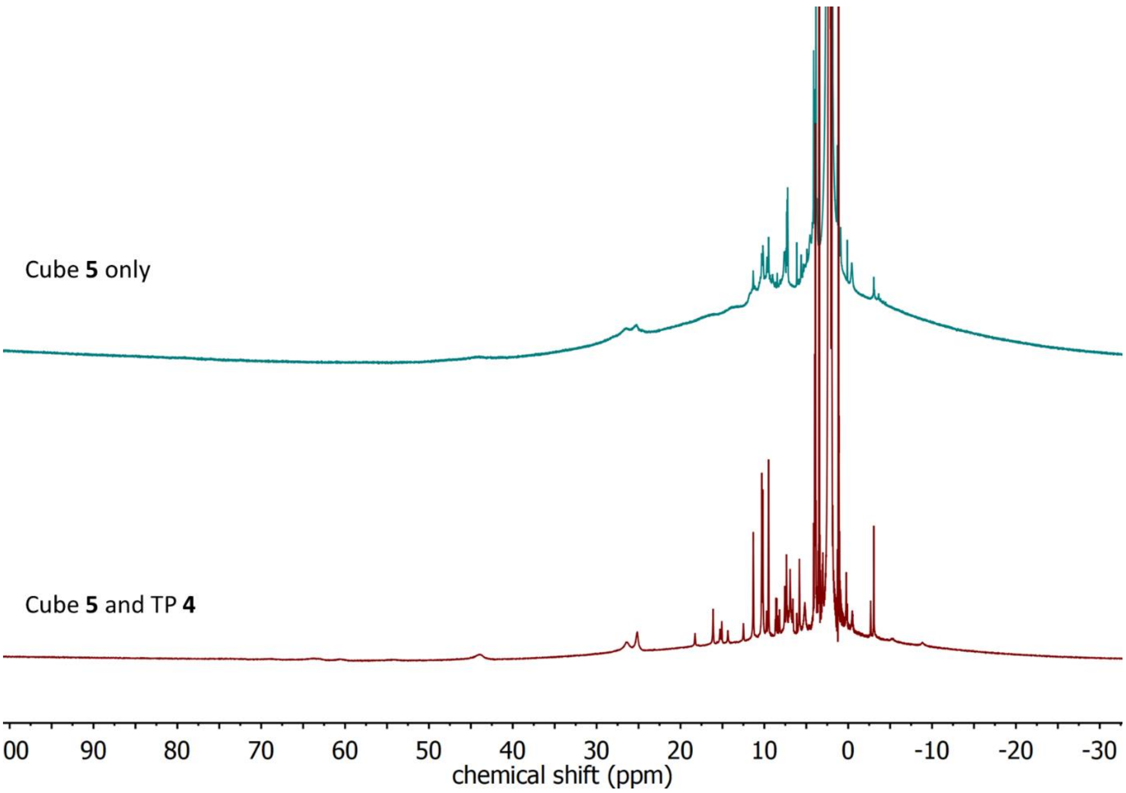

Figure S20 ^{1}H NMR spectra of a mixture of cube **4** and trigonal prism **5** (bottom) and cube **5** (top) (CD_3CN, 500 MHz, 298 K).

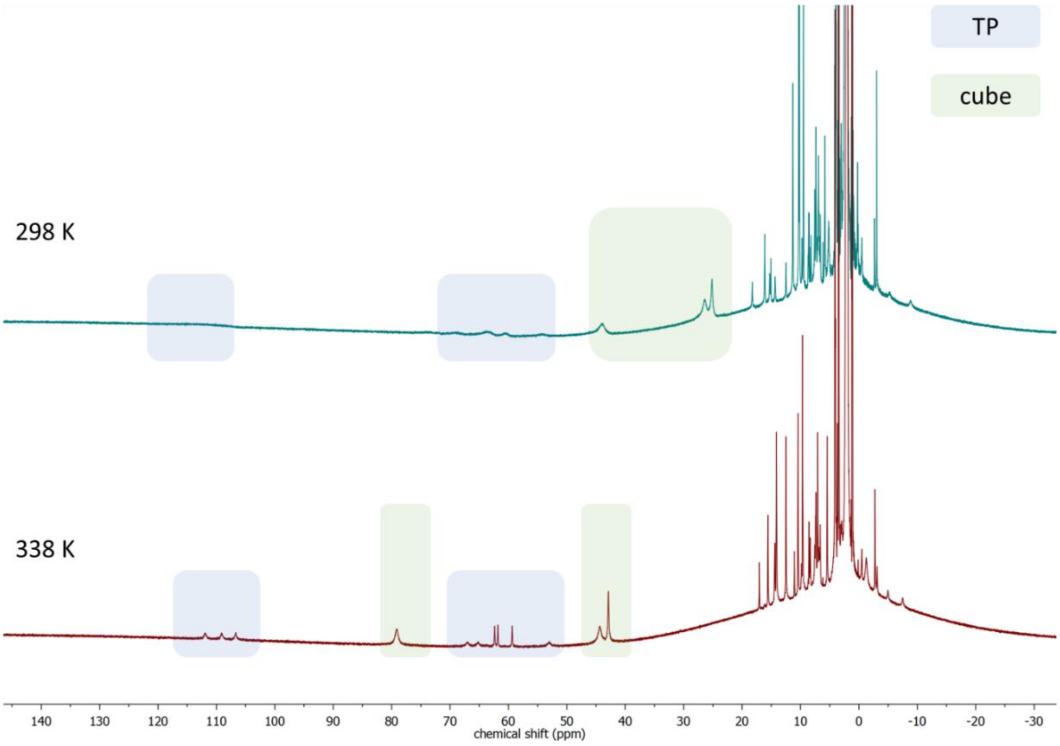

Figure S21 ^{1}H NMR spectra of a mixture of trigonal prism **5** and cube **4** (CD_3CN, 500 MHz, 298 K and 338 K).

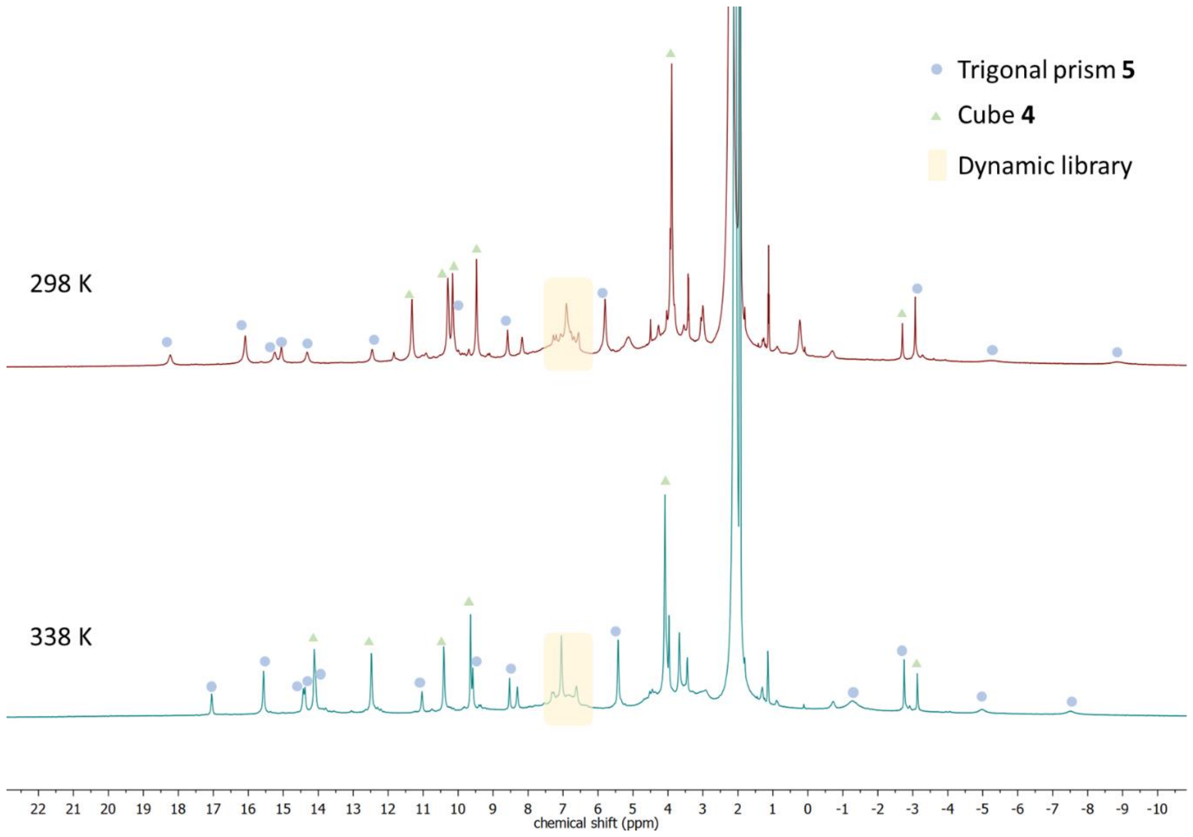

Figure S22 ^{1}H NMR spectra of a mixture of trigonal prism **5** and cube **4** in the diamagnetic region, Peaks corresponding to a putative dynamic library containing the subcomponents of **1** is marked with a yellow square (CD_3CN, 500 MHz, 298 K and 338 K).

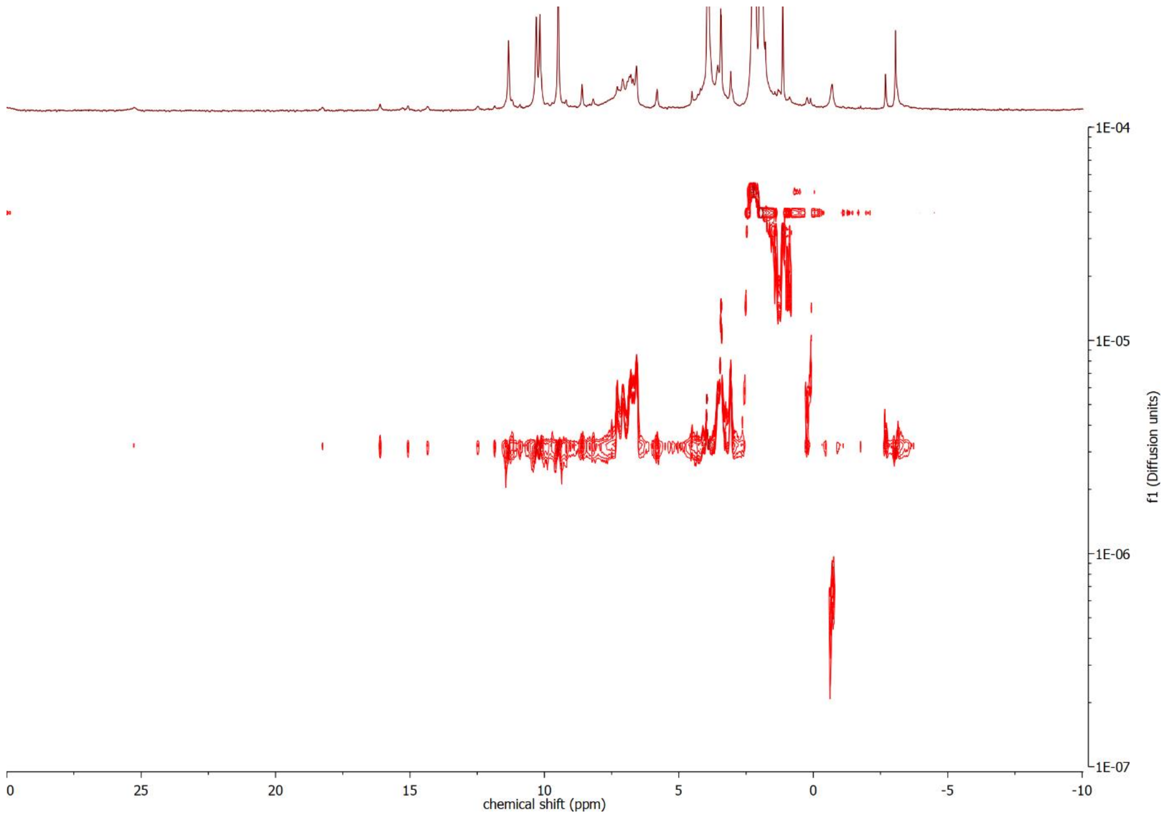

Figure S23 ^{1}H DOSY spectrum of a mixture of **4** and **5** (CD_3CN, 400 MHz, 298 K).

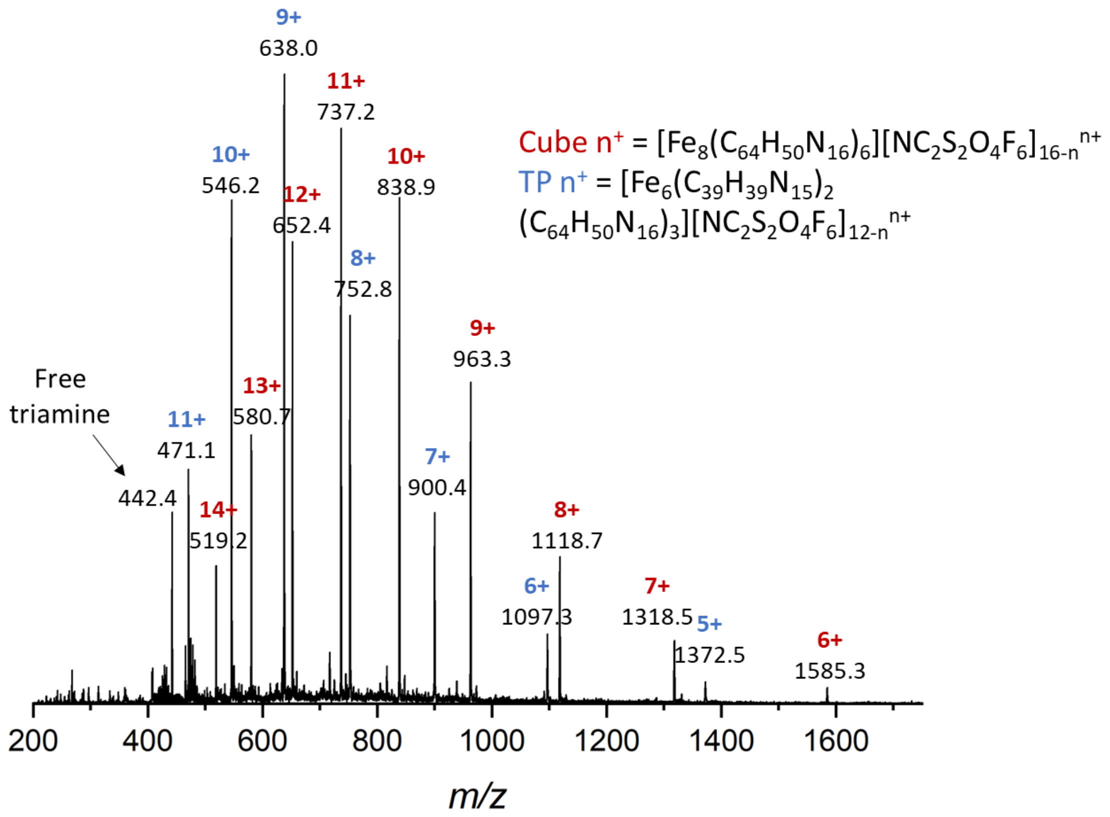

Figure S24 LRMS (ESI^+-Q, CH_3CN) of a mixture of trigonal prism **5** and cube **4**.

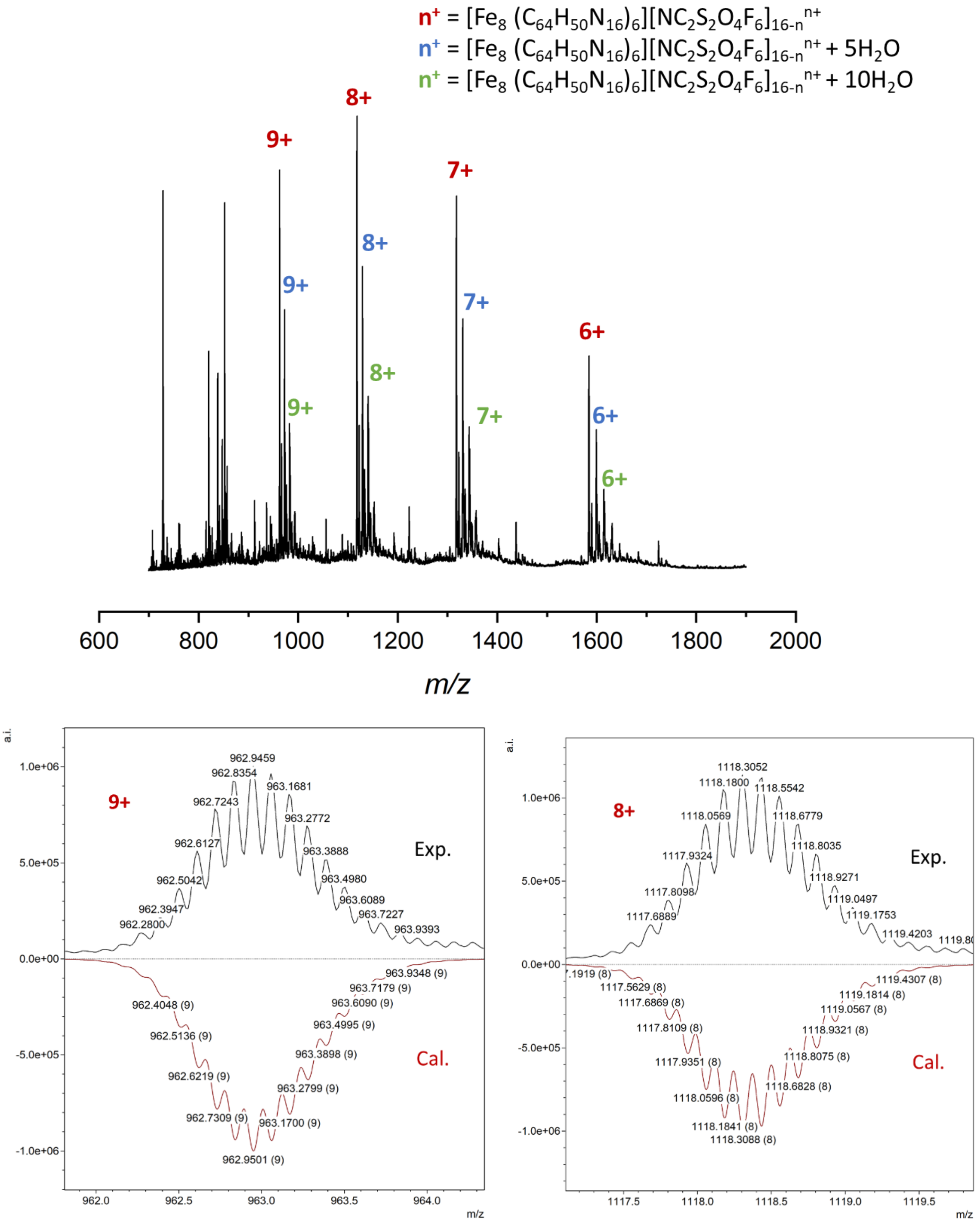

Figure S25 HRMS (ESI$^+$-QTOF, CH_3CN) of cube **4** only (full spectrum and peaks corresponding to 9+ and 8+, from top to bottom).

3 X-ray crystallography and volume calculations

Data for tetrahedron **1** was collected using a Bruker D8 VENTURE diffractometer equipped with high-brilliance IμS Cu-Kα radiation (1.54178 Å), with ω and ψ scans at 180(2) K, and the rest were collected at Beamline I19 of Diamond Light Source employing silicon double crystal monochromated synchrotron radiation (0.6889 Å) with ω scans at 100(2) K.[8] Data integration and reduction were undertaken with SAINT[9,10] and XPREP7 or Xia2.[11–13] Subsequent computations were carried out using the WinGX-32 graphical user interface[14] and Olex-2.[15] Multi-scan empirical absorption corrections were applied to the data using SADABS[16] or the AIMLESS[17] tool in the CCP4 suite.[18] Structures were solved by intrinsic phasing using SHELXT-2013[19] then refined and extended with SHELXL-2014.[20] In general, non-hydrogen atoms with occupancies greater than 0.5 were refined anisotropically. Carbon-bound hydrogen atoms were included in idealized positions and refined using a riding model. Disorder was modelled using standard crystallographic methods including constraints, restraints and rigid bodies where necessary (SIMU, ISOR, DFIX, etc). Crystallographic data along with specific details pertaining to the refinement follow. Crystallographic data have been deposited with the CCDC (2176135-2176139).

3.1 Tetrahedron 1

A B

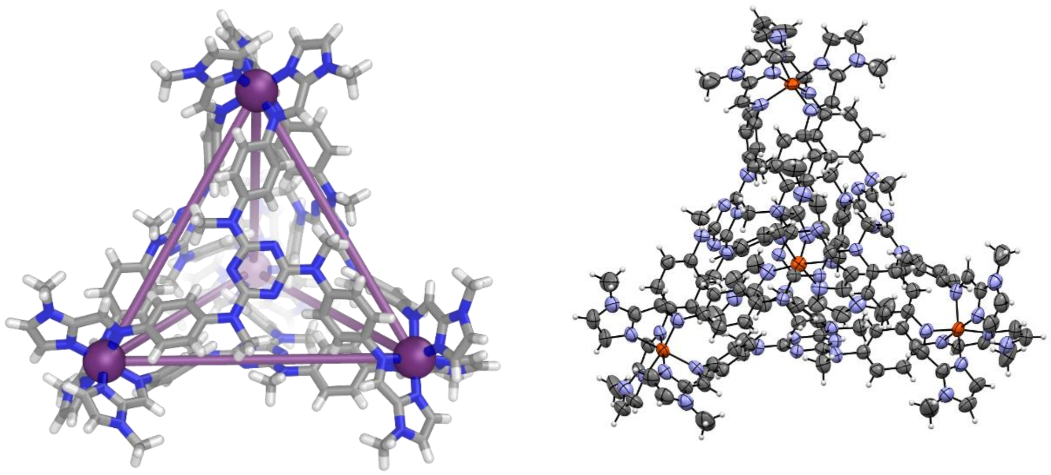

Figure S26 (A) X-ray crystal structure of tetrahedron **1**. (B) Ortep-style plot of the cationic portion of the crystal structure of tetrahedron **1**, showing thermal ellipsoids at a probability of 40%.

Formula $C_{156}H_{156}Fe_4N_{60}$, M 3094.80, cubic, space group I23 (#197), a 22.6087(6), b 22.6087(6), c 22.6087(6) Å, V 11556.5(9) Å^3 , D_c 1.205 g cm^{-3} , Z 2, temperature 180(2) Kelvin, λ(CuKα) 1.54178 Å, μ(CuKα) 2.677 mm^{-1}, crystal size 0.30 × 0.25 × 0.2 mm, color violet, habit needle, T(Analytical)$_{min,max}$ 0.6310, 0.7528, $2\theta_{max}$ 133.48, hkl range -24 19, -23 26, -26 26, N 27455, N_{ind} 3391(R_{int} 0.0784, R_{sigma} 0.0594), residuals* $R_1(F)$ 0.0487, $wR_2(F^2)$ 0.1529, GoF(all) 1.042, $\Delta\rho_{min,max}$ -0.20, 0.17 e Å^{-3}, Flack parameter 0.104(12).

* $R_1 = \Sigma||F_o|-|F_c||/\Sigma|F_o|$ for $F_o>2\sigma(F_o)$; $wR_2 = (\Sigma w(F_o^2-F_c^2)^2/\Sigma(wF_c^2)^2)^{1/2}$ all reflections

Specific refinement details:

The asymmetric unit of tetrahedron **1** was found to contain one twelfth of a Fe_4L_4 assembly. The anions and solvent molecules within the structure are highly disordered and solvent loss contributes to a significant amount of void volume in the lattice, which results in some smeared electron density. Consequently, the SQUEEZE[21] function of PLATON[22] was employed to remove the contribution of the electron density associated with anions and highly disordered solvent, which gave a potential solvent accessible void of 5117 Å^3 per unit cell (a total of approximately 1849 electrons). Thus, the molecular weight and density given above are underestimated.

3.2 Adamantane⊂**1**

A B

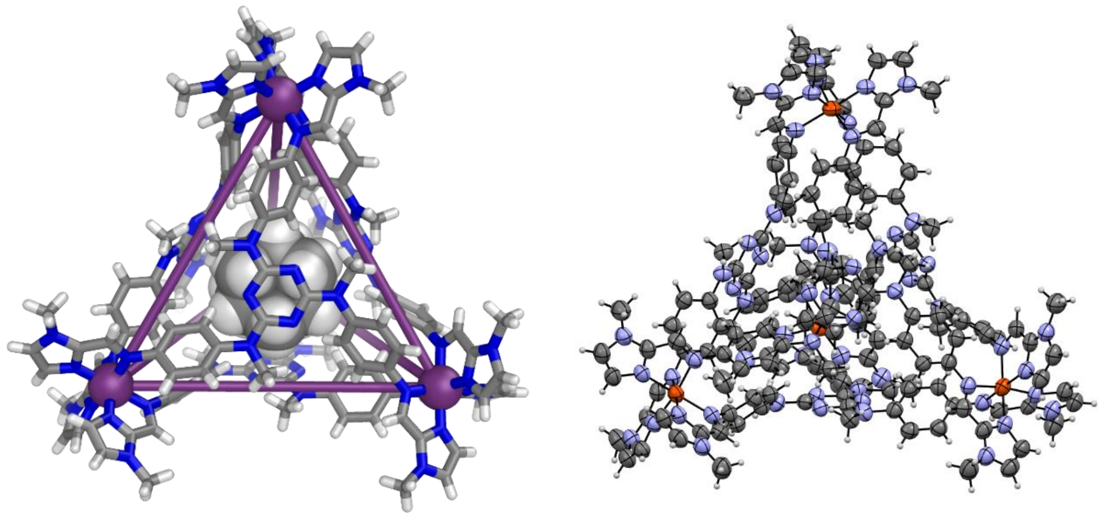

Figure S27 (A) X-ray crystal structure of adamantane⊂**1**. (B) Ortep-style plot of the cationic portion of the crystal structure of adamantane⊂**1**, showing thermal ellipsoids at a probability of 40%.

Formula $C_{166}H_{172}Fe_4N_{60}$, *M* 3231.03, cubic, space group *I*23 (#197), *a* 22.44400(10), *b* 22.44400(10), *c* 22.44400(10) Å, *V* 11305.79(15) Å^3 , D_c 0.949 g cm^{-3} , Z 2, temperature 100(2) Kelvin, λ(Synchrotron) 0.6889 Å, μ(Synchrotron) 0.296 mm^{-1} , $2\theta_{max}$ 71.976, crystal size 0.063 × 0.06 × 0.058 mm, color violet, habit block, $T(Analytical)_{min,max}$ 0.9826428693575137, 1 hkl range -36 37, -34 37, -25 37, *N* 89128, N_{ind} 9388(R_{int} 0.0564, R_{sigma} 0.0434), residuals* $R_1(F)$ 0.0486, $wR_2(F^2)$ 0.1748, GoF(all) 0.753, $\Delta\rho_{min,max}$ -0.20, 0.19 e Å^{-3}, Flack parameter 0.034(10).

* $R_1 = \Sigma||F_o|-|F_c||/\Sigma|F_o|$ for $F_o>2\sigma(F_o)$; $wR_2 = (\Sigma w(F_o^2-F_c^2)^2/\Sigma(wF_c^2)^2)^{1/2}$ all reflections

Specific refinement details:

The asymmetric unit of adamantane⊂**1** was found to contain one twelfth of a Fe_4L_4 assembly and the adamantane. The anions and solvent molecules within the structure are highly disordered and solvent loss contributes to a significant amount of void volume in the lattice, which results in some smeared electron density. Consequently, the SQUEEZE function of PLATON was employed to remove the contribution of the electron density associated with anions and highly disordered solvent, which gave a potential solvent accessible void of 4561 Å^3 per unit cell (a total of approximately 1483 electrons). Thus, the molecular weight and density given above are underestimated.

3.3 Adamantane⊂**2**

A B

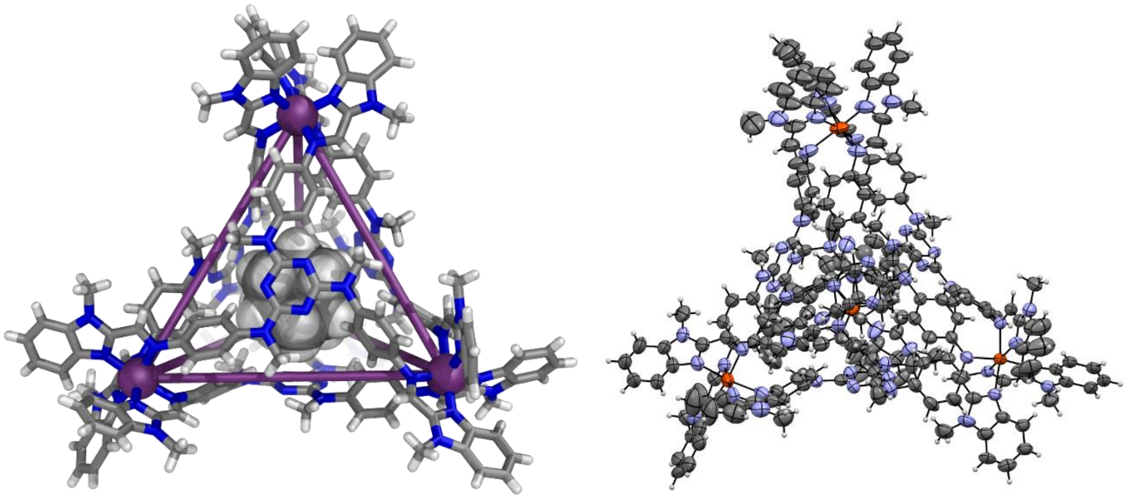

Figure S28 (A) X-ray crystal structure of adamantane⊂**2** (counterions are omitted for clarity). (B) Ortep-style plot of the cationic portion of the crystal structure of adamantane⊂**2**, showing thermal ellipsoids at a probability of 40%.

Formula $C_{217}H_{197}F_9Fe_4N_{60}O_9S_3$, *M* 4278.91, monoclinic, space group *C2/c* (#15), *a* 39.5898(2), *b* 21.64050(10), *c* 61.9145(4) Å, *β* 98.7340(10)°, V 52429.7(5) Å^3, D_c 1.171 g cm^{-3}, Z 8, temperature 100(2) Kelvin, λ(Synchrotron) 0.6889 Å, μ(Synchrotron) 0.280 mm^{-1}, $2\theta_{max}$ 47.812, crystal size 0.052 × 0.05 × 0.048 mm, color violet, habit block, T(Analytical)$_{min,max}$ 0.9762696595594602, 1, hkl range -46 46, -25 25, -69 72, *N* 230232, N_{ind} 44331(R_{int} 0.0566, R_{sigma} 0.0511), residuals* $R_1(F)$ 0.0942, $wR_2(F^2)$ 0.3013, GoF(all) 1.060, $\Delta\rho_{min,max}$ -0.75, 0.075 e Å^{-3}.

* $R_1 = \Sigma||F_o|-|F_c||/\Sigma|F_o|$ for $F_o>2\sigma(F_o)$; $wR_2 = (\Sigma w(F_o^2-F_c^2)^2/\Sigma(wF_c^2)^2)^{1/2}$ all reflections

Specific refinement details:

The asymmetric unit of adamantane⊂**2** was found to contain a complete Fe_4L_4 assembly and an adamantane with associated counterions. One of the triflate anions and two benzimidazole rings were modelled as disordered over two locations.

The anions and solvent molecules within the structure are highly disordered and solvent loss contributes to a significant amount of void volume in the lattice, which results in some smeared electron density. Consequently, the SQUEEZE function of PLATON was employed to remove the contribution of the electron density associated with anions and highly disordered solvent, which gave a potential solvent accessible void of 16161 Å^3 per unit cell (a total of approximately 5586 electrons). Thus, the molecular weight and density given above are underestimated.

3.4 Tetrahedron **3**

A B

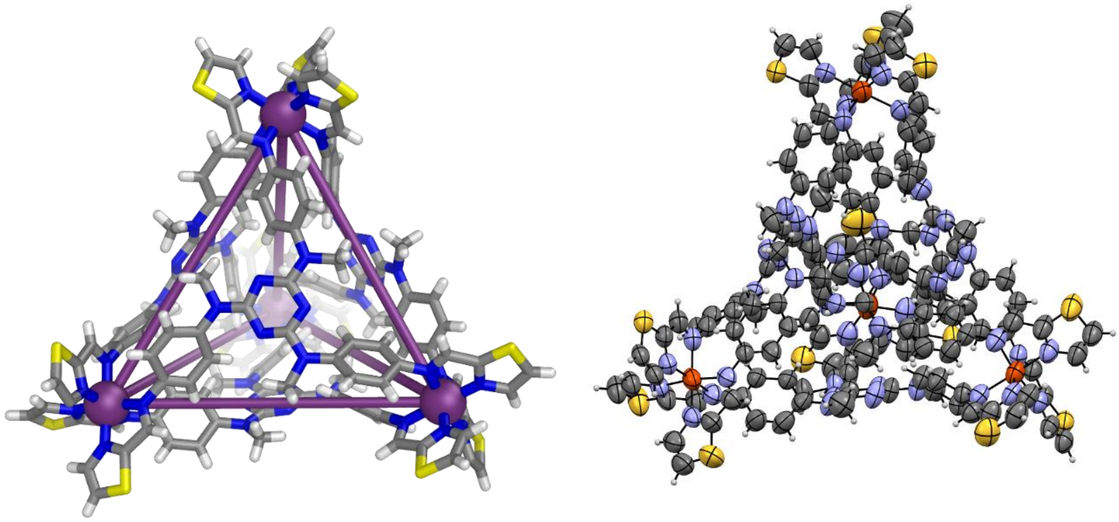

Figure S29 (A) X-ray crystal structure of **3** (counterions are omitted for clarity). (B) Ortep-style plot of the cationic portion of the crystal structure of tetrahedron **3**, showing thermal ellipsoids at a probability of 40%.

Formula $C_{152}H_{128}F_{12}Fe_{4}N_{52}O_{8}S_{16}$, *M* 3773.40, trigonal, space group *R-3c* (#167), *a* 38.26750(10), *b* 38.26750(10), *c* 94.2929(4) Å, V 119583.0(8) Å^3 , D_c 0.944 g cm^{-3} , Z 18, temperature 100(2) Kelvin, λ(Synchrotron) 0.6889 Å, μ(Synchrotron) 0.359 mm^{-1} , $2\theta_{max}$ 40.296, crystal size 0.063 × 0.06 × 0.058 mm, color violet, habit block, $T(Analytical)_{min,max}$ 0.992173297217262, 1, hkl range -38 38, -38 38, -94 90, *N* 132152, N_{ind} 13907(R_{int} 0.0571, R_{sigma} 0.0369), residuals* $R_1(F)$ 0.1118, $wR_2(F^2)$ 0.3714, GoF(all) 1.312, $\Delta\rho_{min,max}$ -0.57, 0.68 e Å^{-3}.

* $R_1 = \Sigma||F_o|-|F_c||/\Sigma|F_o|$ for $F_o>2\sigma(F_o)$; $wR_2 = (\Sigma w(F_o^2-F_c^2)^2/\Sigma(wF_c^2)^2)^{1/2}$ all reflections

Specific refinement details:

Despite the use of synchrotron radiation few reflections at greater than 1.0 Å resolution were observed and the data were trimmed accordingly.

The asymmetric unit of **3** was found to contain one twelfth of a Fe_4L_4 assembly and the adamantane. Thermal parameter restraints (SIMU, RIGU) were applied to all atoms except for iron. The anions and solvents within the structure are highly disordered and solvent loss contributes to a significant amount of void volume in the lattice, which results in some smeared electron density. Consequently, the SQUEEZE function of PLATON was employed to remove the contribution of the electron density associated with the remaining anions and highly disordered solvent, which gave a potential solvent accessible void of 56259 Å^3 per unit cell (a total of approximately 25018 electrons). Thus, the molecular weight and density given above are underestimated.

3.5 Trigonal prism **5**

A B

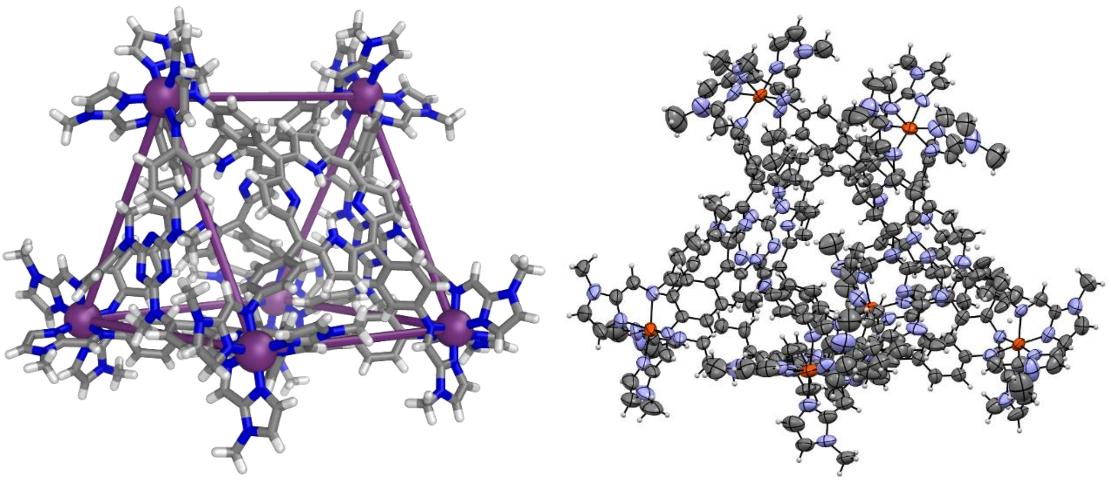

Figure S30 (A) X-ray crystal structure of **5** (counterions are omitted for clarity). (B) Ortep-style plot of the cationic portion of the crystal structure of trigonal prism **5**, showing thermal ellipsoids at a probability of 40%.

Formula $C_{282}H_{228}F_{36}Fe_6N_{84}O_{24}S_{12}$, *M* 6581.29, orthorhombic, space group *Fdd*2 (#43), *a* 67.5820(3), *b* 26.66660(10), *c* 47.6240(2) Å, *V* 85827.1(6) $Å^3$, D_c 1.019 g cm^{-3}, Z 8, temperature 100(2) Kelvin, λ(Synchrotron) 0.6889 Å, μ(Synchrotron) 0.297 mm^{-1}, $2\theta_{max}$ 58.954, crystal size 0.053 × 0.05 × 0.045 mm, color violet, habit block, $T(Analytical)_{min,max}$ 0.9887060084380634, 1 hkl range -96 96, -38 38, -68 68, *N* 372837, N_{ind} 9388(R_{int} 0.0360, R_{sigma} 0.0550), residuals* $R_1(F)$ 0.0543, $wR_2(F^2)$ 0.1597, GoF(all) 0.755, $\Delta\rho_{min,max}$ -0.37, 0.55 e $Å^{-3}$, Flack parameter 0.031(3).

* $R_1 = \Sigma||F_o|-|F_c||/\Sigma|F_o|$ for $F_o>2\sigma(F_o)$; $wR_2 = (\Sigma w(F_o^2-F_c^2)^2/\Sigma(wF_c^2)^2)^{1/2}$ all reflections

Specific refinement details:

The asymmetric unit was found to contain half of a Fe_6 $Fe_6(C_{39}H_{39}N_{15})_2(C_{64}H_{50}N_{16})_3$ assembly and associated counter ions. Two phenyl rings on the triazine ligand were modelled as disordered over two locations with bond length and thermal parameter restraints applied to facilitate a reasonable refinement.

The anions and solvent molecules within the structure are highly disordered and the solvent loss contribute to a significant amount of void volume in the lattice, which results in some smeared electron density. Consequently, the SQUEEZE function of PLATON was employed to remove the contribution of the electron density associated with anions and highly disordered solvent, which gave a potential solvent accessible void of 35043 $Å^3$ per unit cell (a total of approximately 13583 electrons). Thus, the molecular weight and density given above are underestimated.

3.6 Volume calculations

Cavity volumes were calculated with MoloVol 1.0.0[23] based on the crystal structures. For adamantane⊂**1** and adamantane⊂**2**, their volumes were calculated after omitting the encapsulated guests. The following parameters were used in the calculations:

Probe mode:	one probe
Probe radius:	1.4 Å
Grid resolution:	0.1 Å
Optimization depth:	4
Element radii:	N: 1.66 Å
	H: 1.2 Å
	Fe: 2.44 Å
	C: 1.77 Å
	S: 1.89 Å

Table S2 The cavity volumes of the cages

**cage**	**cavity volume** / Å^3
**1**	227
Adamantane⊂**1**	223
Adamantane⊂**2**	241
**3**	236
**4**	622

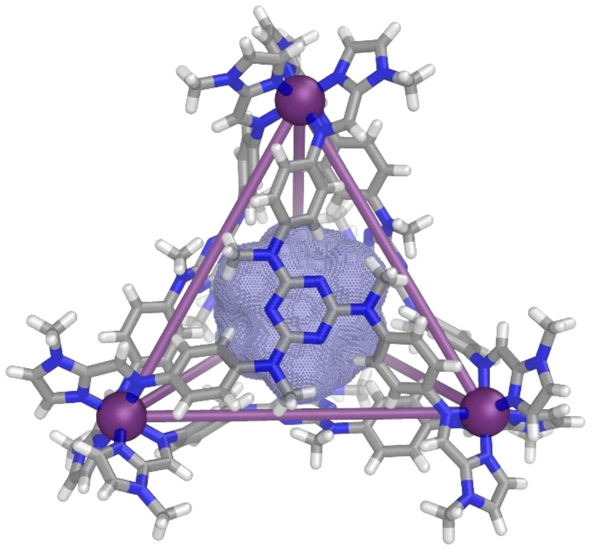

Figure S31 Crystal structure and cavity of tetrahedron **1** (cavity volume: 227 Å^3).

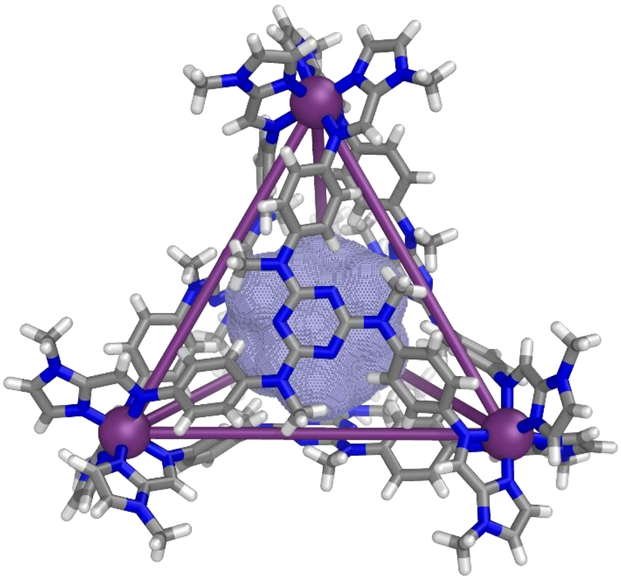

Figure S32 Crystal structure and cavity of adamantane⊂**1** (cavity volume: 223 Å^3).

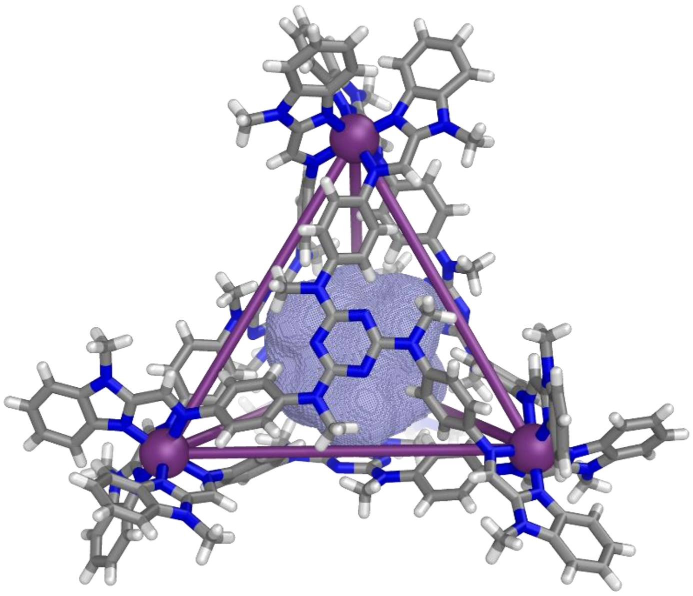

Figure S33 Crystal structure and cavity of adamantane⊂**2** (cavity volume: 241 Å^3).

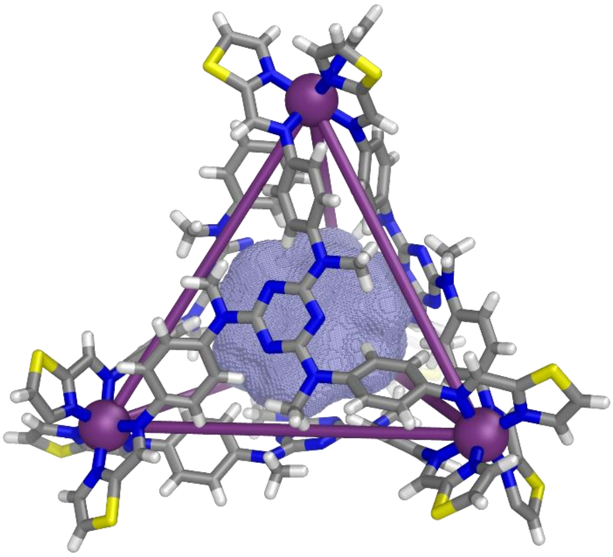

Figure S34 Crystal structure and cavity of **3** (cavity volume: 236 Å^3).

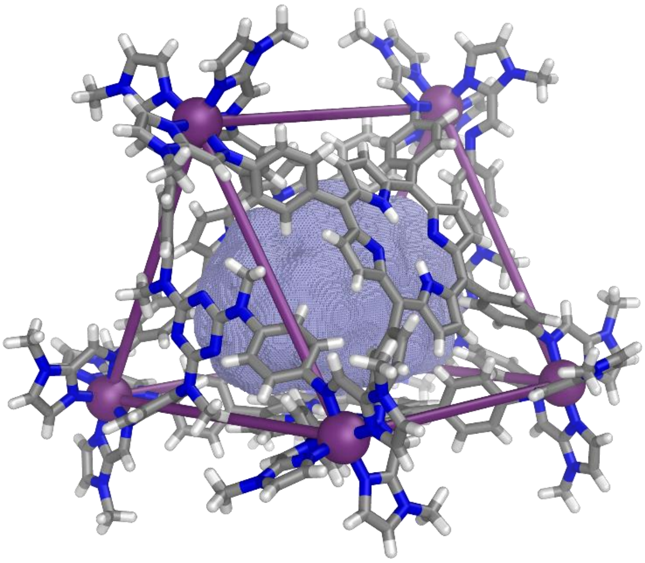

Figure S35 Crystal structure and cavity of **4** (622 Å^3).

Guest volumes were calculated with MoloVol 1.0.0[23] (Table S3) based on molecular force field simulations (MM2 force field) obtained with Scrigress 3.4.5 (Fujitsu Limited, Figure S36). The following parameters were used in the volume calculations:

Probe mode:	one probe
Probe radius:	1.4 Å
Grid resolution:	0.1 Å
Optimization depth:	4
Element radii:	O: 1.5 Å
	H: 1.2 Å
	C: 1.77 Å

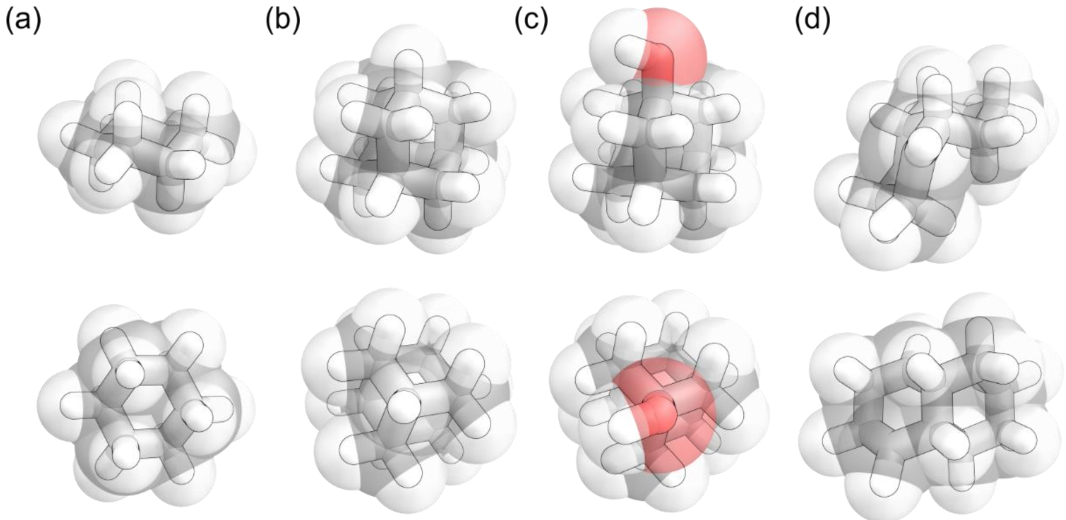

Figure S36 Side and top views of MM2 force field molecular models of (a) cyclohexane, (b) adamantane, (c) 1-adamantanol, (d) *cis*-decalin.

Van der Waals volumes V_{vdW} and molecular volumes V_{mol} (including cavities) were calculated (Table S3). Considering the molecular volume V_{mol} when comparing sizes of mono-, bi-, and tetracyclic hydrocarbons is crucial, because their cavities will not appear in the in their van der Waals volumes but have a considerable contribution to their sizes. The molecular volumes of the investigated guests increase in the order
cyclohexane < adamantane < *cis*-decalin < 1-adamantanol.

However, due to its open, convex structure, *cis*-decalin has larger dimensions than 1-adamantanol albeit having a smaller volume. The empty space within the convex side of *cis*-decalin's structure does not contribute to its volume, but that "void" is not accessible to the cage cavity either, rendering *cis*-decalin larger than 1-adamantanol. To correct for that discrepancy between volume and size, we introduce the sphericity Ψ[24] (Equation S2):

$$\Psi = \frac{\pi^{\frac{1}{3}} \cdot (6\,V_{\mathrm{mol}})^{\frac{2}{3}}}{S_{\mathrm{excl}}} \qquad \text{(S2.)}$$

with V_{mol} being the molecular volume of the guest, and S_{excl} the probe excluded molecular surface (Table S3).[23] The higher the sphericity of a non-tetrahedral guest at a given volume, the better the match to a tetrahedral or nearly spherical cavity.

When considering the sphericity corrected molecular volumes $V_{\mathrm{mol}} \cdot \Psi^{-1}$ (Table S3), 1-adamantanol and *cis*-decalin, which have virtually the same van der Waals (both 158 Å^3) and molecular volumes (169 and 167 Å^3, respectively), the sphericity corrected molecular volumes $V_{\mathrm{mol}} \cdot \Psi^{-1}$ give a good estimation of the size distribution: compact 1-adamantanol is the second to largest (189 Å^3) and *cis*-decalin being the largest guest in the series (195 Å^3).

Table S3 Guest volumes calculated with MoloVol 1.0.0[23] based on molecular force field simulations (MM2 force field) obtained with Scrigress 3.4.5 (Fujitsu Limited) and occupancy of LS **1**'s and "HS **1**'s" cavities (227 Å^3 and 241 Å^3, respectively).

	V_{vdw} [Å^3]	V_{mol} [Å^3]	S_{vdw} [Å^2]	S_{excl} [Å^2]	Ψ	$V_{mol}\cdot\Psi^{-1}$ [Å^3]	occupancy of LS 1/HS 1 [%]
cyclohexane	105	110	133	126	0.89	125	55
adamantane	151	160	173	158	0.90	178	78
1-adamantanol	158	169	182	165	0.89	189	83
cis-decalin	158	167	186	171	0.86	195	86 / 81

4 Host-Guest Chemistry

Preparation of host-guest complexes: 10-20 equivalents of the respective guest were added a solution of the respective cage in CD_3CN (approx. 0.5 mM, 0.4−0.5 mL). The solution was transferred to an NMR tube, then left to equilibrate at 298 K for 15 min.

4.1 Adamantane⊂**1**

^1H NMR (400 MHz, 298 K, CD_3CN): δ 51.94 (H_a), 29.05 (H_d), 28.65 (H_c), 10.80 (H_f), 4.33 (H_g), 4.01 (H_b), 3.04 (encapsulated adamantane, H_x), 2.66 (encapsulated adamantane, H_y), 1.60 (H_e) ppm.

LRMS (ESI^+-Q, CH_3CN) [charge, calculated for $(C_{10}H_{16})Fe_4(C_{39}H_{39}N_{15})_4(CF_3SO_3)_8$]: *m/z* = 403.8 [adamantane⊂$\mathbf{1}^{8+}$ 403.9], 483.0 [adamantane⊂$\mathbf{1}(OTf)_1^{7+}$ 482.8], 588.2 [adamantane⊂$\mathbf{1}(OTf)_2^{6+}$ 588.2], 735.7 [adamantane⊂$\mathbf{1}(OTf)_3^{5+}$ 735.6,], 956.8 [adamantane⊂$\mathbf{1}(OTf)_4^{4+}$ 956.8].

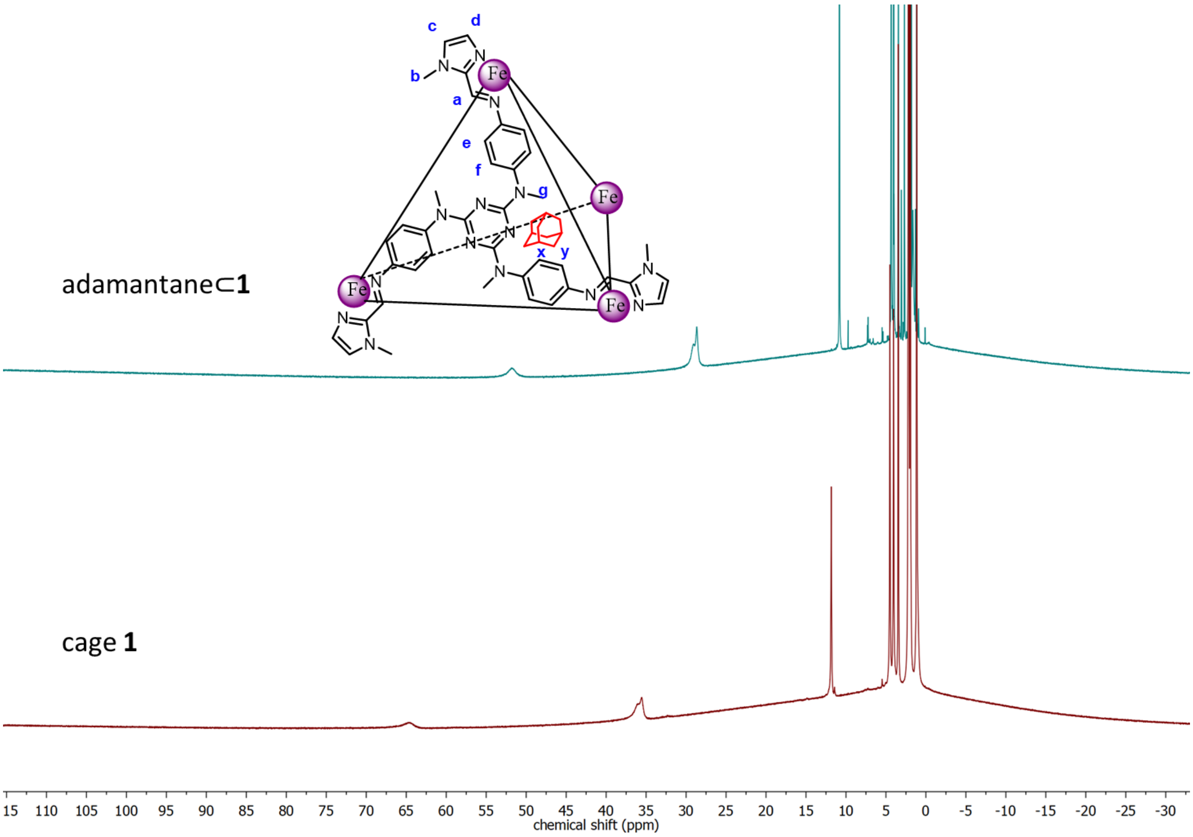

Figure S37 ^1H NMR spectra of empty cage **1** (bottom) and adamantane⊂**1** (top) (CD_3CN, 400 MHz, 298 K).

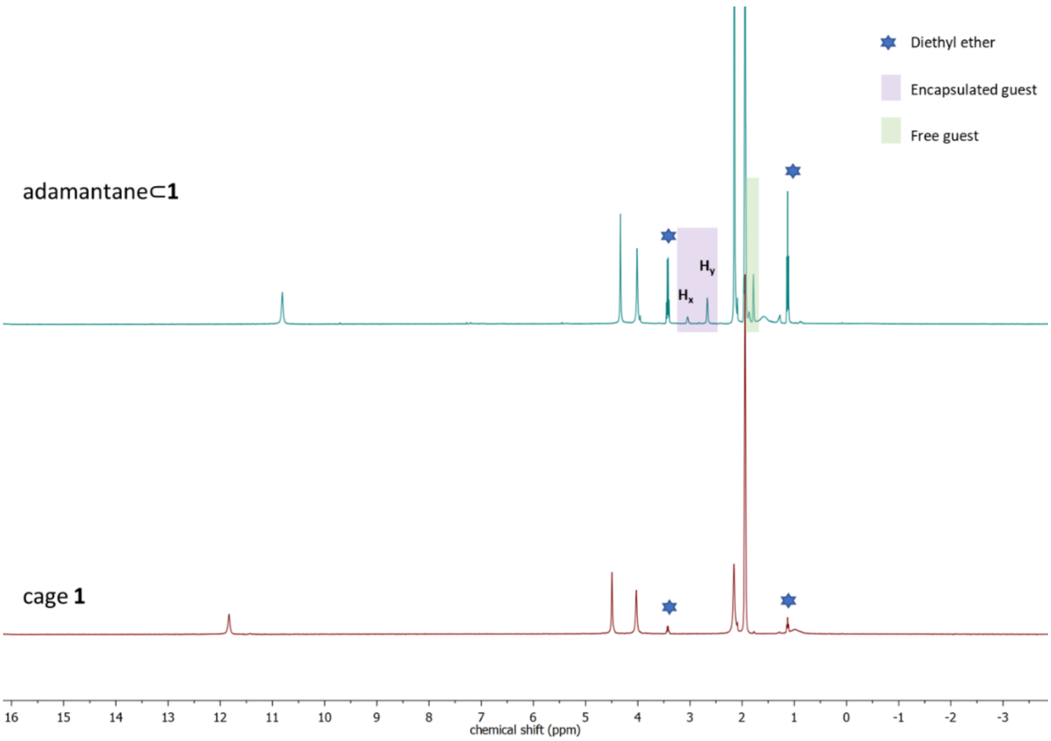

Figure S38 ^{1}H NMR spectrum of adamantane⊂**1** in the diamagnetic region (CD_3CN, 400 MHz, 298 K).

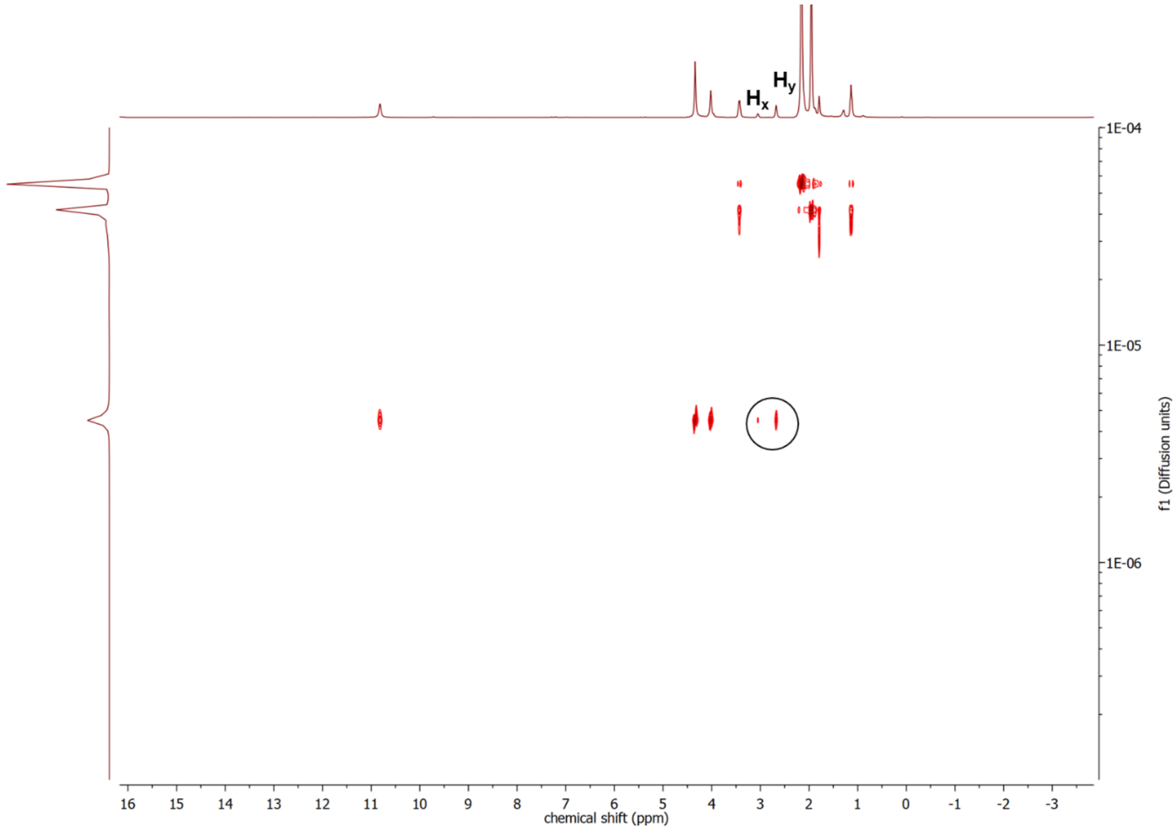

Figure S39 ^{1}H DOSY spectrum of adamantane⊂**1** in the diamagnetic region (CD_3CN, 400 MHz, 298 K). The black circle shows that the proton signals for encapsulated adamantane (H_x, H_y) diffuse at the same rate as the cage's protons.

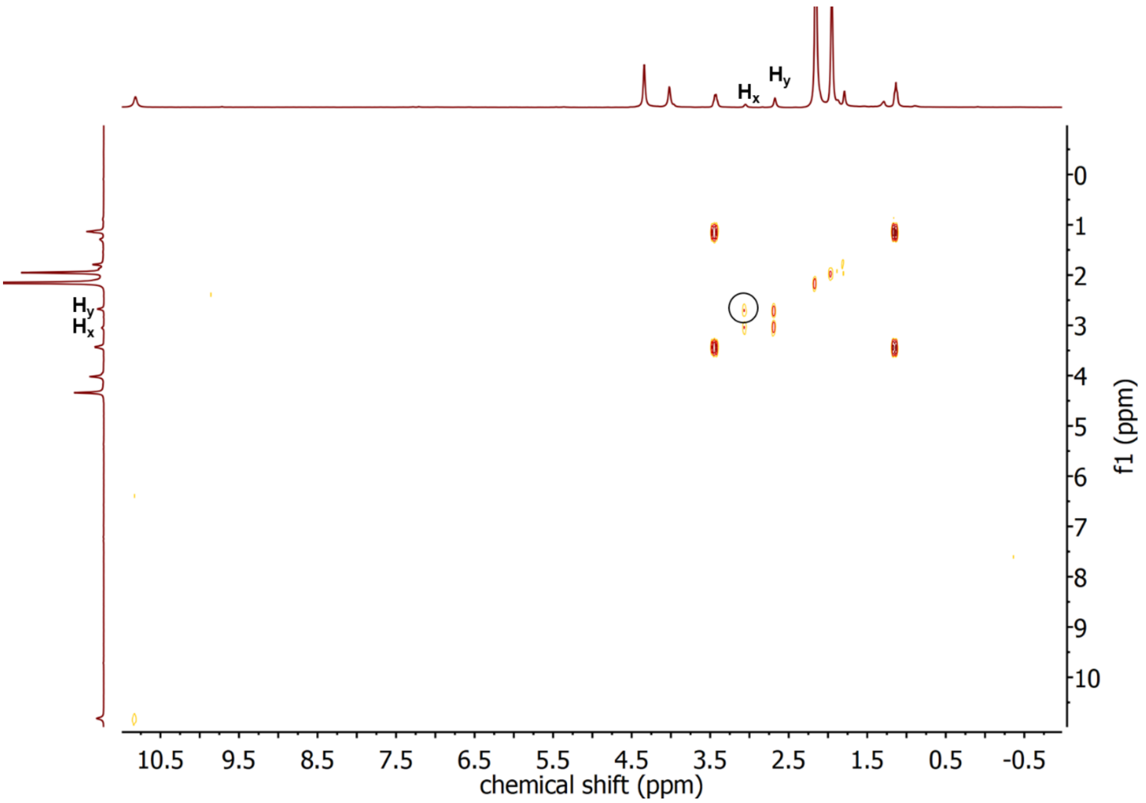

Figure S40 ^{1}H-^{1}H COSY spectrum of adamantane⊂**1** in the diamagnetic region (CD_3CN, 400 MHz, 298 K). The black circle marks the cross peak between H_x and H_y.

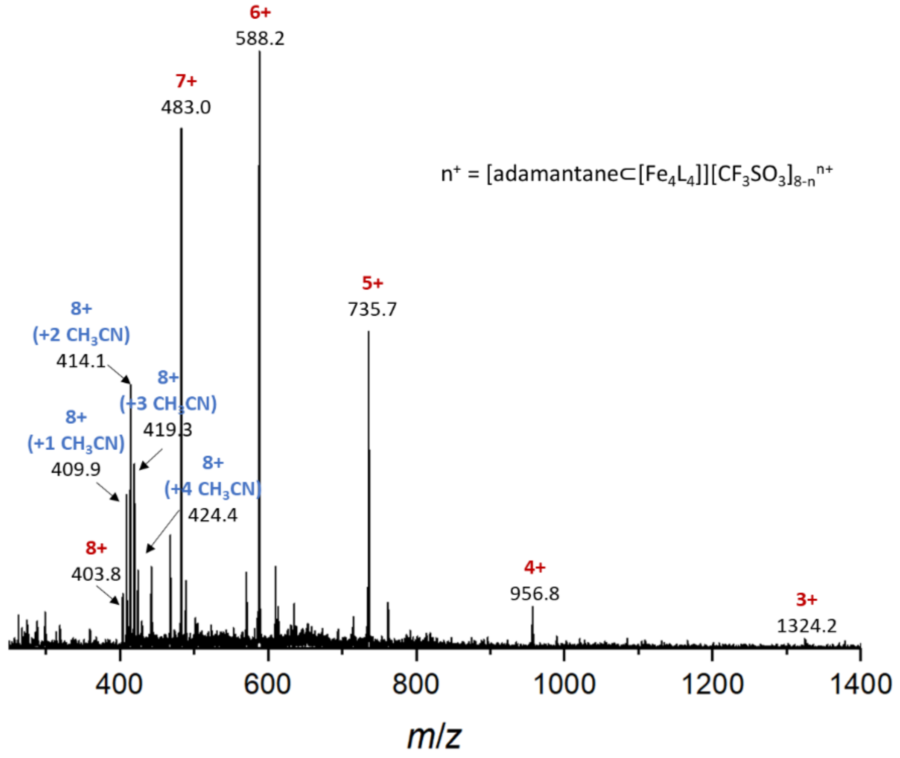

Figure S41 LRMS (ESI$^+$-Q, CH_3CN) of adamantane⊂**1**.

4.2 1-Adamantanol⊂**1**

^{1}H NMR (400 MHz, 298 K, CD_3CN): δ 57.70 (H_a), 32.40 (H_d), 31.76 (H_c), 11.40 (H_f), 4.48 (H_g), 4.00 (H_b), 3.40 (encapsulated 1-adamantanol, H_x), 2.60−2.70 (encapsulated 1-adamantanol, H_y, H_z,) ppm. (signal of H_e was not observed, which might lie under a solvent signal or be too broad to be observed)

LRMS (ESI$^+$-Q, CH_3CN) [charge, calculated for $(C_{10}H_{16}O)Fe_4(C_{39}H_{39}N_{15})_4(CF_3SO_3)_8$]: *m/z* = 405.9 [1-adamantanol⊂**1**$^{8+}$ 405.9], 485.1 [1-adamantanol⊂**1**$(OTf)_1{}^{7+}$ 485.1], 590.8 [1-adamantanol⊂**1**$(OTf)_2{}^{6+}$ 590.8], 960.7 [1-adamantanol⊂**1**$(OTf)_4{}^{4+}$ 960.8]

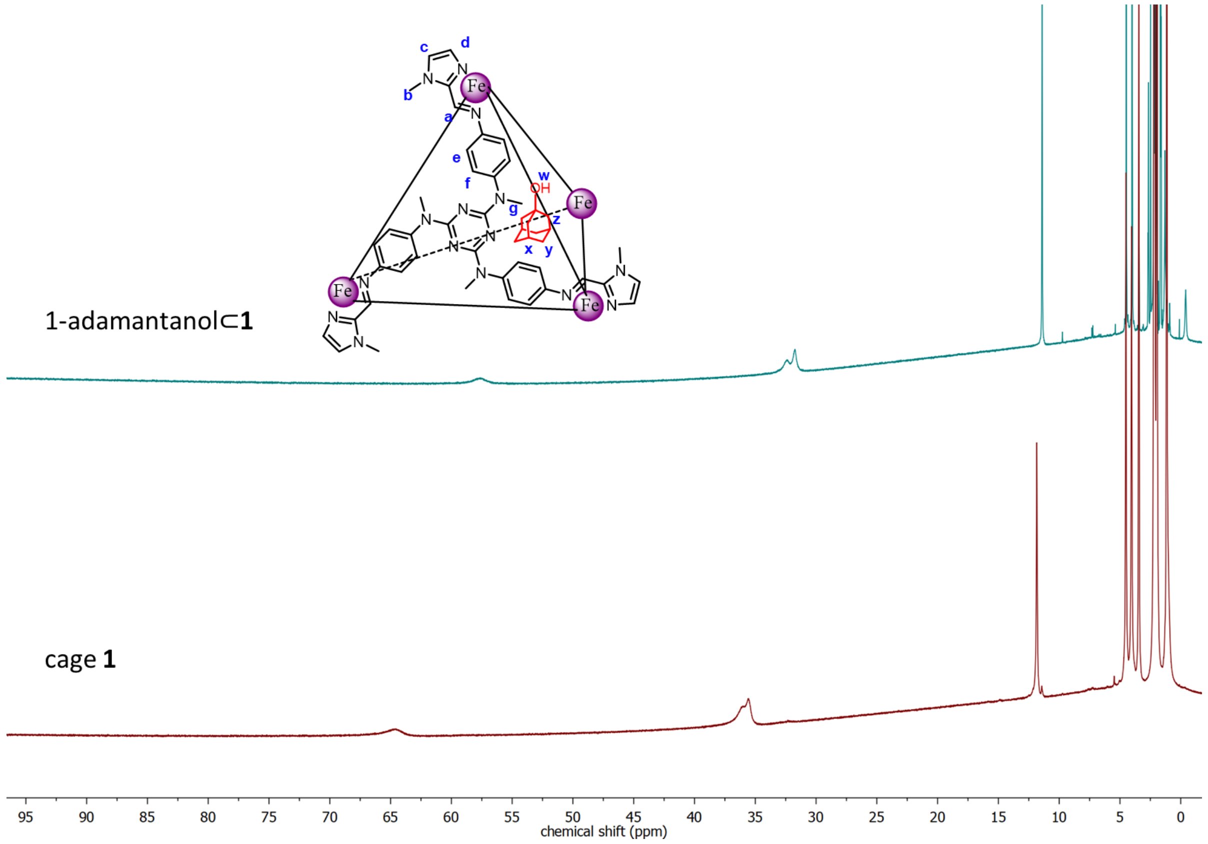

Figure S42 ^{1}H NMR spectrum of 1-adamantanol⊂**1** (CD_3CN, 400 MHz, 298 K).

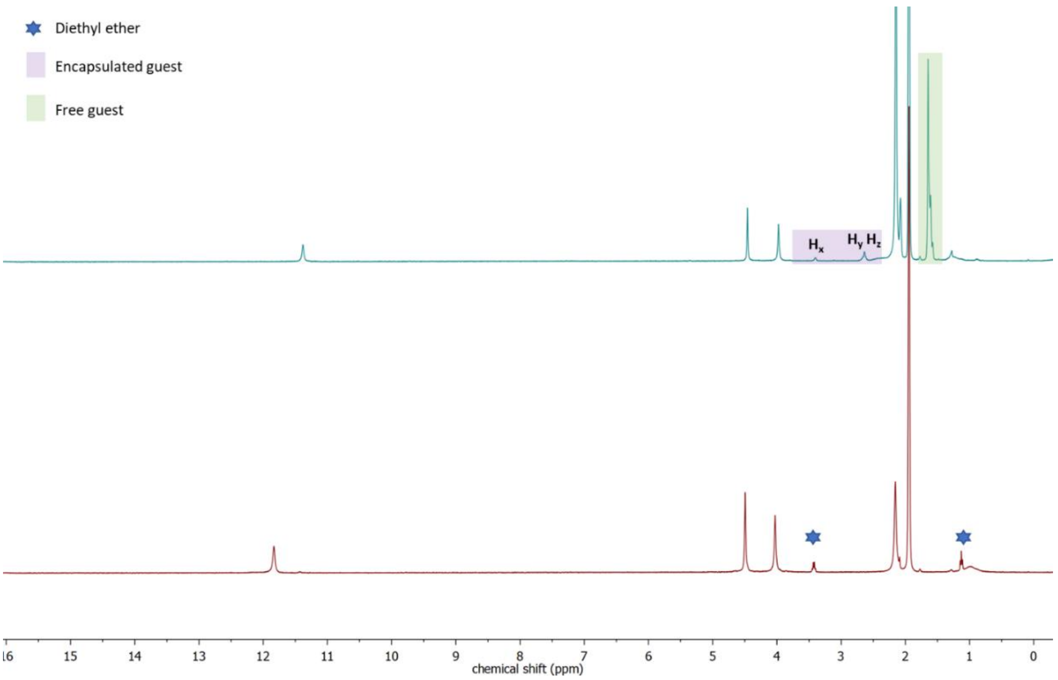

Figure S43 ^{1}H NMR spectra (CD_3CN, 400 MHz, 298 K) of 1-adamantanol⊂**1** (top) and tetrahedron **1** (bottom) in the diamagnetic region.

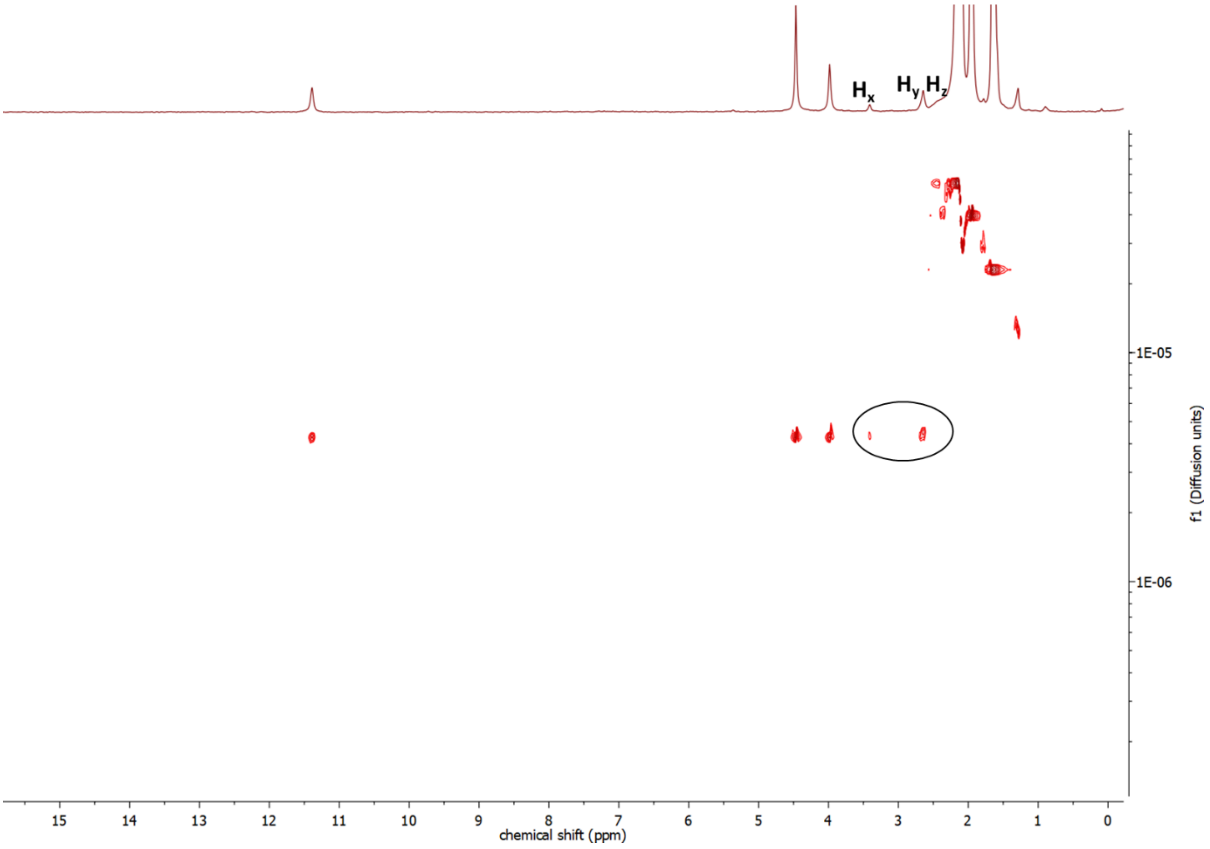

Figure S44 ^{1}H DOSY spectrum of 1-adamantanol⊂**1** in the diamagnetic region (CD_3CN, 400 MHz, 298 K). The black circle shows that proton signals for encapsulated 1-adamantanol (H_x, H_y, H_z) diffuse at the same rate as the cage's protons.

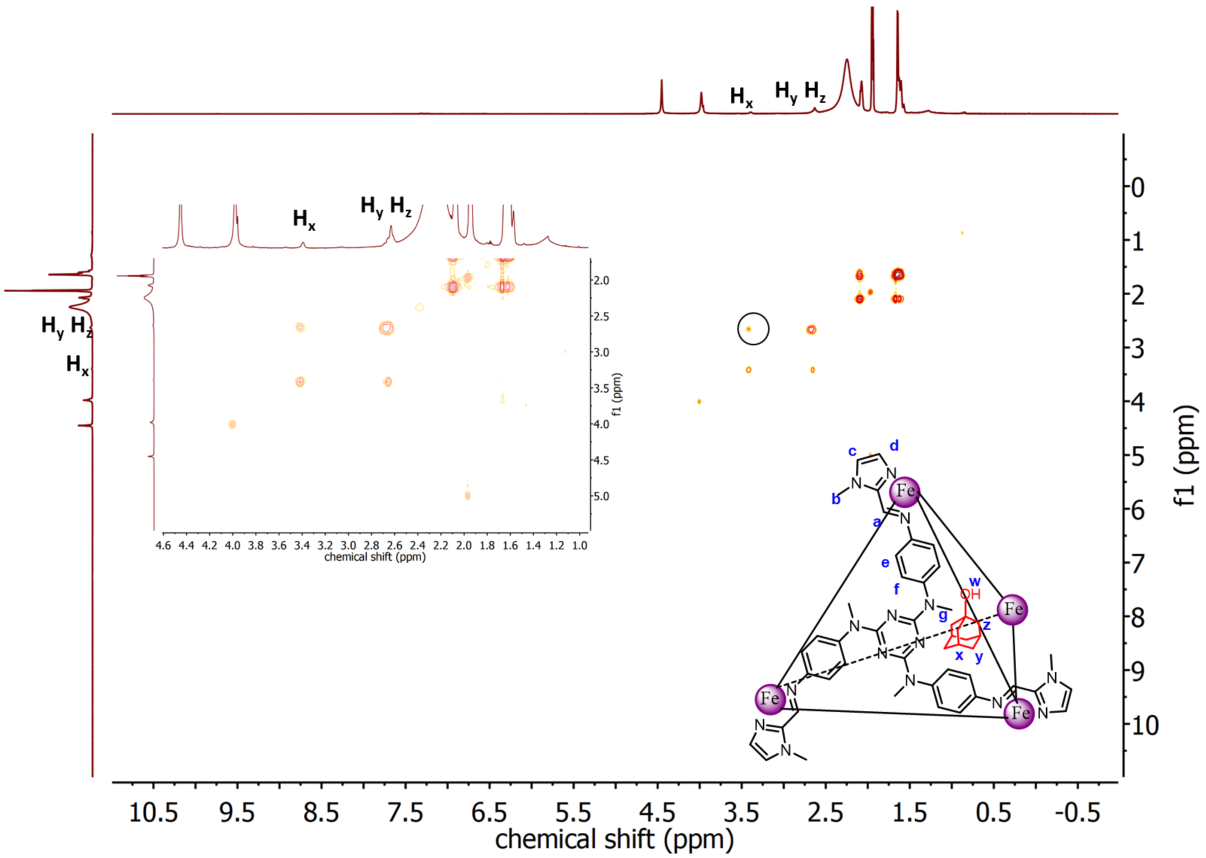

Figure S45 ^{1}H-^{1}H COSY spectrum of 1-adamantanol⊂**1** in the diamagnetic region with insert showing the signals for the encapsulated guest (CD_3CN, 400 MHz, 298 K). The black circle marks the cross peak between H_x with H_y and H_z.

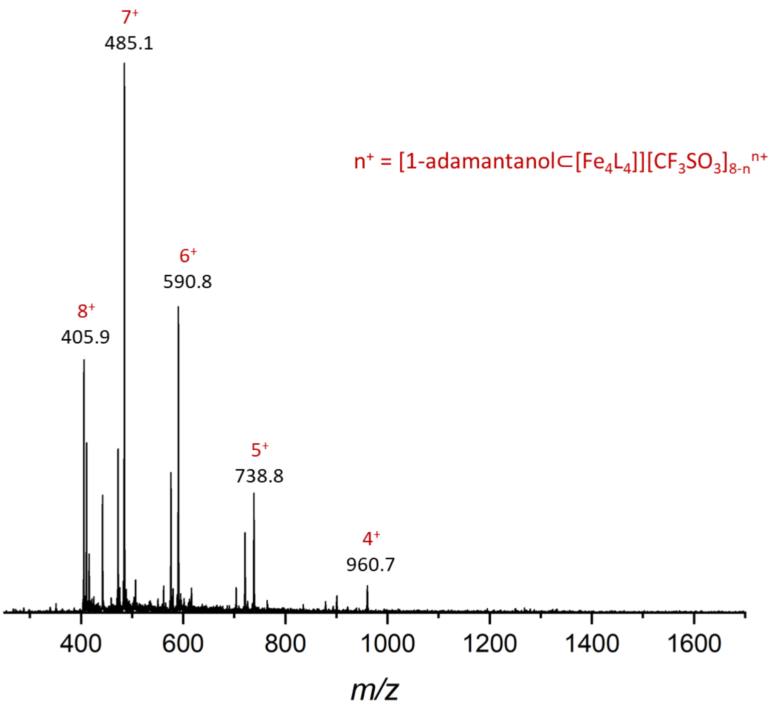

Figure S46 LRMS (ESI$^+$-Q, CH_3CN) of 1-adamantanol⊂**1**.

4.3 Cyclohexane⊂**1**

^{1}H NMR (400 MHz, 298 K, CD_3CN): δ 57.68 (H_a), 32.16 (H_d), 31.65 (H_c), 11.27 (H_f), 4.43 (H_g), 4.04 (H_b), 2.38 (encapsulated cyclohexane) ppm. (signal of H_e was not observed, which might lie under a solvent signal or was too broad to be observed)

LRMS (ESI$^+$-Q, CH_3CN) [charge, calculated for $(C_6H_{12})Fe_4(C_{39}H_{39}N_{15})_4(CF_3SO_3)_8$]: *m/z* = 402.4 [cyclohexane⊂**1**$^{8+}$ 410.1]

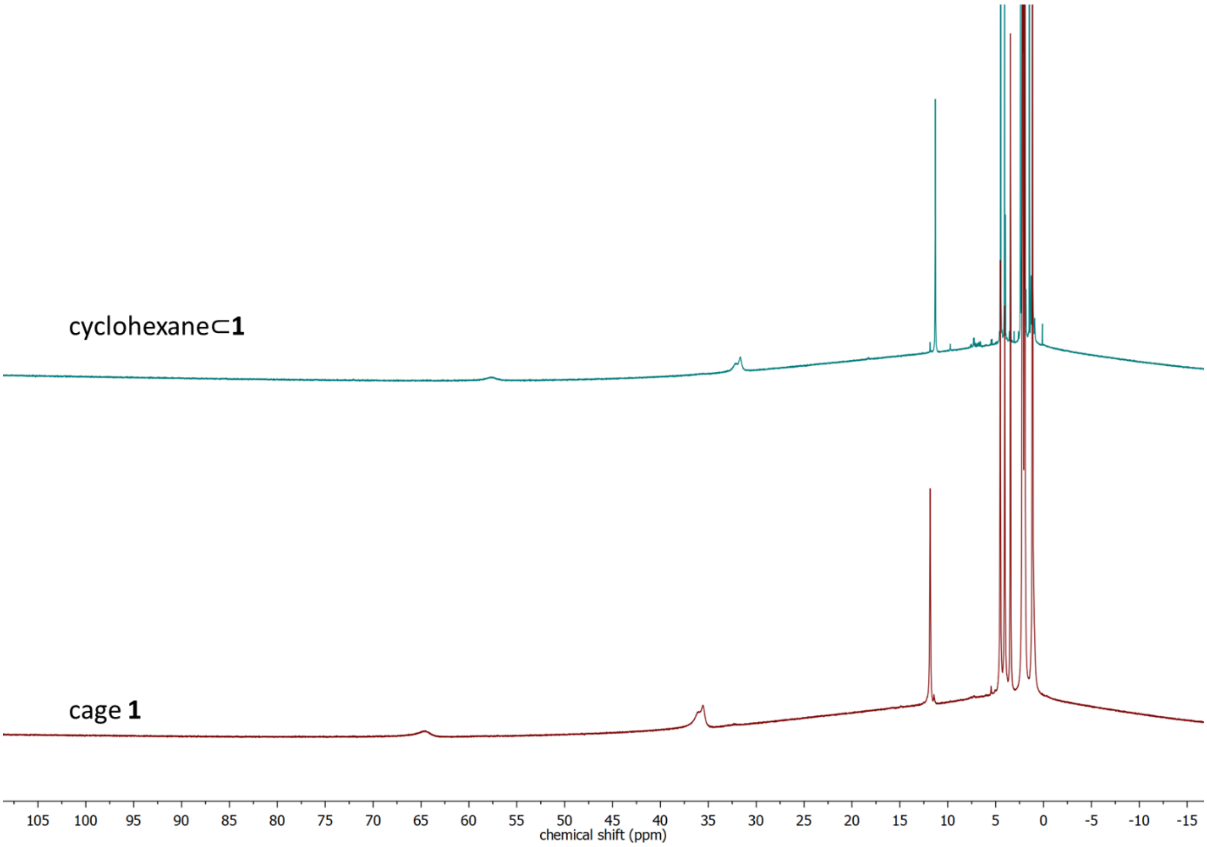

Figure S47 ^{1}H NMR spectrum of cyclohexane⊂**1** (CD_3CN, 400 MHz, 298 K).

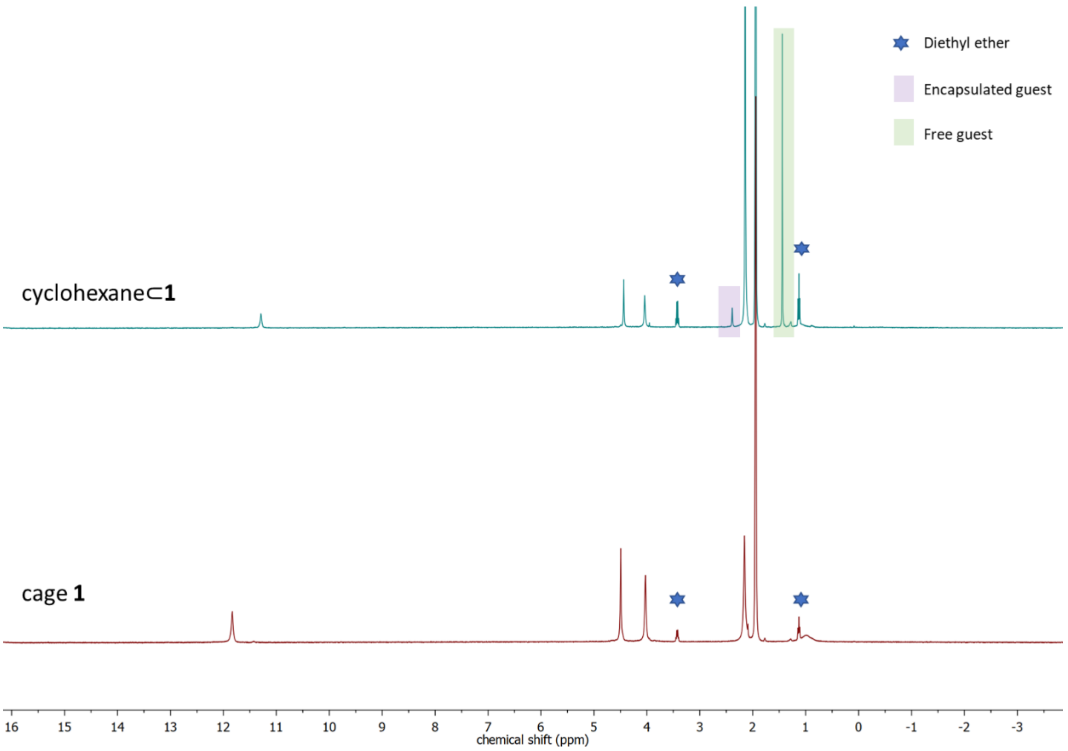

Figure S48 ^{1}H NMR spectrum of cyclohexane⊂**1** in the diamagnetic region (CD_3CN, 400 MHz, 298 K).

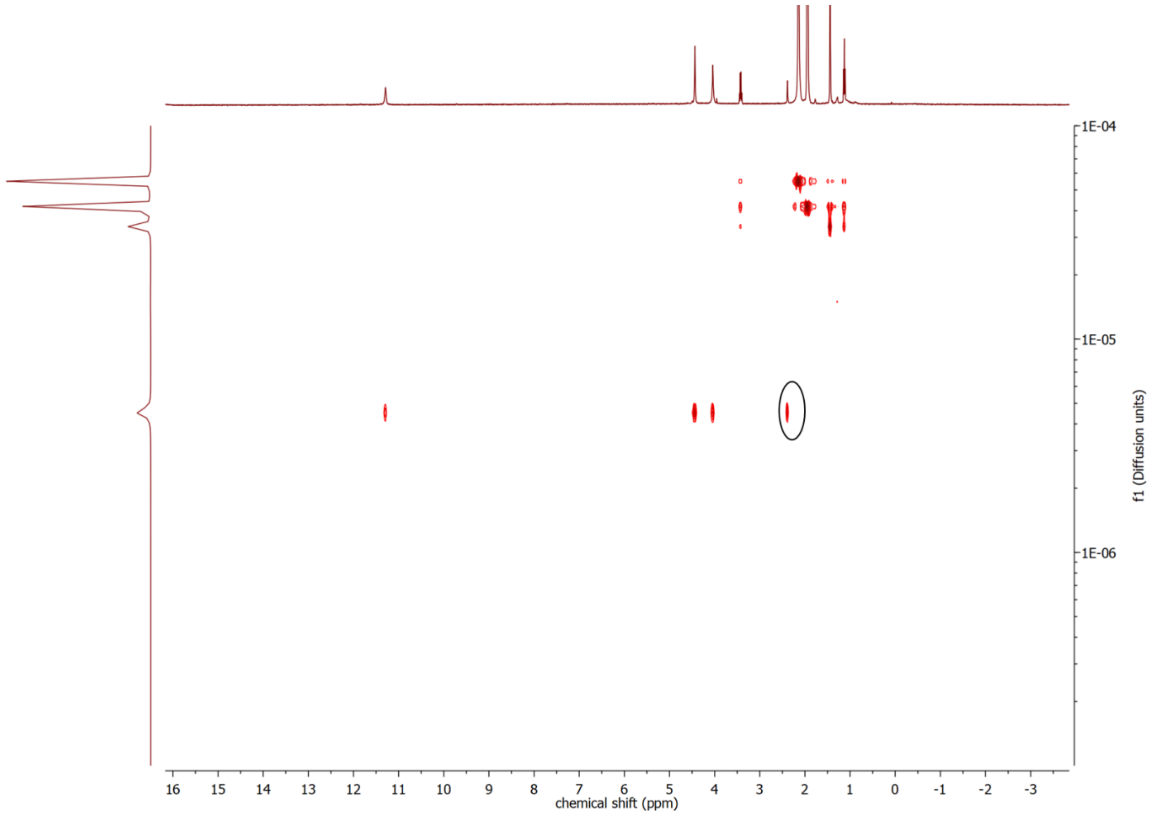

Figure S49 ^{1}H DOSY spectrum of cyclohexane⊂**1** in the diamagnetic region (CD_3CN, 400 MHz, 298 K). The black circle shows that the proton signal for encapsulated cyclohexane diffuses at the same rate as the cage's protons.

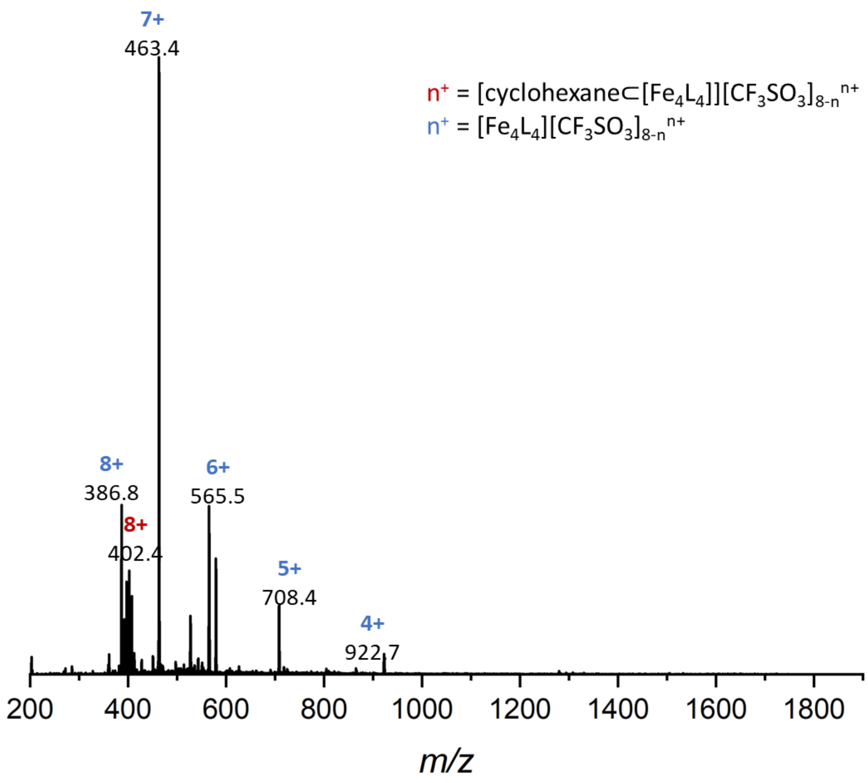

Figure S50 LRMS (ESI$^+$-Q, CH_3CN) of cyclohexane⊂**1**.

4.4 *cis*-Decalin⊂**1**

^{1}H NMR (500 MHz, 298 K, CD_3CN): δ 73.30 (H_a), 40.77 (H_d and H_c), 12.22 (H_f), 4.39 (H_b), 4.14 (H_e) ppm. (signals of H_d and H_c overlap; the signal of H_e was not observed, which might lie underneath a solvent signal or was too broad to be observed; signals corresponding to encapsulated guests overlap with solvent signals)

LRMS (ESI^+-Q, CH_3CN) [charge, calculated for $(C_{10}H_{18})Fe_4(C_{39}H_{39}N_{15})_4(CF_3SO_3)_8$]: *m/z* = 483.4 [*cis*-decalin⊂**1**$(OTf)_1^{7+}$ 483.1], 588.7 [*cis*-decalin⊂**1**$(OTf)_2^{6+}$ 588.5], 736.2 [*cis*-decalin⊂**1**$(OTf)_3^{5+}$ 736.0], 957.5 [*cis*-decalin⊂**1**$(OTf)_4^{4+}$ 957.3]

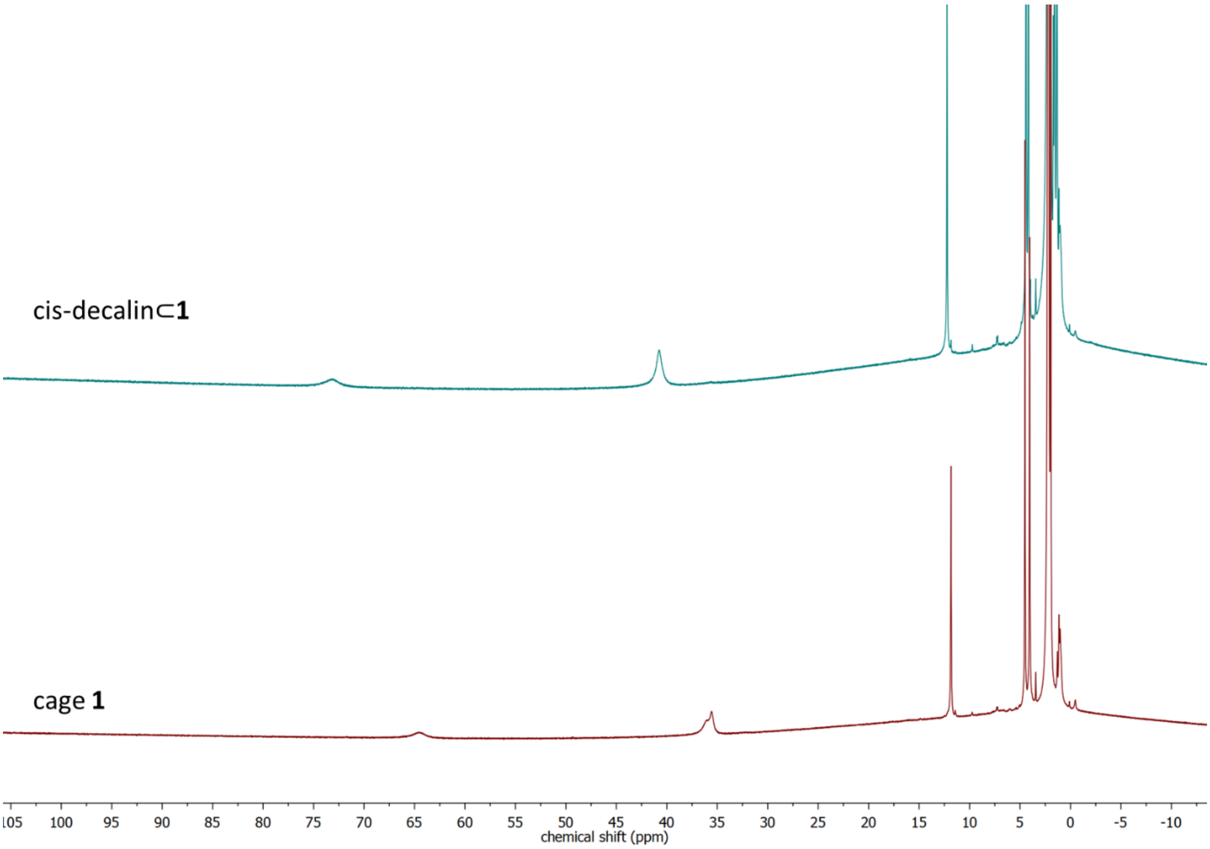

Figure S51 ^{1}H NMR spectrum of *cis*-decalin⊂**1** (CD_3CN, 500 MHz, 298 K).

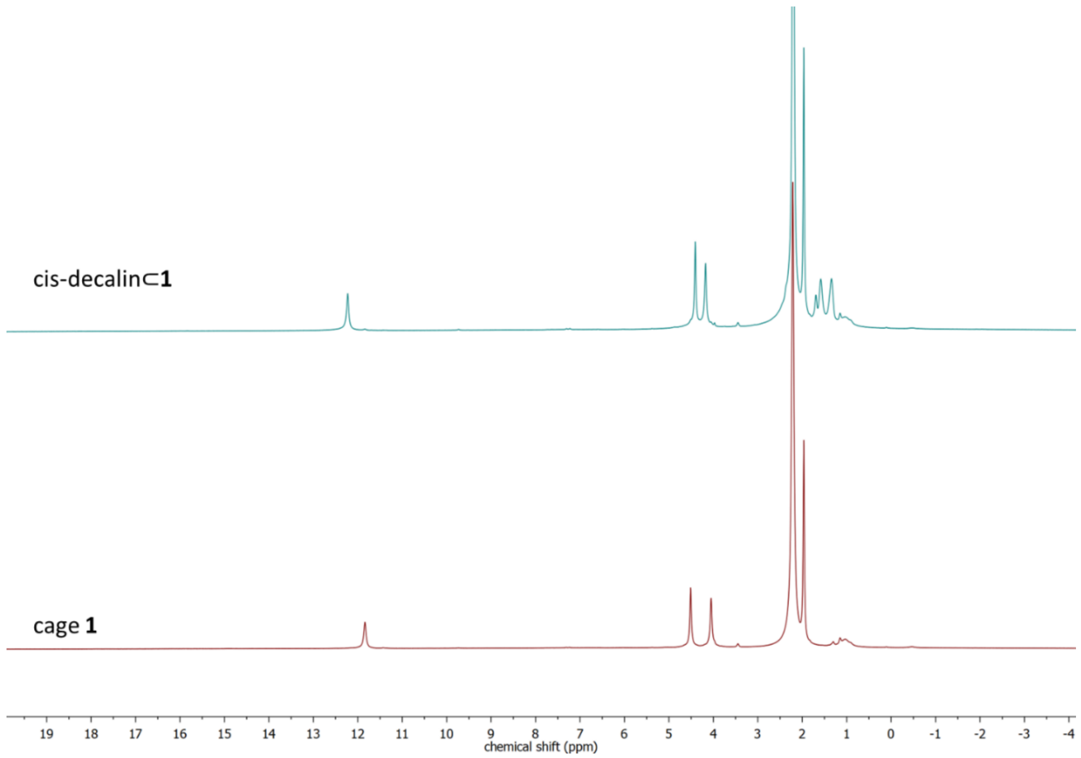

Figure S52 ^{1}H NMR spectrum of *cis*-decalin⊂**1** at diamagnetic region (CD_3CN, 500 MHz, 298 K).

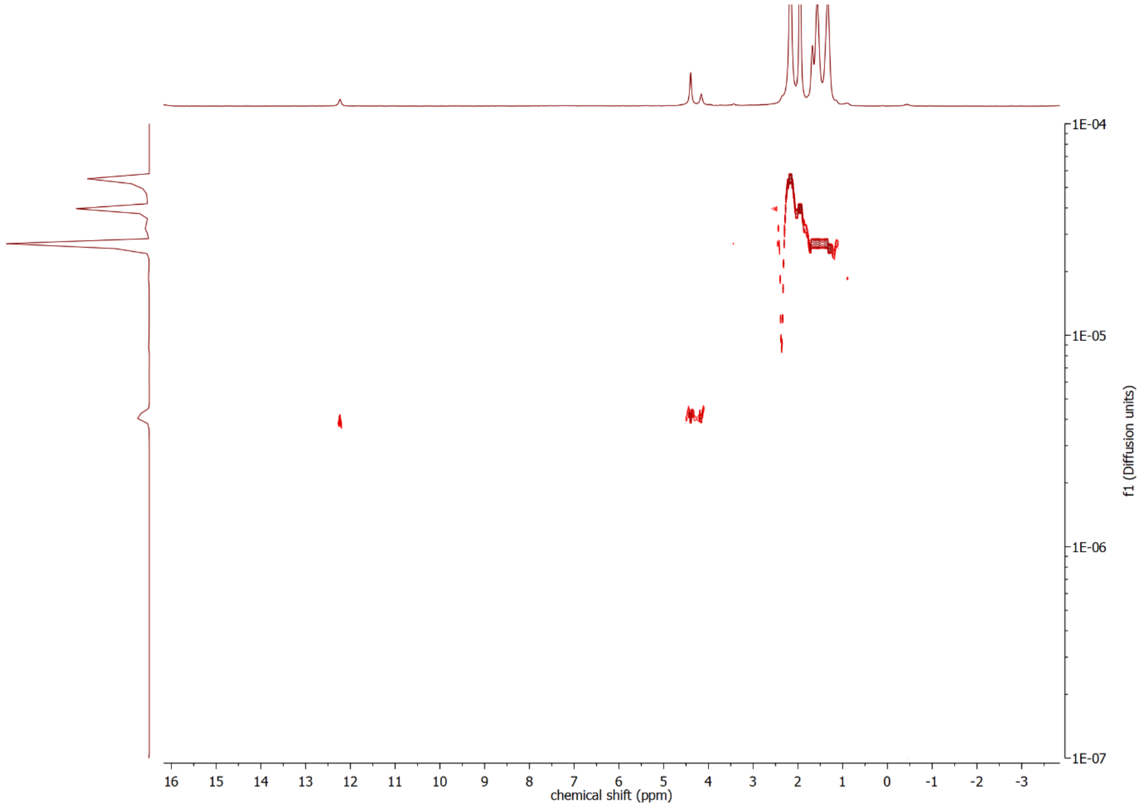

Figure S53 ^{1}H DOSY spectrum of *cis*-decalin⊂**1** in the diamagnetic region (CD_3CN, 400 MHz, 298 K).

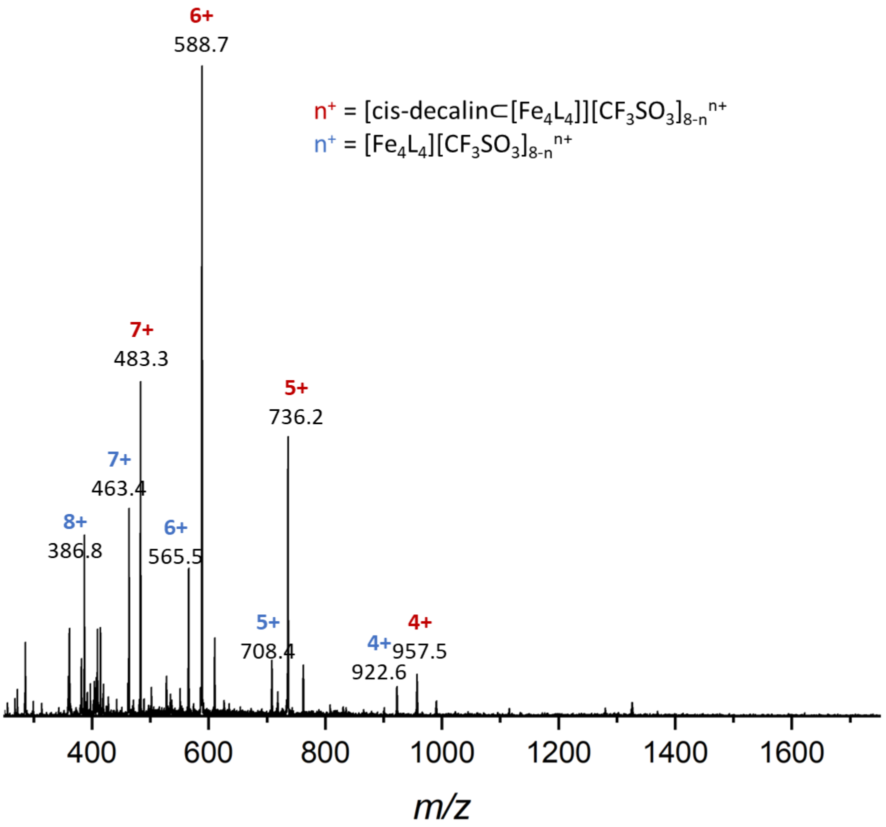

Figure S54 LRMS (ESI$^+$-Q, CH_3CN) of *cis*-decalin⊂**1**.

4.5 Adamantane⊂**2**

^{1}H NMR (400 MHz, 298 K, CD_3CN): δ 141.395, 31.521, 13.339, 12.051, 6.205, 4.856, 2.941, −1.917, −2.353, −8.218 ppm.

LR-ESI-MS [charge, calculated for $(C_{10}H_{16})Fe_4(C_{51}H_{45}N_{15})_4(CF_3SO_3)_8$]: m/z = 478.8 [adamantane⊂**2**$^{8+}$ 478.9], 568.6 [adamantane⊂**2**$(OTf)_1^{7+}$ 568.7], 688.3 [adamantane⊂**2**$(OTf)_2^{6+}$ 688.3], 855.7 [adamantane⊂**2**$(OTf)_3^{5+}$ 855.8], 1107.0 [adamantane⊂**2**$(OTf)_4^{4+}$ 1107.0]

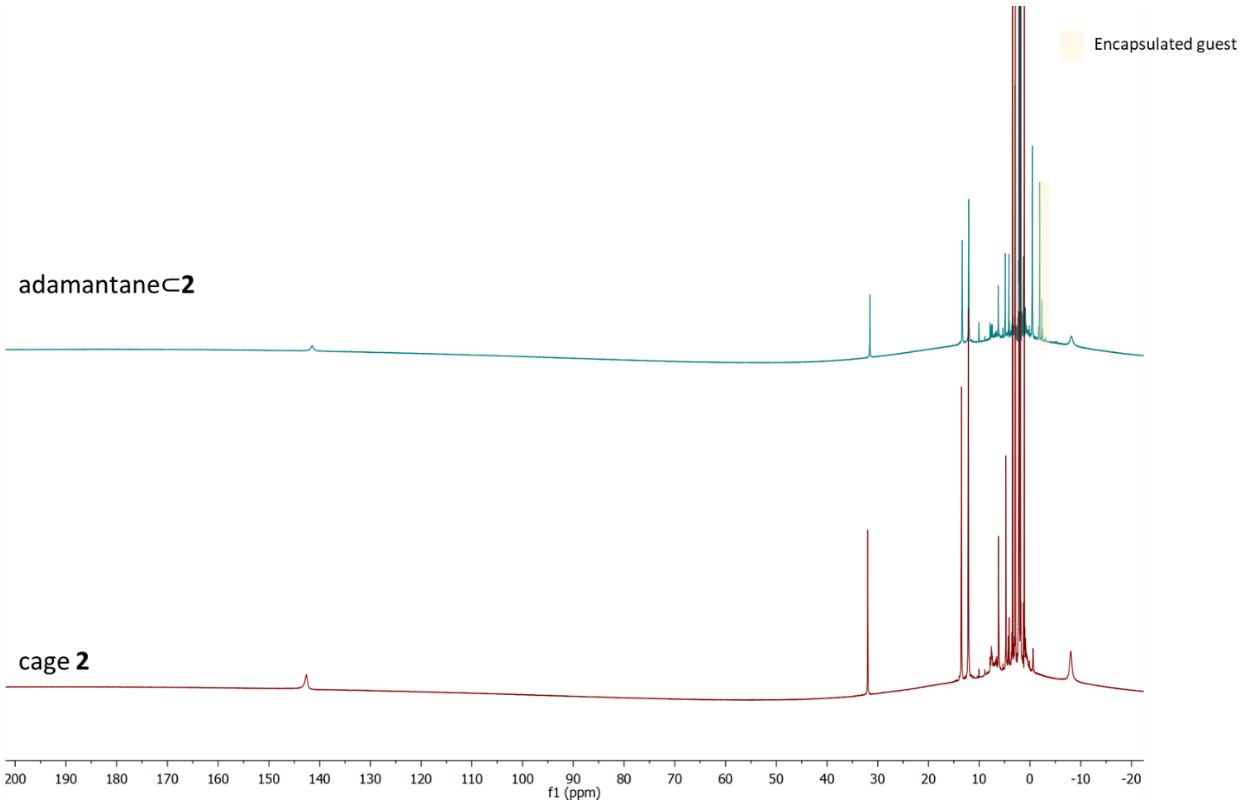

Figure S55 ^{1}H NMR spectrum of adamantane⊂**2** (CD_3CN, 400 MHz, 298 K).

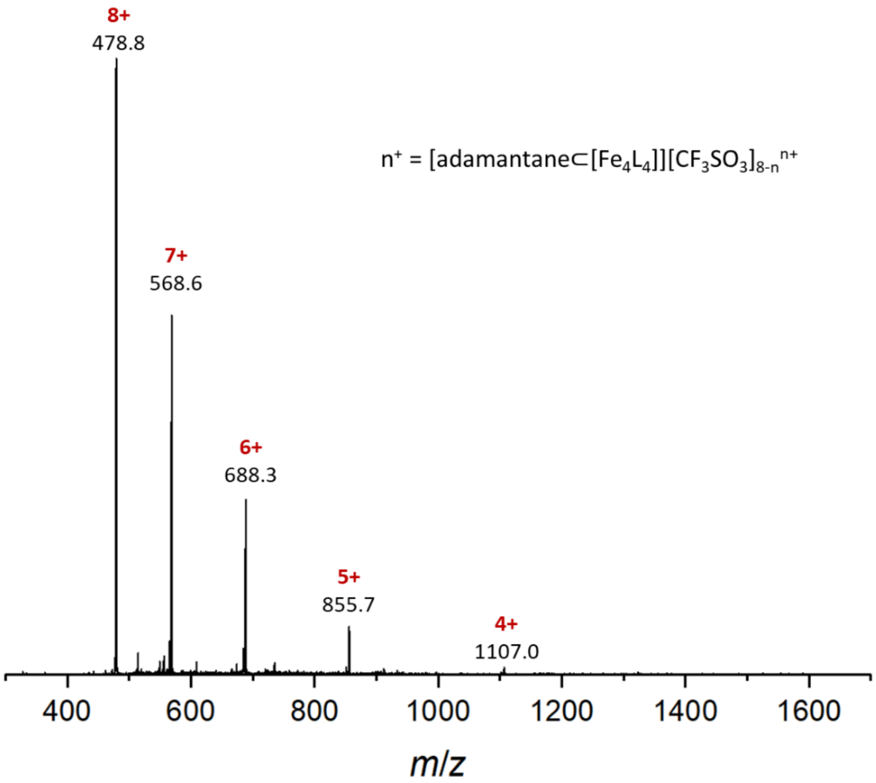

Figure S56 LRMS (ESI$^+$-Q, CH_3CN) of adamantane⊂**2**.

4.6 Adamantane⊂**3**

^{1}H NMR (400 MHz, 298 K, CD_3CN): δ 9.77, 8.86, 7.44 (d, *J* = 8.4 Hz), 7.39, 5.00 (d, *J* = 7.4 Hz), 3.43, 1.64 ppm.

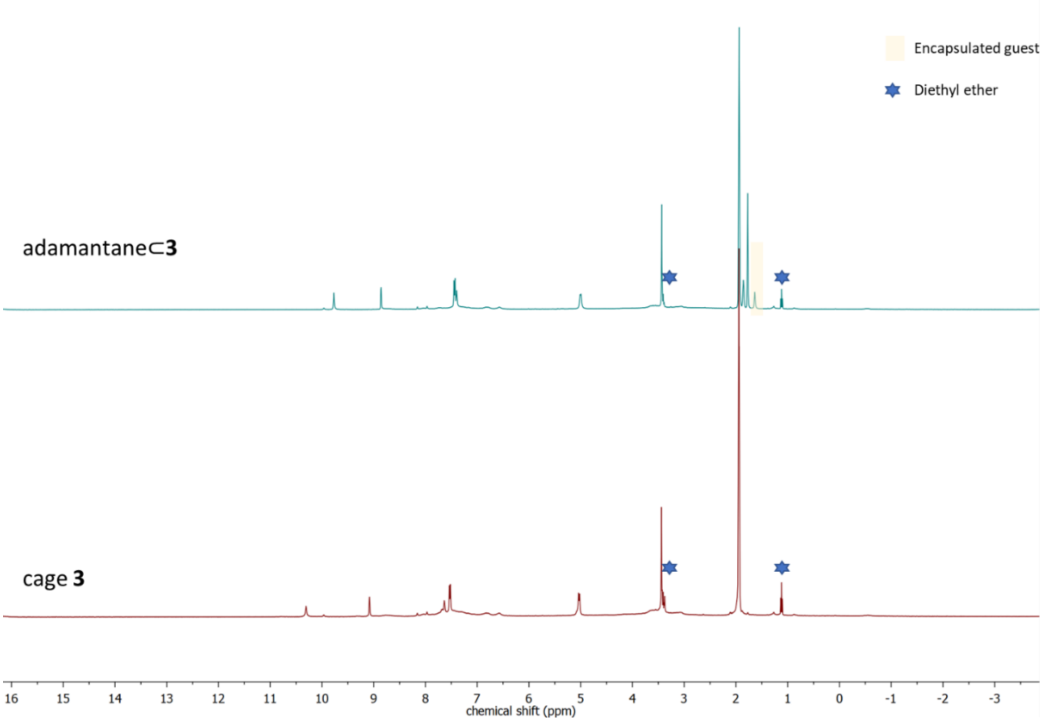

Figure S57 ^{1}H NMR spectrum of adamantane⊂**3** (CD_3CN, 400 MHz, 298 K).

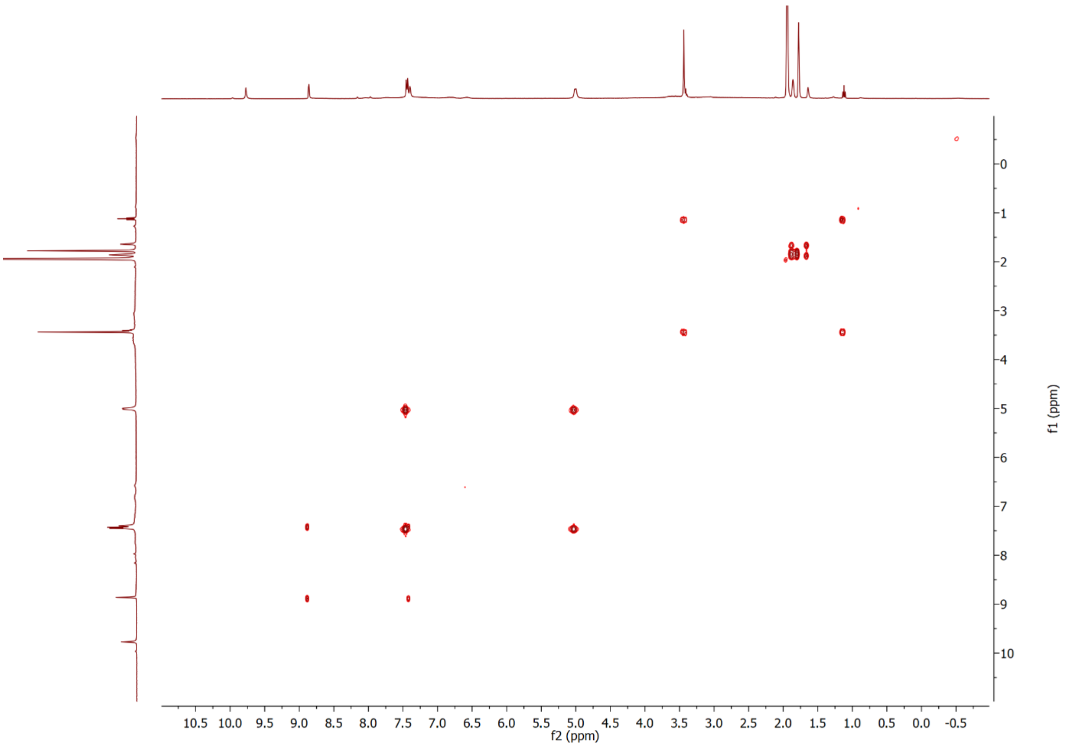

Figure S58 ^{1}H-^{1}H COSY spectrum of adamantane⊂**3** (CD_3CN, 400 MHz, 298 K).

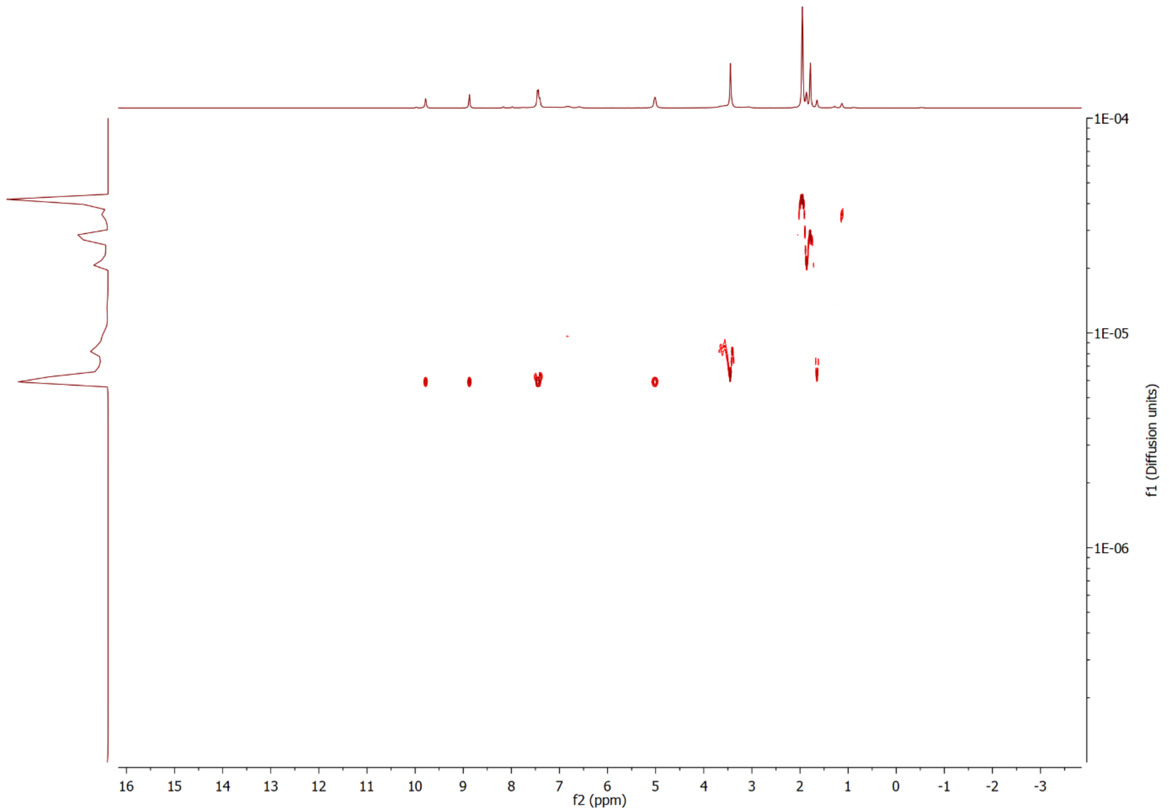

Figure S59 ^{1}H DOSY spectrum of adamantane⊂**3** (CD_3CN, 400 MHz, 298 K). Proton signals for encapsulated adamantane (H_x, H_y) diffuse at the same rate as the cage's protons.

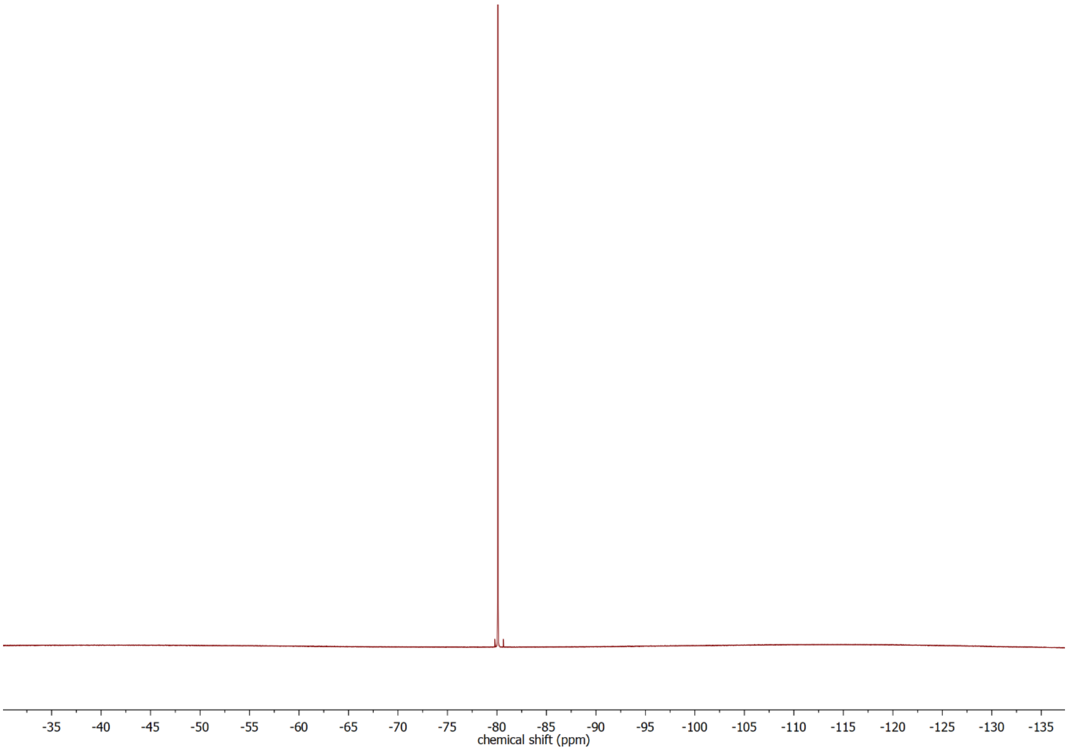

Figure S60 ^{19}F NMR spectrum of adamantane⊂**3** (CD_3CN, 376 MHz, 298 K) showing no encapsulated NTf_2^- signal (compare with Figure **S17**).

4.7 Comparison of guest chemical shifts in host-guest complexes of tetrahedron **1**

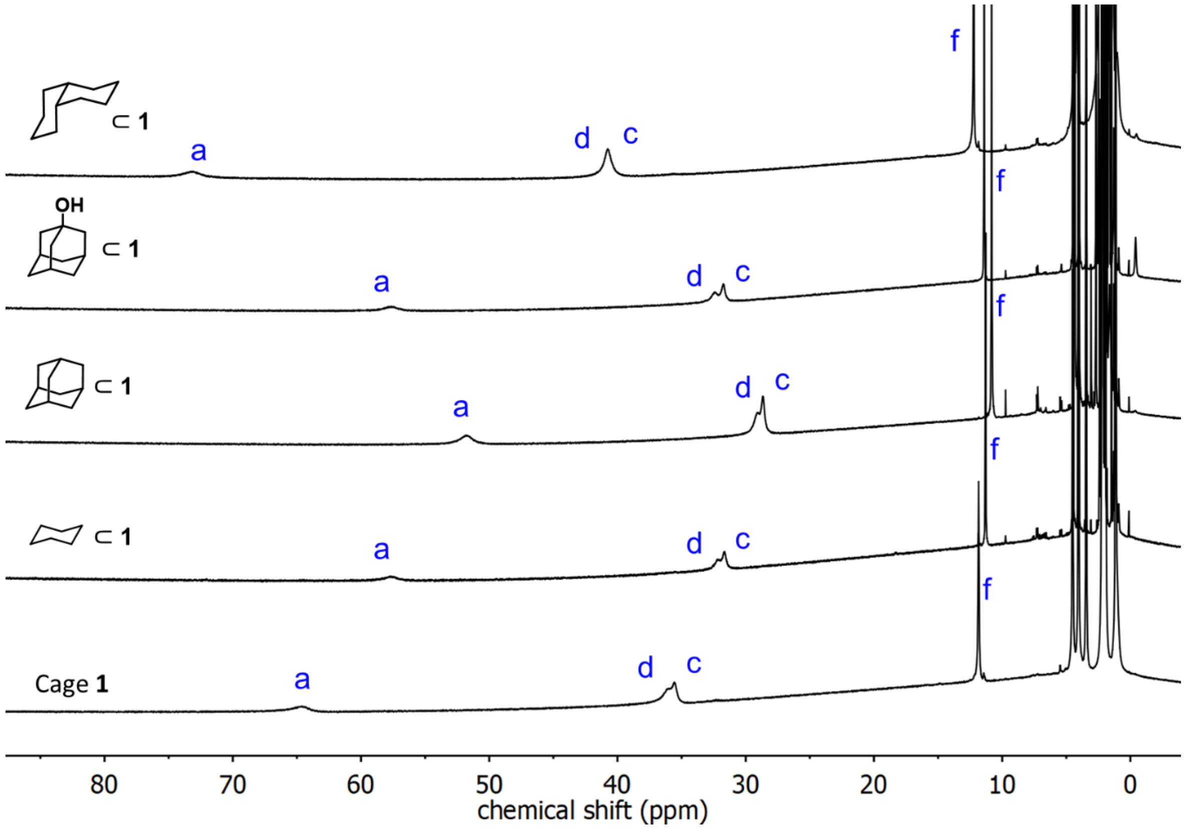

Figure S61^{1}H NMR (400 MHz, 298 K, CD_3CN) of tetrahedron **1** and its host-guest complexes with cyclohexane, adamantane, 1-adamantanol, and *cis*-decalin (top to bottom). Note that guests which stabilize LS tetrahedron **1**, i.e. cyclohexane, adamantane, and 1-adamantanol, show a upfield shift of cage proton H_a compared to empty tetrahedron **1**, while larger *cis*-decalin, which stabilized the HS state, induces a downfield shift in H_a.

4.8 Zn(II) analogue of tetrahedron **1** and its host-guest compounds

The synthetic procedure for a Zn(II) analogue of tetrahedron **1** (Zn**1**) is similar to that of tetrahedron **1**. Triamine **D** (8.00 mg, 18.0 µmol, 4 equiv.), zinc(II) triflate (6.58 mg, 18.0 µmol, 4 equiv.), and aldehyde **A** (5.97 mg, 54.0 µmol, 12 equiv.) were suspended in MeCN (4 mL). The reaction mixture was stirred overnight at room temperature. After washing with diethyl ether three times, the amorphous product was used for characterization directly (yield: 15.2 mg, 78%). 5-20 equivalents of the guest were added to the cage solution in CD_3CN (approx. 0.5 mᴍ, 0.4-0.5 mL). The solution was transferred to an NMR tube, then left to equilibrate at 298 K for 15 min.

^{1}H NMR Zn**1** (500 MHz, 298 K, CD_3CN): δ 8.37, 7.58, 7.48 (d, *J* = 8.7 Hz), 7.01, 5.73 (d, *J* = 8.7 Hz), 4.03, 3.42 ppm.

^{1}H NMR adamantane⊂Zn**1** (500 MHz, 298 K, CD_3CN): δ 8.35, 7.59, 7.42 (d, *J* = 8.8 Hz), 7.05, 5.51 (d, *J* = 8.7 Hz), 4.03, 3.45, 1.80−1.70 (encapsulated adamantane) ppm.

^{1}H NMR adamantanol⊂Zn**1** (500 MHz, 298 K, CD_3CN): δ 8.34, 7.59, 7.49 (d, *J* = 8.7 Hz), 7.04, 5.56 (d, *J* = 8.8 Hz), 4.03, 3.45, 1.85−1.42 (encapsulated 1-adamantanol) ppm.

^{1}H NMR cyclohexane⊂Zn**1** (500 MHz, 298 K, CD_3CN): δ 8.36, 7.59, 7.45 (d, *J* = 8.7 Hz), 7.04, 5.54 (d, *J* = 8.7 Hz), 4.03, 3.46, 1.34 (encapsulated cyclohexane) ppm.

^{1}H NMR *cis*-decalin⊂Zn**1** (500 MHz, 298 K, CD_3CN): δ 8.39, 7.58, 7.44 (d, *J* = 8.6 Hz), 6.98, 5.90 (d, *J* = 8.6 Hz), 4.02, 3.39 1.08−0.83 (encapsulated *cis*-decalin) ppm.

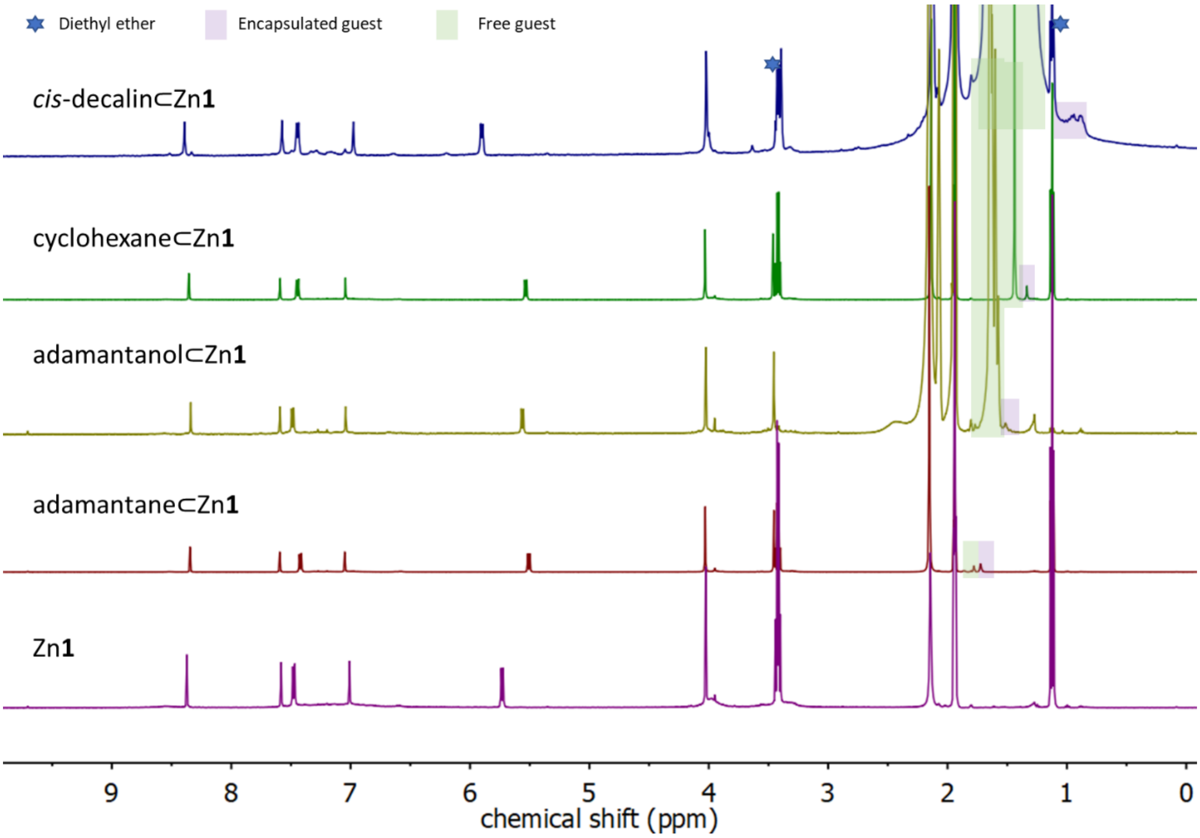

Figure S62 ^{1}H NMR spectra (CD_3CN, 500 M, 298 K) of Zn**1**, adamantane⊂Zn**1**, 1-adamantanol⊂Zn**1**, cyclohexane⊂Zn**1**, and *cis*-decalin⊂Zn**1** (bottom to top).

4.9 Guest binding affinity depending on the spin states of tetrahedron **1**

As cage **1** is a SCO system and exists in mixed spin states at room temperature, determining the association constants of different guests to tetrahedron **1** is not straightforward. The following sections demonstrate, how we developed a simple approximation to derive the association constants for the two extremes of the SCO process, i.e. all high-spin or all low-spin metal centers, from a simple 1:1 isotherm. Association constants were determined by NMR titrations as detailed below (Section 4.9.2).

4.9.1 Deriving individual association constants for both spin states of tetrahedron **1**

A simple host-guest equilibrium between a guest **G** and a host **H** can be described by Scheme S5.

The association constant K_{obs} is defined as

$$K_{obs} = \frac{[\mathbf{G}\subset\mathbf{H}]}{[\mathbf{G}][\mathbf{H}]} \,. \qquad \text{(S3.)}$$

If the metal centers of the cage, which acts as the host here, can reside in different spin states at a given temperature, the simple equilibrium of Scheme S5 leads to the thermodynamic cycle depicted in Scheme S6 (also Figure 4a in the main text) under the simplifying assumption, that all spin centers are either high-spin (**HS**) or low-spin (**LS**) and no mixtures of spin states exist within one structure.

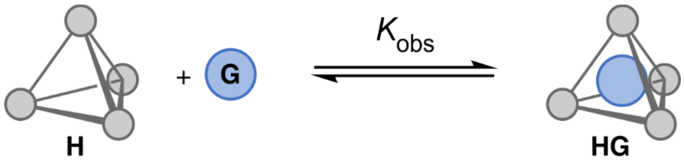

Scheme S5 A simple 1:1 host-guest association.

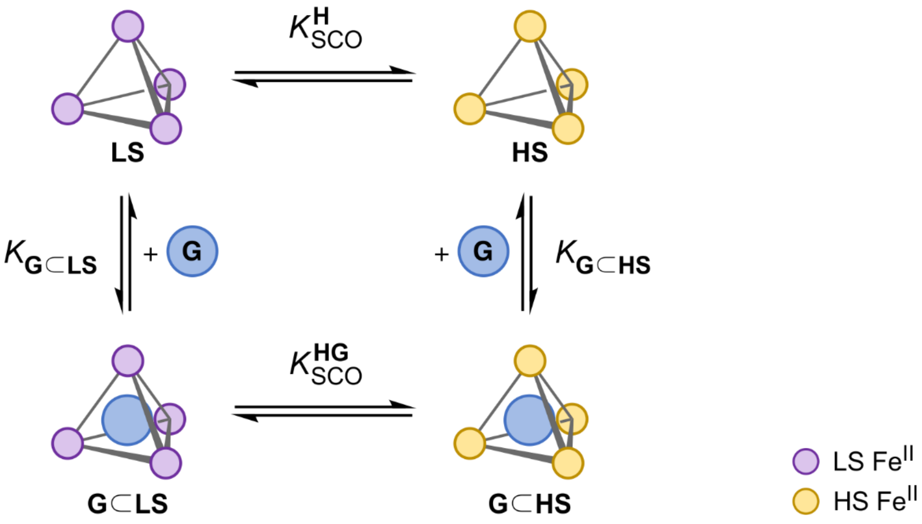

Scheme S6 Thermodynamic cycle for the association of a guest **G** to a host **H** to form a host-guest complex **HG**. **H** and **HG** can reside in either an all low-spin state (**LS**, purple) or an all high-spin state (**HS**, yellow) and show SCO between those two states with the equilibrium constants $K_{SCO}^{\mathbf{H}}$ and $K_{SCO}^{\mathbf{HG}}$, respectively. Since the cavity size of the cage is affected by the spin state of the metal centres, the different spin states are expected to show different affinities to any given guest, $K_{\mathbf{G}\subset\mathbf{HS}}$ and $K_{\mathbf{G}\subset\mathbf{LS}}$, respectively.

The respective equilibrium constants are defined as follows:

$$K_{\text{SCO}}^{\mathbf{H}} = \frac{[\mathbf{HS}]}{[\mathbf{LS}]} \quad \text{(S4.)}$$

$$K_{\text{SCO}}^{\mathbf{HG}} = \frac{[\mathbf{G{\subset}HS}]}{[\mathbf{G{\subset}LS}]} \quad \text{(S5.)}$$

$$K_{\mathbf{G{\subset}HS}} = \frac{[\mathbf{G{\subset}HS}]}{[\mathbf{G}][\mathbf{HS}]} \quad \text{(S6.)}$$

$$K_{\mathbf{G{\subset}LS}} = \frac{[\mathbf{G{\subset}LS}]}{[\mathbf{G}][\mathbf{LS}]} \quad \text{(S7.)}$$

As for any thermodynamic cycle

$$\frac{K_{\text{SCO}}^{\mathbf{H}}\, K_{\mathbf{G{\subset}HS}}}{K_{\text{SCO}}^{\mathbf{HG}}\, K_{\mathbf{G{\subset}LS}}} = 1 \quad \text{(S8.)}$$

and, hence,

$$\Delta G_{\text{SCO}}^{\mathbf{H}} + \Delta G_{\mathbf{G{\subset}HS}} - \Delta G_{\text{SCO}}^{\mathbf{HG}} - \Delta G_{\mathbf{G{\subset}LS}} = 0 \quad \text{(S9.)}$$

hold true, it follows

$$\therefore\ \Delta G_{\mathbf{G{\subset}HS}} - \Delta G_{\mathbf{G{\subset}LS}} = \Delta G_{\text{SCO}}^{\mathbf{HG}} - \Delta G_{\text{SCO}}^{\mathbf{H}} \quad \text{(S10.)}$$

or

$$\frac{K_{\mathbf{G{\subset}HS}}}{K_{\mathbf{G{\subset}LS}}} = \frac{K_{\text{SCO}}^{\mathbf{HG}}}{K_{\text{SCO}}^{\mathbf{H}}}, \quad \text{(S11.)}$$

which shows that the ratio of association constants between the high-spin and low-spin states of the host, **HS** and **LS**, respectively, are directly related to the spin-crossover behavior of host **H** and host-guest complex **HG**. The ratio of the association constants of the different host spin states to the guest can therefore be written as

$$\therefore\ \frac{K_{\mathbf{G{\subset}HS}}}{K_{\mathbf{G{\subset}LS}}} = e^{\left(-\frac{\Delta G_{\text{SCO}}^{\mathbf{HG}} - \Delta G_{\text{SCO}}^{\mathbf{H}}}{RT}\right)} \quad \text{(S12.)}$$

or

$$\frac{K_{\mathbf{G{\subset}HS}}}{K_{\mathbf{G{\subset}LS}}} = e^{\left(-\frac{\Delta H_{\text{SCO}}^{\mathbf{HG}} - \Delta H_{\text{SCO}}^{\mathbf{H}}}{RT} + \frac{\Delta S_{\text{SCO}}^{\mathbf{HG}} - \Delta S_{\text{SCO}}^{\mathbf{H}}}{R}\right)} \quad \text{(S13.)}$$

The thermodynamic parameters were determined by VT NMR (Section S5) and are summarized in Table S4. The resulting ratios of $K_{\mathbf{G{\subset}HS}}$ and $K_{\mathbf{G{\subset}LS}}$ are summarized in the same table.

Table S4 Thermodynamic parameters for the SCO process of tetrahedron **1** and its various host-guest complexes at T = 298 K as well as ratios of $K_{\mathbf{G\subset HS}}$ and $K_{\mathbf{G\subset LS}}$ derived from Eq. S13.

	**Δ*H*** kJ mol⁻¹	**Δ*S*** J K⁻¹ mol⁻¹	***T* Δ*S*** kJ mol⁻¹	**Δ*G*** kJ mol⁻¹	$\frac{K_{\mathbf{G\subset HS}}}{K_{\mathbf{G\subset LS}}}$	$\frac{K_{\mathbf{G\subset LS}}}{K_{\mathbf{G\subset HS}}}$
tetrahedron **1**	26.2 ± 0.2	85 ± 1	25.3 ± 0.3	0.9 ± 0.5		
cyclohexane⊂**1**	27.1 ± 0.2	86 ± 1	25.6 ± 0.3	1.4 ± 0.5	0.82 ± 0.04	1.22 ± 0.05
adamantane⊂**1**	27.1 ± 0.3	84 ± 1	25.0 ± 0.3	2.1 ± 0.6	0.61 ± 0.03	1.65 ± 0.07
1-adamantanol⊂**1**	26.4 ± 0.3	84 ± 1	25.0 ± 0.3	1.4 ± 0.6	0.82 ± 0.03	1.22 ± 0.05
cis-decalin⊂**1**	17.9 ± 0.2	59 ± 1	17.6 ± 0.3	0.3 ± 0.5	1.25 ± 0.06	0.80 ± 0.04

If Eq. S8 and Scheme S6 hold true, the ratio $K_{\mathbf{G\subset HS}}$ and $K_{\mathbf{G\subset LS}}$ of can be directly derived from the spin-state populations γ (determined in Section S5) of the host (**HS**, **LS**) or host-guest complexes (**G⊂HS**, **G⊂LS**) a given temperature.

We define the overall host concentration as

$$[\mathbf{H}] = [\mathbf{LS}] + [\mathbf{HS}] \qquad \text{(S14.)}$$

and the concentrations of the two spin states **HS**, **LS** according to the spin state populations $\gamma_{\mathbf{LS}}$ and $\gamma_{\mathbf{LS}}$ of the host as

$$[\mathbf{LS}] = \gamma_{\mathbf{LS}}\,[\mathbf{H}] \qquad \text{(S15.)}$$

$$[\mathbf{HS}] = \gamma_{\mathbf{HS}}\,[\mathbf{H}] = (1-\gamma_{\mathbf{LS}})\,[\mathbf{H}]. \qquad \text{(S16.)}$$

Similarly, we can define the overall host-guest complex concentration in solution

$$[\mathbf{HG}] = [\mathbf{G}\subset\mathbf{LS}] + [\mathbf{G}\subset\mathbf{HS}] \qquad \text{(S17.)}$$

and derive the concentrations of the complexes at different spin states (**G⊂HS**, **G⊂LS**) from the spin state populations $\gamma_{\mathbf{G\subset LS}}$ and $\gamma_{\mathbf{G\subset HS}}$ of the host-guest complexes as

$$[\mathbf{G}\subset\mathbf{LS}] = \gamma_{\mathbf{G\subset LS}}\,[\mathbf{HG}] \qquad \text{(S18.)}$$

$$[\mathbf{G}\subset\mathbf{HS}] = \gamma_{\mathbf{G\subset HS}}\,[\mathbf{HG}] = (1-\gamma_{\mathbf{G\subset LS}})\,[\mathbf{HG}]. \qquad \text{(S19.)}$$

Given the concentrations of the four host and host-guest species derived from the spin-state populations (Eqs S15, S16, S18, S19), the ratio of the host-guest association constants of the different spin states $K_{\mathbf{G\subset HS}}$ and $K_{\mathbf{G\subset LS}}$ (Eqs S6&S7) can be derived:

$$\frac{K_{\mathbf{G\subset LS}}}{K_{\mathbf{G\subset HS}}} = \frac{\frac{[\mathbf{G\subset LS}]}{[\mathbf{G}][\mathbf{LS}]}}{\frac{[\mathbf{G\subset HS}]}{[\mathbf{G}][\mathbf{HS}]}} = \frac{[\mathbf{G\subset LS}]}{[\mathbf{G\subset HS}]}\,\frac{[\mathbf{HS}]}{[\mathbf{LS}]} = \frac{K^{\mathbf{H}}_{\mathbf{SCO}}}{K^{\mathbf{HG}}_{\mathbf{SCO}}} \qquad \text{(S20.)}$$

which is equivalent to Equation S11. Inserting Eqs S15,S16,S18,S19 into S20 results in

$$\frac{K_{\mathbf{G\subset LS}}}{K_{\mathbf{G\subset HS}}} = \frac{K^{\mathbf{H}}_{\mathbf{SCO}}}{K^{\mathbf{HG}}_{\mathbf{SCO}}} = \frac{\gamma_{\mathbf{G\subset LS}}\,[\mathbf{HG}]}{(1-\gamma_{\mathbf{G\subset LS}})[\mathbf{HG}]}\,\frac{(1-\gamma_{\mathbf{LS}})\,[\mathbf{H}]}{\gamma_{\mathbf{LS}}\,[\mathbf{H}]} = \frac{\gamma_{\mathbf{G\subset LS}}-\gamma_{\mathbf{LS}}\cdot\gamma_{\mathbf{G\subset LS}}}{\gamma_{\mathbf{LS}}-\gamma_{\mathbf{LS}}\cdot\gamma_{\mathbf{G\subset LS}}} \qquad \text{(S21.)}$$

As expected, S21 results in similar values as S12 (compare Tables S4 and S5).

Table S5 Spin-state populations of the low-spin host γ_{LS} and LS host-guest complexes $\gamma_{\mathrm{G\subset LS}}$ as derived by VT NMR (Section S5). Ratios of $K_{\mathrm{G\subset HS}}$ and $K_{\mathrm{G\subset LS}}$ were derived from the spin state populations according to Eq. S21. As expected, values are in good agreement to those in Table S4.

	γ_{LS} or $\gamma_{\mathrm{G\subset LS}}$	$\frac{K_{\mathrm{G\subset HS}}}{K_{\mathrm{G\subset LS}}}$	$\frac{K_{\mathrm{G\subset LS}}}{K_{\mathrm{G\subset HS}}}$	$\ln\left(\frac{K_{\mathrm{G\subset HS}}}{K_{\mathrm{G\subset LS}}}\right)$	$\ln\left(\frac{K_{\mathrm{G\subset LS}}}{K_{\mathrm{G\subset HS}}}\right)$
tetrahedron **1**	0.59 ± 0.05				
cyclohexane⊂**1**	0.63 ± 0.06	0.85 ± 0.15	1.18 ± 0.21	−(0.16 ± 0.03)	0.17 ± 0.03
adamantane⊂**1**	0.70 ± 0.05	0.62 ± 0.10	1.62 ± 0.25	−(0.48 ± 0.07)	0.48 ± 0.07
1-adamantanol⊂**1**	0.63 ± 0.05	0.85 ± 0.14	1.18 ± 0.19	−(0.16 ± 0.03)	0.17 ± 0.03
cis-decalin⊂**1**	0.52 ± 0.05	1.32 ± 0.24	0.75 ± 0.14	0.28 ± 0.05	−(0.29 ± 0.05)

The association of guest **G** to the host **H** is slow on the NMR time scale. SCO, however, is fast on the NMR time scale. Thus, no parameters depending on the host's spin states can be derived from NMR. Hence, the only observable association constant by NMR is that of the overall host-guest system K_{obs} as shown in Scheme S5. With Eqs S14&17, Eq. S3 can be written as

$$K_{\mathrm{obs}} = \frac{[\mathbf{HG}]}{[\mathbf{H}]\,[\mathbf{G}]} = \frac{[\mathbf{G\subset HS}]+[\mathbf{G\subset LS}]}{([\mathbf{HS}]+[\mathbf{LS}])\,[\mathbf{G}]} = \frac{[\mathbf{G\subset HS}]}{[\mathbf{H}]\,[\mathbf{G}]} + \frac{[\mathbf{G\subset LS}]}{[\mathbf{H}]\,[\mathbf{G}]} \qquad \text{(S22.)}$$

With Eqs S15&16

$$[\mathbf{H}] = \frac{[\mathbf{LS}]}{\gamma_{\mathbf{LS}}} \qquad \text{(S15)}$$

$$[\mathbf{H}] = \frac{[\mathbf{HS}]}{1-\gamma_{\mathbf{LS}}} \qquad \text{(S16)}$$

Eq. S22 can be written as

$$K_{\mathrm{obs}} = \frac{[\mathbf{G\subset HS}]}{\frac{[\mathbf{HS}]}{1-\gamma_{\mathbf{LS}}}[\mathbf{G}]} + \frac{[\mathbf{G\subset LS}]}{\frac{[\mathbf{LS}]}{\gamma_{\mathbf{LS}}}[\mathbf{G}]} = (1-\gamma_{\mathbf{LS}})\,K_{\mathbf{G\subset HS}} + \gamma_{\mathbf{LS}}\,K_{\mathbf{G\subset LS}} \qquad \text{(S23.)}$$

Inserting Eq. S21 into S23 allows to calculate $K_{\mathrm{G\subset HS}}$ and $K_{\mathrm{G\subset LS}}$ from K_{obs}:

$$K_{\mathbf{G\subset LS}} = \frac{\gamma_{\mathbf{G\subset LS}}-\gamma_{\mathbf{LS}}\cdot\gamma_{\mathbf{G\subset LS}}}{\gamma_{\mathbf{LS}}-\gamma_{\mathbf{LS}}\cdot\gamma_{\mathbf{G\subset LS}}}\,K_{\mathbf{G\subset HS}} \;\therefore\; K_{\mathbf{G\subset HS}} = \frac{\gamma_{\mathbf{LS}}-\gamma_{\mathbf{LS}}\cdot\gamma_{\mathbf{G\subset LS}}}{\gamma_{\mathbf{G\subset LS}}-\gamma_{\mathbf{LS}}\cdot\gamma_{\mathbf{G\subset LS}}}\,K_{\mathbf{G\subset LS}} \qquad \text{(S21)}$$

$$\begin{aligned} K_{\mathrm{obs}} &= (1-\gamma_{\mathbf{LS}})\,\frac{\gamma_{\mathbf{LS}}-\gamma_{\mathbf{LS}}\cdot\gamma_{\mathbf{G\subset LS}}}{\gamma_{\mathbf{G\subset LS}}-\gamma_{\mathbf{LS}}\cdot\gamma_{\mathbf{G\subset LS}}}\,K_{\mathbf{G\subset LS}} + \gamma_{\mathbf{LS}}\,K_{\mathbf{G\subset LS}} \\ &= \left(\frac{(1-\gamma_{\mathbf{LS}})(\gamma_{\mathbf{LS}}-\gamma_{\mathbf{LS}}\cdot\gamma_{\mathbf{G\subset LS}})}{\gamma_{\mathbf{G\subset LS}}\,(1-\gamma_{\mathbf{LS}})} + \gamma_{\mathbf{LS}}\right)K_{\mathbf{G\subset LS}} \\ &= \left(\frac{\gamma_{\mathbf{LS}}-\gamma_{\mathbf{LS}}\cdot\gamma_{\mathbf{G\subset LS}}}{\gamma_{\mathbf{G\subset LS}}} + \frac{\gamma_{\mathbf{LS}}\cdot\gamma_{\mathbf{G\subset LS}}}{\gamma_{\mathbf{G\subset LS}}}\right)K_{\mathbf{G\subset LS}} \end{aligned}$$

$$= \frac{\gamma_{\mathbf{LS}}}{\gamma_{\mathbf{G\subset LS}}}\,K_{\mathbf{G\subset LS}} \qquad \text{(S24.)}$$

and

$$\begin{aligned} K_{\mathrm{obs}} &= (1-\gamma_{\mathbf{LS}})\,K_{\mathbf{G\subset HS}} + \gamma_{\mathbf{LS}}\,\frac{\gamma_{\mathbf{G\subset LS}}-\gamma_{\mathbf{LS}}\cdot\gamma_{\mathbf{G\subset LS}}}{\gamma_{\mathbf{LS}}-\gamma_{\mathbf{LS}}\cdot\gamma_{\mathbf{G\subset LS}}}\,K_{\mathbf{G\subset HS}} \\ &= \left((1-\gamma_{\mathbf{LS}}) + \frac{\gamma_{\mathbf{G\subset LS}}-\gamma_{\mathbf{LS}}\cdot\gamma_{\mathbf{G\subset LS}}}{1-\gamma_{\mathbf{G\subset LS}}}\right)K_{\mathbf{G\subset HS}} \end{aligned}$$

$$= \left(\frac{(1-\gamma_{\mathbf{LS}})(1-\gamma_{\mathbf{G\subset LS}})}{1-\gamma_{\mathbf{G\subset LS}}} + \frac{\gamma_{\mathbf{G\subset LS}} - \gamma_{\mathbf{LS}} \cdot \gamma_{\mathbf{G\subset LS}}}{1-\gamma_{\mathbf{G\subset LS}}} \right) K_{\mathbf{G\subset HS}}$$

$$= \left(\frac{1-\gamma_{\mathbf{G\subset LS}} - \gamma_{\mathbf{LS}} + \gamma_{\mathbf{LS}} \cdot \gamma_{\mathbf{G\subset LS}} + \gamma_{\mathbf{G\subset LS}} - \gamma_{\mathbf{LS}} \cdot \gamma_{\mathbf{G\subset LS}}}{1-\gamma_{\mathbf{G\subset LS}}} \right) K_{\mathbf{G\subset HS}}$$

$$= \frac{1-\gamma_{\mathbf{LS}}}{1-\gamma_{\mathbf{G\subset LS}}} K_{\mathbf{G\subset HS}}. \qquad \text{(S25.)}$$

Hence $K_{\mathbf{G\subset HS}}$ and $K_{\mathbf{G\subset LS}}$ can be calculated directly from K_{obs} and the spin state populations $\gamma_{\mathbf{LS}}$ and $\gamma_{\mathbf{G\subset LS}}$:

$$K_{\mathbf{G\subset HS}} = \frac{1-\gamma_{\mathbf{G\subset LS}}}{1-\gamma_{\mathbf{LS}}} K_{\mathrm{obs}} \qquad \text{(S26.)}$$

$$K_{\mathbf{G\subset LS}} = \frac{\gamma_{\mathbf{G\subset LS}}}{\gamma_{\mathbf{LS}}} K_{\mathrm{obs}} \qquad \text{(S27.)}$$

4.9.2 NMR titrations

NMR titration data of the different guests binding to tetrahedron **1** can be fitted to a simple 1:1 isotherm to determine K_{obs} (see section 4.9.1). With Eqs S26 and S27, the association constants of the guests to the two spin states (all high-spin **HS** or all low-spin **LS**) $K_{\mathbf{G\subset HS}}$ and $K_{\mathbf{G\subset LS}}$ can be calculated.

Solutions of the different guests (between 4.12 and 18.5 mM, CD_3CN) containing two internal standards, 1,3,5-trimethoxybenzene and 1,4-dimethoxybenzene at 1.683 mM and 1.954 mM, respectively, were titrated into solutions of tetrahedron **1** (0.32 mM, 0.4 mL, CD_3CN) containing internal standard at 1,3,5-trimethoxybenzene at 1.683 mM. After each addition, the system was left to equilibrate for four minutes at room temperature before the ^{1}H NMR spectrum (Figures S63−S66) was taken (equilibrating for longer times leads to the formation of the host-guest complex from NMR silent fragments/intermediates in the mixture via templation by the guest). Additionally, a spectrum of each guest stock solution was recorded. Since host and guest solutions both contained the same internal standard (1,3,5-trimethoxybenzene) at the same concentration, the concentration of the standard 1,3,5-trimethoxybenzene remained constant throughout the titration. Concentrations of tetrahedron **1** and its host-guest complexes **G⊂1** were determined via integration with reference to internal standard 1,3,5-trimethoxybenzene. The second internal standard, 1,4-dimethoxybenzene, was only present in the guest solutions and, hence, it's concentration in the samples rose together with the guest concentration. Therefore, precise guest concentrations were determined with reference to both internal standards and not directly from integration of guest signals, because these often overlapped with other signals. Integrals were determined by calculated Lorentzian peaks.

As guest binding is slow on the NMR time scale, the data were analyzed by plotting the ratio of occupied cage **G⊂1** to total concentration of tetrahedron **1** ([**G⊂1**]/([**G⊂1**]+[**1**])) as a function of the host-guest ratio [**G**]/[**1**] (Figures S67−S68).[25–27] The resulting isotherm was fitted to a standard 1:1 host-guest binding model using Origin Pro 8G:

$$\frac{[\mathbf{G\subset 1}]}{[\mathbf{G\subset 1}]+[\mathbf{1}]} = \frac{1}{2}\left(1+\frac{[\mathbf{G}]_0}{[\mathbf{1}]_0}+\frac{1}{[\mathbf{1}]_0 K_{\mathbf{G\subset 1}}}\right) - \sqrt{\frac{1}{4}\left(1+\frac{[\mathbf{G}]_0}{[\mathbf{1}]_0}+\frac{1}{[\mathbf{1}]_0 K_{\mathbf{G\subset 1}}}\right)^2 - \frac{[\mathbf{G}]_0}{[\mathbf{1}]_0}} \quad \text{(S28.)}$$

Note that the maximum value of this isotherm is 1, because there cannot be more than 100% of the cages occupied. Hence, the isotherm does not need as many points above [**G**]/[**1**]=1 as a system, which is on fast exchange on the NMR timescale and the maximum value of the isotherm δ_{max} is unknown and needs to be determined by fitting.

To reduce errors, two signals were followed for each titration and the collated data fitted using Origin's Global Fit function (Figures S67−S68, Table S6).

Table S6 Spin-state populations of the low-spin host $\gamma_{\mathbf{LS}}$ and LS host-guest complexes $\gamma_{\mathbf{G\subset LS}}$ as derived by VT NMR (Section S5). Association constants K_{obs} obtained by global non-linear curve fitting according to Equation S28 (graphs see Figures S67–S68) of the isotherms obtained by ^{1}H NMR titrations (Figures S63–S66). The individual association constants of the two spin states $K_{\mathbf{G\subset HS}}$ and $K_{\mathbf{G\subset LS}}$ were obtained from K_{obs} according to Equations S26 and S27. Logarithmic ratios of $K_{\mathbf{G\subset HS}}$ and $K_{\mathbf{G\subset LS}}$ $\ln(K_{\mathbf{G\subset HS}} \cdot K_{\mathbf{G\subset LS}}^{-1})$ were derived from the spin state populations according to Equation S21.

	$\gamma_{\mathbf{LS}}$ or $\gamma_{\mathbf{G\subset LS}}$	K_{obs} [10^3 M^{-1}]	$K_{\mathbf{G\subset LS}}$ [10^3 M^{-1}]	$K_{\mathbf{G\subset HS}}$ [10^3 M^{-1}]	$\ln\left(\frac{K_{\mathbf{G\subset HS}}}{K_{\mathbf{G\subset LS}}}\right)$
tetrahedron **1**	0.59 ± 0.05				
cyclohexane⊂**1**	0.63 ± 0.06	9.0 ± 0.4	9.6 ± 2.2	8.1 ± 1.8	−(0.16 ± 0.03)
adamantane⊂**1**	0.70 ± 0.05	73 ± 16	87 ± 33	53 ± 20	−(0.48 ± 0.07)
1-adamantanol⊂**1**	0.63 ± 0.05	230 ± 50	250 ± 90	210 ± 80	−(0.16 ± 0.03)
cis-decalin⊂**1**	0.52 ± 0.05	7.2 ± 0.5	6.3 ± 1.6	8.4 ± 2.1	0.28 ± 0.05

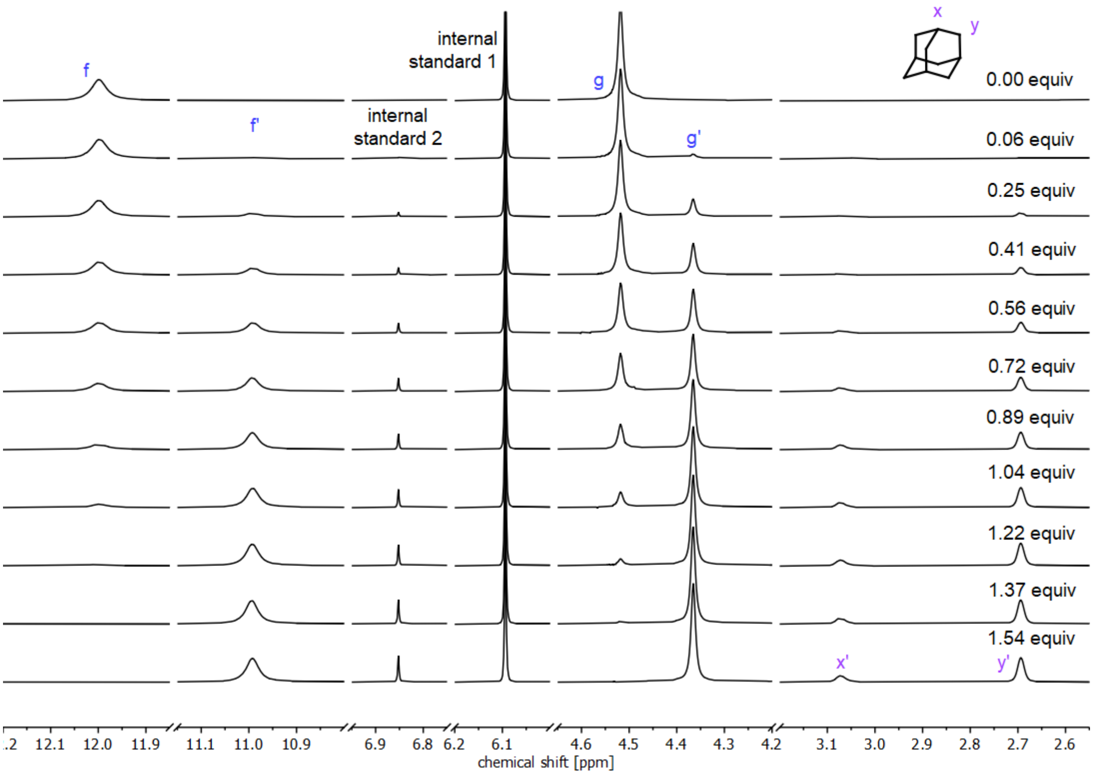

Figure S63 Partial ^1H NMR spectra (CD_3CN, 500 MHz, 298 K) of tetrahedron **1** (c = 0.32 mM) upon addition of 0 − 1.5 equiv. of adamantane. Phenyl protons H_f and methyl protons H_g of tetrahedron **1** and the adamantane protons H_x and H_y are marked in blue and purple, respectively. Protons signals from the host-guest complexes are indicated with a prime.

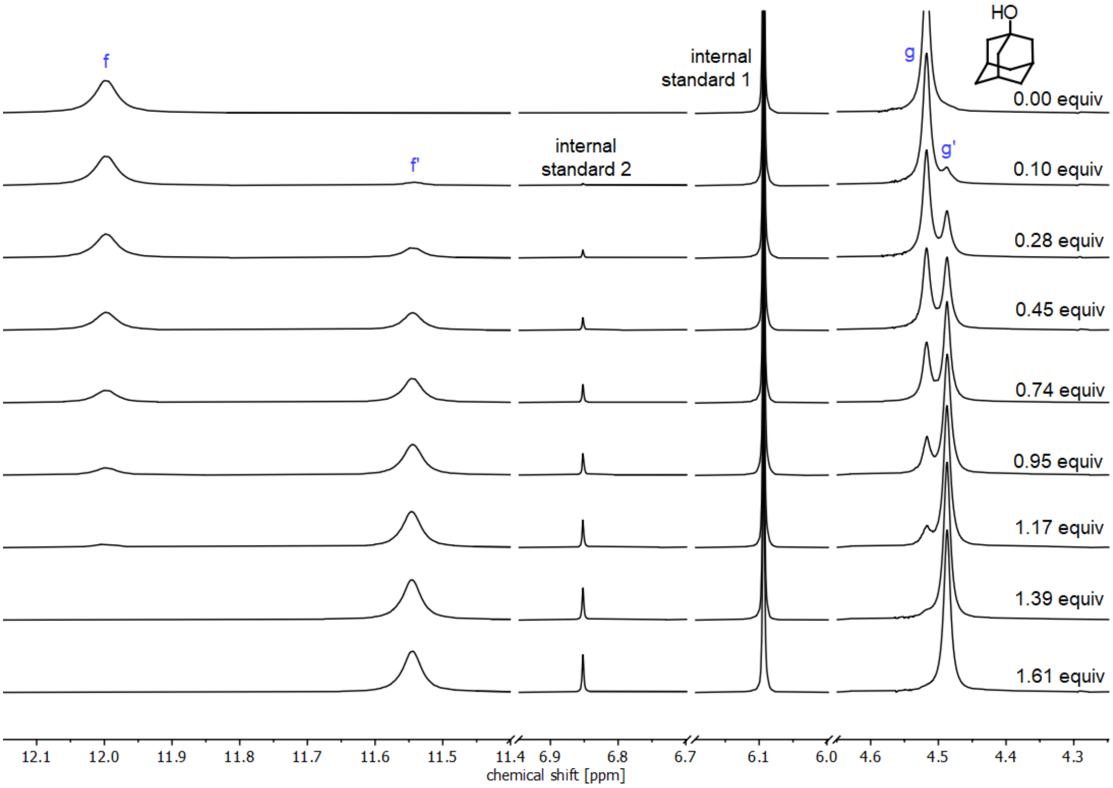

Figure S64 Partial ^1H NMR spectra (CD_3CN, 500 MHz, 298 K) of tetrahedron **1** (c = 0.32 mM) upon addition of 0 − 1.6 equiv. of 1-adamantanol. Phenyl protons H_f and methyl protons H_g of tetrahedron **1** are marked in blue. Protons signals from the host-guest complexes are indicated with a prime.

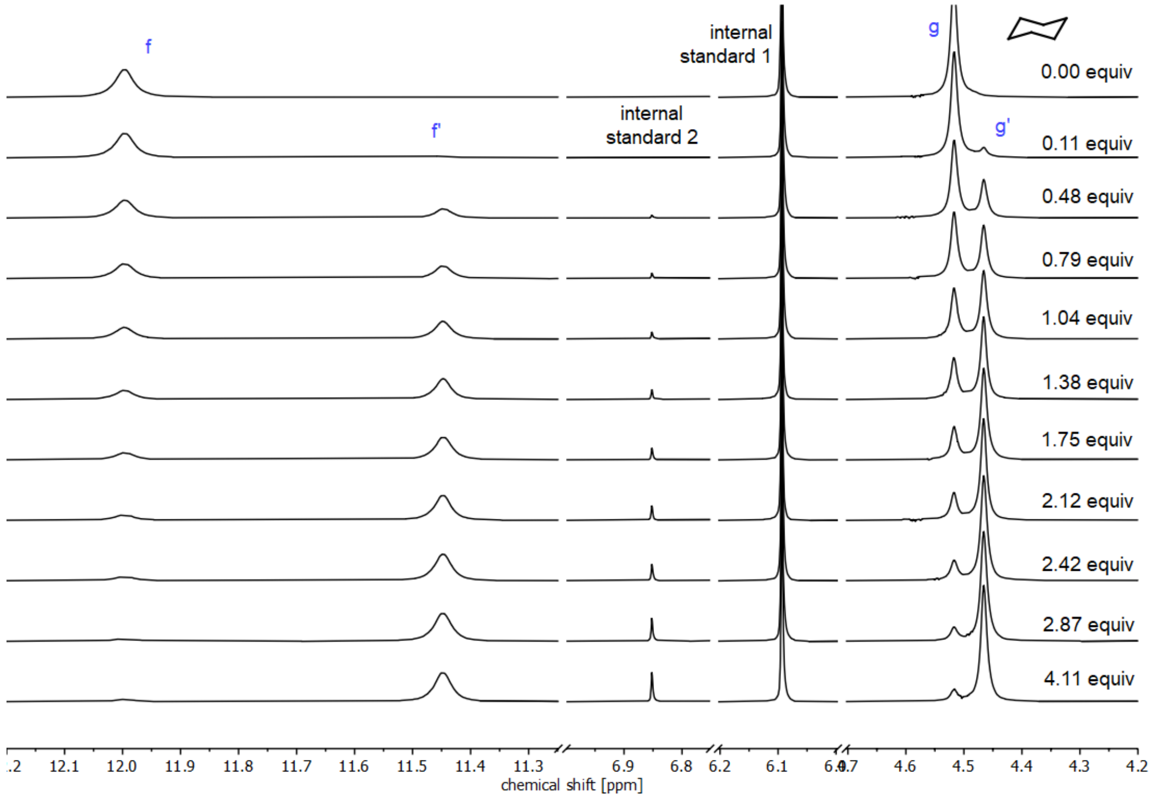

Figure S65 Partial ^{1}H NMR spectra (CD_3CN, 500 MHz, 298 K) of tetrahedron **1** (c = 0.32 mM) upon addition of 0 – 4.1 equiv. of cyclohexane. Phenyl protons H_f and methyl protons H_g of tetrahedron **1** are marked in blue. Protons signals from the host-guest complexes are indicated with a prime.

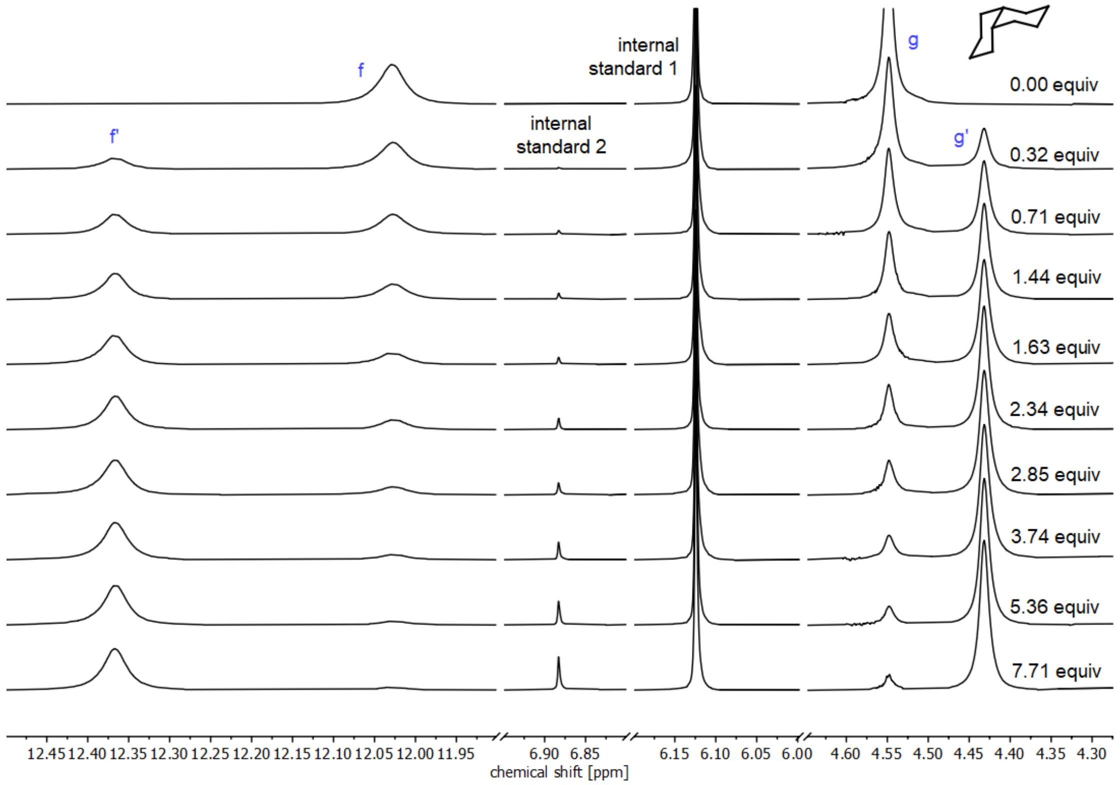

Figure S66 Partial ^{1}H NMR spectra (CD_3CN, 500 MHz, 298 K) of tetrahedron **1** (c = 0.32 mM) upon addition of 0 – 7.7 equiv. of *cis*-decalin. Phenyl protons H_f and methyl protons H_g of tetrahedron **1** are marked in blue. Protons signals from the host-guest complexes are indicated with a prime.

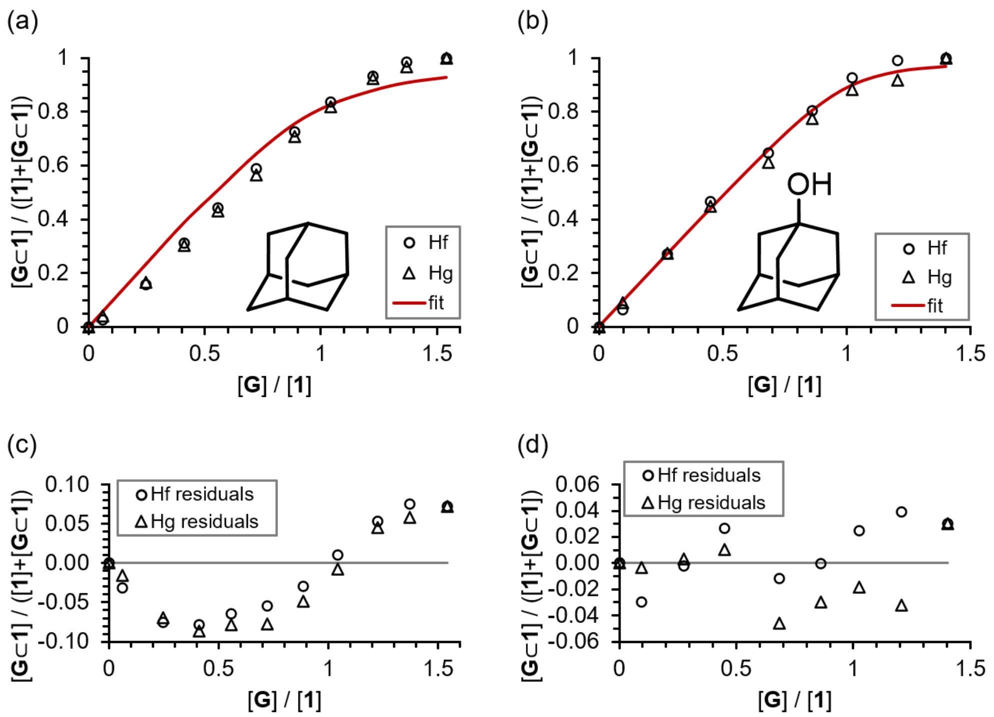

Figure S67 Association isotherms fitted according to Equation S28 and respective fit residuals obtained from titration of different guests to tetrahedron **1** (exemplary spectra see Figures S63&S64). Isotherms from phenylene protons H_f (○) and methyl protons H_g (Δ) were fitted simultaneously (—): (a) adamantane, (b) 1-adamantanol, (c) Fit residuals of (a). (d) Fit residuals of (b).

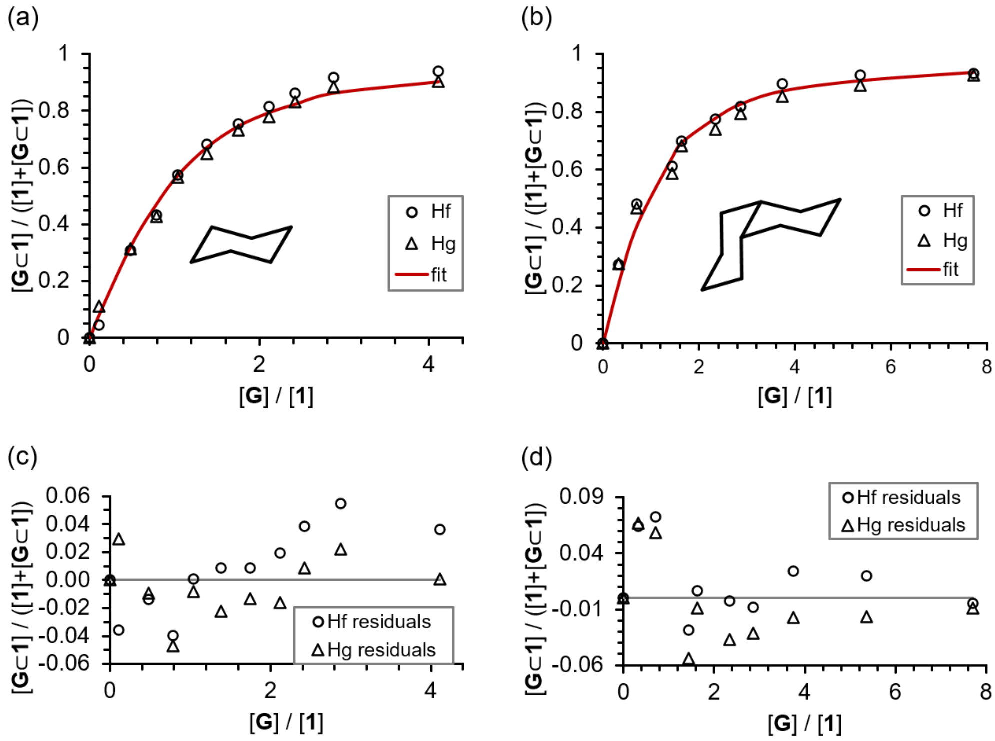

Figure S68 Association isotherms fitted according to Equation S28 and respective fit residuals obtained from titration of different guests to tetrahedron **1** (exemplary spectra see Figures S65&S66). Isotherms from phenylene protons H_f (○) and methyl protons H_g (Δ) were fitted simultaneously (—): (a) cyclohexane, (b) *cis*-decalin, (c) Fit residuals of (a). (d) Fit residuals of (b).

5 Variable-Temperature ^{1}H NMR and Spin-Crossover Studies

Variable-temperature ^{1}H NMR spectroscopy was performed to study the SCO behaviors of tetrahedron **1** and its host-guest complexes. In the solution state, the cooperativity between SCO centers is quite weak and can be neglected. Hence, an ideal solution model was applied to investigate their SCO properties with the following equation:[28]

$$\delta = \delta_{\mathbf{LS}} + \frac{C}{T + Te^{\frac{\Delta H - T\,\Delta S}{RT}}} \qquad \text{(S29.)}$$

In this equation, δ is the chemical shift of the selected peak; $\delta_{\mathbf{LS}}$ is the chemical shift of the peak when the compound is at the LS state; C is a constant; T corresponds to the temperature; ΔH and ΔS correspond to the enthalpy and entropy change of the SCO process and R is the gas constant. In most cases where the spin crossover process is almost complete but the absolute LS state cannot be reached, the value of $\delta_{\mathbf{LS}}$ was fixed to the value of their diamagnetic Zn analogue (see Section S4.8) in the fitting. This method has the key advantage that the selected chemical shifts of the ^{1}H NMR resonances of the SCO complex directly reflect its magnetic state and will not interfere with other compounds in the mixture, which is a drawback of bulk methods such as the Evans method.

Spin state populations were calculated with the thermodynamic parameters obtained from the fitting.[30]

$$\gamma_{\mathbf{LS}} = \frac{1}{1 + e^{-\frac{\Delta H - T\,\Delta S}{RT}}} \qquad \text{(S30.)}$$

$$\gamma_{\mathbf{HS}} = 1 - \frac{1}{1 + e^{-\frac{\Delta H - T\,\Delta S}{RT}}} \qquad \text{(S31.)}$$

Table S7 Summary of thermodynamic parameters of all compounds

	**Δ*H*** **kJ mol⁻¹**	**Δ*S*** **J mol⁻¹·K⁻¹**	**$T_{1/2}$** **K**
tetrahedron **1**	26.2 ± 0.2	85 ± 1	308
adamantane⊂**1**	27.1 ± 0.3	84 ± 1	323
1-adamantanol⊂**1**	26.4 ± 0.3	84 ± 1	314
cyclohexane⊂**1**	27.0 ± 0.3	86 ± 1	314
cis-decalin⊂**1**	17.9 ± 0.2	59 ± 1	303
tetrahedron **3**	44.7 ± 2	108 ± 11	414
adamantane⊂**3**	44.7 ± 1	108 ± 4	414
cube **4**	27.1 ± 0.6	81 ± 2	313

5.1 Tetrahedron **1**

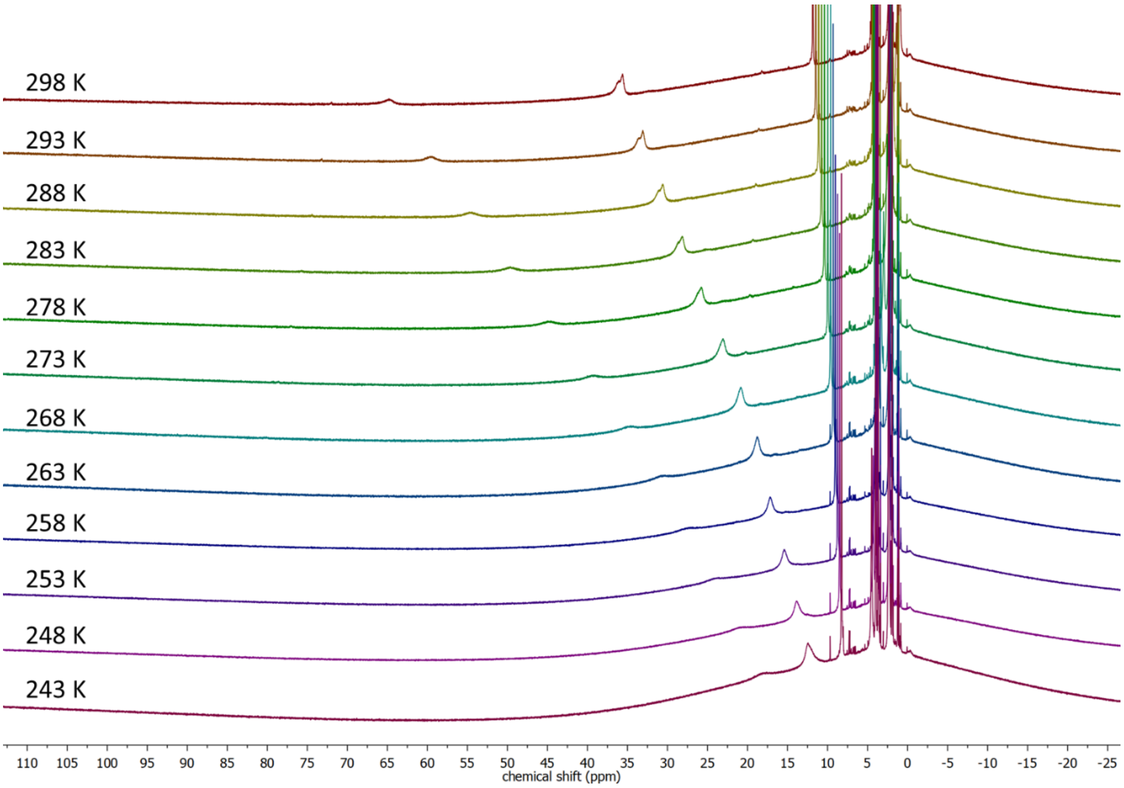

Figure S69 VT ^1H NMR spectra of tetrahedron **1** (CD_3CN, 500 MHz, from 298 K to 243 K).

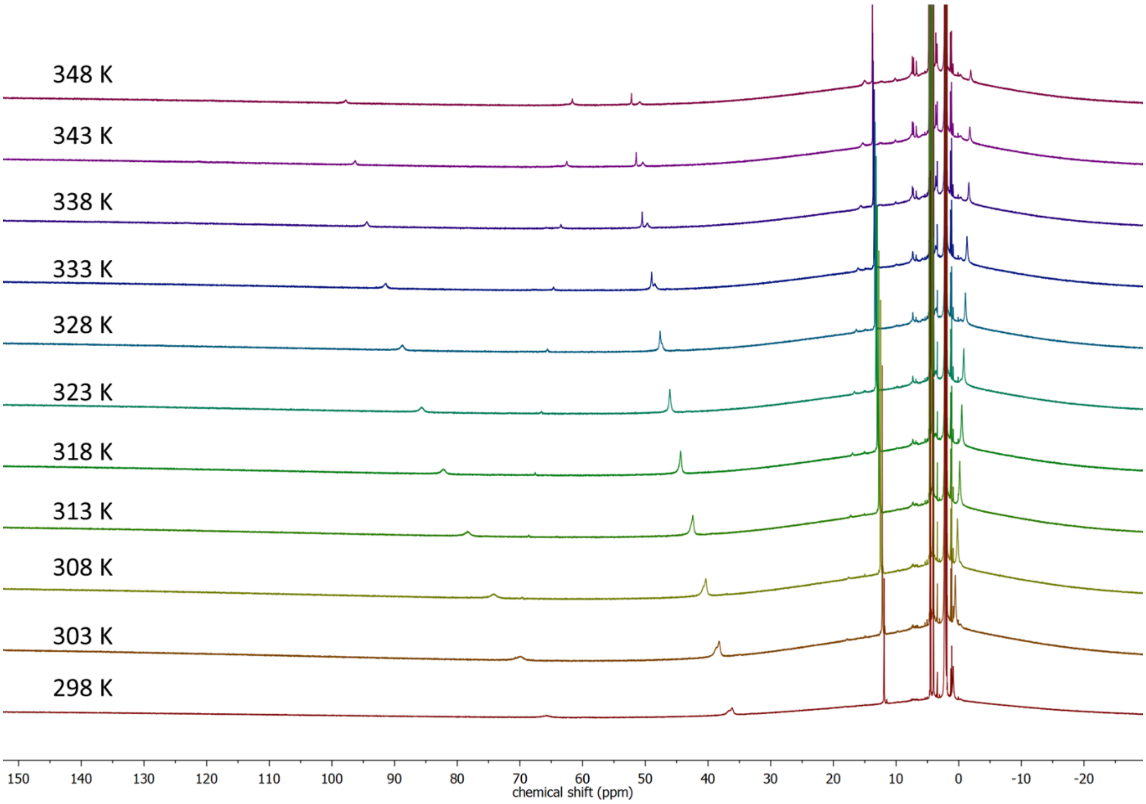

Figure S70 VT ^1H NMR spectra of **1** (CD_3CN, 500 MHz, from 298 K to 348 K).

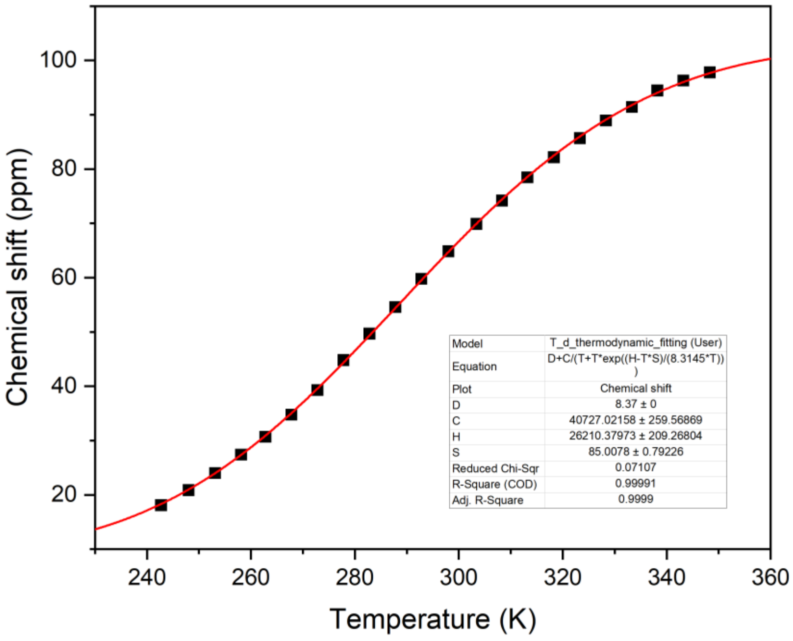

Figure S71 ^{1}H NMR chemical shifts of the imine proton H_a of **1** as a function of temperature (■, Figures S69, S70) fitted according to Equation S29 (—).

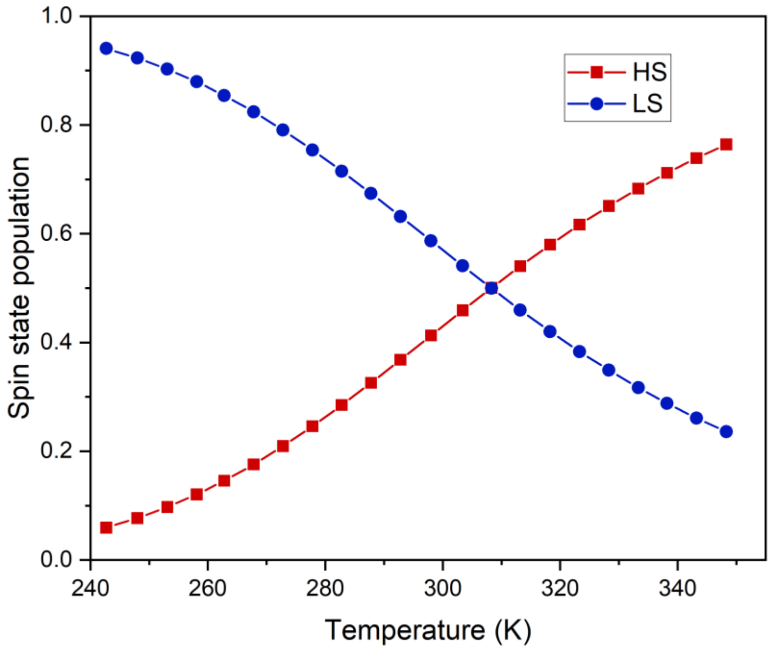

Figure S72 Spin state populations of **1** calculated according to Equations S30 and S31.

5.2 Adamantane⊂**1**

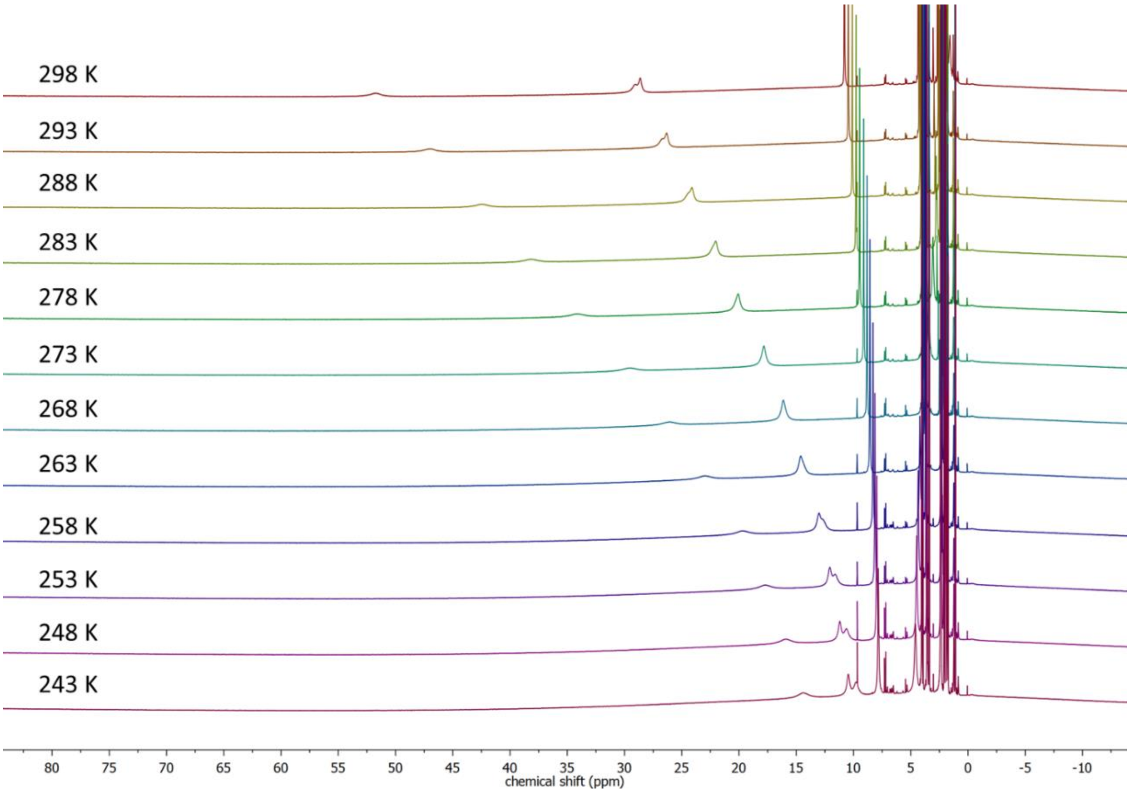

Figure S73 VT ^{1}H NMR spectra of adamantane⊂**1** (CD_3CN, 500 MHz, from 298 K to 243 K).

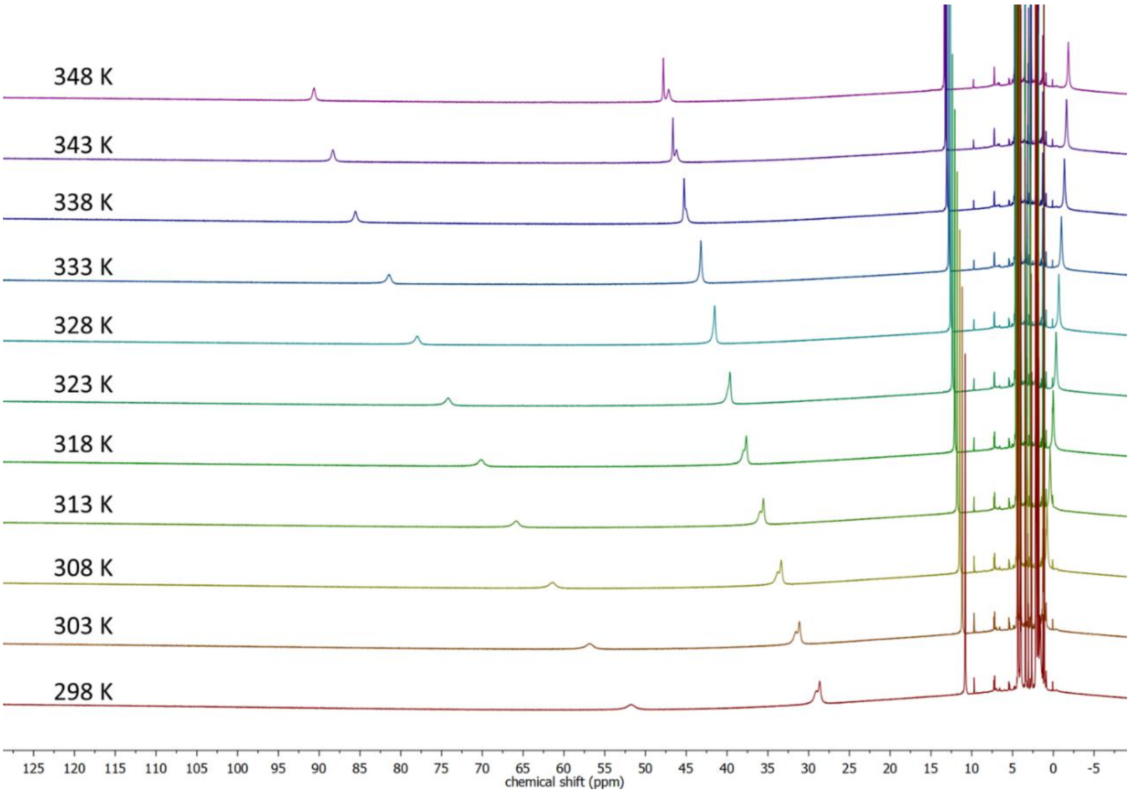

Figure S74 VT ^{1}H NMR spectra of adamantane⊂**1** (CD_3CN, 500 MHz, from 298 K to 348 K).

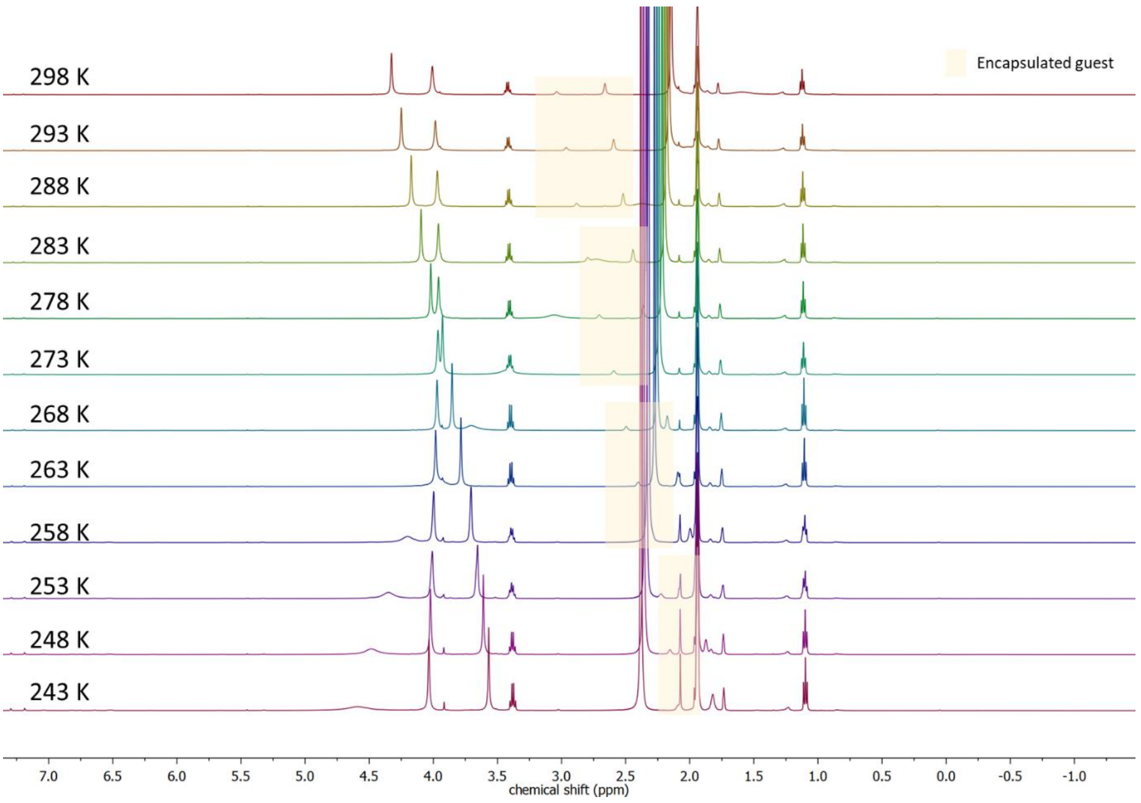

Figure S75 VT ^{1}H NMR spectra of adamantane⊂**1** in the diamagnetic region (CD_3CN, 500 MHz, from 298 K to 243 K).

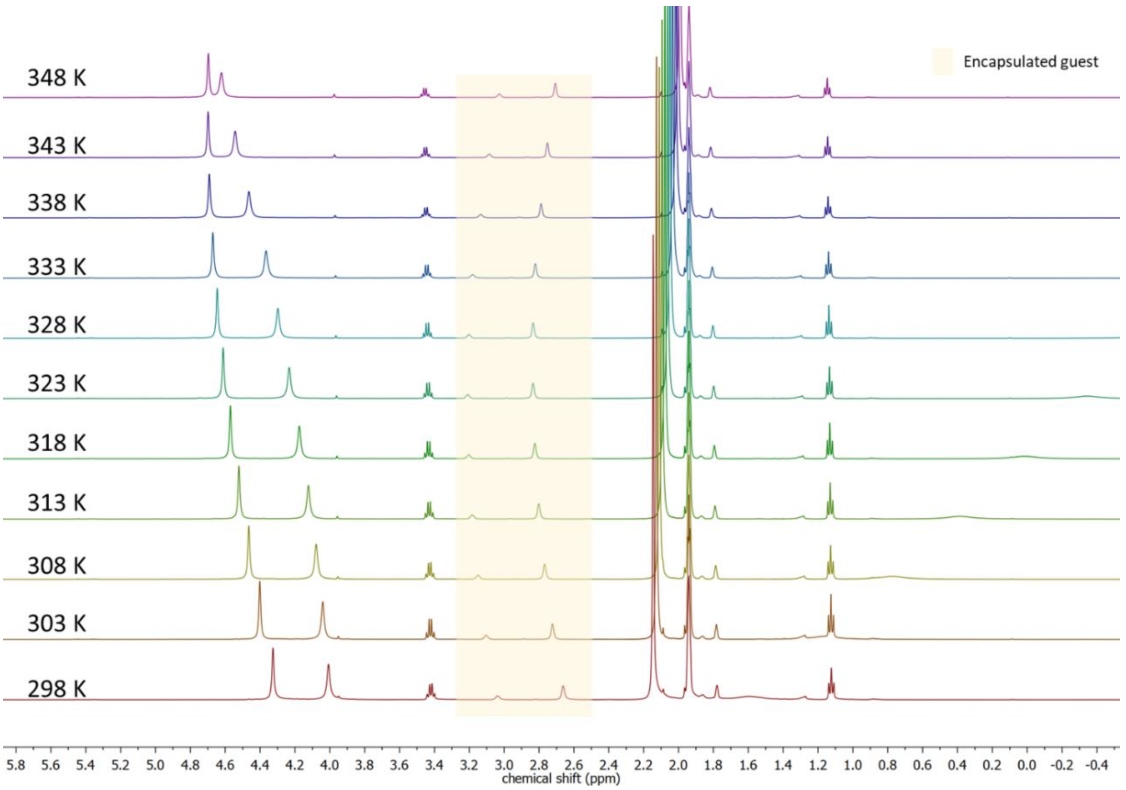

Figure S76 VT ^{1}H NMR spectra of adamantane⊂**1** in the diamagnetic region (CD_3CN, 500 MHz, from 298 K to 348 K).

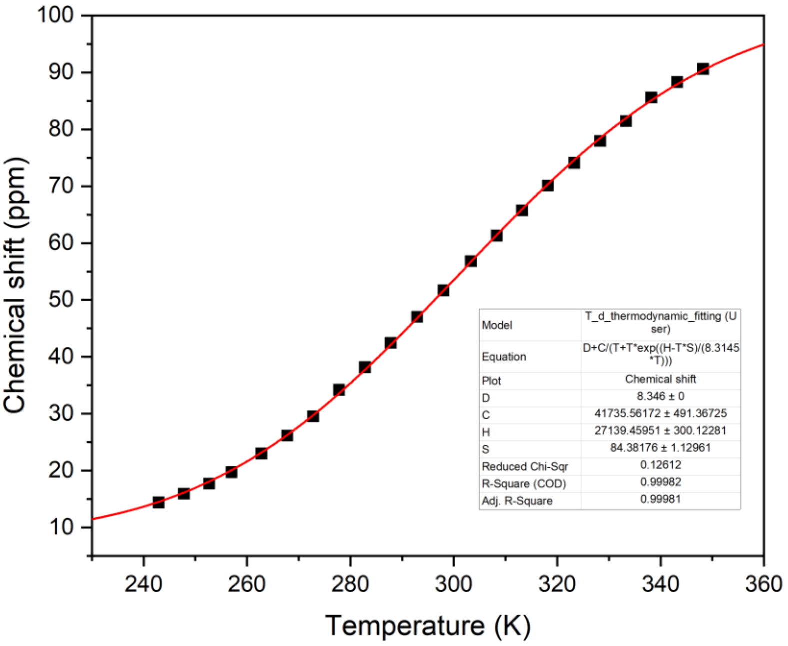

Figure S77 ^{1}H NMR chemical shifts of the imine proton H_a of adamantane⊂**1** as a function of temperature (■, Figures S73, S74) fitted according to Equation S29 (—).

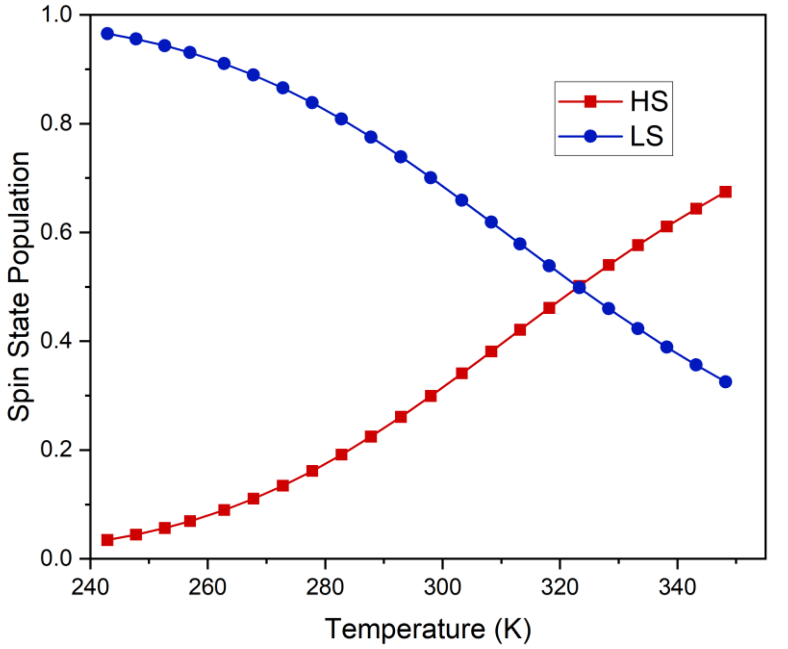

Figure S78 Spin state population of adamantane⊂**1** calculated according to Equations S30 and S31.

5.3 1-Adamantanol⊂**1**

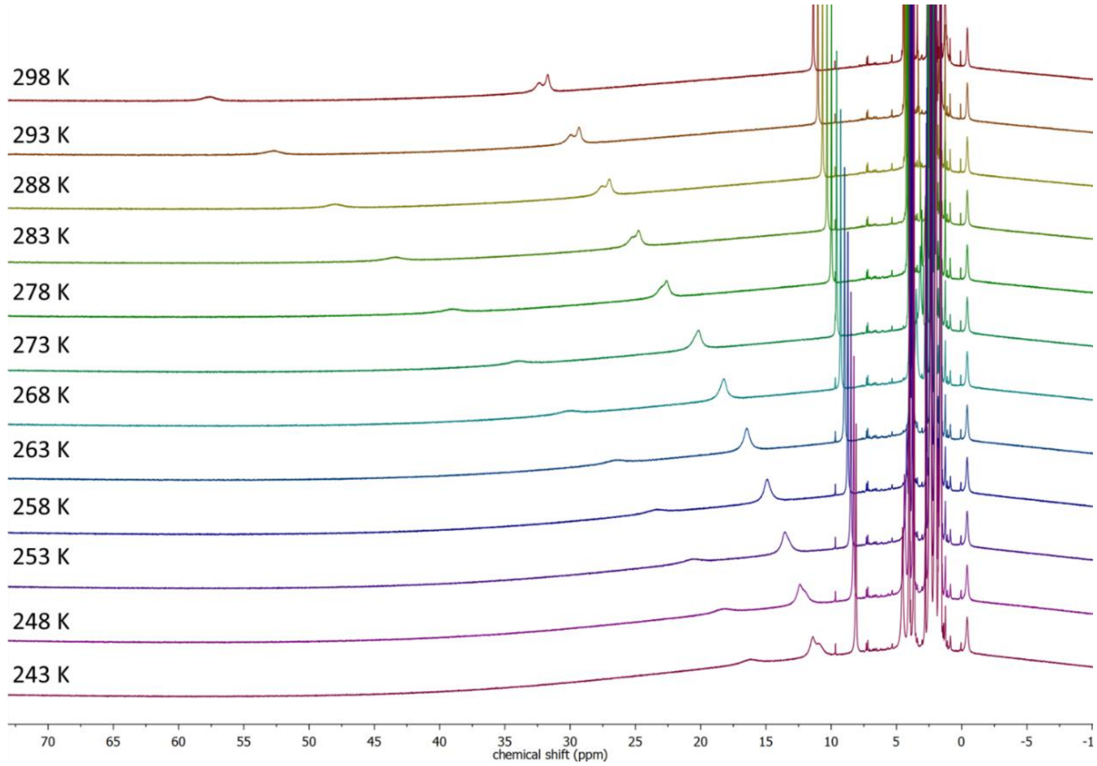

Figure S79 VT ^{1}H NMR spectra of 1-adamantanol⊂**1** (CD_3CN, 500 MHz, from 298 K to 243 K).

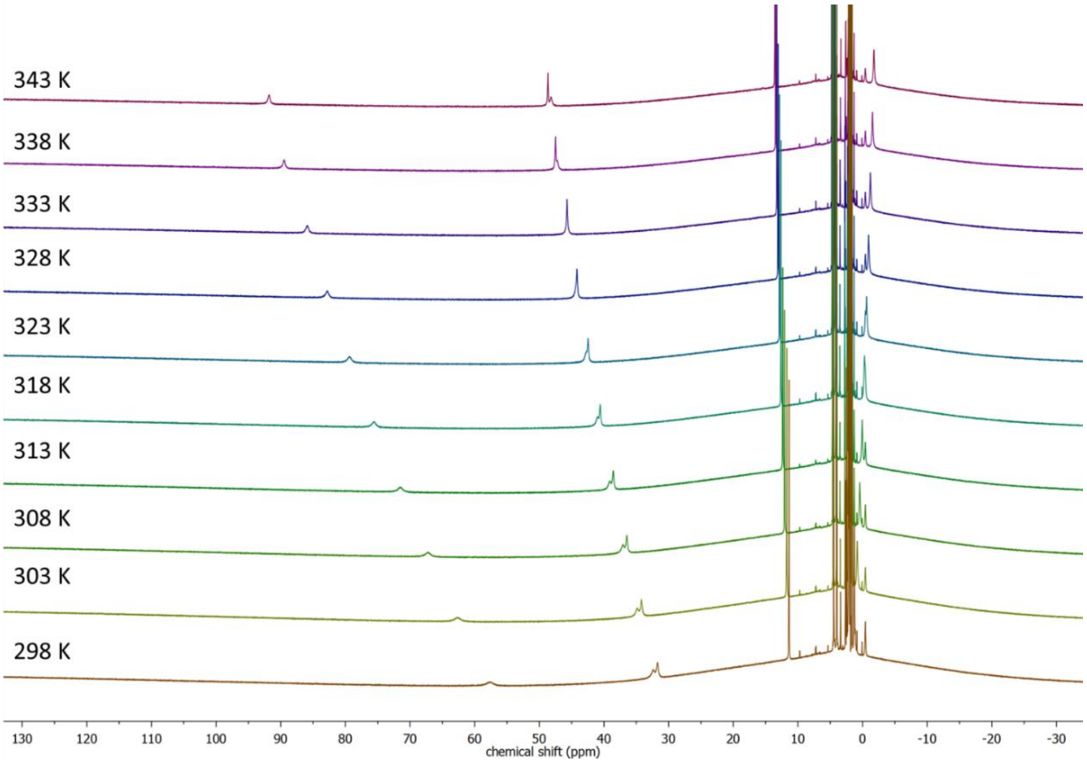

Figure S80 VT ^{1}H NMR spectra of 1-adamantanol⊂**1** (CD_3CN, 500 MHz, from 298 K to 343 K).

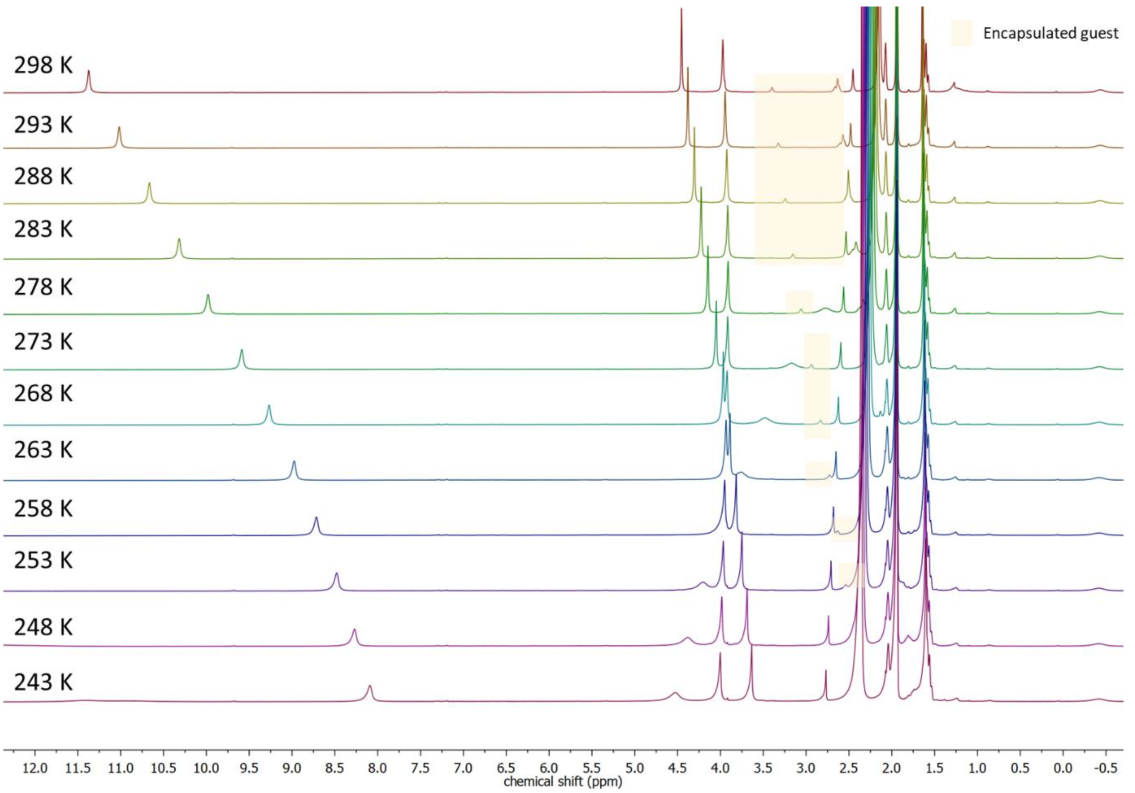

Figure S81 VT ^{1}H NMR spectra of 1-adamantanol⊂**1** in the diamagnetic region (CD_3CN, 500 MHz, from 298 K to 243 K).

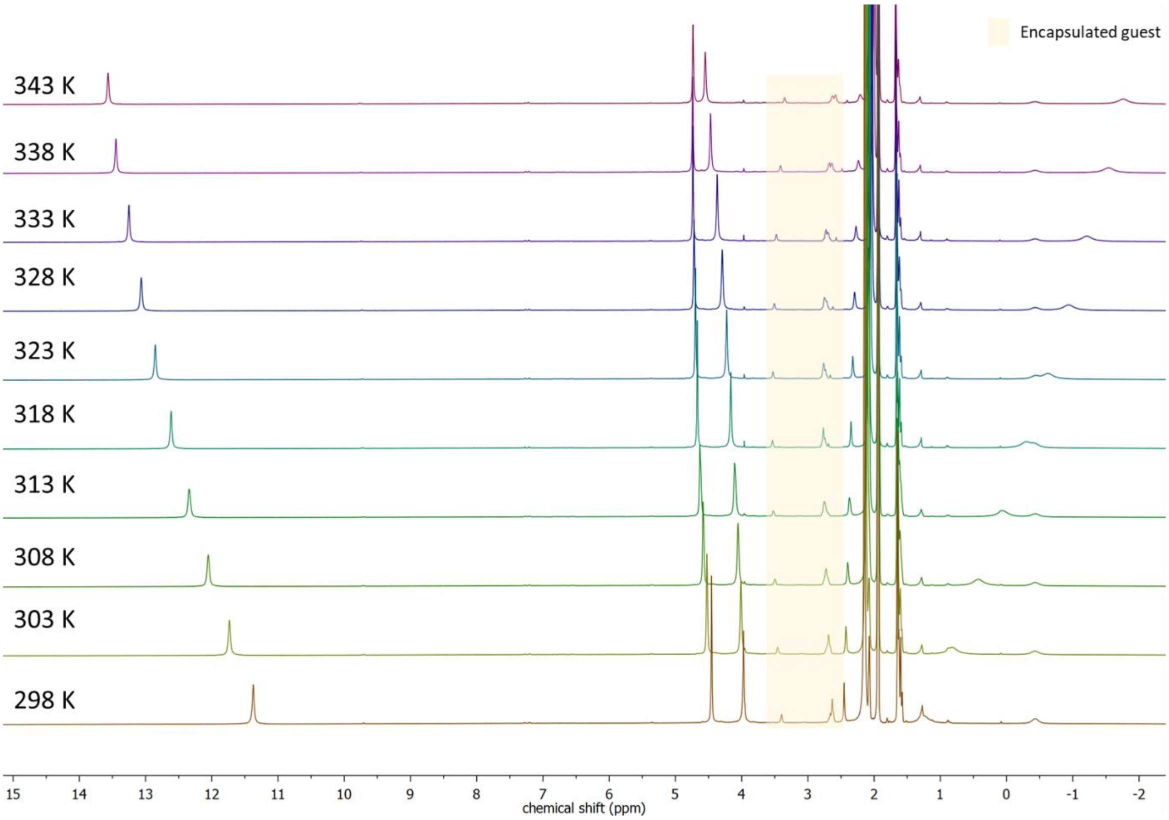

Figure S82 VT ^{1}H NMR spectra of 1-adamantanol⊂**1** in the diamagnetic region (CD_3CN, from 298 K to 343 K).

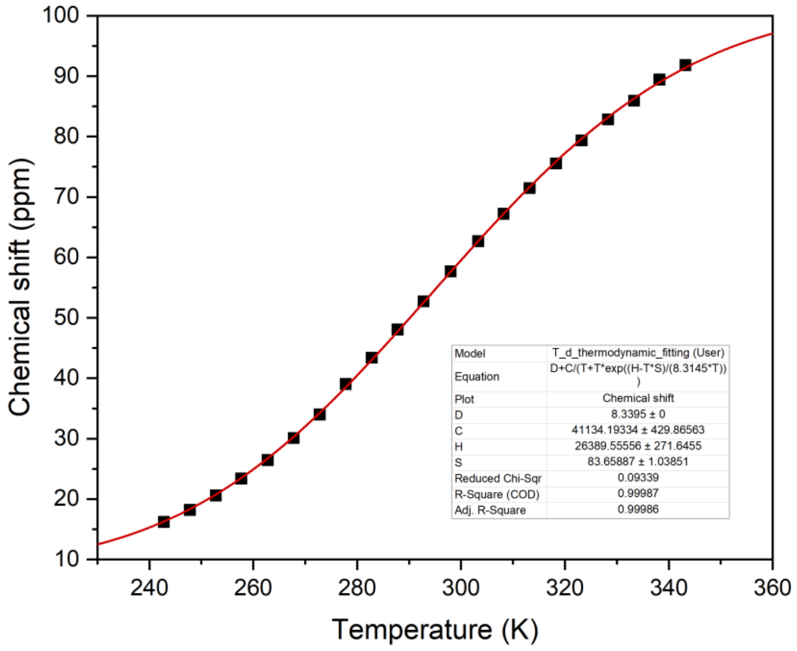

Figure S83 ^{1}H NMR chemical shifts of the imine proton H_a of 1-adamantanol⊂**1** as a function of temperature (■, Figures S77, S78) fitted according to Equation S29 (—).

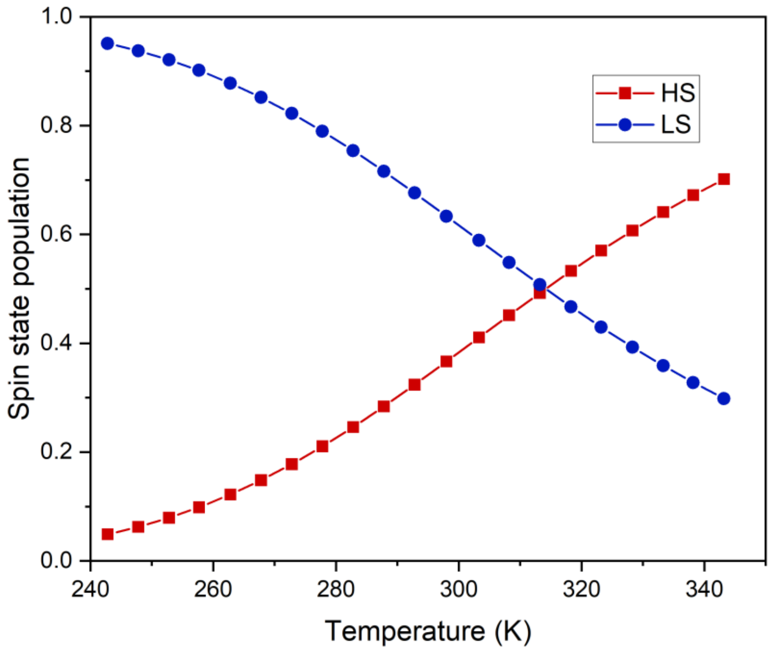

Figure S84 Spin state population of 1-adamantanol⊂**1** calculated according to Equations S30 and S31.

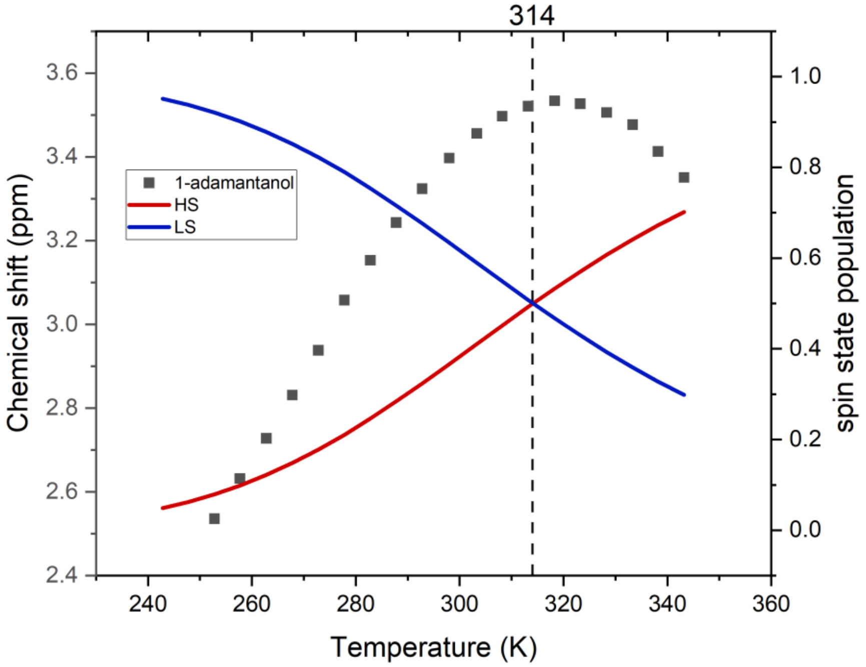

Figure S85 Chemical shifts of encapsulated 1-adamantanol (H_x, black squares) and spin state population of 1-adamantanol⊂**1** (blue and red curves) in the investigated temperature range. The SCO $T_{1/2}$ is given as a vertical line.

5.4 Cyclohexane⊂**1**

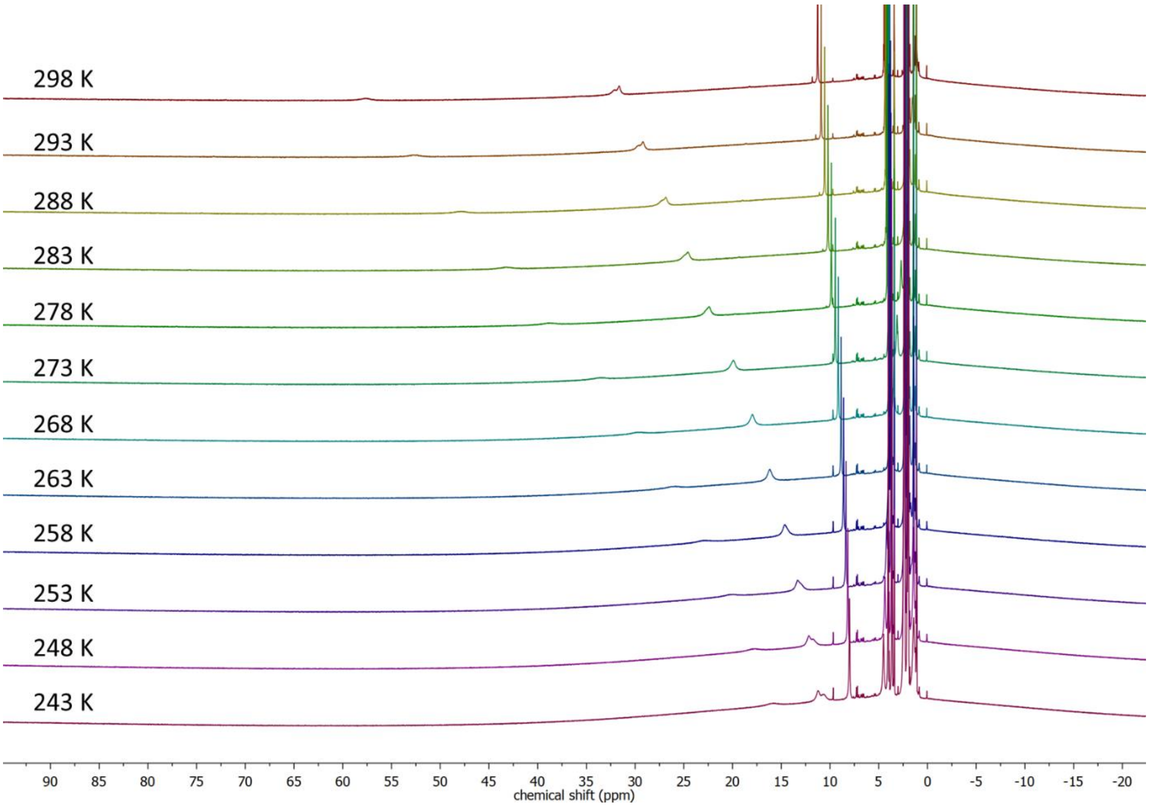

Figure S86 VT ^{1}H NMR spectra of cyclohexane⊂**1** (CD_3CN, 500 MHz, from 298 K to 243 K).

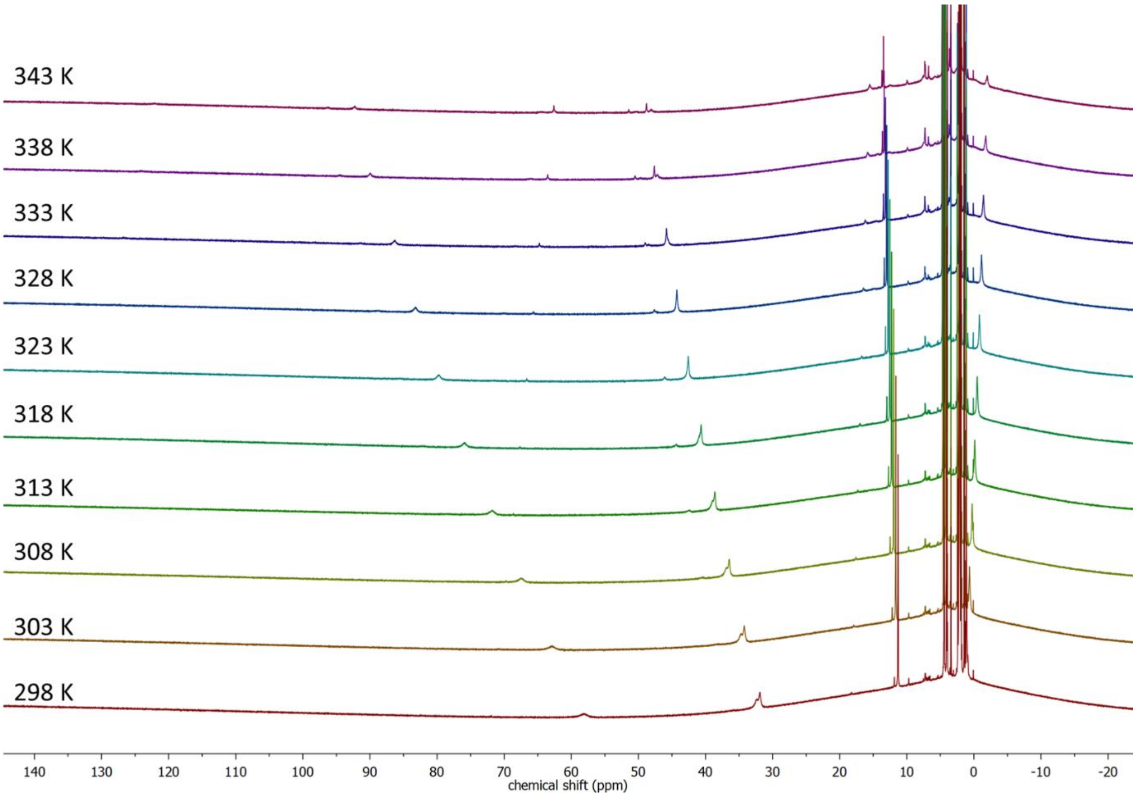

Figure S87 VT ^{1}H NMR spectra of cyclohexane⊂**1** (CD_3CN, 500 MHz, from 298 K to 343 K).

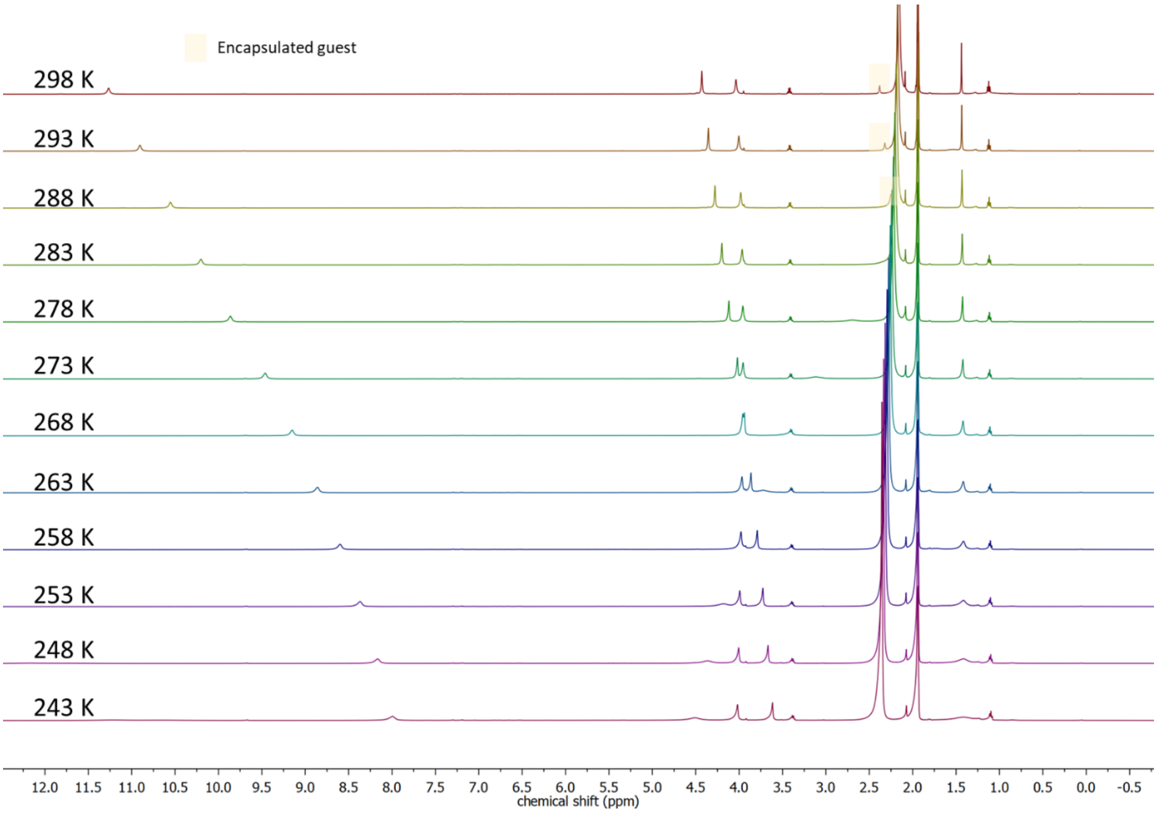

Figure S88 VT ^{1}H NMR spectra of cyclohexane⊂**1** in the diamagnetic region (CD_3CN, 500 MHz, from 298 K to 243 K).

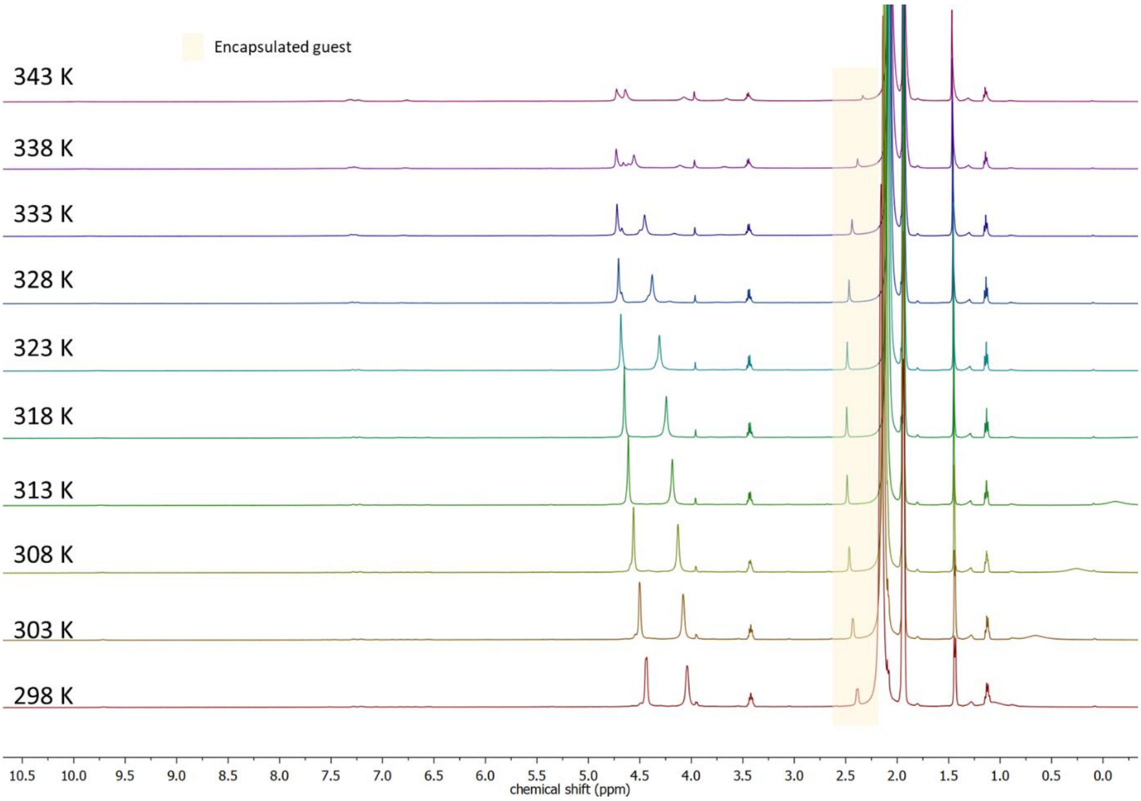

Figure S89 VT ^{1}H NMR spectra of cyclohexane⊂**1** in the diamagnetic region (CD_3CN, 500 MHz, from 298 K to 343 K).

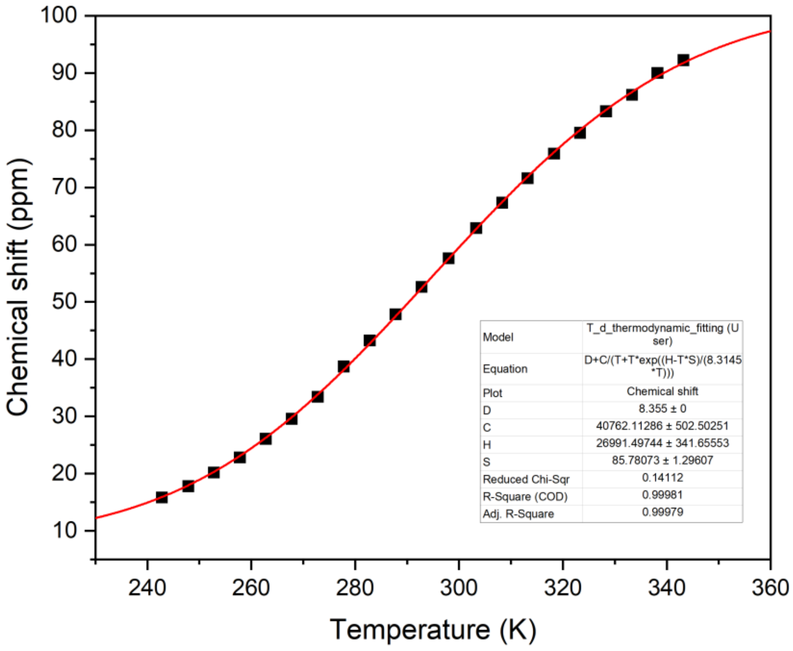

Figure S90 ^{1}H NMR chemical shifts of the imine proton H_a of cyclohexane⊂**1** as a function of temperature in CD_3CN (■, Figures S81, S82) fitted according to Equation S29 (—).

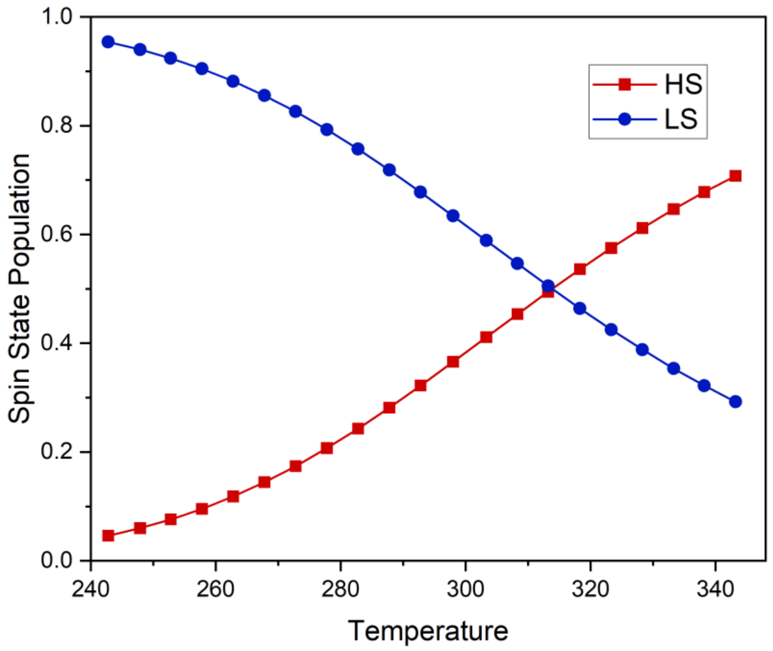

Figure S91 Spin state population of cyclohexane⊂**1** in CD_3CN calculated according to Equations S30 and S31.

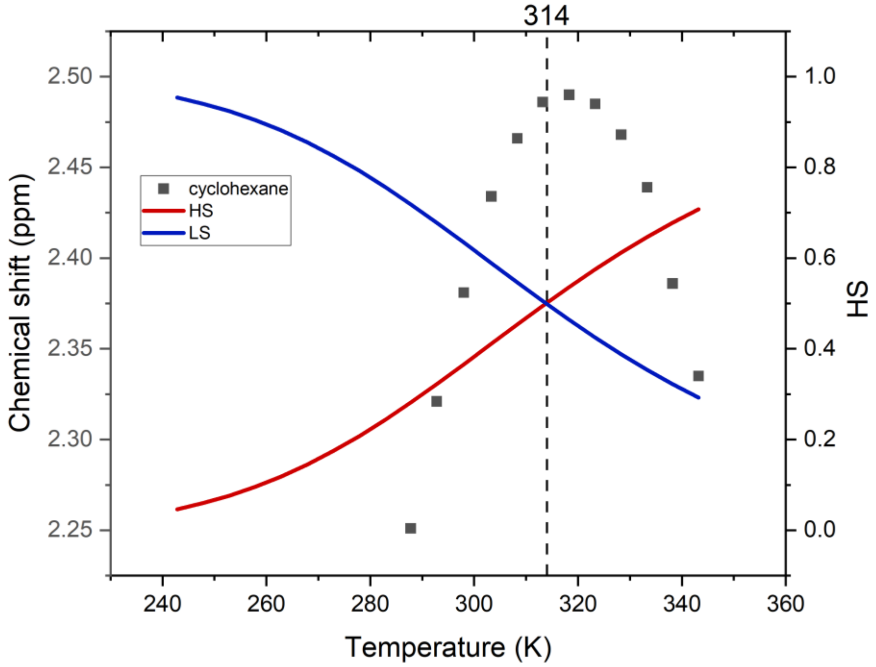

Figure S92 Chemical shifts of encapsulated cyclohexane (black squares) and spin state population of cyclohexane⊂**1** (blue and red curves) in the investigated temperature range in CD_3CN. The SCO $T_{1/2}$ is given as a vertical line.

5.5 *cis*-Decalin⊂**1**

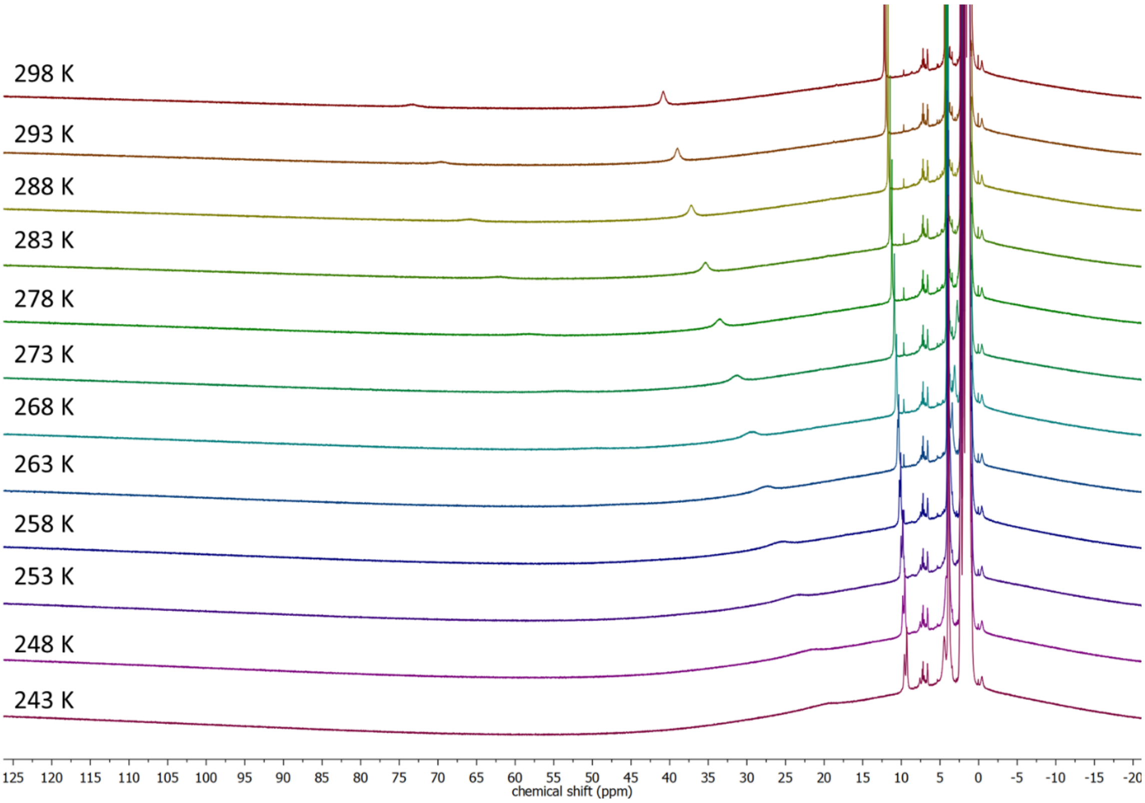

Figure S93 VT ^{1}H NMR spectra of *cis*-decalin⊂**1** (CD_3CN, 500 MHz, from 298 K to 243 K).

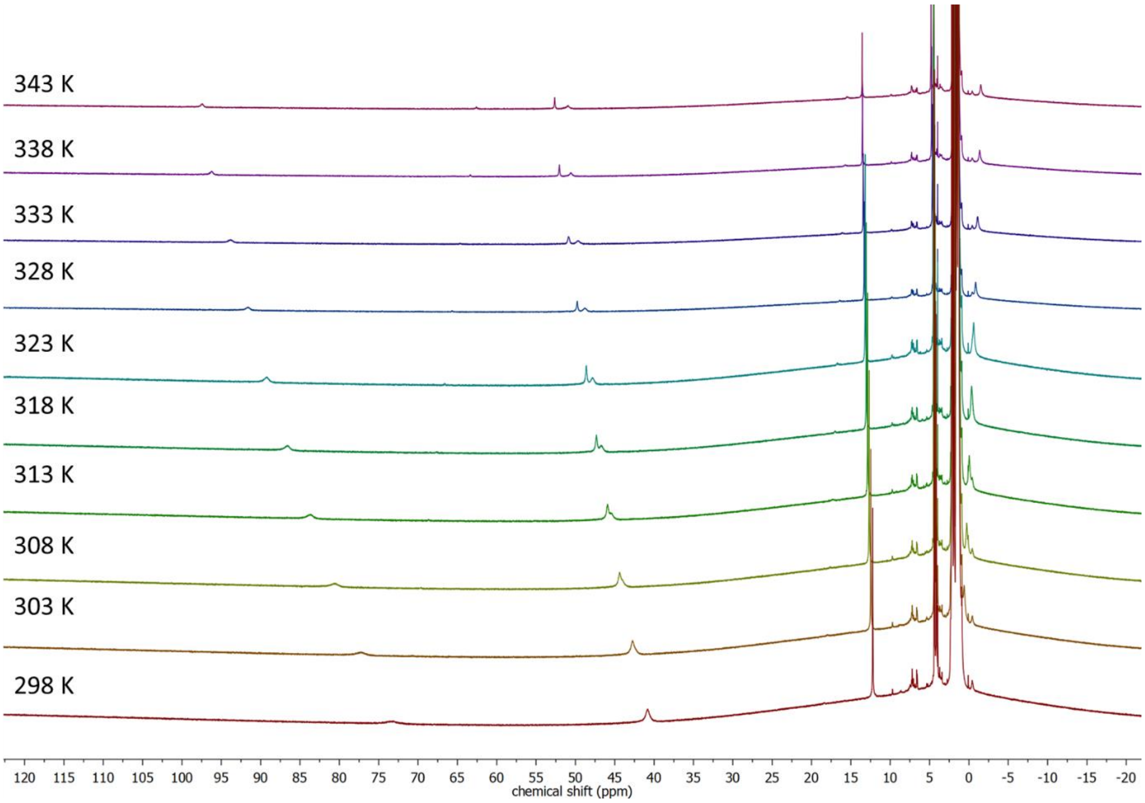

Figure S94 VT ^{1}H NMR spectra of *cis*-decalin⊂**1** (CD_3CN, 500 MHz, from 298 K to 343 K).

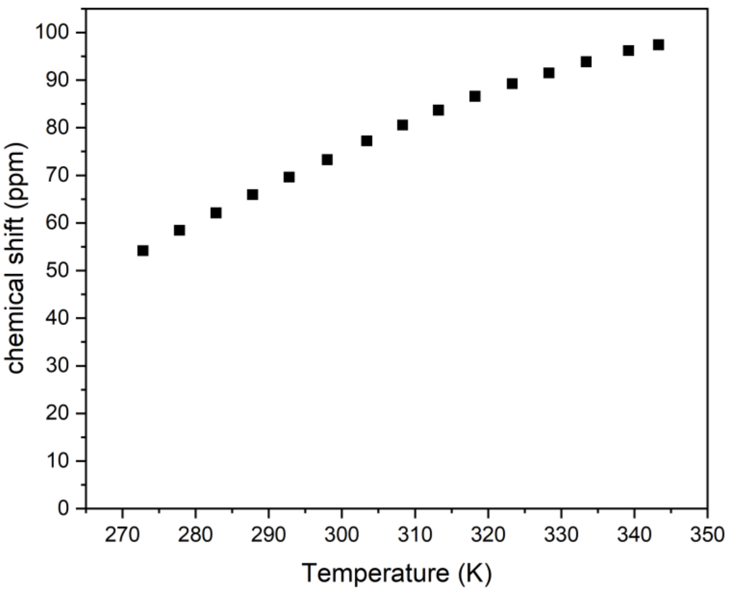

Figure S95 ^{1}H NMR chemical shifts of the imine proton H_a of *cis*-decalin⊂**1** as a function of temperature in CD_3CN (Figures S85, S86). The imine signals become too broad to be observed at low temperature and fitting with the available data points at higher temperatures did not provide a good and reasonable fit.

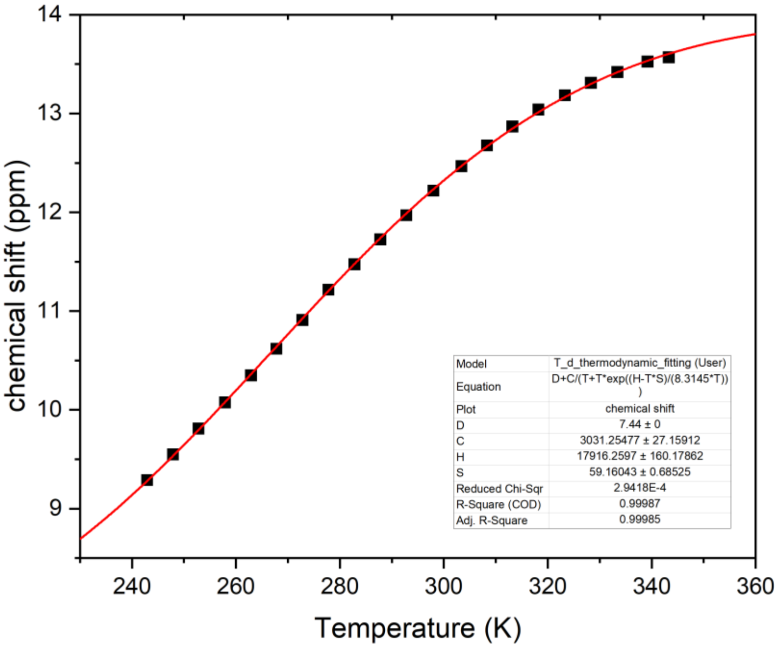

Figure S96 ^{1}H NMR chemical shifts of the phenyl proton H_f of *cis*-decalin⊂**1** as a function of temperature in CD_3CN (■, Figures S85, S86) fitted according to Equation S29 (—).

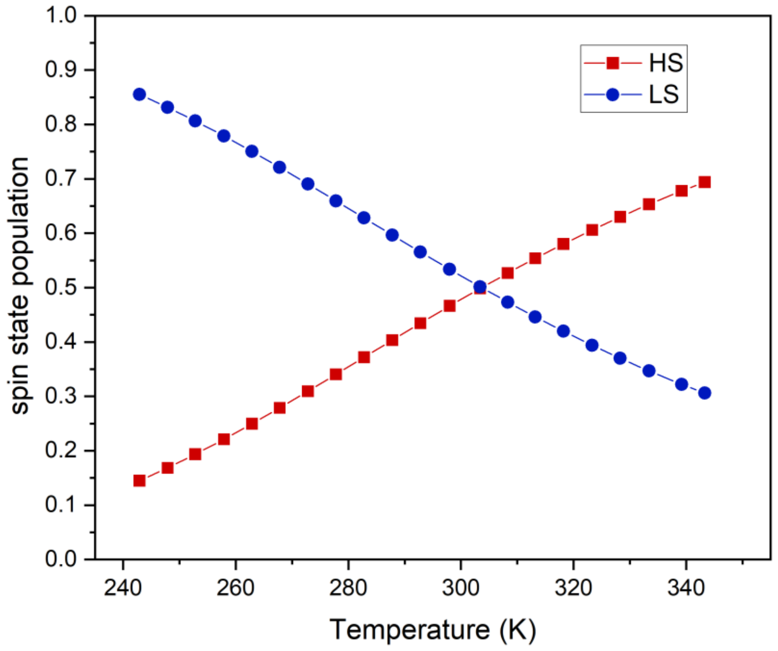

Figure S97 Spin state population of *cis*-decalin⊂**1** in CD_3CN calculated according to Equations S30 and S31.

5.6 Tetrahedron **2**

The behavior of tetrahedron **2** was completely different from that of tetrahedron **1**. At above 273 K, signals corresponding to tetrahedron **2** moved upfield upon heating, displaying HS paramagnetic nature following Curie's law. However, cooling from 273 K also resulted in an upfield shift of the signals, which indicated the loss of paramagnetism. This might be attributed to the SCO process. However, even at the lowest temperature (243 K) in the experiment, the value of the imine proton resonance (H_a) was above 100 ppm, implying that only a small proportion of HS Fe(II) starts to transit into LS. Due to the line broadness at low temperature, only experiments within a small range of temperatures provided usable values of chemical shifts for the model, which was far from enough for a rationalized calculation. Thus, thermodynamic parameters could not be determined. Nonetheless, it can be concluded that tetrahedron **2** preferred the HS state at all investigated temperatures in solution. This is consistent with aldehyde **B** used for assembling tetrahedron **2** offering a weaker ligand field compared to aldehyde **A** used in tetrahedron **1**.

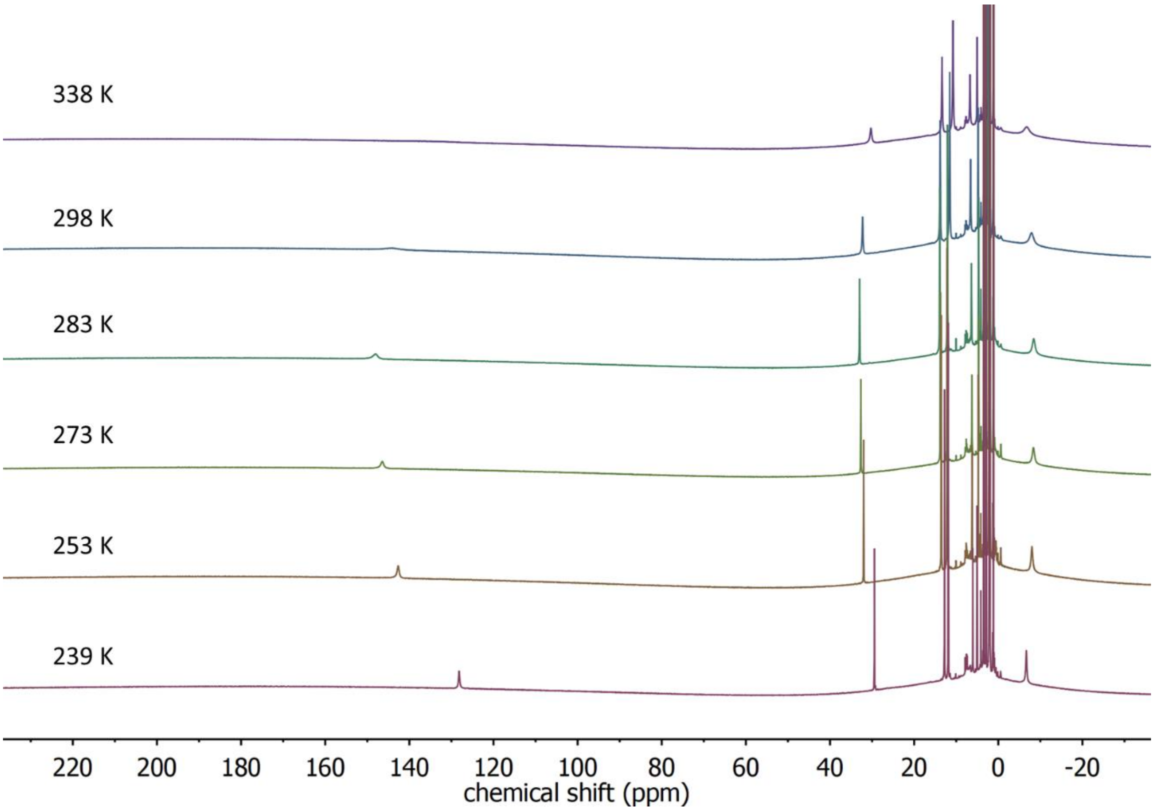

Figure S98 VT ^{1}H NMR spectra of **2** (CD_3CN, 500 MHz, from 239 K to 338 K).

5.7 Adamantane⊂**2**

The encapsulation of adamantane in tetrahedron **2** still gave rise to a HS complex, behaving very similar to **2**. Upfield shift also only started when temperature decreased below 273 K.

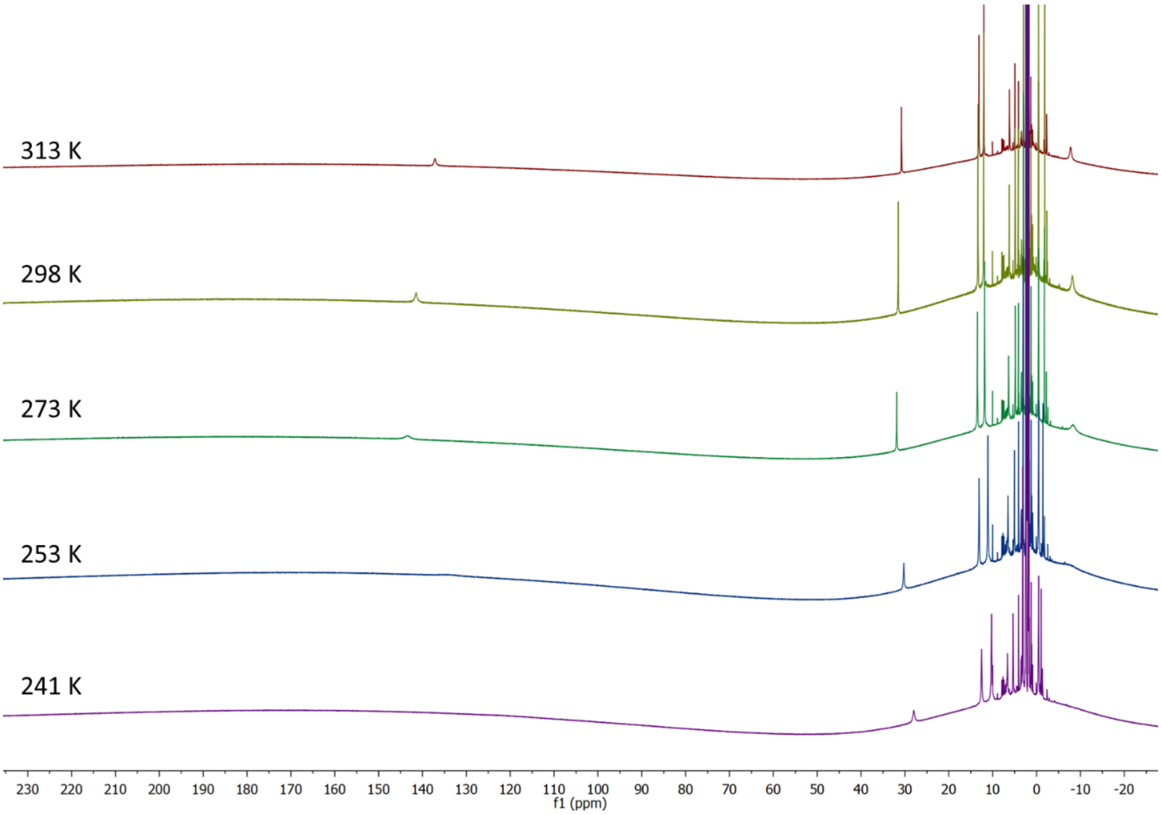

Figure S99 VT ^{1}H NMR spectra of adamantane⊂**2** (CD_3CN, 500 MHz, from 241 K to 313 K).

5.8 Tetrahedron **3**

SCO of tetrahedron **3** was initially studied in CD_3CN as well. However, due to the limit of the boiling point of acetonitrile, data points at higher temperature where more HS population would be reached could not be obtained. Fitting with the same equation (Equation 29) used for tetrahedron **1** didn't converge in the case of tetrahedron **3**. Thus, CD_3CN was replaced by CD_3NO_2 which has similar polarity as CD_3CN but a higher boiling point which allows investigation at higher temperatures than CD_3CN. Similar SCO behaviors were observed in both solvents and the study in CD_3NO_2 led to converged fitting results.

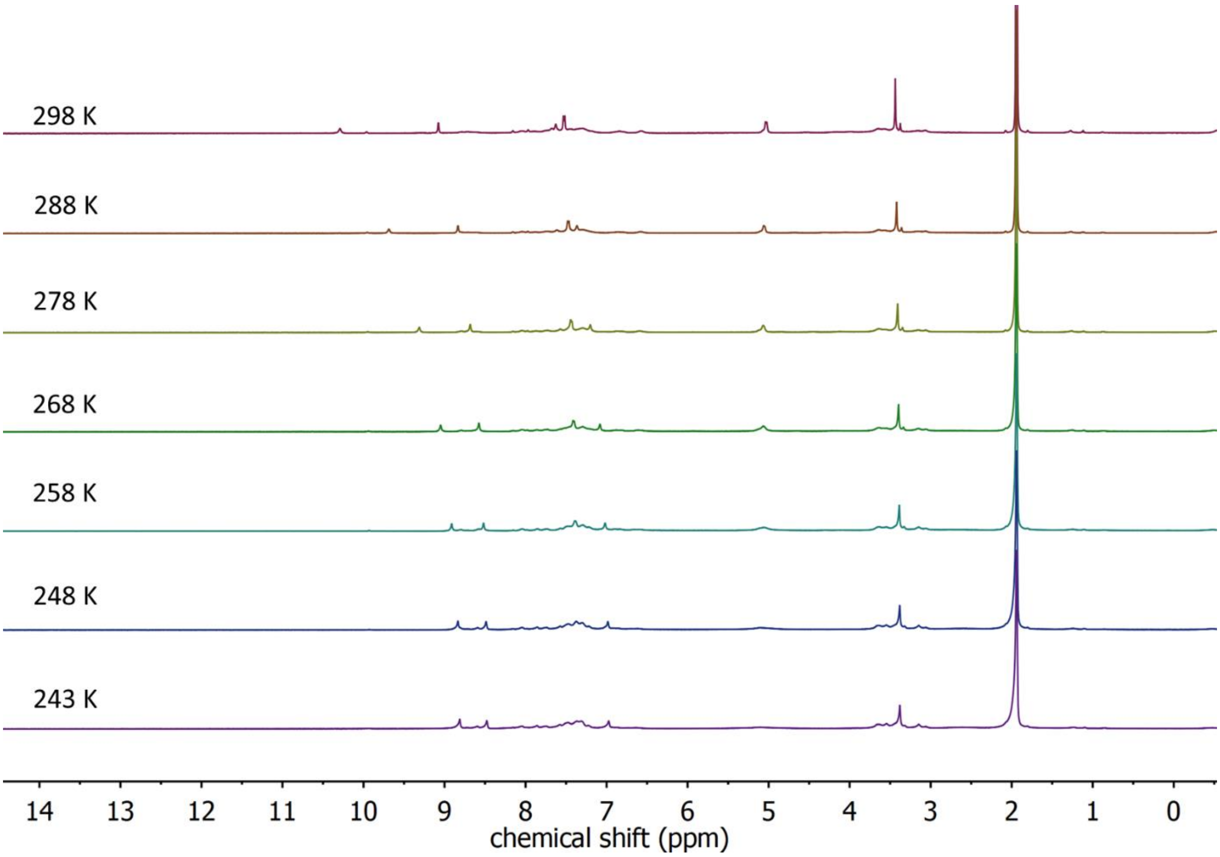

Figure S100 VT ^{1}H NMR spectra of tetrahedron **3** (CD_3CN, 500 MHz, from 298 K to 243 K).

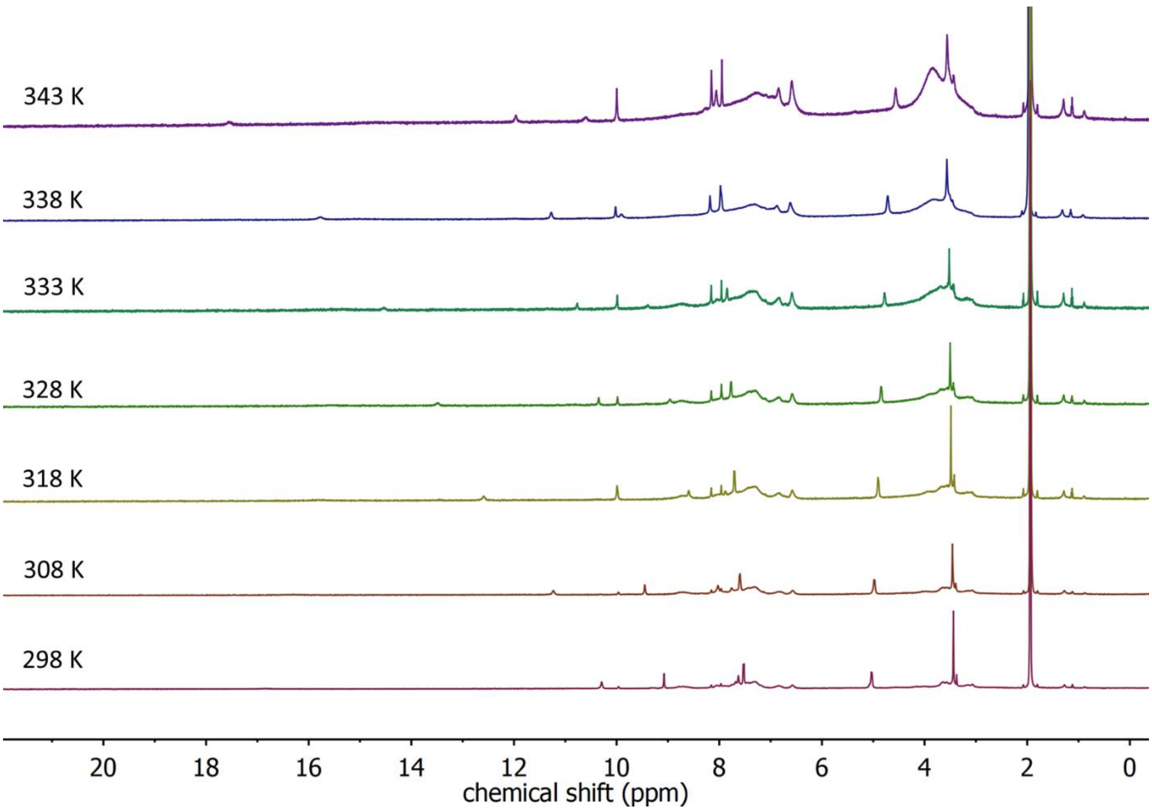

Figure S101 VT ^{1}H NMR spectra of tetrahedron **3** (CD_3CN, 500 MHz, from 298 K to 343 K).

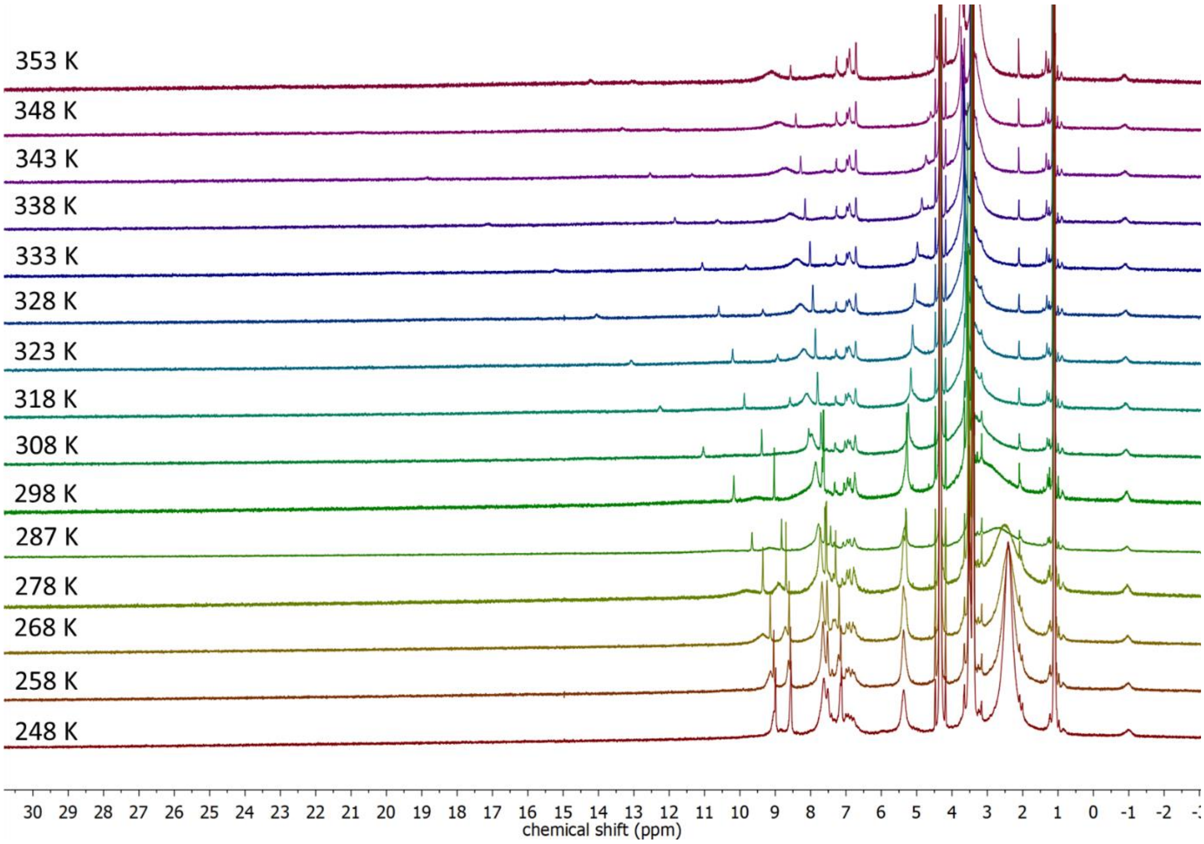

Figure S102 VT ^{1}H NMR spectra of tetrahedron **3** (CD_3NO_2, 500 MHz, from 248 K to 353 K).

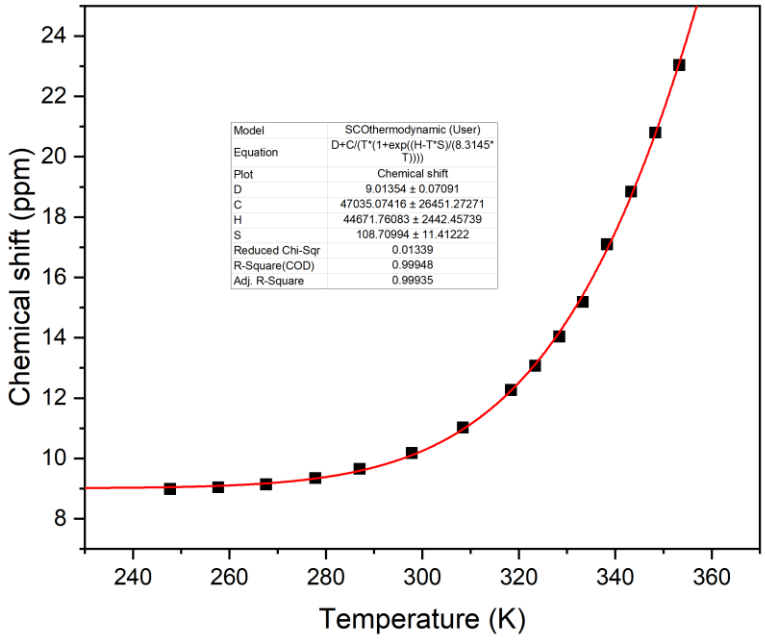

Figure S103 ^{1}H NMR chemical shifts of the imine proton H_a of **3** as a function of temperature in CD_3NO_2 (■, Figure S94) fitted according to Equation S29 (—).

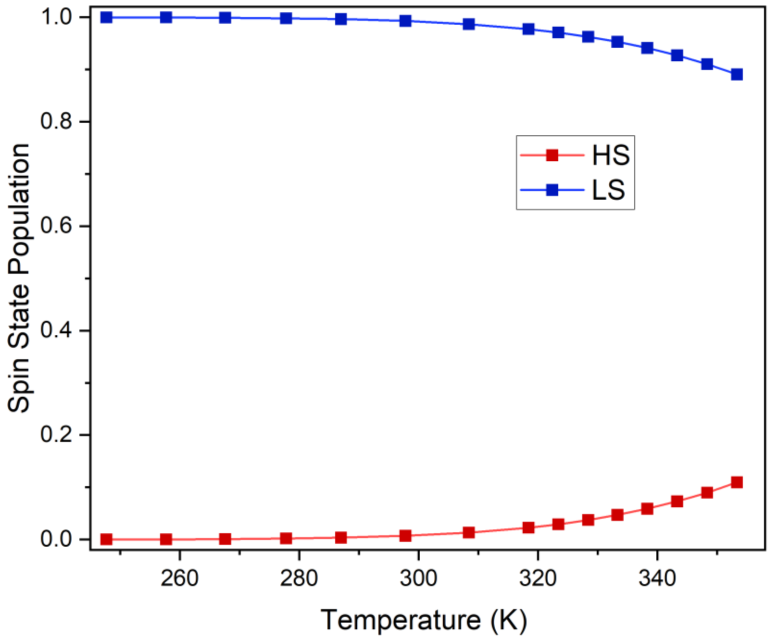

Figure S104 Spin state population of **3** in CD_3NO_2 calculated according to Equations S30 and S31.

5.9 Adamantane⊂**3**

The encapsulation of adamantane did not have much influence on tetrahedron **3**'s SCO behavior. Since tetrahedron **3** was already a mostly LS cage within the investigated temperature range, the cavity volume change was not significant with varying temperature. Furthermore, adamantane preferred the small cavity that LS tetrahedron **3** offers, so the influence of the guest on tetrahedron **3**'s SCO was little.

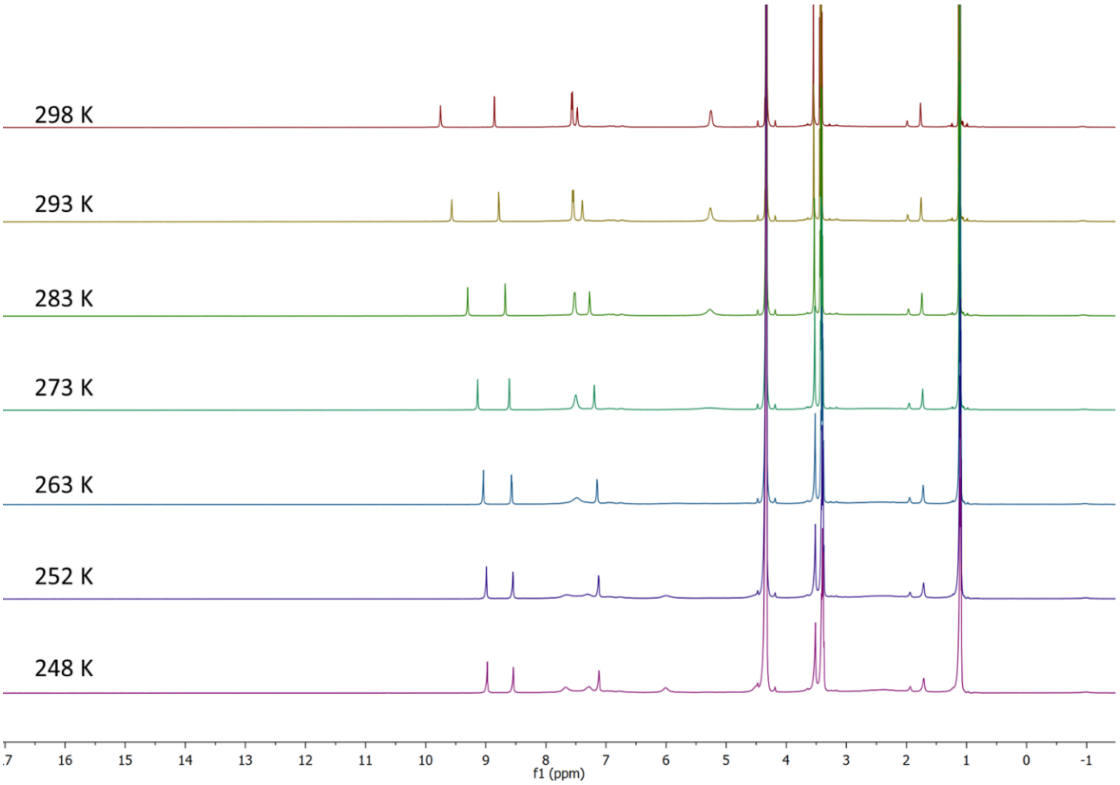

Figure S105 VT ^{1}H NMR spectra of adamantane⊂**3** (CD_3NO_2, 500 MHz, from 248 K to 298 K).

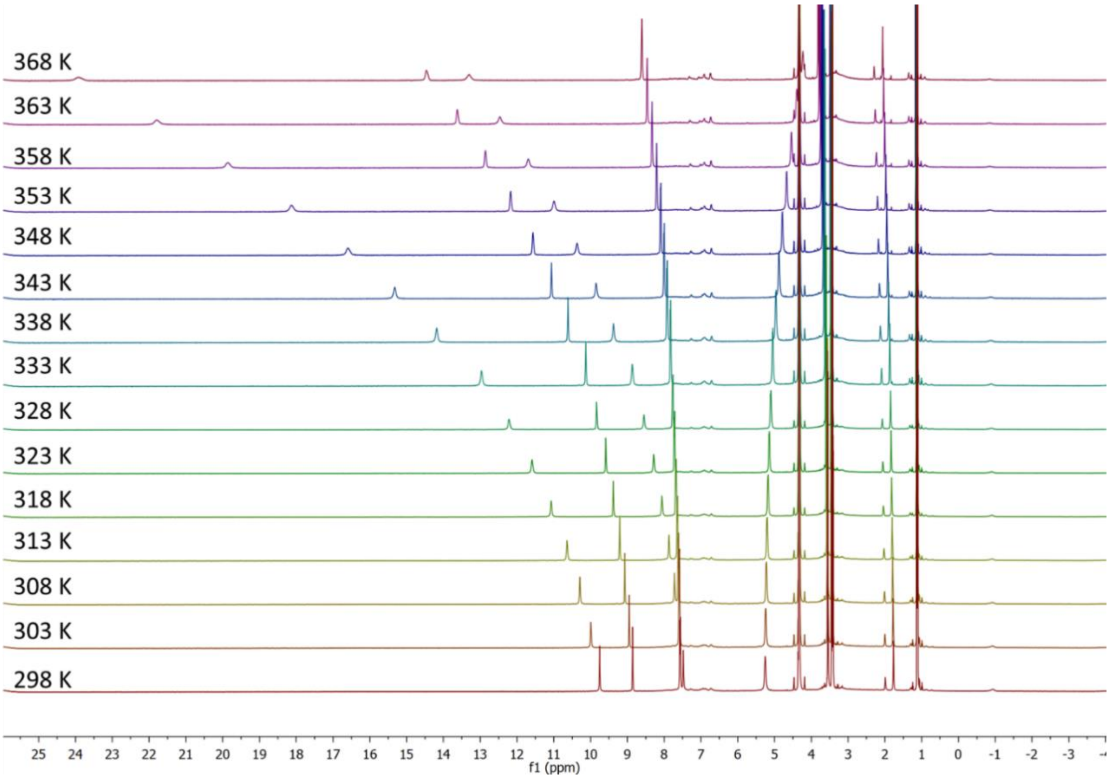

Figure S106 VT ^{1}H NMR spectra of adamantane⊂**3** (CD_3NO_2, 500 MHz, from 298 K to 368 K).

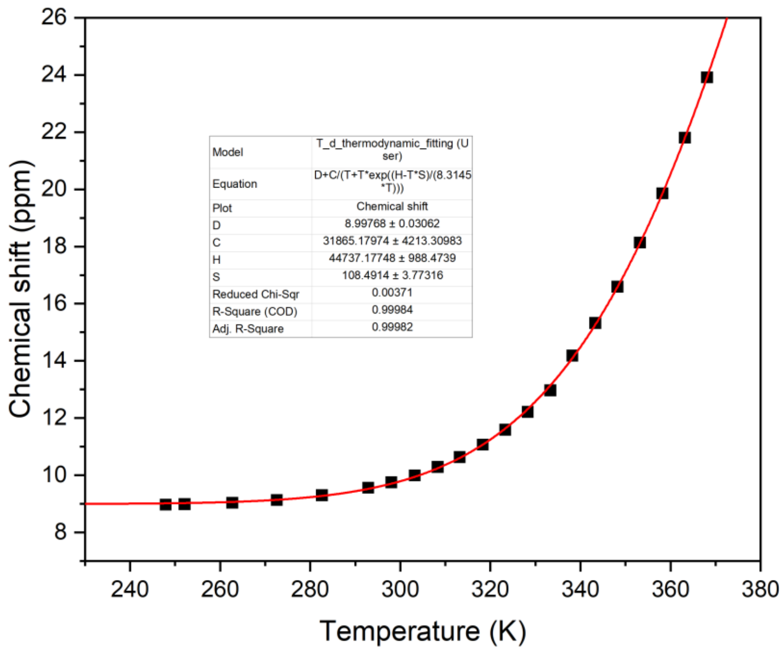

Figure S107 ^{1}H NMR chemical shifts of the imine proton H_a of adamantane⊂**3** as a function of temperature in CD_3NO_2 (■, Figure S98) fitted according to Equation S29 (—).

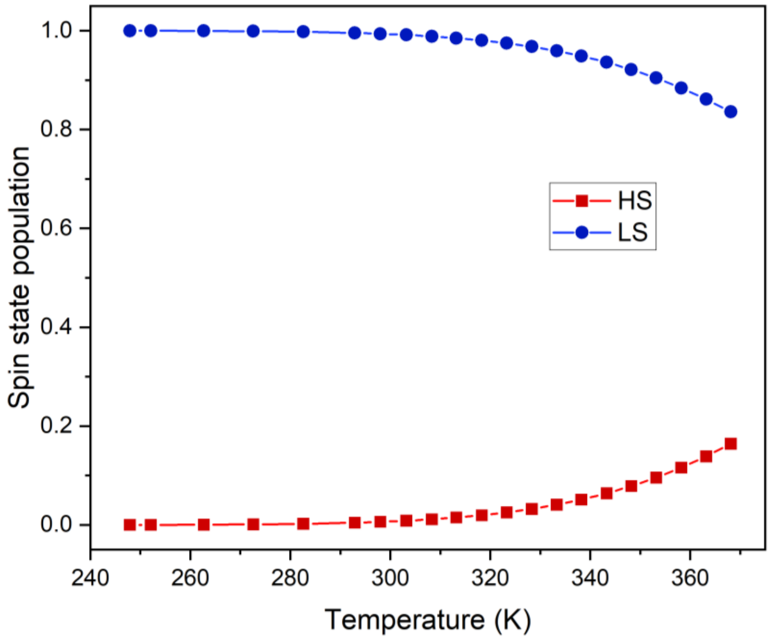

Figure S108 Spin state population of adamantane⊂**3** in CD_3NO_2 calculated according to Equations S30 and S31.

5.10 Cube **4** and trigonal prism **5**

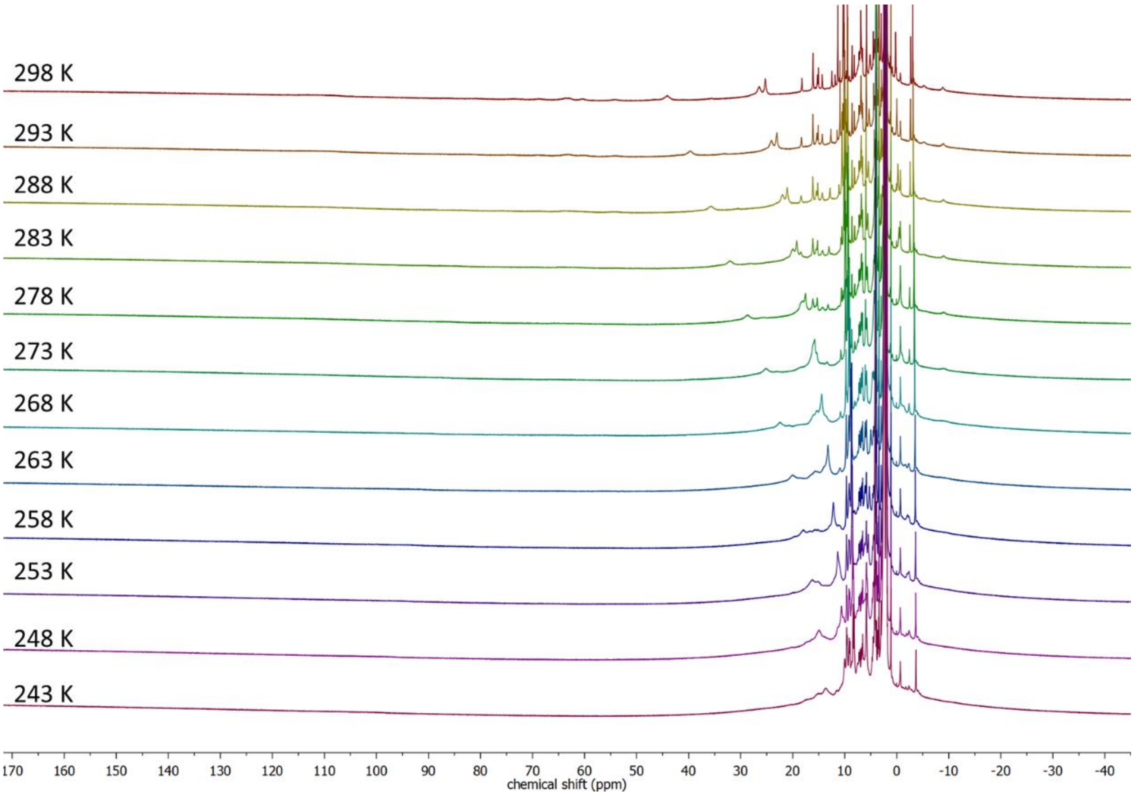

Figure S109 VT ^{1}H NMR spectra of a mixture of **4** and **5**. (CD_3CN, 500 MHz, from 298 K to 243 K).

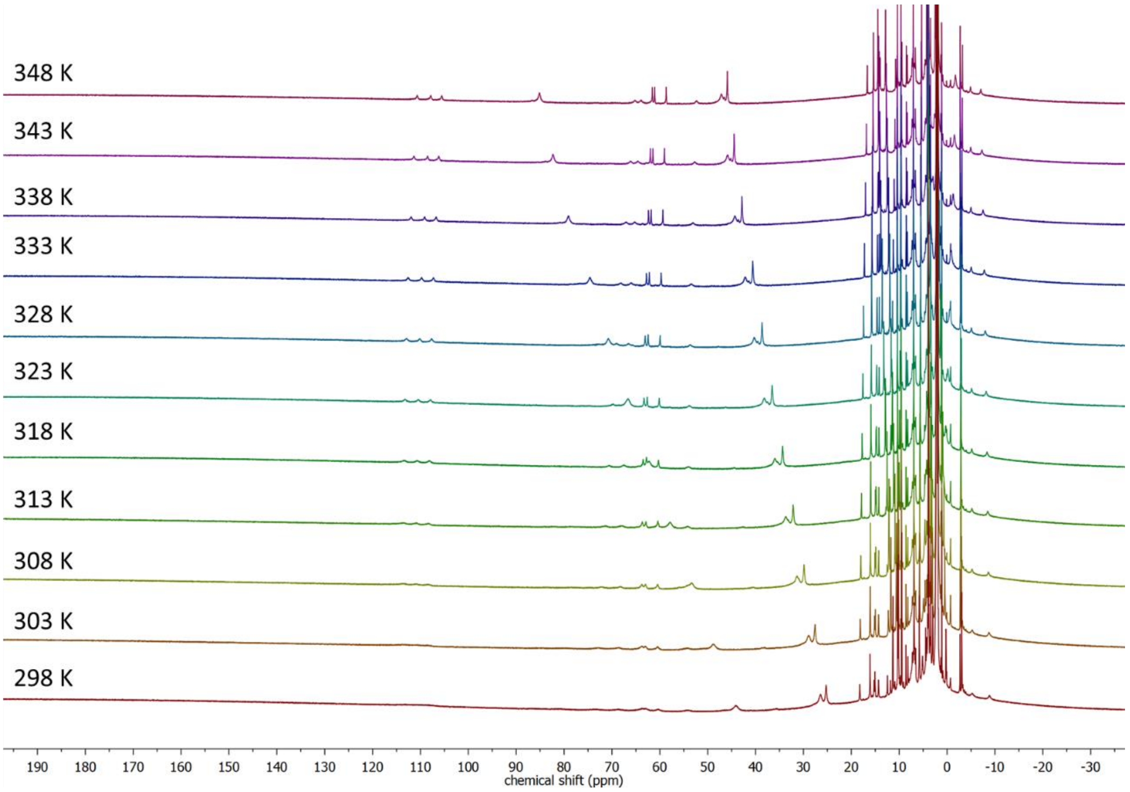

Figure S110 VT ^{1}H NMR spectra of a mixture of **4** and **5**. (CD_3CN, from 298 K to 348 K).

S31.

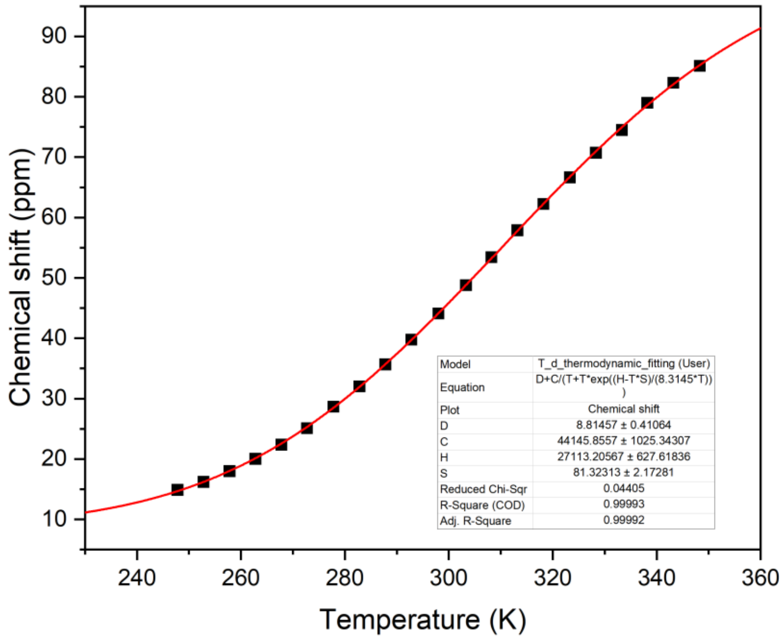

Figure S111 ^{1}H NMR chemical shifts of the imine proton H_a of cube **4** as a function of temperature in CD_3CN (■, Figures S101, S102) fitted according to Equation S29 (—).

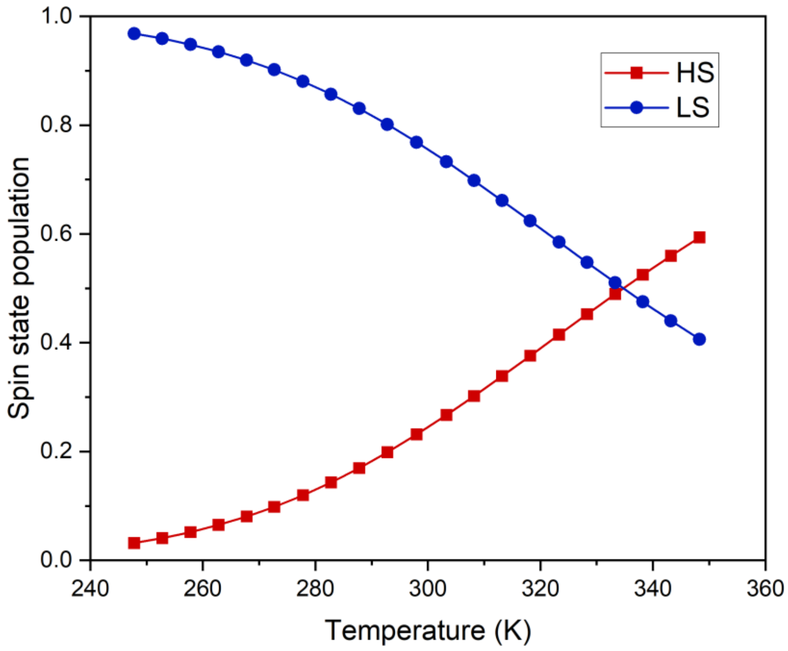

Figure S112 Spin state population of cube **4** in CD_3CN calculated according to Equations S30 and S31.

6 Supplemental References

(1) Sibi, M. P.; Petrovic, G. Enantioselective Radical Reactions: The Use of Metal Triflimides as Lewis Acids. *Tetrahedron Asymmetry* **2003**, *14*, 2879–2882.

(2) Ryan, H. P.; Haynes, C. J. E.; Smith, A.; Grommet, A. B.; Nitschke, J. R. Guest Encapsulation within Surface-Adsorbed Self-Assembled Cages. *Adv. Mater.* **2021**, *33*, 1–6.

(3) Solomon, I. Relaxation Processes in a System of Two Spins. *Phys. Rev.* **1955**, *99*, 559–565.

(4) McConnell, A. J.; Aitchison, C. M.; Grommet, A. B.; Nitschke, J. R. Subcomponent Exchange Transforms an FeII4L4 Cage from High- to Low-Spin, Switching Guest Release in a Two-Cage System. *J. Am. Chem. Soc.* **2017**, *139*, 6294–6297.

(5) Amouri, H.; Mimassi, L.; Rager, M. N.; Mann, B. E.; Guyard-Duhayon, C.; Raehm, L. Host-Guest Interactions: Design Strategy and Structure of an Unusual Cobalt Cage That Encapsulates a Tetrafluoroborate Anion. *Angew. Chem. Int. Ed.* **2005**, *44*, 4543–4546.

(6) Tidmarsh, I. S.; Taylor, B. F.; Hardie, M. J.; Russo, L.; Clegg, W.; Ward, M. D. Further Investigations into Tetrahedral M4L6 Cage Complexes Containing Guest Anions: New Structures and NMR Spectroscopic Studies. *New J. Chem.* **2009**, *33*, 366–375.

(7) Struch, N.; Bannwarth, C.; Ronson, T. K.; Lorenz, Y.; Mienert, B.; Wagner, N.; Engeser, M.; Bill, E.; Puttreddy, R.; Rissanen, K.; Beck, J.; Grimme, S.; Nitschke, J. R.; Lützen, A. An Octanuclear Metallosupramolecular Cage Designed To Exhibit Spin-Crossover Behavior. *Angew. Chem. Int. Ed.* **2017**, *56*, 4930–4935.

(8) Allan, D. R.; Nowell, H.; Barnett, S. A.; Warren, M. R.; Wilcox, A.; Christensen, J.; Saunders, L. K.; Peach, A.; Hooper, M. T.; Zaja, L.; Patel, S.; Cahill, L.; Marshall, R.; Trimnell, S.; Foster, A. J.; Bates, T.; Lay, S.; Williams, M. A.; Hathaway, P. V.; Winter, G.; Gerstel, M.; Wooley, R. W. A Novel Dual Air-Bearing Fixed-χ Diffractometer for Small-Molecule Single-Crystal X-Ray Diffraction on Beamline I19 at Diamond Light Source. *Crystals* **2017**, *7*.

(9) Bruker. SAINT. Bruker AXS Inc: Madison, Wisconsin, USA 2012.

(10) Bruker. Apex 3. Bruker AXS Inc: Madison, Wisconsin, USA 2012.

(11) Collaborative Computational Project, N. 4. The CCP4 Suite: Programs for Protein Crystallography. *Acta Crystallogr. Sect. D Biol. Crystallogr.* **1994**, *50*.

(12) Evans, P. Scaling and Assessment of Data Quality. *Acta Crystallogr. Sect. D Biol. Crystallogr.* **2006**, *62*.

(13) Winter, G. Xia2 : An Expert System for Macromolecular Crystallography Data Reduction. *J. Appl. Crystallogr.* **2010**, *43*.

(14) Farrugia, L. J. WinGX and ORTEP for Windows : An Update. *J. Appl. Crystallogr.* **2012**, *45*.

(15) Dolomanov, O. V.; Bourhis, L. J.; Gildea, R. J.; Howard, J. A. K.; Puschmann, H. OLEX2 : A Complete Structure Solution, Refinement and Analysis Program. *J. Appl. Crystallogr.* **2009**, *42*, 339–341.

(16) Bruker. SADABS. Bruker AXS Inc: Madison, Wisconsin, USA 2001.

(17) Evans, P. R.; Murshudov, G. N. How Good Are My Data and What Is the Resolution? *Acta Crystallogr. Sect. D Biol. Crystallogr.* **2013**, *69*.

(18) Winn, M. D.; Ballard, C. C.; Cowtan, K. D.; Dodson, E. J.; Emsley, P.; Evans, P. R.; Keegan, R. M.; Krissinel, E. B.; Leslie, A. G. W.; McCoy, A.; McNicholas, S. J.; Murshudov, G. N.; Pannu, N. S.; Potterton, E. A.; Powell, H. R.; Read, R. J.; Vagin, A.; Wilson, K. S. Overview of the CCP4 Suite and Current Developments. *Acta Crystallogr. Sect. D Biol. Crystallogr.* **2011**, *67*.

(19) Sheldrick, G. M. SHELXT – Integrated Space-Group and Crystal-Structure Determination. *Acta Crystallogr. Sect. A Found. Adv.* **2015**, *71*.

(20) Sheldrick, G. M. Crystal Structure Refinement with SHELXL. *Acta Crystallogr. Sect. C Struct. Chem.* **2015**, *71*.

(21) van der Sluis, P.; Spek, A. L. BYPASS: An Effective Method for the Refinement of Crystal Structures Containing Disordered Solvent Regions. *Acta Crystallogr. Sect. A Found. Crystallogr.* **1990**, *46*.

(22) Spek, A. L. *PLATON: A Multipurpose Crystallographic Tool.*; Utrecht University: Utrecht, The Netherlands, 2008.

(23) Maglic, J. B.; Lavendomme, R. MoloVol : An Easy-to-Use Program for Analyzing Cavities, Volumes and Surface Areas of Chemical Structures. *J. Appl. Crystallogr.* **2022**, *55*.

(24) Wadell, H. Volume, Shape, and Roundness of Quartz Particles. *J. Geol.* **1935**, *43*, 250–280.

(25) Rios, P.; Carter, T. S.; Mooibroek, T. J.; Crump, M. P.; Lisbjerg, M.; Pittelkow, M.; Supekar, N. T.; Boons, G.-J.; Davis, A. P. Synthetic Receptors for the High-Affinity Recognition of O-GlcNAc Derivatives. *Angew. Chem. Int. Ed.* **2016**, *55*, 3387–3392.

(26) Greenfield, J. L.; Evans, E. W.; Di Nuzzo, D.; Di Antonio, M.; Friend, R. H.; Nitschke, J. R. Unraveling Mechanisms of Chiral Induction in Double-Helical Metallopolymers. *J. Am. Chem. Soc.* **2018**, *140*, 10344–10353.

(27) Schalley, C. A. *Analytical Methods in Supramolecular Chemistry*; Schalley, C. A., Ed.; Wiley: Weinheim, 2012; Vol. 1–2.

(28) Klaeui, W.; Eberspach, W.; Guetlich, P.; Kläui, W.; Eberspach, W.; Gütlich, P. Spin-Crossover Cobalt(III) Complexes: Steric and Electronic Control of Spin State. *Inorg. Chem.* **1987**, *26*, 3977–3982.

(29) De, S.; Tewary, S.; Garnier, D.; Li, Y.; Gontard, G.; Lisnard, L.; Flambard, A.; Breher, F.; Boillot, M.; Rajaraman, G.; Lescouëzec, R. Solution and Solid-State Study of the Spin-Crossover [Fe II (R-bik) 3](BF 4) 2 Complexes (R = Me, Et, Vinyl). *Eur. J. Inorg. Chem.* **2018**, *2018*, 414–428.

(30) Bilbeisi, R. A.; Zarra, S.; Feltham, H. L. C.; Jameson, G. N. L.; Clegg, J. K.; Brooker, S.; Nitschke, J. R. Guest Binding Subtly Influences Spin Crossover in an FeII4L4 Capsule. *Chem. - Eur. J.* **2013**, *19*, 8058–8062.
